# Contrasting evolutionary trajectories of terrestrial vertebrates in the Hengduan Mountains hotspot

**DOI:** 10.1093/nsr/nwaf157

**Published:** 2025-05-02

**Authors:** Chenqi Lu, Wenna Ding, Wei Xu, Quan Li, Shui-Wang He, Fei Wu, Wenjie Dong, Jie-Qiong Jin, Feng Dong, Xue-Long Jiang, Kai Wang, Peng Guo, Robert W Murphy, Ya-Ping Zhang, Jing Che

**Affiliations:** State Key Laboratory of Genetic Evolution & Animal Models, and Yunnan Key Laboratory of Biodiversity and Ecological Conservation of Gaoligong Mountain, Kunming Institute of Zoology, Chinese Academy of Sciences, Kunming 650223, China; Kunming College of Life Science, University of Chinese Academy of Sciences, Kunming 650204, China; Southeast Asia Biodiversity Research Institute, Chinese Academy of Sciences, Yezin, Nay Pyi Taw 05282, Myanmar; CAS Key Laboratory of Tropical Forest Ecology, Xishuangbanna Tropical Botanical Garden, Chinese Academy of Sciences, Mengla 666303, China; Swiss Federal Research Institute WSL, Birmensdorf 8903, Switzerland; State Key Laboratory of Genetic Evolution & Animal Models, and Yunnan Key Laboratory of Biodiversity and Ecological Conservation of Gaoligong Mountain, Kunming Institute of Zoology, Chinese Academy of Sciences, Kunming 650223, China; State Key Laboratory of Genetic Evolution & Animal Models, and Yunnan Key Laboratory of Biodiversity and Ecological Conservation of Gaoligong Mountain, Kunming Institute of Zoology, Chinese Academy of Sciences, Kunming 650223, China; State Key Laboratory of Genetic Evolution & Animal Models, and Yunnan Key Laboratory of Biodiversity and Ecological Conservation of Gaoligong Mountain, Kunming Institute of Zoology, Chinese Academy of Sciences, Kunming 650223, China; State Key Laboratory of Genetic Evolution & Animal Models, and Yunnan Key Laboratory of Biodiversity and Ecological Conservation of Gaoligong Mountain, Kunming Institute of Zoology, Chinese Academy of Sciences, Kunming 650223, China; State Key Laboratory of Genetic Evolution & Animal Models, and Yunnan Key Laboratory of Biodiversity and Ecological Conservation of Gaoligong Mountain, Kunming Institute of Zoology, Chinese Academy of Sciences, Kunming 650223, China; Kunming College of Life Science, University of Chinese Academy of Sciences, Kunming 650204, China; State Key Laboratory of Genetic Evolution & Animal Models, and Yunnan Key Laboratory of Biodiversity and Ecological Conservation of Gaoligong Mountain, Kunming Institute of Zoology, Chinese Academy of Sciences, Kunming 650223, China; Southeast Asia Biodiversity Research Institute, Chinese Academy of Sciences, Yezin, Nay Pyi Taw 05282, Myanmar; State Key Laboratory of Genetic Evolution & Animal Models, and Yunnan Key Laboratory of Biodiversity and Ecological Conservation of Gaoligong Mountain, Kunming Institute of Zoology, Chinese Academy of Sciences, Kunming 650223, China; State Key Laboratory of Genetic Evolution & Animal Models, and Yunnan Key Laboratory of Biodiversity and Ecological Conservation of Gaoligong Mountain, Kunming Institute of Zoology, Chinese Academy of Sciences, Kunming 650223, China; State Key Laboratory of Genetic Evolution & Animal Models, and Yunnan Key Laboratory of Biodiversity and Ecological Conservation of Gaoligong Mountain, Kunming Institute of Zoology, Chinese Academy of Sciences, Kunming 650223, China; Southeast Asia Biodiversity Research Institute, Chinese Academy of Sciences, Yezin, Nay Pyi Taw 05282, Myanmar; Faculty of Agriculture, Forestry and Food Engineering, Yibin University, Yibin 644007, China; State Key Laboratory of Genetic Evolution & Animal Models, and Yunnan Key Laboratory of Biodiversity and Ecological Conservation of Gaoligong Mountain, Kunming Institute of Zoology, Chinese Academy of Sciences, Kunming 650223, China; Centre for Biodiversity and Conservation Biology, Royal Ontario Museum, Toronto ON M5S 2C6, Canada; State Key Laboratory of Genetic Evolution & Animal Models, and Yunnan Key Laboratory of Biodiversity and Ecological Conservation of Gaoligong Mountain, Kunming Institute of Zoology, Chinese Academy of Sciences, Kunming 650223, China; State Key Laboratory of Genetic Evolution & Animal Models, and Yunnan Key Laboratory of Biodiversity and Ecological Conservation of Gaoligong Mountain, Kunming Institute of Zoology, Chinese Academy of Sciences, Kunming 650223, China; Southeast Asia Biodiversity Research Institute, Chinese Academy of Sciences, Yezin, Nay Pyi Taw 05282, Myanmar

**Keywords:** biogeography, evolution, biodiversity, colonization, speciation

## Abstract

The Hengduan Mountains (HDM) harbor the richest temperate diversity in the Northern Hemisphere, yet our understanding of how this exceptionally diverse biota evolved remains obscure. Large-scale historical biogeographic analyses of 851 terrestrial vertebrate species and their relatives (totaling 4862 species) reveal that multiple evolutionary pathways formed this biodiversity hotspot. Whereas *in situ* speciation dominates in amphibians and non-avian reptiles, near-equal *in situ* speciation and colonization occurs in mammals, and colonization happens primarily in birds. HDM are a ‘cradle’ for neo-endemics and a ‘sink’ receiving surrounding biotas, mostly (>30%) coming from the Indo-Malay region. Orogenesis and monsoon intensification triggered *in situ* speciation initiated in the early Oligocene and peaking around 7–8 Ma. Analyses of different taxonomic groups reveal contrasting evolutionary processes and how major geo-climatic events override taxon-specific attributes. Results highlight the need to incorporate taxon-specific traits into future conservation planning to effectively address the unique needs and challenges of different groups.

## INTRODUCTION

Biodiversity is disproportionately concentrated in mountains, which stand out as centers of extraordinary diversity and endemism [1–3]. The synergistic effects of long-term geological processes and climate change drive mountain biodiversity, which ultimately arise through the dynamics of colonization, *in situ* speciation, and extinction [4–7]. These processes likely vary among taxa due to species’ biological and ecological differences [8,9]. The links between evolutionary processes, paleoenvironmental events, and taxon-specific traits have rarely been explored simultaneously within a comprehensive framework. To understand the formation of mountain biodiversity hotspots, it is pivotal to uncover the tempo and mode of various species and biotic assemblies, and their relationship to geo-climatic dynamics [4,5,7], which could effectively inform us on how to mitigate against the threats to species under global climate change.

**Figure 1.**
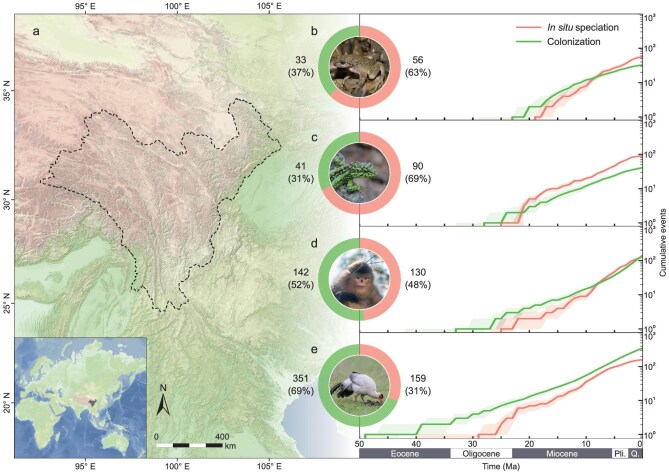
Location of Hengduan Mountains and assembly of four terrestrial vertebrate taxa through time. (a) Topographic map of Hengduan Mountains modified from [44]. Lower left inset shows the global location of the Hengduan Mountains. Faunal assemblies of amphibians (b), reptiles (c), mammals (d) and birds (e) by *in situ* speciation and colonization through time. Solid lines indicate median value and shaded regions indicate the 25% to 75% quantile intervals estimated from 1000 replicate simulations of biogeographic histories. Donut charts display the contribution proportions of *in situ* speciation and colonization to the biotic assembly of each taxon. Embedded species images showcase representative species from the region: *Scutiger nyingchiensis* (Amphibia), *Diploderma iadinum* (Reptilia), *Rhinopithecus bieti* (Mammalia), and *Crossoptilon crossoptilon* (Aves). Images courtesy of Kai Wang and Chencan Liao. Pli., Pliocene; Q., Quaternary. Review drawing number: GS京 (2025)1005号.

The Hengduan Mountains (HDM) in southwestern China are one of the world's most notable biodiversity hotspots [1,10] (Fig. 1a). The mountains consist of a series of north-south oriented ranges with striking alternating ridges and valleys. This complexity features profound topographic relief and rugged landscapes, and a concomitant variety of diverse habitats [10] (Fig. 1a). The geophysical dynamics of this region reflect a complex, but not fully understood landscape evolution. Notwithstanding, recent fossil and tectonic evidence indicate that part of the HDM attained near-modern elevations at the end of the Eocene [11] and underwent a period of rapid and recent uplift from the middle Miocene to the Pliocene [12,13]. Unlike most hotspots located within the tropics or Mediterranean climate zones [1], the HDM harbor the richest temperate flora and fauna in the Northern Hemisphere [1,10]. This includes ∼12 000 species of vascular plants and over 1000 terrestrial vertebrates, with an endemism proportion of ∼30% [1,10]. On a broader geographic scale, the HDM not only sit at the junction of the Palearctic and Oriental zoogeographic realms [14], but also connect the Himalayan and Indo-Burmese biodiversity hotspots [1]. The remarkable richness and endemism have been documented since early 19th-century explorations [10], yet our understanding about the spatiotemporal evolution of its exceptionally diverse biota remains poorly understood.

Studies on the floristic evolution in the HDM have demonstrated the important role played by *in situ* speciation in the assembly of the richest temperate flora [15,16]. By contrast, colonization appears to primarily drive the high species richness of mammals [17]. Thus, a biodiversity hotspot may result from taxa-specific evolutionary pathways [18]. The underlying drivers of the HDM diversity and how they affect the evolutionary dynamics of taxa remain poorly understood. To what extent does taxonomic grouping underlie this divergence? Can major geological and climatic events override taxon-specific attributes, leading to the synchronous evolution among taxa?

To address these questions, we reconstruct the evolutionary histories of birds, mammals, non-avian reptiles (reptiles), and amphibians using a newly compiled comprehensive dataset and quantify the faunal assemblies of the HDM. Cross-taxonomic biogeographic analyses focus on comparisons between diverse groups to explore if each group manifests a distinct evolutionary trajectory owing to their inherent biological and ecological characteristics. We subsequently synthesize data from the different groups to discern the tempo and mode of the total biotic assembly. Finally, we investigate the role that historical environmental changes play in shaping its biodiversity. Reconstructed biogeographic histories of time-calibrated phylogenies for 851 HDM species serve to test the null hypothesis that clade attributes lead to divergent evolutionary trajectories. By employing the inferred biogeographic events, we estimate diversity accumulation, including rates of *in situ* speciation and colonization, which allows us to uncover the dynamics of spatiotemporal evolution, and relate these phenomena to taxonomic characteristics and geo-climatologic events.

## RESULTS

### Data compilation and analyses

As of December 31, 2022, 1160 species of terrestrial vertebrates have been recorded in the HDM, including 97 amphibians, 132 reptiles, 293 mammals, and 638 birds (Table S1). Our analysis dataset encompassed 4862 HDM-associated species in 121 clades, among which 851 species occurred within the HDM, representing >70% of the terrestrial vertebrates in the region (Tables S1 and S2). These data included 16 clades of amphibians (73 species; representing 75.3% of the total number of Hengduan amphibians), 17 clades of reptiles (95; 72.0%), 40 clades of mammals (225; 76.8%), and 48 clades of birds (458; 71.8%) (Table S2). By extracting from published mega-phylogenies (87 clades) and constructing new phylogenies (34 clades), we generated clade-specific, time-calibrated phylogenies (Fig. S1, Dataset S1). Using the cladogenetic state change speciation extinction (ClaSSE) model, we reconstructed biogeographic histories across 121 clades (Figs S2–S122). Through analysis of the diverse combinations of clades, we inferred the process by which various taxa and the entire terrestrial vertebrate fauna of the HDM were assembled through time (Figs 1–4, Figs S123–S127, Tables S3–S8).

**Figure 2.**
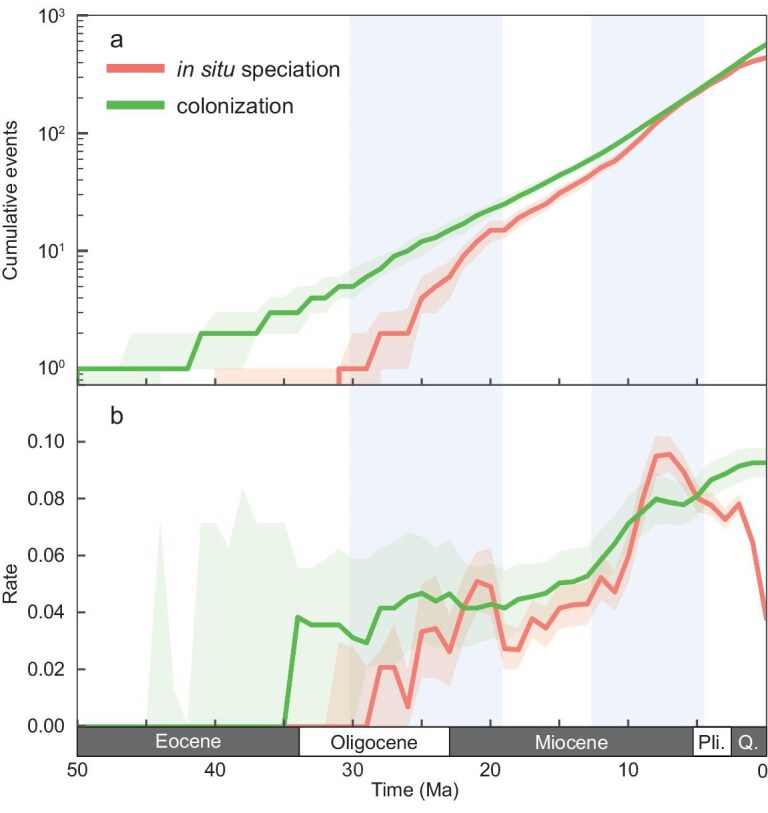
Assembly of terrestrial vertebrate fauna in the Hengduan Mountains. (a) Assembly of terrestrial vertebrate fauna by *in situ* speciation and colonization through time. (b) Rolling estimates of per-capita rate through time. Solid lines indicate median value and shaded regions indicate the 25% to 75% quantile intervals estimated from 1000 replicate simulations of biogeographic histories. Blue highlighting shows two bursts of *in situ* speciation.

**Figure 3.**
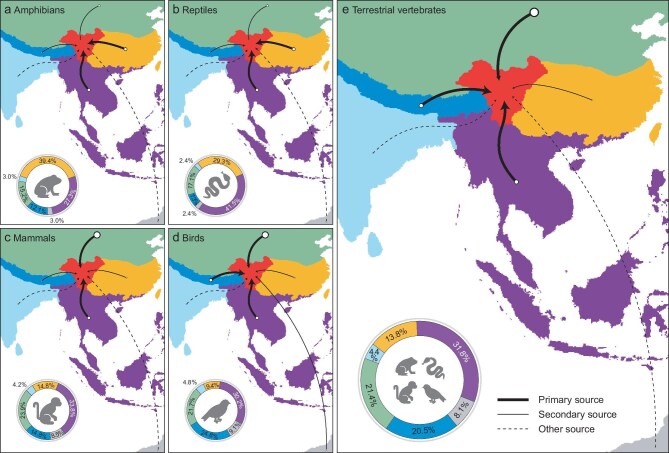
Summary of colonization routes of terrestrial vertebrates and each taxon in the Hengduan Mountains. Number of colonization events from each region indicate median values estimated from 1000 replicate simulations of biogeographic histories. Width and shape of lines represent the relative numbers of colonization events. Inset donut charts show the contribution proportions of colonization events from each region. Review drawing number: GS京 (2025)1005号.

**Figure 4.**
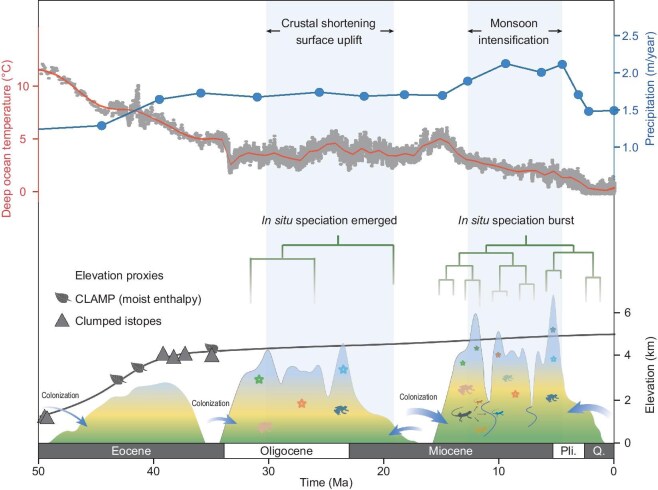
The evolution of biota in relation to climate and geological history in the Hengduan Mountains. Upper: Evolution of global climate is represented by deep-sea oxygen isotopes [45] and estimated deep ocean temperatures (red) [46]. Monsoon conditions are indicated by the modeled mean annual precipitation at idealized CO_2_ levels (blue) (modified from [39]). Lower: Schematic representation of Hengduan Mountains topography and biodiversity formation involving vertebrates and flora [15,16] in three phases, from the Late Eocene to the present. Multiproxy paleoelevation history based on Climate Leaf Analysis Multivariate Program (CLAMP) and clumped isotopes in eastern Xizang (present-day Hengduan Mountains) (modified from [38]). The phylogenetic diagram indicates the relative number of *in situ* speciation events. Arrow widths reflect relative number of colonization events. Pli., Pliocene; Q., Quaternary.

### Tempo and mode of biotic assembly

Reconstructions of biogeographic histories of selected clades revealed contrasting histories of biotic assemblies across taxonomic groups (Fig. 1b–e, Fig. S127, Table S3). For both amphibians and reptiles, *in situ* speciation was the dominant mode of species accumulation, contributing 63% (56 out of 89 inferred biogeographic events; all values represent the median estimated from the 1000 replicate simulations of biogeographic histories) and 69% (90/131) of all inferred biogeographic events, respectively (Fig. 1b and c, Table S3). The rate of *in situ* speciation in amphibians increased during the mid-Miocene, reaching its first peak ∼7 million years ago (Ma) (Fig. S127a). Concurrently, the cumulative number of *in situ* speciation surpassed that of colonization, and this gap has continued to widen ever since, especially during a second burst within the last 5 Ma. (Fig. 1b, Fig. S127a, Table S4). In reptiles, the shift in dominance between *in situ* speciation and colonization occurred earlier but the dominance of colonization in the early stage was insignificant (Fig. 1c, Table S5). The *in situ* speciation rate of reptiles exhibited a bimodal-like pattern, with the initial burst occurring ∼20 Ma (Fig. S127b), surpassing the contribution of colonization to establish dominance (Fig. 1b, Table S5). This was followed by a subsequent burst within the last 10 Ma (Fig. S127b). While the colonization rates of both amphibians and reptiles have generally remained stable, a recent declining trend was detected, particularly within the last 5 Ma (Fig. S127a and b). By contrast, the origin and diversification of mammals and birds showed somewhat distinct trajectories (Fig.1d and e). In mammals, colonization and *in situ* speciation played comparative roles in the assembly of the biota, albeit colonization had a very slight advantage (142 colonization versus 130 *in situ* speciation; Fig. 1d, Table S3). Especially in the last 10 Ma, the contributions of the two events became closely intertwined due to the surge of *in situ* speciation (Fig. 1d, Fig. S127c, Table S6). However, since the Miocene (∼20 Ma), the colonization rate consistently increased, eventually with colonization slightly overtaking *in situ* speciation (Fig. 1d, Fig. S127c, Table S6). The dominant mode of species accumulation in birds underwent a radical shift; colonization contributed to the avian assembly more than twice as much as *in situ* speciation (351 versus 159; Fig. 1e, Table S3). Two bursts of *in situ* speciation in the early and late Miocene narrowed the gap between it and colonization, but colonization still dominated, persisting across the entire history of accumulation for birds (Fig. 1e, Fig. S127d, Table S7).

When combining diverse taxonomic groups, the evolutionary trajectory exhibited reduced fluctuations and more apparent trends (Fig. 2). Both *in situ* speciation and colonization played important roles in shaping the modern terrestrial vertebrate diversity of the HDM, with medians of 566 for *in situ* speciation [56% (566/1003)] and 437 for colonization [44% (437/1003)] (Fig. 2a, Table S3). The onset of colonization occurred around the early Eocene (∼50 Ma; Fig. 2a, Table S8), primarily driven by birds (Fig. 1e). Notably, since its emergence, colonization has consistently been the dominant mode of species accumulation (Fig. 2a, Table S8). Following a phase of relative stability from the Oligocene to early Miocene, the colonization rate gradually increased from the middle Miocene and has maintained an upward trend ever since (Fig. 2b). By contrast, *in situ* speciation occurred later, starting in the early Oligocene (∼31 Ma; Fig. 2a, Table S8). The rate of *in situ* speciation began to increase from the middle Oligocene, with a pulse at the late Oligocene to the early Miocene (∼20–25 Ma), briefly exceeding the colonization rate (Fig. 2b). By the middle Miocene, *in situ* speciation rapidly increased and peaked ∼7–8 Ma, with the per-capita colonization rate of 0.10 species per million years temporarily surpassing colonization once more (Fig. 2b). Despite *in situ* speciation and colonization being detected in the Paleogene, their overall contribution was quite limited. A substantial increase in diversity did not occur until the Miocene, and particularly in the last 10 Ma, with >80% of events taking place unevenly during this period (Table S8).

### Source of colonization

Different taxa exhibited distinctive colonization networks, yet the Indo-Malay region consistently dominated (Fig. 3a–d). Except for amphibians, the Indo-Malay contributed most colonization events of reptiles, mammals, and birds (Fig. 3a–d). Amphibians and reptiles in the HDM showed a high level of congruence in their geographic origins (Fig. 3a and b). The predominant sources of colonization for both groups were Indo-Malay, contributing to 27.3% (9/33) of amphibians and 41.5% (17/41) of reptiles, and South China [39.4% (13/33); 29.3% (12/41), respectively] (Fig. 3a and b). Secondary sources included the Palearctic [15.2% (5/33); 17.1% (7/41), respectively] and Himalayas [12.1% (4/33); 7.3% (3/41), respectively] (Fig. 3a and b). Additionally, sporadic contributions involved India [3.0% (1/33); 2.4% (1/41), respectively] and other regions [3.0% (1/33); 2.4% (1/41), respectively] (Fig. 3a and b). For mammals, northern regions played a greater role than for amphibians and reptiles, with Indo-Malay contributing 33.8% (48/142) and the Palearctic 23.9% (34/142) (Fig. 3c). The Himalayas contributed 14.8% (21/142), South China 14.8% (21/142), and other regions 8.5% (12/142), with India contributing 4.2% (6/142) (Fig. 3c). Birds exhibited multiple sources of colonization, with 30.2% (106/351) coming from Indo-Malay, 24.8% (87/351) from the Himalayas, and 21.7% (76/351) from the Palearctic (Fig. 3d). South China accounted for 9.4% (33/351), and other regions 9.1% (32/351) and, thus, served as secondary sources (Fig. 3d). India contributed merely 4.8% (17/351) of the avifauna (Fig. 3d). Overall, Indo-Malay was the most important source of faunal diversity for the HDM, contributing to 31.8% (180/566) of all colonization events (Fig. 3e). This was ∼1.5 times as many as the second most important source region, the Palearctic contributing 21.4% (121/566), followed by the Himalayas at 20.5% (116/566), South China with 13.8% (78/566), other regions only 8.1% (46/566), and India merely 4.4% (25/566) (Fig. 3e).

## DISCUSSION

### Contrasting evolutionary processes across different taxonomic groups

Our analysis provides a comprehensive portrayal of the spatiotemporal evolutionary dynamics among terrestrial vertebrates in the HDM, encompassing all four terrestrial vertebrate clades. Quantitative assessments reveal the relative contributions of *in situ* speciation and colonization events to the assembly of each taxonomic group (Figs 1–3, Figs S123–S127, Tables S3–S8).

Considering the substantial biological differences among taxa, our findings fail to reject the hypothesis that clade attributes lead to divergent evolutionary trajectories (Fig. 1b–e, Table S3). From intercontinentally mobile birds to amphibians with limited mobility, differences in taxon-specific inherent characteristics, particularly the environmental adaptability and dispersal capabilities, markedly influence the evolutionary trajectories of various taxa (Fig. 1b–e, Table S3). The dominant processes of species accumulation accentuate this difference (Fig. 1b–e, Table S3). Amphibians and reptiles, generally characterized by limited dispersal abilities and low physiological tolerances, typically exhibit more confined and highly fragmented ranges [19,20], which may promote allopatric speciation during geologically active periods, such as orogenesis and concomitant climatic shifts. In these taxa, *in situ* speciation is the dominant process of species accumulation, with relative contributions of 63% and 69% of the total assembly, respectively (Fig. 1b and c, Table S3). By contrast, endothermy endows the physiological capacity to colonize and thrive in a broader range of climatic niches, thereby increasing their chances of successful colonization [21,22]. Accordingly, mammals exhibit a comparatively high number of colonization events relative to *in situ* speciation, and colonization also predominates in birds (Fig. 1d and e, Table S3). Notably, flight abilities in birds tend to inhibit speciation by maintaining genetic cohesion among populations [23], with *in situ* speciation merely contributing 31% of the total avian assembly (Fig. 1e, Table S3).

Biological characteristics are highly heterogeneous even within a group. Correspondingly, our results reveal a consistent relationship between macroevolutionary processes and biological attributes at a finer scale (Figs S123–S126). Mammalian body size closely correlates with dispersal ability, with smaller body sizes restricting dispersal [24,25]. Accordingly, *in situ* speciation contributes more to small mammals, represented by Arvicolinae (voles) and Soricinae (shrews), compared to larger mammals (Fig. S125). Our dataset covers mammalian diversity in the HDM more comprehensively than a previous study [17], particularly regarding small mammals (Table S2). This sampling likely explains why both *in situ* speciation and colonization contribute to diversity almost equally, rather than colonization being overwhelmingly dominant as suggested previously [17]. Similarly, in birds where colonization strongly dominates, three exceptions—Phasianidae (pheasants), Paradoxornithidae (parrotbills), and Leiothrichidae (laughingthrushes)—deviate from the pattern by exhibiting a high number of *in situ* speciation events (Fig. S126). Among them, Phasianidae primarily live on the ground, preferring walking or short-distance flights, and are generally considered a group of birds with weak dispersal abilities [26]. The low dispersal abilities of both Paradoxornithidae and Leiothrichidae are confirmed in the global dataset of avian hand-wing index (HWI), a widely adopted metric for estimating wing shape as a proxy for dispersal abilities in birds [27]. Their mean HWI values, 11.4 and 9.1, respectively, fall far below the global avian average of 25.7 [27].

### Indo-Malay as the major source of the Hengduan Mountains biodiversity

Although different groups exhibit distinct preferences for modes of species accumulation (Fig. 1b–e, Table S3), *in situ* speciation and colonization play comparable roles overall in shaping the terrestrial vertebrate fauna of the HDM (Fig. 2a, Table S3). Our analysis infers 567 *in situ* speciation events and 438 colonization events (Table S3), indicating the dual role the HDM plays as both a ‘cradle’ for neo-endemics and a ‘sink’ receiving various biotas. Our comprehensive dataset covers >70% of the terrestrial vertebrate diversity in the HDM (Table S1), treating groups with different biological traits as equally as possible when data were available. This strategy allows for a more objective reflection of the roles of *in situ* speciation and colonization, helping to mitigate the impact of sampling bias.

Our further analyses quantify the contributions of colonization from adjacent regions revealing that the Indo-Malay region is the most important source of biodiversity for the HDM, accounting for >30% of total colonization events (Fig. 3e). This predominant role the region plays occurs consistently across all four taxonomic groups, regardless of their biological and ecological distinctions (Fig. 3a–d). On the one hand, this could be due to the north-south oriented topology of the HDM with higher elevations in the north, lower elevations in the south, and no significant geographical barriers separating them from the Indo-Malay region (Fig. 1a). The presence of several river valleys further facilitates the connectivity between the mountains and the low-elevation Indo-Malay region, creating potential routes for colonization. On the other hand, older and more diverse regions tend to pump new lineages into less diverse regions [28]. The HDM lineages often nest within and are younger than Indo-Malay lineages (e.g. Figs S14, S25 and S116), which is consistent with the fossil record. For example, fossils from Myanmar and Thailand indicate a diverse mammalian fauna in the Indo-Malay region during the Eocene and Oligocene [29–32].

### Key geo-climate changes drove synchronous evolution of biota in the Hengduan Mountains

The exact timing and formation of the HDM have long been debated due to its highly complex tectonic setting [33]. The formation is often attributed to the rapid uplifting from the late Miocene to the Pliocene [33,34]. However, recent fossil and tectonic evidence suggest that parts of the HDM had reached near-modern elevations by the end of the Eocene [11,13,35]. The regional trans-tensional motion and southeastward extrusion of the Indochina Block during the Eocene to the Miocene continually contributed to the rise of the HDM [36–38] (Fig. 4). Furthermore, the intensification of the Asian summer monsoon since the mid-Miocene [39] has brought abundant rainfall that hastened river erosion [40] (Fig. 4). This is also considered crucial in shaping the current unique landscape of the HDM [40,41].

The intensification of orogenesis and monsoons appears to have jointly triggered the evolution of the terrestrial vertebrate fauna (Figs 2 and 4). The overall *in situ* accumulation began at ∼31 Ma (Fig. 2a, Table S8), coinciding with the development of HDM high topography [11,13,35] (Fig.  4). Continued *in situ* speciation peaked in the late Miocene at ∼7 to 8 Ma (Fig. 2b). These results are temporally consistent with a phase of active crustal deformation [37,41] and the intensification of the Asian summer monsoons during the middle to late Miocene [39], which together facilitated the creation of the modern high relief and rugged topography [38–41] (Fig. 4). The topographically complex landscape then increased isolation and appears to have led to the emergence of specialized habitats, significantly contributing to the development of local endemic groups (Figs 2b and 4). In particular, the secular decline of global temperatures since the middle Miocene coupled with the high terrain of the HDM may have facilitated the expansion of alpine habitats (Fig. 4). Correspondingly, numerous regional endemic alpine taxa or clades experienced rapid diversification since the late Miocene at ∼10 Ma, including *Scutiger* (lazy toads; Fig. S10), *Gloydius* (Asian pit vipers; Fig. S27), *Eothenomys* (oriental voles; Fig. S49), and others. By contrast, the rapid radiation in the *Diploderma* (mountain dragons; Fig. S31), which typically inhabits river valleys and exhibits remarkable diversity and endemism [42], may reflect the formation of a dissected landscape due to the river incision driven by intensified monsoons [40,41] (Fig. 4). The emergence of new habitats and vacant niches also appears to have facilitated an influx of colonizers. While no distinct bursts are evident, the colonization rate consistently shows an upward trend, with >80% of colonization events occurring unevenly over the last 10 Ma (Fig. 2a, Table S8).

Notable imprints of diversification bursts associated with geo-climatic shifts occur in the evolutionary history of all four terrestrial vertebrate groups (Fig. 1b–e, Fig. S127). Despite biological distinctions, these groups exhibit significant signals of diversification bursts ∼7–10 Ma (Fig. S127). Not limited to fauna, similar diversification trajectories have been reported in the highly specialized alpine flora. This flora appeared almost simultaneously in the Oligocene [16], intertwined with the pulse of *in situ* speciation in the fauna during the Oligocene to the early Miocene, indicating the coevolution of the region's distinctive mountainous landscape and its unique biota (Fig. 4). Both alpine and integrated flora exhibited significant acceleration in *in situ* speciation during the middle Miocene [15,16], with alpine flora peaking ∼7 Ma [16]. This resemblance suggests that major geo-climatic events may override taxon-specific attributes, thereby triggering the synchronous evolution of biota (Fig. 4). Although *in situ* speciation rates slowed down after a pulse of rapid diversification ∼7–8 Ma, they remained relatively high before showing a marked decline in the Quaternary (Fig. 2b), suggesting that geophysical changes continued to drive diversification, albeit to a lesser extent than during the earlier period.

Collectively, our study provides a comprehensive cross-taxonomic biogeographic analysis of the spatiotemporal evolution of terrestrial vertebrates in the HDM. By examining four distinct groups, analyses reveal multiple evolutionary processes (Fig. 1b–e, Fig. S127) and elucidate the shared influence of major geo-climatic events, on both vertebrates and flora (Fig. 4). This result offers new insights into the multiple pathways leading to the formation of the HDM biodiversity hotspot. Furthermore, our finding of contrasting evolutionary pathways has critical implications for conservation. It highlights the necessity of integrating taxon-specific attributes into future conservation planning to effectively address and manage the unique needs and challenges of different groups [43].

## Supplementary Material

nwaf157_Supplemental_Files

## MATERIALS AND METHODS

Detailed Materials and Methods are available in the Supplementary Information.

## DATA AND MATERIALS AVAILABILITY

All scripts used in this study have been stored on GitHub at https://github.com/CheLab-KIZ/Paper-codes/tree/main/CJPA202402, along with intermediate analysis files. All other data are included in the manuscript and/or Supplementary Information.
