# Supplementary material for: Contrasting evolutionary trajectories of terrestrial vertebrates in the Hengduan Mountains hotspot
Source: Natl Sci Rev. 2025 May 2;12(8):nwaf157. doi: 10.1093/nsr/nwaf157 (PMC12368496; doi:10.1093/nsr/nwaf157)
Supplement: nwaf157_Supplemental_Files [file nwaf157_supplemental_files.zip › NSR_Supplementary Information_0630.pdf]

Supplementary Information for

Contrasting evolutionary trajectories of terrestrial vertebrates in the Hengduan Mountains hotspot

Author

Chenqi Lu[1, 3, 7, #], Wenna Ding[2, 4, #], Wei Xu[1, #], Quan Li[1], Shui-Wang He[1], Fei Wu[1], Wenjie Dong[1, 3], Jie-Qiong Jin[1, 7], Feng Dong[1], Xue-Long Jiang[1], Kai Wang[1, 7], Peng Guo[5], Robert W. Murphy[1, 6], Ya-Ping Zhang[1, *], Jing Che[1, 7, *]

Affiliation

[1] State Key Laboratory of Genetic Evolution & Animal Models, and Yunnan Key Laboratory of Biodiversity and Ecological Conservation of Gaoligong Mountain, Kunming Institute of Zoology, Chinese Academy of Sciences, Kunming 650223, China
[2] CAS Key Laboratory of Tropical Forest Ecology, Xishuangbanna Tropical Botanical Garden, Chinese Academy of Sciences, Mengla, Yunnan 666303, China
[3] Kunming College of Life Science, University of Chinese Academy of Sciences, Kunming 650204, China
[4] Swiss Federal Research Institute WSL, Birmensdorf 8903, Switzerland
[5] Faculty of Agriculture, Forestry and Food Engineering, Yibin University, Yibin 644007, China
[6] Centre for Biodiversity and Conservation Biology, Royal Ontario Museum, Toronto ON M5S 2C6, Canada
[7] Southeast Asia Biodiversity Research Institute, Chinese Academy of Sciences, Yezin, Nay Pyi Taw 05282, Myanmar

[#] Authors contributed equally to this work

[*] Corresponding authors: Jing Che, Ya-Ping Zhang

Email: chej@mail.kiz.ac.cn; zhangyp@mail.kiz.ac.cn

This PDF file includes:

Supplementary Text
Supplementary Figures S1 to S128
Supplementary Tables S1 to S9

Other supplementary materials for this manuscript include the following:

Dataset S1. GenBank accession numbers for gene sequences used in phylogenetic reconstruction, grouped by clade.

Supplementary Text

MATERIALS AND METHODS

Clade selection and data compilation

Our analyses concentrated on the HDM as defined by Liu, et al. [1], who utilized geological and geomorphological features, as well as the integrity of mountain ecosystems, as defining criteria (Fig. 1a). We reviewed literature on the biodiversity in this region, combined with extensive distributional data accumulated from field surveys and recommendations from experts of each taxon. This culminated in the compilation of a comprehensive checklist of terrestrial vertebrates in the HDM (Table S1).

The selection of HDM-associated clades for analysis followed two criteria: (i) molecular data sufficient to infer a phylogeny that covered most of the taxonomic diversity within this clade, and (ii) availability of suitable calibration data to estimate divergence times. This sampling strategy maximized taxon representation in the HDM. For birds, we focused exclusively on breeding birds due to their relatively stable distributions compared to non-breeding birds, resulting in more consistent responses to environmental factors [2]. Considering the feasibility and efficiency of the analysis, the taxonomic rank of clades was prioritized to genera, and in some cases, extended to subfamilies or families to accommodate monotypic or oligotypic groups.

The phylogenies had two components: (i) available phylogenies of 87 clades extracted from previously published mega-phylogenies, and (ii) new phylogenies for 34 clades constructed herein. We first selected the most comprehensively dated trees available for each taxonomic group up to December 31, 2022, with amphibians sourced from Jetz and Pyron [3], reptiles from Tonini, et al. [4], mammals from Upham, Esselstyn and Jetz [5], and birds from Jetz, et al. [6]. These phylogenies were accessed from VertLife (vertlife.org). We only used clades with both good global sampling (covering at least 30% of the total species of the clade) and HDM sampling (covering at least 60% of the Hengduan species of the clade). R package ape [7] was used to extract relevant clades from a randomly selected tree built using genetic data. Finally, 87 clades, including 425 HDM species, met these criteria.

For the remaining 34 clades (426 HDM species), we constructed new phylogenies. Drawing on previous studies, we selected genes widely used within each clade. For details of the genes used for each clade, see the section on ***Phylogenetic analysis and calibration details for each clade*** below. The complete molecular dataset for phylogenetic analysis included 51 genes, ranging from 2 to 21 genes per clade, totaling 7,383 sequences. All sequences were downloaded from GenBank (through December 2022). GenBank accession numbers are listed in Dataset S1. Phylogenetic analyses and divergence time estimations are given below.

Phylogenetic analysis and divergence time estimation

All sequences were aligned using MAFFT v7.511 [8] via the MAFFT-online server (https://mafft.cbrc.jp/alignment/server). For protein-coding genes (e.g. *Cytb*, *COI*, totaling 48 genes) and the D-loop, with default settings, the appropriate strategy was automatically selected from among FFT-NS-1, FFT-NS-2, FFT-NS-I, or L-INS-i, based on the size of the dataset. Alignments were visually inspected and manually trimmed in Mega X [9] after translation into amino acids, ensuring the maintenance of an open

reading frame. The Q-INS-i strategy was chosen for all ribosomal genes (12S rRNA, 16S rRNA) due to its consideration of RNA secondary structure, resulting in higher accuracy [10]. The highly divergent and poorly aligned regions were removed using GBlocks v0.91b [11].

Multiple loci were concatenated using a custom Python script, generating data blocks corresponding to the full length for each gene. The assembled supermatrices for each clade were used to construct phylogenetic trees under the maximum likelihood criterion, using IQ-TREE v2.2.0.3 [12]. ModelFinder [13] implemented in IQ-TREE was employed for the auto-determination of the best-fit model for all partitions (Table S9), applying the Bayesian information criterion (BIC). One thousand ultra-fast bootstraps [14] and the Shimodaira-Hasegawa approximate likelihood ratio test (SH-aLRT) [15] were performed to evaluate node support statistics. After confirming that the phylogenetic relationships of each clade were consistent with published studies, the tree was used for subsequent analyses.

Time-calibrated trees were constructed using the penalized likelihood method as implemented in treePL v1.0 [16]. Through a comprehensive review of phylogenetic studies that provide temporal frameworks for target clades, we selected the latest and most extensively sampled studies as our source of secondary calibration data. For details of the calibrations used for each clade, see the section on ***Phylogenetic analysis and calibration details for each clade*** below. The optimal maximum likelihood tree inferred from IQ-TREE was used as the reference topology. The “prime” option was applied to identify the best optimization parameters, and a “thorough” analysis was then carried out with the optimal parameters determined above (opt, optad, and optcvad). To identify the best smoothing parameter that affected the penalty for rate variation over the phylogram, a random subsample and replicate cross-validation (RSRCV) was conducted with treePL. Finally, the critical node ages of the 34 newly reconstructed time-calibrated phylogenies in this study were systematically compared with published studies to validate their concordance.

Phylogenetic analysis and calibration details for each clade

(1) *Scutiger* (Megophryidae, Anura, Amphibia)
One nuclear [recombination activating 1 (*RAG1*)] and three mitochondrial genes [16S ribosomal RNA (16S rRNA); cytochrome c oxidase subunit I (*COI)*; cytochrome b (*Cytb*)] were used to construct a dated phylogeny for *Scutiger.* Following the study of Hofmann et al. [17], we designated *Oreolalax omeimontis* (Megophryidae) as the outgroup and used a secondary calibration point of 53.4 Ma [95% highest posterior density (HPD): 39.4–70.5] for the stem age of *Scutiger.*
(2) *Amolops* (Ranidae, Anura, Amphibia)
Three mitochondrial genes [16S rRNA, *COI* and NADH dehydrogenase subunit 2 (*ND2*)] were used to construct a dated phylogeny for *Amolops*. Following the study of Wu et al. [18], we designated *Rana jiemuxiensis* (Ranidae) and *Odorrana jingdongensis* (Ranidae) as outgroups and used secondary calibration points of 32.03 Ma (95% HPD: 27.35–36.56) for the stem age of *Amolops* and 25.01 Ma (95% HPD: 21.31–28.58) for the crown age.
(3) *Lycodon* (Colubridae, Serpentes, Reptilia)
One nuclear [oocyte maturation factor MOS (*C-mos*)] and three mitochondrial genes (16S rRNA, *COI, Cytb*) were used to construct a dated phylogeny for *Lycodon.* We

designated *Boiga kraepelini* (Colubridae) as the outgroup and used 16.8 Ma (95% HPD: 12.2–22.8) as the secondary calibration point for the split age of *Boiga* and *Lycodon* as estimated by Li et al. [19].

(4) *Hebius* (Natricidae, Serpentes, Reptilia)

Three nuclear [*C-mos*, *RAG1*, neurotrophin-3 (*NT3*)] and one mitochondrial gene (*Cytb*) were used to construct a dated phylogeny for *Hebius.* We designated *Trachischium monticola* (Natricidae) and *Trachischium guentheri* (Natricidae) as outgroups and used secondary calibration points of 23.04 Ma for the stem age of *Hebius* and 17.97 Ma for the crown age as estimated in a broad study of Natricidae [20].

(5) *Rhabdophis* (Natricidae, Serpentes, Reptilia)

Two nuclear (*C-mos*, *NT3*) and three mitochondrial genes [16S rRNA, *Cytb*, NADH dehydrogenase subunit 4 (ND4)] were used to construct a dated phylogeny for *Rhabdophis.* We designated *Tropidonophis dahlii* (Natricidae) and *Xenochrophis maculatus* (Natricidae) as outgroups and used a secondary calibration point of 24.56 Ma for the stem age of monophyletic *Rhabdophis* as estimated in a broad study of Natricidae [20].

(6) *Pareas* (Pareidae, Serpentes, Reptilia)

Two nuclear (*C-mos*, *RAG1*) and two mitochondrial genes (*Cytb*, *ND4*) were used to construct a dated phylogeny for *Pareas.* Following the study of Poyarkov et al. [21], we designated *Aplopeltura boa* (Pareidae) as the outgroup and used secondary calibration points of 33.62 Ma (95% HPD: 26.33–40.36) for the stem age of *Pareas* and 31.25 Ma (95% HPD: 24.65–37.92) for the crown age.

(7) *Gloydius* (Viperidae, Serpentes, Reptilia)

Four mitochondrial genes [12S ribosomal RNA (12S rRNA), 16S rRNA, *Cytb*, *ND4*] were used to construct a dated phylogeny for *Gloydius*. We designated "*Ovophis*" *okinavensis* (Viperidae) and "*Trimeresurus*" *gracilis* (Viperidae) as outgroups and used a secondary calibration point of 23.76 Ma for the stem age of *Gloydius* as estimated in a broad study of Viperidae [22].

(8) *Diploderma* (Agamidae, Lacertilia, Reptilia)

Three nuclear [*C-mos*, brain-derived neurotrophic factor (*BDNF*), 35 G protein-coupled receptor 149 (*R35*)] and one mitochondrial gene (*ND2*) were used to construct a dated phylogeny for *Diploderma.* We designated *Pseudocalotes kakhiensis* (Agamidae) and *Pseudocalotes kingdonwardi* (Agamidae) as outgroups and used a secondary calibration point of 33.49 Ma for the stem age of *Diploderma* as estimated in the broad study of Tonini et al. [4].

(9) *Scincella* (Scincidae, Lacertilia, Reptilia)

Six mitochondrial genes [12S rRNA, 16S rRNA, *COI*, *Cytb*, NADH dehydrogenase subunit 1 (*ND1*), *ND2*] were used to construct a dated phylogeny for *Scincella.* We designated *Sphenomorphus cryptotis* (Scincidae) as the outgroup and used a secondary calibration point of 38.29 Ma for the stem age of *Scincella* as estimated in the broad study of Tonini et al. [4].

(10) *Ochotona* (Ochotonidae, Lagomorpha, Mammalia)

Five nuclear [*RAG1*, recombination activating 2 (*RAG2*), oxidase assembly 1-like (*OXA1L*), titin (*TTN*), and interleukin 1 receptor accessory protein-like 1 (*IL1RAPL1*)] and two mitochondrial genes (*COI, Cytb*) were used to construct a dated phylogeny for *Ochotona*. Following the study of Koju et al. [23], we designated *Oryctolagus cuniculus*

(Leporidae) as the outgroup and used a secondary calibration point of 52.61 Ma (95% HPD: 47.58-58.03) for the stem age of *Ochotona.*

(11) Erinaceidae (Eulipotyphla, Mammalia)

Five nuclear [*RAG1*, growth hormone receptor (*GHR*), breast cancer protein 1 (*BRCA1*), von Willebrand factor (*vWF*), transthyretin (*TTR*)] and two mitochondrial genes (12S rRNA*, Cytb*) were used to construct a dated phylogeny for Erinaceidae. Following the study of Bannikova et al. [24], we designated *Galemys pyrenaicus* (Talpidae) as the outgroup and used secondary calibration points of 53.55 Ma (95% HPD: 49.4–56.0) for the crown age of Erinaceidae, 36.90 Ma (95% HPD: 32.0–41.6) for the crown age of Galericinae and 6.70 Ma (95% HPD: 5.5–8.2) for the crown age of Erinaceinae.

(12) Spalacidae (Rodentia, Mammalia)

Five mitochondrial genes [12S rRNA, 16S rRNA, *COI, Cytb, ND4*] were used to construct a dated phylogeny for *Scincella*. Following the study of He et al. [25], we designated *Neotoma fuscipes* (Cricetidae) as the outgroup and used secondary calibration points of 31.84 Ma (95% HPD: 27.25–36.57) for the stem age of Spalacidae and 25.11 Ma (95% HPD: 21.19–28.81) for the crown age.

(13) *Niviventer* (Muridae, Rodentia, Mammalia)

One nuclear [interphotoreceptor retinoid-binding protein (*IRBP*)] and three mitochondrial genes (*COI, Cytb,* D-loop) were used to construct a dated phylogeny for *Niviventer*. Following the study of Lu et al. [26], we designated *Chiromyscus langbianis* (Muridae) as the outgroup and used a secondary calibration point of 6.41 Ma (95% HPD: 5.09–7.78) for the stem age of *Niviventer.*

(14) *Rattus* (Muridae, Rodentia, Mammalia)

One nuclear (*IRBP*) and three mitochondrial genes (*COI, Cytb,* D-loop) were used to construct a dated phylogeny for *Rattus*. Following the study of Robins et al. [27], we designated *Mus musculus* (Muridae) as the outgroup and used a secondary calibration point of 11.62 Ma (95% HPD: 10.94–12.27) for the stem age of *Rattus.*

(15) Arvicolinae (Circetidae, Rodentia, Mammalia)

Twelve mitochondrial genes [ATP synthase F0 subunit 6 (*ATP6*), ATP synthase F0 subunit 8 (*ATP8*), *Cytb*, *ND1*, *ND2*, NADH dehydrogenase subunit 3 (*ND3*), *ND4*, NADH dehydrogenase subunit 5 (*ND5*), NADH dehydrogenase subunit 6 (*ND6*), *COI*, cytochrome c oxidase subunit II (*COII*), and cytochrome c oxidase subunit III (*COIII*)] were used to construct a dated phylogeny for Arvicolinae. Following the study of Wang et al. [28], we designated *Cricetulus kamensis* (Cricetinae) and *Cricetulus longicaudatus* (Cricetinae) as outgroups and used a secondary calibration point of 14.9 Ma for the crown age of Arvicolinae.

(16) Sciuridae (Rodentia, Mammalia)

Fourteen mitochondrial genes (12S rRNA, 16S rRNA, *ATP6, ATP8*, *Cytb*, *ND1*, *ND2*, *ND3*, *ND4*, *ND5*, *ND6*, COI, *COII*, *COIII*) were used to construct a dated phylogeny for Sciuridae. Following the study of Mercer and Roth [29], we designated *Aplodontia rufa* (Aplodontiidae) as the outgroup and used secondary calibration points of 50 Ma for the stem age of Sciuridae and 36 Ma for the crown age.

(17) Talpidae (Eulipotyphla, Mammalia)

Nineteen nuclear [adenosine A3 receptor (*ADORA3*), adrenergic receptor beta-2 (*ADRB2*), apolipoprotein B (*APOB*), amyloid beta precursor (*APP*), ATPase Cu^{++} transporting alpha polypeptide (*ATP7A*), butyrylcholinesterase (*BCHE*), *BDNF*, BMI1

proto-oncogene polycomb ring finger (*BMI1*), BRCA1, breast cancer protein 2 (*BRCA2*), cAMP responsive element moderator (*CREM*), dentin matrix acidic phosphoprotein 1 (*DMP1*), enamelin (*ENAM*), GHR, phospholipase C beta 1 (*PLCB*), *RAG1*, *RAG2*, *TTN*, *vWF*] and two mitochondrial genes (12S rRNA, *Cytb*) were used to construct a dated phylogeny for Talpidae. Following the study of He et al. [30], we designated *Crocidura dsinezumi* (Soricidae) and *Sorex unguiculatus* (Soricidae) as outgroups and used a secondary calibration point of 47 Ma (95% HPD: 42–57) for the crown age of the Talpidae.

(18) Soricinae (Soricidae, Eulipotyphla, Mammalia)

Three nuclear (*RAG2*, *APOB*, *BRCA1*) and two mitochondrial genes (*COI, Cytb*) were used to construct a dated phylogeny for Soricinae. Following the study of Springer et al. [31], we designated *Crocidura attenuata* (Crocidurinae) as the outgroup and used a secondary calibration point of 36.31 Ma (95% HPD: 28.59–44.02) for the stem age of Soricinae.

(19) Cervidae (Artiodactyla, Mammalia)

Fourteen mitochondrial genes (12S rRNA, 16S rRNA, *ATP6, ATP8*, *Cytb*, *ND1*, *ND2*, *ND3*, *ND4*, *ND5*, *ND6*, COI, *COII*, *COIII*) were used to construct a dated phylogeny for Cervidae. Following the study of Gilbert et al. [32] we designated *Boselaphus tragocamelus* (Bovidae) as the outgroup and used a secondary calibration point of 8.5 Ma (95% HPD: 7.7–9.6) for the crown age of Cervidae.

(20) Viverridae (Carnivora, Mammalia)

Two nuclear [transthyretin intron 1 (*TriI)*, *IRBP*] and one mitochondrial gene (*Cytb*) were used to construct a dated phylogeny for Viverridae. Following the study of Gaubert and Cordeiro-Estrela [33], we designated *Herpestes javanicus* (Herpestidae) and *Herpestes edwardsii* (Herpestidae) as outgroups and used secondary calibration points of 44.81 Ma for the stem age of Viverridae and 34.29 Ma for the crown age.

(21) Leiothrichidae (Passeriformes, Aves)

Seven nuclear [fibrinogen beta chain intron 5 (*FIB5*), muscle skeletal receptor tyrosine kinase intro 3 (*MUSK3*), transforming growth factor beta 2 intron 5 (*TGFB25*), ornithine decarboxylase intron 6 and 7 (*ODC6/7*), myoglobin intron 2 (*MB2*), Glyceraldehyde-3-phosphodehydrogenase intron 11 (*GAPDH11*), *RAG1*] and four mitochondrial genes (*Cytb, ND2, ND3, COI*) were used to construct a dated phylogeny for Leiothrichidae. We designated *Alcippe poioicephala* (Alcippeidae) as the outgroup and used a secondary calibration point of 17.77 Ma (95% HPD: 15.06–21.41) for the stem age of Leiothrichidae as estimated in a broad study of babblers [34].

(22) Paradoxornithidae (Passeriformes, Aves)

Seven nuclear (*FIB5*, *MUSK3*, *TGFB25*, *ODC6/7*, *MB2*, *GAPDH11*, *RAG1*) and four mitochondrial genes (*Cytb, ND2, ND3, COI*) were used to construct a dated phylogeny for Paradoxornithidae. We designated *Curruca nana* (Sylviidae) as the outgroup and used a secondary calibration point of 19.05 Ma (95% HPD: 16.12–23.08) for the stem age of Paradoxornithidae as estimated in a broad study of babblers [34].

(23) Passeridae (Passeriformes, Aves)

Two nuclear (*MB2*, *ODC6/7*) and two mitochondrial genes (*Cytb, ND2*) were used to construct a dated phylogeny for Passeridae. Following the study of Päckert at al. [35], we designated *Ploceus manyar* (Ploceidae) as the outgroup and used a secondary calibration point of 17.5 Ma for the crown age of Passeridae.

(24) Sittidae (Passeriformes, Aves)
One nuclear (*RAG1*) and two mitochondrial genes (*Cytb, COI*) were used to construct a dated phylogeny for Sittidae. Following the study of Pasquet at al. [36], we designated *Certhia familiaris* (Certhiidae) as the outgroup and used secondary calibration points of 33.89 Ma for the stem age of Sittidae and 21.09 Ma for the crown age.
(25) Corvidae (Passeriformes, Aves)
Four nuclear (*MB2*, *GAPDH11*, *ODC6/7*, *TGFB25*) and four mitochondrial genes (*Cytb, COI, ND2, ND3*) were used to construct a dated phylogeny for Corvidae. We designated *Lanius excubitor* (Laniidae) as the outgroup and used secondary calibration points of 22.62 Ma for the stem age of Corvidae and 20.47 Ma for the crown age as estimated in a broad study of Corvides [37].
(26) Fringillidae (Passeriformes, Aves)
Three nuclear (*MB2*, *GAPDH11*, *ODC6/7*) and three mitochondrial genes (*Cytb, ND2, ND3*) were used to construct a dated phylogeny for Fringillidae. We designated *Sturnella superciliaris* (Icteridae) as the outgroup and used a secondary calibration point of 24.51 Ma for the stem age of Fringillidae as estimated in a broad study of Jetz et al. [6].
(27) Vireonidae (Passeriformes, Aves)
Three mitochondrial genes (*Cytb, COI, ND2*) were used to construct a dated phylogeny for Vireonidae. We designated *Oreocharis arfaki* (Paramythiidae) as the outgroup and used secondary calibration points of 25.88 Ma for the stem age of Vireonidae and 23.66 Ma for the crown age as estimated in a broad study of Corvides [37].
(28) Timaliidae (Passeriformes, Aves)
Seven nuclear (*FIB5*, *MUSK3*, *TGFB25*, *ODC6/7*, *MB2*, *GAPDH11*, *RAG1*) and four mitochondrial genes (*Cytb, ND2, ND3, COI*) were used to construct a dated phylogeny for Timaliidae. We designated *Pellorneum ruficeps* (Pellorneidae) as the outgroup and used a secondary calibration point of 19.21 Ma (95% HPD: 16.34–23.13) for the stem age of Timaliidae as estimated in a broad study of babblers [34].
(29) Pellorneidae (Passeriformes, Aves)
Seven nuclear (*FIB5*, *MUSK3*, *TGFB25*, *ODC6/7*, *MB2*, *GAPDH11*, *RAG1*) and four mitochondrial genes (*Cytb, ND2, ND3, COI*) were used to construct a dated phylogeny for Pellorneidae. We designated *Alcippe poioicephala* (Alcippeidae) as the outgroup and used a secondary calibration point of 18.46 Ma (95% HPD: 15.69–22.23) for the stem age of Pellorneidae as estimated in a broad study of babblers [34].
(30) Alcippeidae (Passeriformes, Aves)
Seven nuclear (*FIB5*, *MUSK3*, *TGFB25*, *ODC6/7*, *MB2*, *GAPDH11*, *RAG1*) and four mitochondrial genes (*Cytb, ND2, ND3, COI*) were used to construct a dated phylogeny for Alcippeidae. We designated *Trochalopteron_affine* (Leiothrichidae) as outgroup and used a secondary calibration point of 17.77 Ma (95% HPD: 15.06–21.41 for the stem age of Alcippeidae as estimated in a broad study of babblers [34].
(31) Cettiidae (Passeriformes, Aves)
Three nuclear (*MB2*, *GAPDH11*, *ODC6/7*) and one mitochondrial gene (*Cytb*) were used to construct a dated phylogeny for Cettiidae. We designated *Phylloscopus fuscatus* (Phylloscopidae) as the outgroup and used a secondary calibration point of 18.06 Ma for the stem age of Cettiidae as estimated in a broad study of Jetz et al. [6].
(32) Asian Pycnonotidae clade (Passeriformes, Aves)

Four nuclear [fibrinogen beta chain intron 7 (*FIB7*), *TGFB25*, *ODC6/7*, *MB2*] and four mitochondrial genes (*ATP6*, *Cytb, ND2, ND3*) were used to construct a dated phylogeny for Asian Pycnonotidae. Following the study of Fuchs et al. [38], we designated *Phyllastrephus albigularis* (African Pycnonotidae clade) and *Phyllastrephus xavieri* (African Pycnonotidae clade) as outgroups and used secondary calibration points of 18.2 Ma (95% HPD: 15.1–21.8) for the split age of Asian and African clade and 17.3 Ma (95% HPD: 14.1–20.4) for the crown age of Asian Pycnonotidae clade.

(33) Locustellidae (Passeriformes, Aves)

Four nuclear [*MB2*, *ODC6/7*, *GAPDH11*, lactate dehydrogenase (*LDH*)] and one mitochondrial gene (*Cytb*) were used to construct a dated phylogeny for Locustellidae. Following the study of Alström et al. [39], we designated *Acrocephalus orientalis* (Acrocephalidae) as the outgroup and used a secondary calibration point of 22.7 Ma (95% HPD: 16.9–29.2) for the crown age of Locustellidae.

(34) Prunellidae (Passeriformes, Aves)

One nuclear [aconitase intron 9 (*ACO1I9*)] and one mitochondrial gene (*ND2*) were used to construct a dated phylogeny for Prunellidae. Following the study of Drovetski et al. [40], we designated *Quelea quelea* (Ploceidae) as the outgroup and used secondary calibration points of 14.8 Ma (95% HPD: 11.21–18.73) for the stem age of Prunellidae and 7.31 Ma (95% HPD: 5.5–9.32) for the crown age.

Delimitation of biogeographic regions

Seven biogeographic regions were delimited (Fig. S128). Following Liu, et al. [1], the HDM region was bounded to the west by the Himalayas and the Mali River in Myanmar, to the east by the Tao He, Yalong, and Bailong rivers, the Longmenshan Thrust belt, and the Xianshuihe-Xiaojiang Fault, to the south by the Gaoligong, Nu, and Yunling mountains, and to the north by the Nyainqentanglha and Tanggula, Bayan Har, and Amne Machin mountains.

We also delimited other regions. The Palearctic region was consistent with the Palearctic realm in the zoogeographic regions of the world, including Europe, the Arabian Peninsula north of the Tropic of Cancer, Africa north of the Sahara Desert, and Asia north of the Himalayas-Qinling Mountains) [41]. The Himalayan region was the large mountain system west of the HDM, mainly defined by the Indus Tsangpo Suture in the north and west, and the Main Frontal Thrust in the south, and in the east by the Jiali and Puqu faults, which corresponded well with the Yi'ong Zangbo, Palong Zangbo and Gongrigabu Qu rivers southeast of Xizang [1,42]. The South China region involved the subtropical region of China between the Qinling-Huaihe line and the Tropic of Cancer, including Taiwan. The Indo-Malay region included tropical regions of China, the Indochina Peninsula, and the Malay Archipelago. The India region occurred south of the Himalayas, including Sri Lanka. Finally, any region not falling into the aforementioned categories were designated collectively as "other regions".

We coded the species' ranges across the seven defined biogeographic regions using a presence-absence matrix, where presence is denoted by 1 and absence by 0. Data on species distributions were obtained from The IUCN Red List of Threatened Species (https://www.iucnredlist.org/), AmphibiaChina (http://www.amphibiachina.org/), Amphibian Species of the World (https://amphibiansoftheworld.amnh.org/), The Reptile Database (http://www.reptile-database.org/), ASM Mammal Diversity Database

(https://www.mammaldiversity.org/), The World Birds Database (https://avibase.bsc-eoc.org/), and Birds of the World (https://birdsoftheworld.org/).

Ancestral range reconstruction

We adapted a biogeographic model from Ding et al. [43], in which geographic ranges evolved with species birth (cladogenesis) and death. We implemented the model and simulations in RevBayes [44] based on the cladogenetic state change speciation extinction (ClaSSE) framework, and applied it to our data set of 121 time-calibrated clades of terrestrial vertebrates. In this biogeographic model, lineage “states” represent geographic ranges, comprised of one or more discrete regions. Lineages split and transition among these states through five main processes: local extinction, dispersal, sympatric speciation, subset speciation, and vicariant speciation. Each of the five processes were assigned a free parameter. The adapted model included discrete states based on seven component regions: the HDM (abbreviated H), Himalayas (B), Palearctic (C), India (D), South China (E), Indo-Malay (F), and other regions (G) (Fig. S128). There were 127 possible non-empty geographic ranges (presence in one or more regions), along with one representing global extinction (absence from all regions).

We applied stochastic character mapping to generate simulated histories consistent with the observed data from each clade by using the mnStochasticCharacterMap monitor in RevBayes. The stochastic character mapping we employed follows the method described by Freyman and Höhna (2019) [45]. It first traversed the tree post-order to compute conditional likelihoods for every arbitrarily small time interval along each branch and at nodes. States for these intervals were then sampled sequentially toward the tree tips, with each state conditioned on the previous interval’s state. At the end of each interval, forward-time probabilities were adjusted based on the backward-time conditional likelihoods, which were precomputed and stored during the initial post-order traversal. The root state was sampled from probabilities proportional to the marginal likelihood of each state at the root. This method allows for direct sampling of character histories from an SSE process in forward-time, generating a complete stochastic character map without requiring rejection sampling or uniformization. For each clade, we sampled range evolutionary histories and estimated the Bayesian posterior density from the joint distribution conditioned on the tip states and the model parameters during Markov chain Monte Carlo (MCMC). After a preliminary run to tune the proposal parameters, we performed the MCMC until all parameter posteriors achieved effective sample sizes (ESS) surpassing 200 after discarding the first 25% of posterior samples. The resulting stochastic mappings were stored as sequences of range states along the branches and at nodes in the phylogeny, which allowed distinguishing between cladogenic and anagenic changes along branches. In the output log files, each row in the file corresponds to a distinct sample from the MCMC, while each column represents the state history of a specific node within the phylogeny. The last column of the log file contains the complete stochastic character map of the entire tree in SIMMAP format. Using custom Python scripts, we parsed the changes of each MCMC sampling in the log file. To incorporate uncertainty in biogeographic estimates, we randomly resampled 1,000 stochastic maps for each clade and then aggregated different groups to infer the rolling estimates of rates of range evolution and lineage diversification through time. Scripts to run all RevBayes analyses presented here can be found in the repository at https://github.com/CheLab-KIZ/Paper-codes/tree/main/CJPA202402.

Temporal dynamics of assembly across taxonomic groups

We focused our analysis on the temporal dynamics of faunal assembly in the HDM, particularly emphasizing the comparison among different taxonomic groups. Our objective was to estimate, for each taxonomic group and entire terrestrial vertebrates, rates of *in situ* speciation, colonization, and their cumulative contributions to biotic assembly through time, while accounting for phylogenetic uncertainty.

Based on those randomly sampled 1,000 stochastic maps for each clade from the total stored sequences of range evolution, we summarized the events and combined them into different taxonomic groups, including amphibians, reptiles, mammals, birds, and terrestrial vertebrates. Thus, from each resampled stochastic mapping, we extracted the time and transitional events along the branch of phylogeny and calculated *in situ* speciation, colonization, and local extinction events affecting the biotic assembly across different taxonomic groups.

We followed Xing and Ree [46] to calculate rolling estimates of per capita *in situ* speciation rates as

$$\lambda(t) = s(t)/n(t-1)$$

where s(t) is the number of *in situ* speciation events inferred in a region in a 1-My period t and n(t − 1) is the number of inferred lineages in the region in the previous period (the cumulative sum of *in situ* speciation and colonization minus local extinction). Likewise, we calculated rolling per capita rates of colonization between regions as

$$dij(t) = cij(t)/ni(t-1)$$

where cij(t) is the number of inferred colonization events of region j from region i [46]. For all estimates, we plotted the median value and shaded areas indicated the 25% to 75% quantile intervals through time from 1,000 replicated joint biogeographic histories.

SI REFERENCES

1. Liu J, Milne RI, Zhu G-F *et al.* Name and scale matter: clarifying the geography of Tibetan Plateau and adjacent mountain regions. *Glob Planet Change* 2022; **215**: 103893.
2. Liang J-C, Ding Z-F, Li C-L *et al.* Patterns and drivers of avian taxonomic and phylogenetic beta diversity in China vary across geographical backgrounds and dispersal abilities. *Zool Res* 2024; **45**: 125–35.
3. Jetz W, Pyron RA. The interplay of past diversification and evolutionary isolation with present imperilment across the amphibian tree of life. *Nat Ecol Evol* 2018; **2**: 850–8.
4. Tonini JFR, Beard KH, Ferreira RB *et al.* Fully-sampled phylogenies of squamates reveal evolutionary patterns in threat status. *Biol Conserv* 2016; **204**: 23–31.
5. Upham NS, Esselstyn JA, Jetz W. Inferring the mammal tree: species-level sets of phylogenies for questions in ecology, evolution, and conservation. *PLoS Biol* 2019; **17**: e3000494.
6. Jetz W, Thomas GH, Joy JB *et al.* The global diversity of birds in space and time. *Nature* 2012; **491**: 444–8.
7. Paradis E, Schliep K. ape 5.0: an environment for modern phylogenetics and evolutionary analyses in R. *Bioinformatics* 2019; **35**: 526–8.
8. Katoh K, Standley DM. MAFFT multiple sequence alignment software version 7: improvements in performance and usability. *Mol Biol Evol* 2013; **30**: 772–80.
9. Kumar S, Stecher G, Li M *et al.* MEGA X: Molecular evolutionary genetics analysis across computing platforms. *Mol Biol Evol* 2018; **35**: 1547–9.
10. Katoh K, Toh H. Recent developments in the MAFFT multiple sequence alignment program. *Brief Bioinform* 2008; **9**: 286–98.
11. Castresana J. Selection of conserved blocks from multiple alignments for their use in phylogenetic analysis. *Mol Biol Evol* 2000; **17**: 540–52.
12. Minh BQ, Schmidt HA, Chernomor O *et al.* IQ-TREE 2: new models and efficient methods for phylogenetic inference in the genomic era. *Mol Biol Evol* 2020; **37**: 1530–4.
13. Kalyaanamoorthy S, Minh BQ, Wong TKF *et al.* ModelFinder: fast model selection for accurate phylogenetic estimates. *Nat Methods* 2017; **14**: 587–9.
14. Hoang DT, Chernomor O, von Haeseler A *et al.* UFBoot2: improving the ultrafast bootstrap approximation. *Mol Biol Evol* 2018; **35**: 518–22.
15. Guindon S, Dufayard J-F, Lefort V *et al.* New algorithms and methods to estimate maximum-likelihood phylogenies: assessing the performance of PhyML 3.0. *Syst Biol* 2010; **59**: 307–21.
16. Smith SA, O'Meara BC. treePL: divergence time estimation using penalized likelihood for large phylogenies. *Bioinformatics* 2012; **28**: 2689–90.
17. Hofmann S, Stöck M, Zheng Y *et al.* Molecular phylogenies indicate a paleo-Tibetan origin of Himalayan lazy toads (*Scutiger*). *Sci Rep* 2017; **7**: 3308.
18. Wu Y-H, Yan F, Stuart BL *et al.* A combined approach of mitochondrial DNA and anchored nuclear phylogenomics sheds light on unrecognized diversity, phylogeny, and historical biogeography of the torrent frogs, genus *Amolops* (Anura: Ranidae). *Mol Phylogenet Evol* 2020; **148**: 106789.

19. Li J-N, Liang D, Wang Y-Y *et al.* A large-scale systematic framework of Chinese snakes based on a unified multilocus marker system. *Mol Phylogenet Evol* 2020; **148**: 106807.
20. Deepak V, Cooper N, Poyarkov NA *et al.* Multilocus phylogeny, natural history traits and classification of natricine snakes (Serpentes: Natricinae). *Zool J Linn Soc* 2022; **195**: 279–98.
21. Poyarkov NA, Nguyen TV, Pawangkhanant P *et al.* An integrative taxonomic revision of slug-eating snakes (Squamata: Pareidae: Pareineae) reveals unprecedented diversity in Indochina. *PeerJ* 2022; **10**: e12713.
22. Alencar LRV, Quental TB, Grazziotin FG *et al.* Diversification in vipers: phylogenetic relationships, time of divergence and shifts in speciation rates. *Mol Phylogenet Evol* 2016; **105**: 50–62.
23. Koju NP, He K, Chalise MK *et al.* Multilocus approaches reveal underestimated species diversity and inter-specific gene flow in pikas (*Ochotona*) from southwestern China. *Mol Phylogenet Evol* 2017; **107**: 239–45.
24. Bannikova AA, Lebedev VS, Abramov AV *et al.* Contrasting evolutionary history of hedgehogs and gymnures (Mammalia: Erinaceomorpha) as inferred from a multigene study. *Biol J Linn Soc* 2014; **112**: 499–519.
25. He Y, Hu S, Ge D *et al.* Evolutionary history of Spalacidae inferred from fossil occurrences and molecular phylogeny. *Mamm Rev* 2020; **50**: 11–24.
26. Lu L, Ge D, Chesters D *et al.* Molecular phylogeny and the underestimated species diversity of the endemic white-bellied rat (Rodentia: Muridae: *Niviventer*) in Southeast Asia and China. *Zool Scr* 2015; **44**: 475–94.
27. Robins JH, McLenachan PA, Phillips MJ *et al.* Dating of divergences within the *Rattus* genus phylogeny using whole mitochondrial genomes. *Mol Phylogenet Evol* 2008; **49**: 460–6.
28. Wang X, Liang D, Wang X *et al.* Phylogenomics reveals the evolution, biogeography, and diversification history of voles in the Hengduan Mountains. *Commun Biol* 2022; **5**: 1124.
29. Mercer JM, Roth VL. The effects of Cenozoic global change on squirrel phylogeny. *Science* 2003; **299**: 1568–72.
30. He K, Shinohara A, Helgen KM *et al.* Talpid mole phylogeny unites shrew moles and illuminates overlooked cryptic species diversity. *Mol Biol Evol* 2017; **34**: 78–87.
31. Springer MS, Murphy WJ, Roca AL. Appropriate fossil calibrations and tree constraints uphold the Mesozoic divergence of solenodons from other extant mammals. *Mol Phylogenet Evol* 2018; **121**: 158–65.
32. Gilbert C, Ropiquet A, Hassanin A. Mitochondrial and nuclear phylogenies of Cervidae (Mammalia, Ruminantia): systematics, morphology, and biogeography. *Mol Phylogenet Evol* 2006; **40**: 101–17.
33. Gaubert P, Cordeiro-Estrela P. Phylogenetic systematics and tempo of evolution of the Viverrinae (Mammalia, Carnivora, Viverridae) within feliformians: implications for faunal exchanges between Asia and Africa. *Mol Phylogenet Evol* 2006; **41**: 266–78.
34. Cai T, Cibois A, Alström P *et al.* Near-complete phylogeny and taxonomic revision of the world's babblers (Aves: Passeriformes). *Mol Phylogenet Evol* 2019; **130**: 346–56.

35. Päckert M, Hering J, Belkacem AA *et al.* A revised multilocus phylogeny of Old World sparrows (Aves: Passeridae). *Vertebr Zool* 2021; **71**: 353–66.
36. Pasquet E, Barker FK, Martens J *et al.* Evolution within the nuthatches (Sittidae: Aves, Passeriformes): molecular phylogeny, biogeography, and ecological perspectives. *J Ornithol* 2014; **155**: 755–65.
37. Jønsson KA, Fabre P-H, Kennedy JD *et al.* A supermatrix phylogeny of corvoid passerine birds (Aves: Corvides). *Mol Phylogenet Evol* 2016; **94**: 87–94.
38. Fuchs J, Pasquet E, Stuart BL *et al.* Phylogenetic affinities of the enigmatic bare-faced bulbul *Pycnonotus hualon* with description of a new genus. *Ibis* 2018; **160**: 659–65.
39. Alström P, Cibois A, Irestedt M *et al.* Comprehensive molecular phylogeny of the grassbirds and allies (Locustellidae) reveals extensive non-monophyly of traditional genera, and a proposal for a new classification. *Mol Phylogenet Evol* 2018; **127**: 367–75.
40. Drovetski SV, Semenov G, Drovetskaya SS *et al.* Geographic mode of speciation in a mountain specialist avian family endemic to the Palearctic. *Ecol Evol* 2013; **3**: 1518–28.
41. Wallace AR. *The Geographical Distribution of Animals*. Cambridge: Cambridge University Press, 1876.
42. Haproff PJ, Zuza AV, Yin A *et al.* Geologic framework of the northern Indo-Burma Ranges and lateral correlation of Himalayan-Tibetan lithologic units across the eastern Himalayan syntaxis. *Geosphere* 2019; **15**: 856–81.
43. Ding W-N, Ree RH, Spicer RA *et al.* Ancient orogenic and monsoon-driven assembly of the world's richest temperate alpine flora. *Science* 2020; **369**: 578–81.
44. Höhna S, Landis MJ, Heath TA *et al.* RevBayes: Bayesian phylogenetic inference using graphical models and an interactive model-specification language. *Syst Biol* 2016; **65**: 726–36.
45. Freyman WA, Höhna S. Stochastic character mapping of state-dependent diversification reveals the tempo of evolutionary decline in self-compatible Onagraceae lineages. *Syst Biol* 2019; **68**: 505–19.
46. Xing Y, Ree RH. Uplift-driven diversification in the Hengduan Mountains, a temperate biodiversity hotspot. *Proc Natl Acad Sci USA* 2017; **114**: E3444–51.

Supplementary Figures

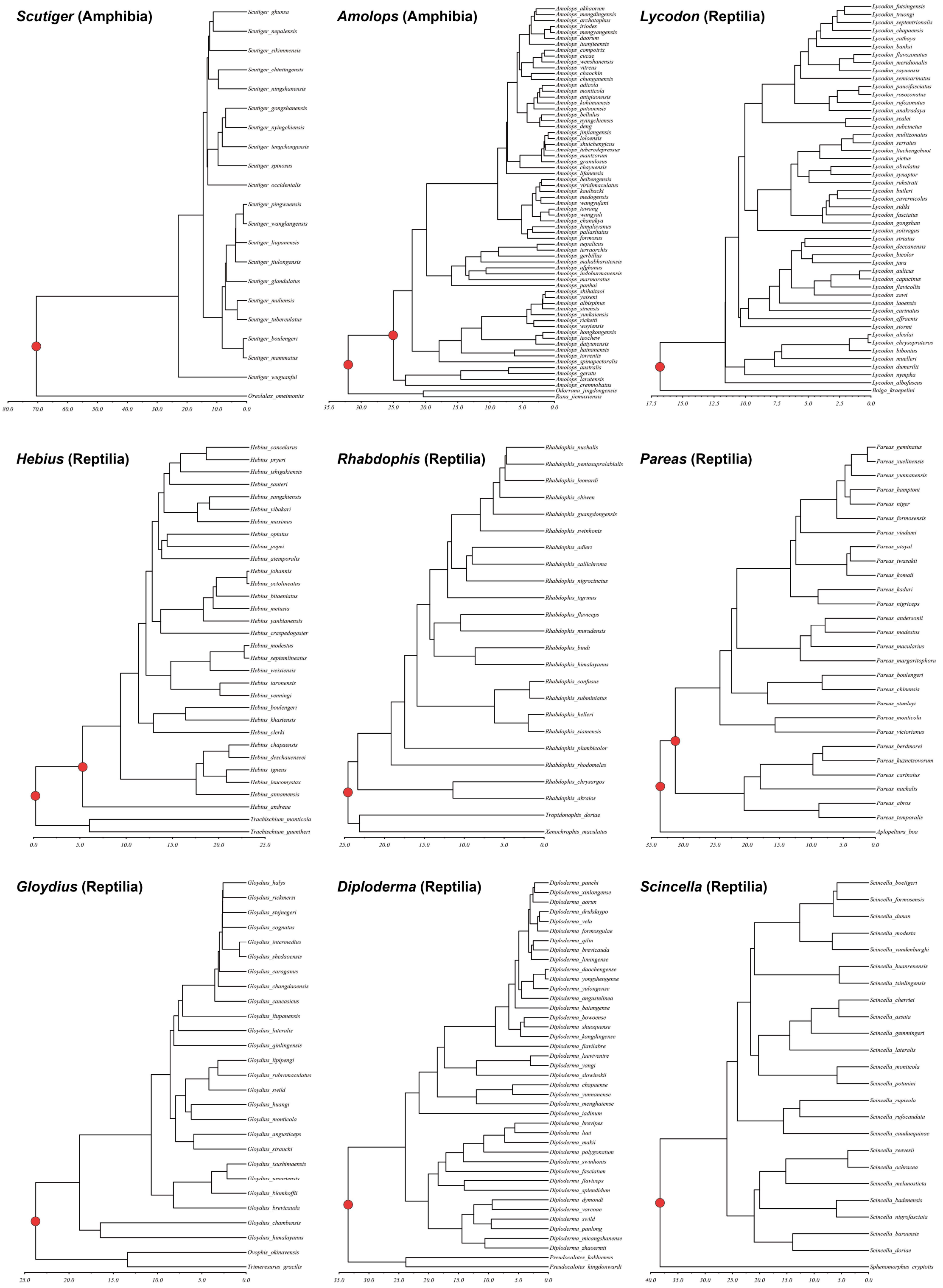

***Ochotona* (Mammalia)**

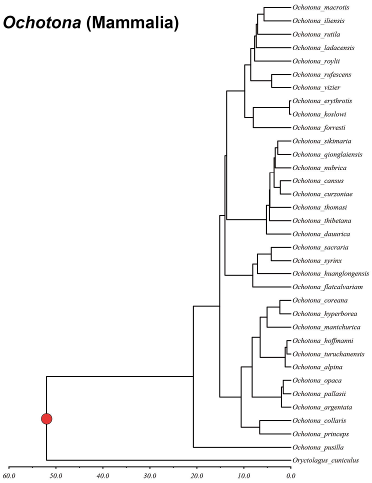

Erinaceidae (Mammalia)

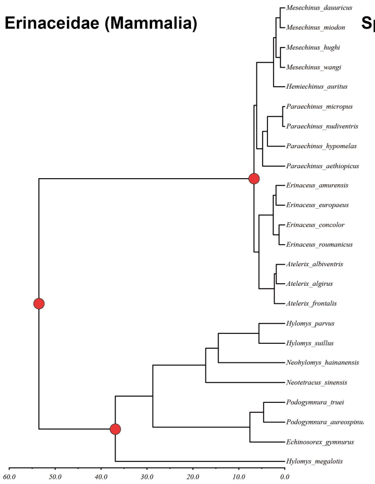

Spalacidae (Mammalia)

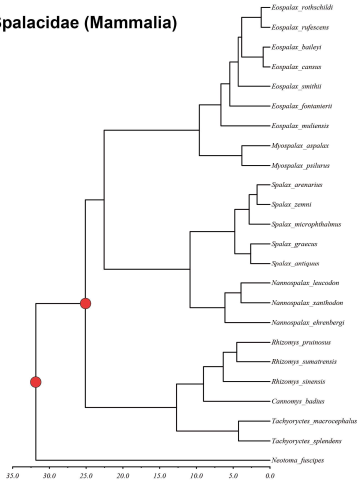

***Niviventer* (Mammalia)**

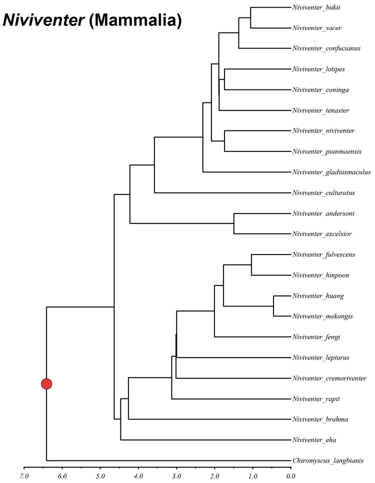

Arvicolinae (Mammalia)

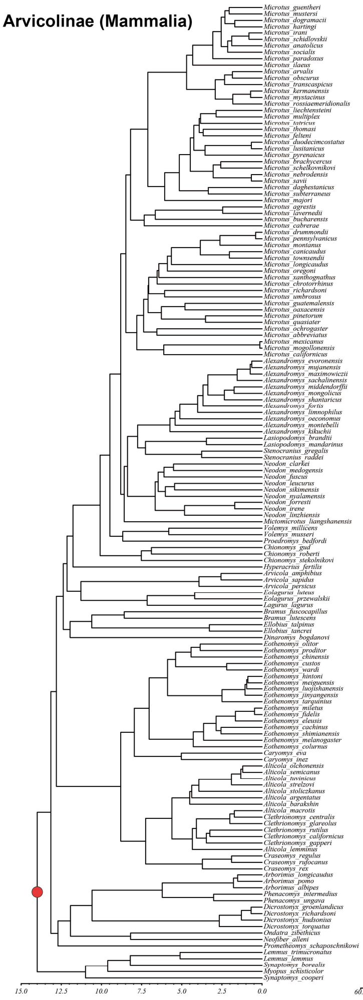

***Rattus* (Mammalia)**

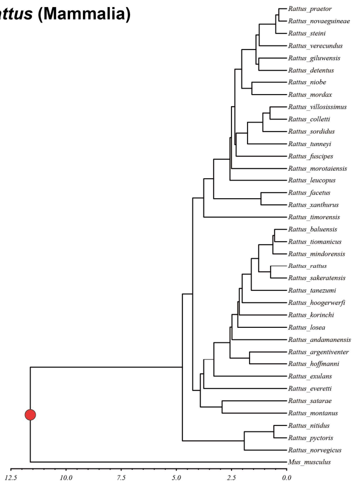

Sciuridae (Mammalia)

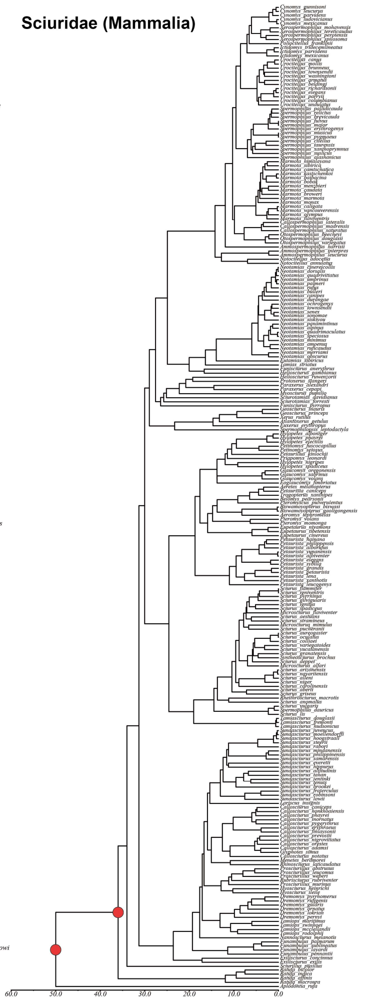

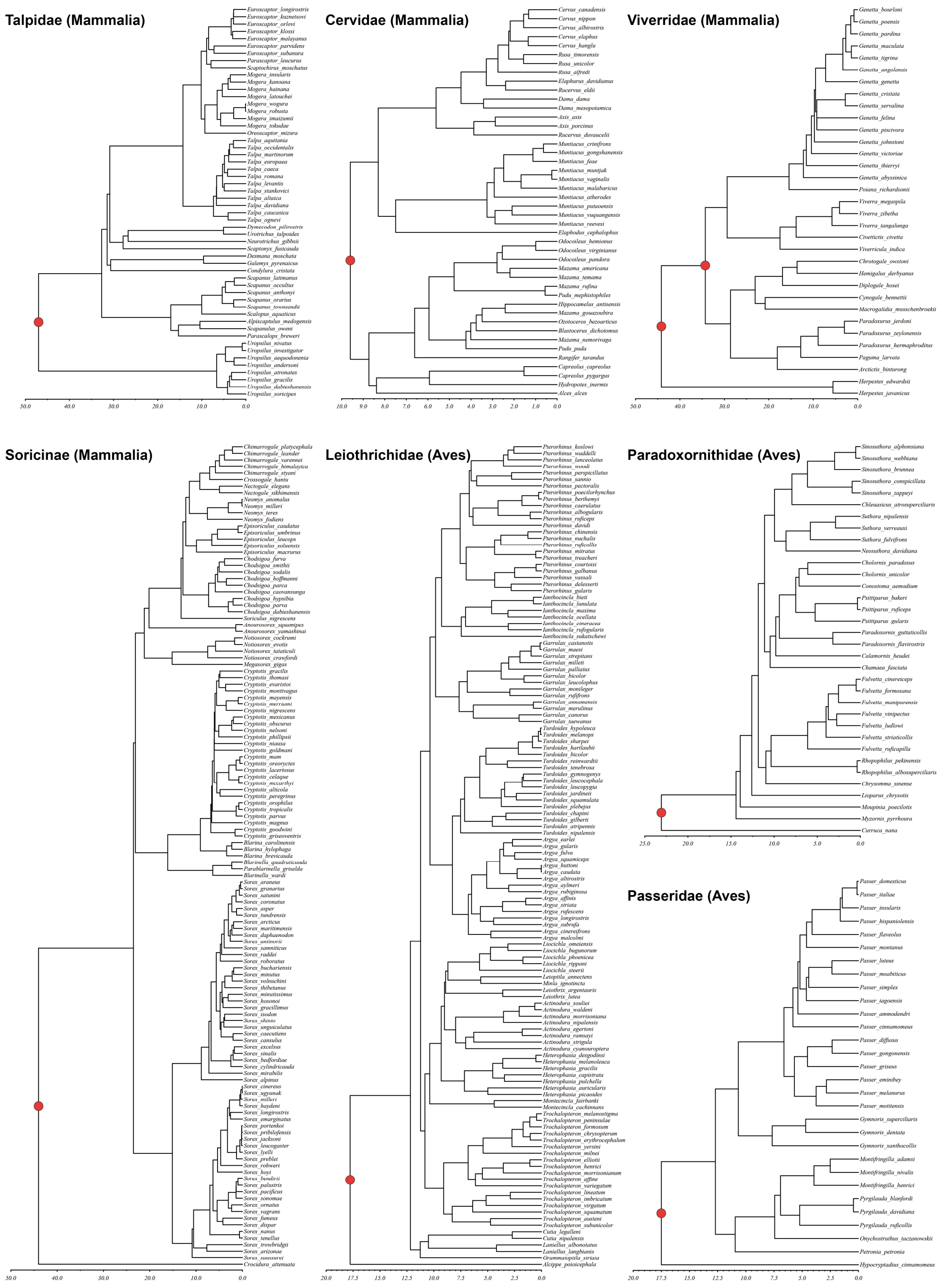
Talpidae (Mammalia)
Cervidae (Mammalia)
Viverridae (Mammalia)
Soricinae (Mammalia)
Leiothrichidae (Aves)
Paradoxornithidae (Aves)
Passeridae (Aves)

Sittidae (Aves)

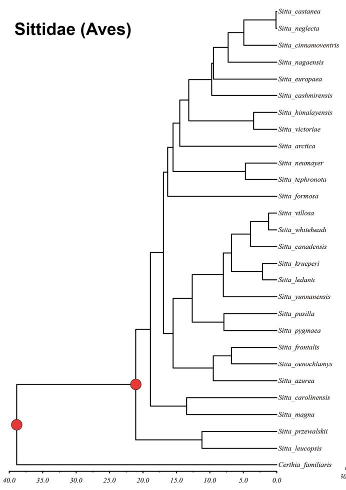

Vireonidae (Aves)

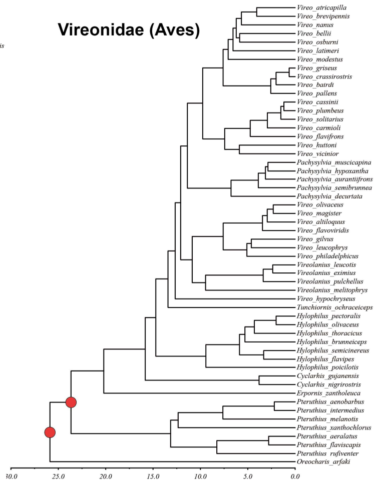

Timaliidae (Aves)

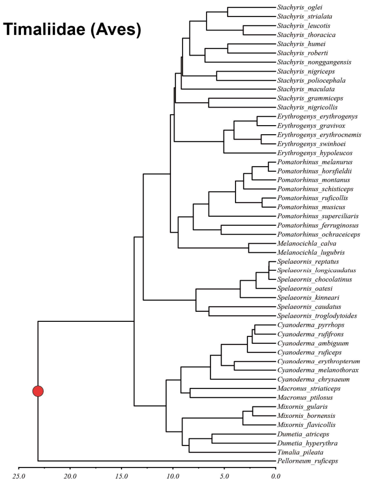

Corvidae (Aves)

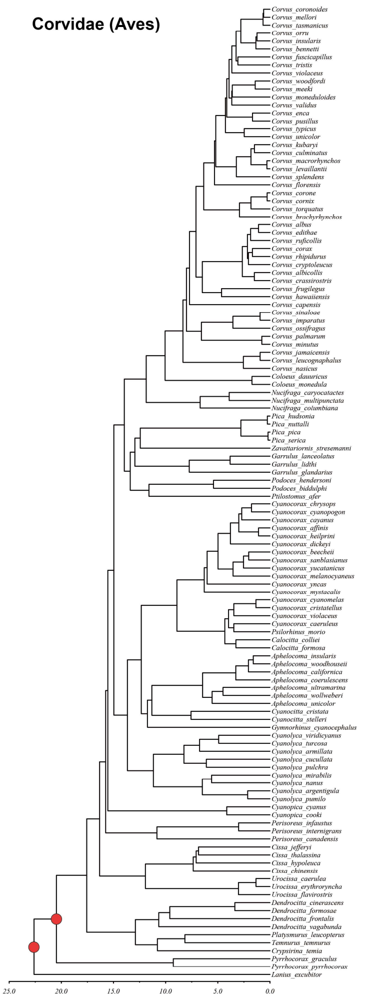

Fringillidae (Aves)

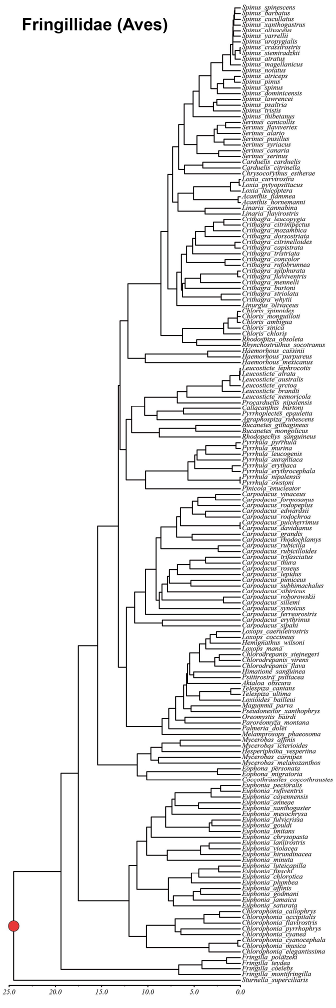

Pellorneidae (Aves)

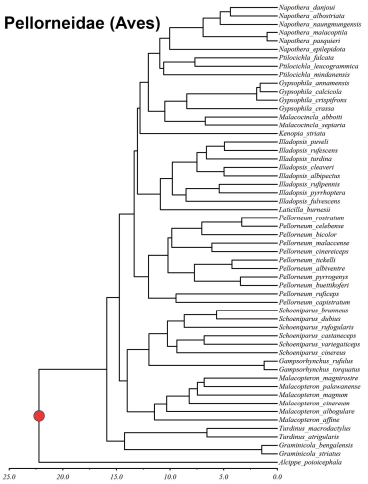

Alcippeidae (Aves)

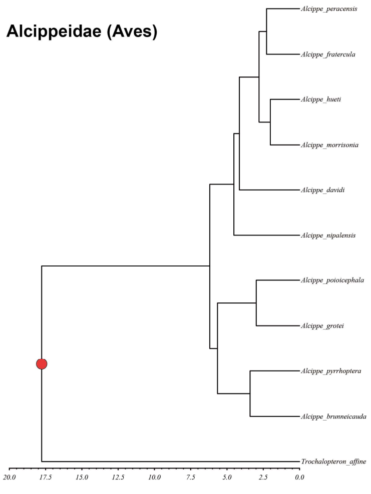

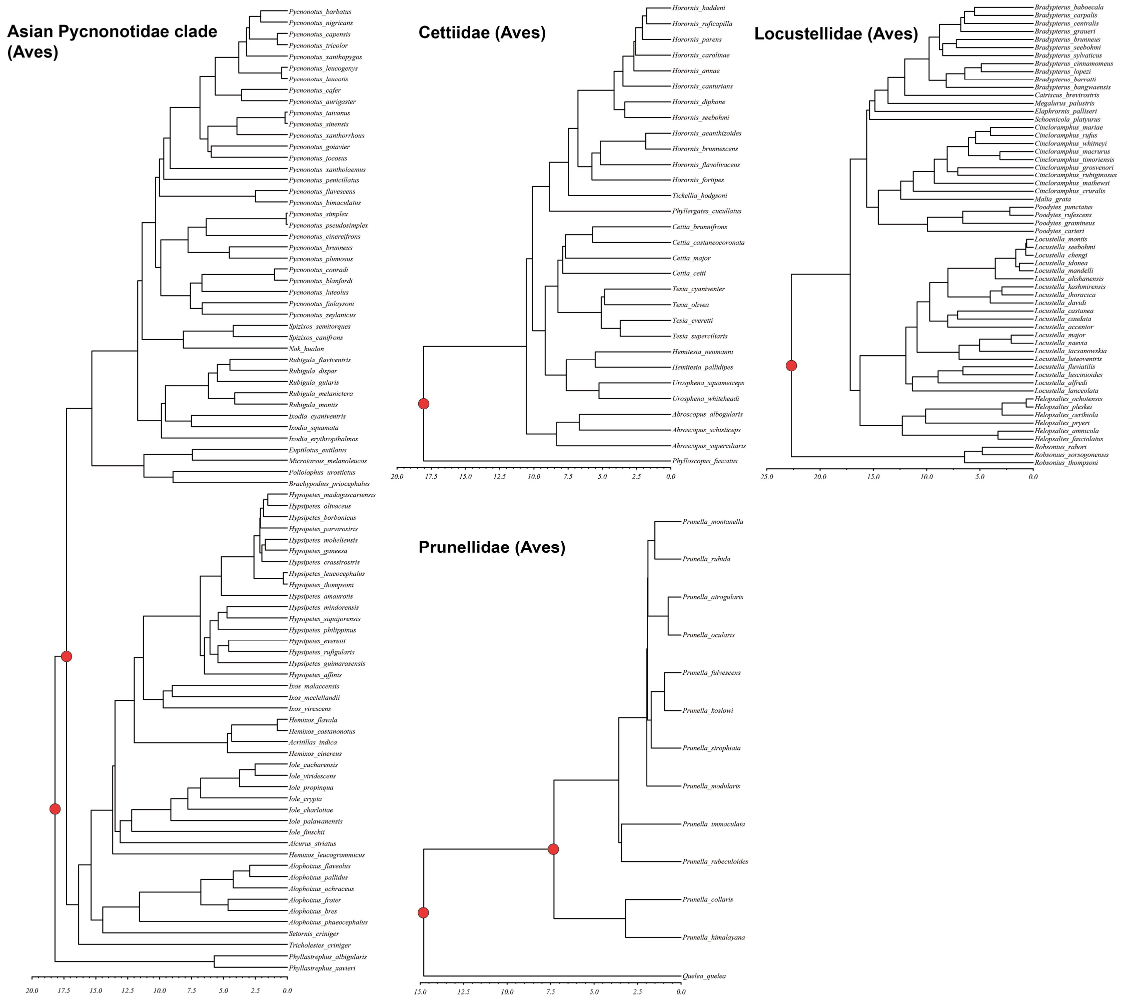

Fig. S1. Time-calibrated phylogenies for the 34 clades constructed in this study. Red circles indicate secondary calibration points used for divergence time estimation.

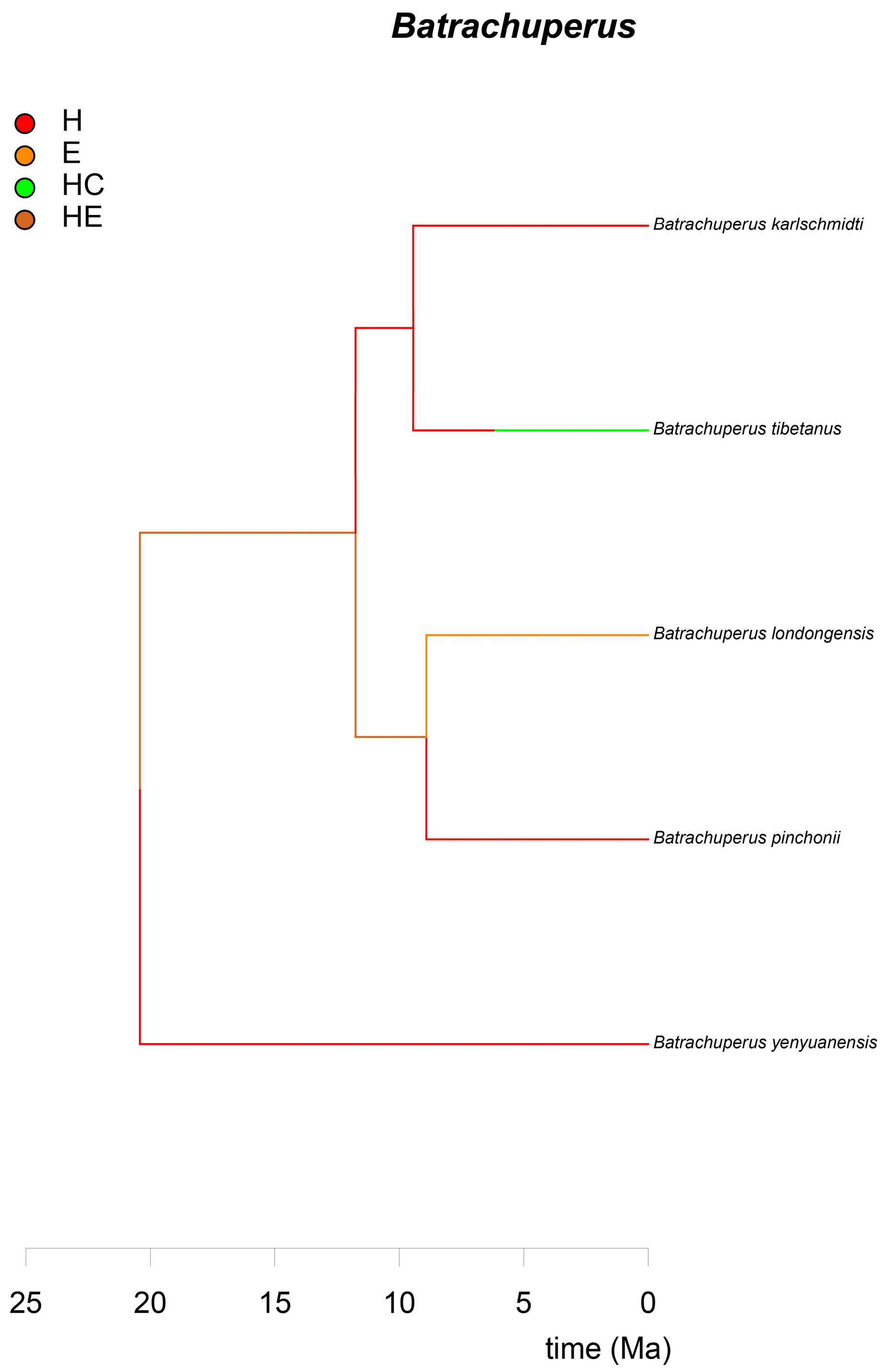

Fig. S2. Marginal maximum *a posteriori* reconstruction of the evolutionary history of geographic range on the maximum clade credibility tree of *Batrachuperus* using RevBayes. Geographic regions are coded as follows: H, Hengduan Mountains; B, Himalayas; C, Palearctic; D, India; E, South China; F, Indo-Malay; G, other regions.

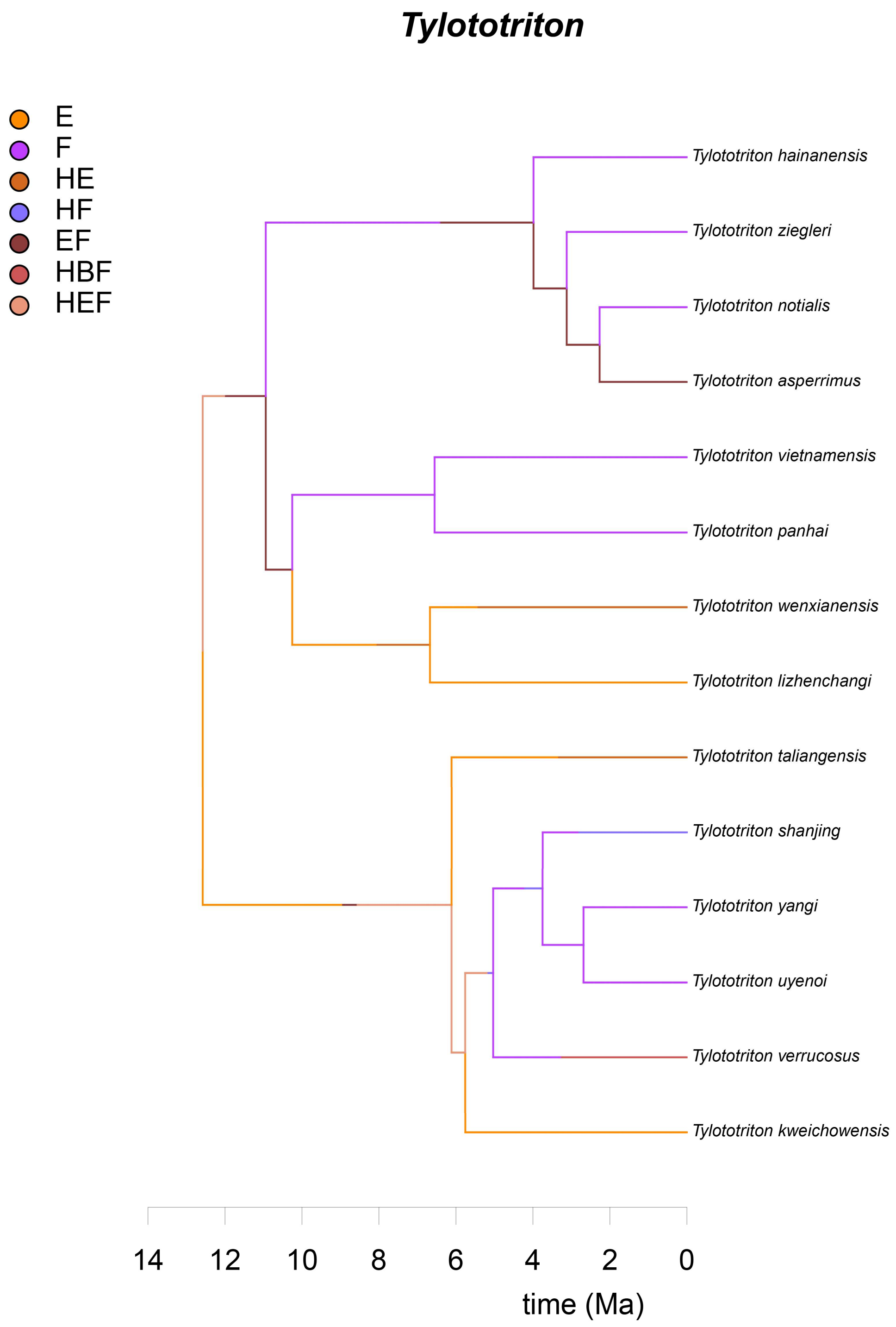

Fig. S3. Marginal maximum *a posteriori* reconstruction of the evolutionary history of geographic range on the maximum clade credibility tree of *Tylototriton* using RevBayes. Labels for geographic regions follow Fig. S2.

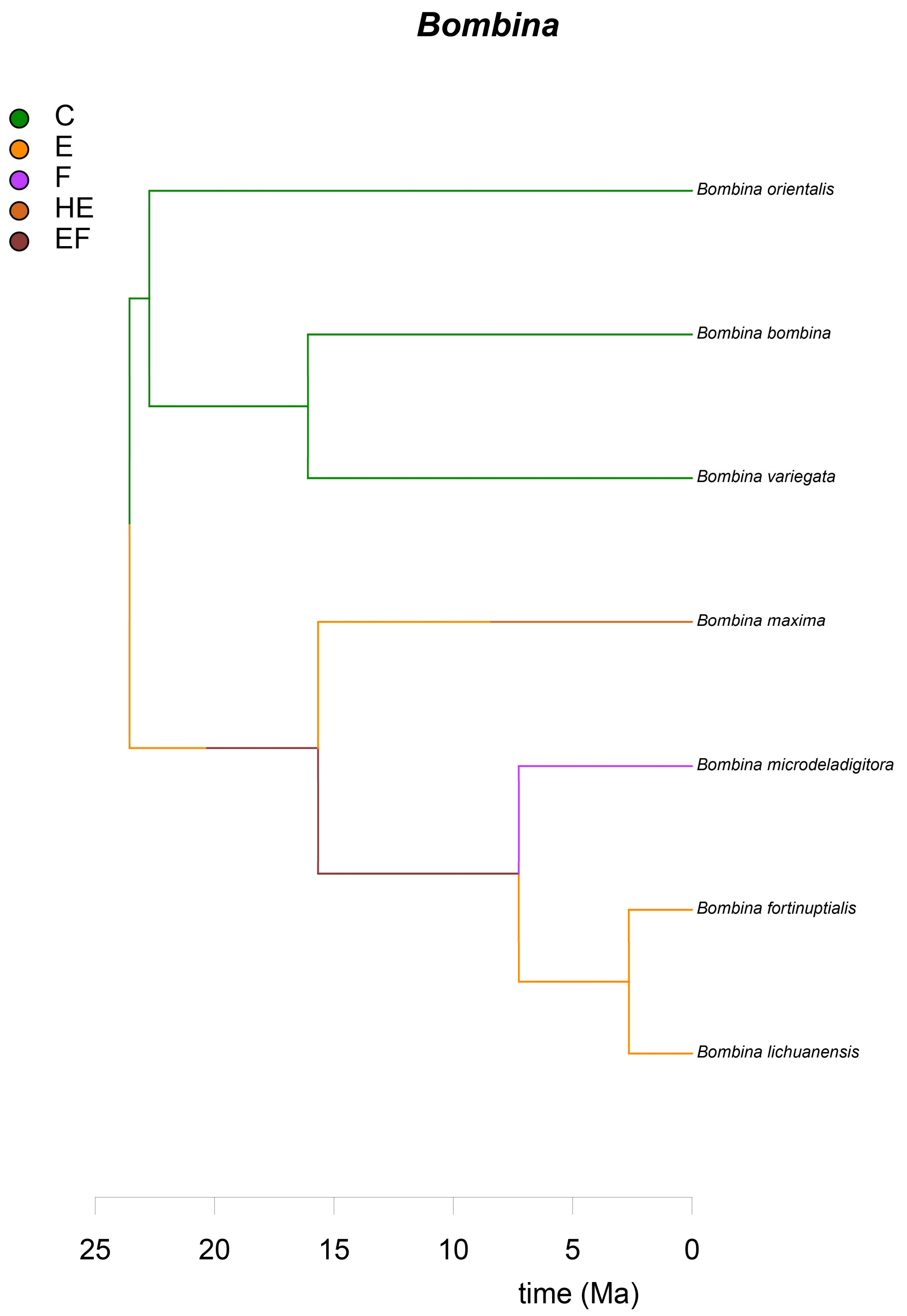

Fig. S4. Marginal maximum *a posteriori* reconstruction of the evolutionary history of geographic range on the maximum clade credibility tree of *Bombina* using RevBayes. Labels for geographic regions follow Fig. S2.

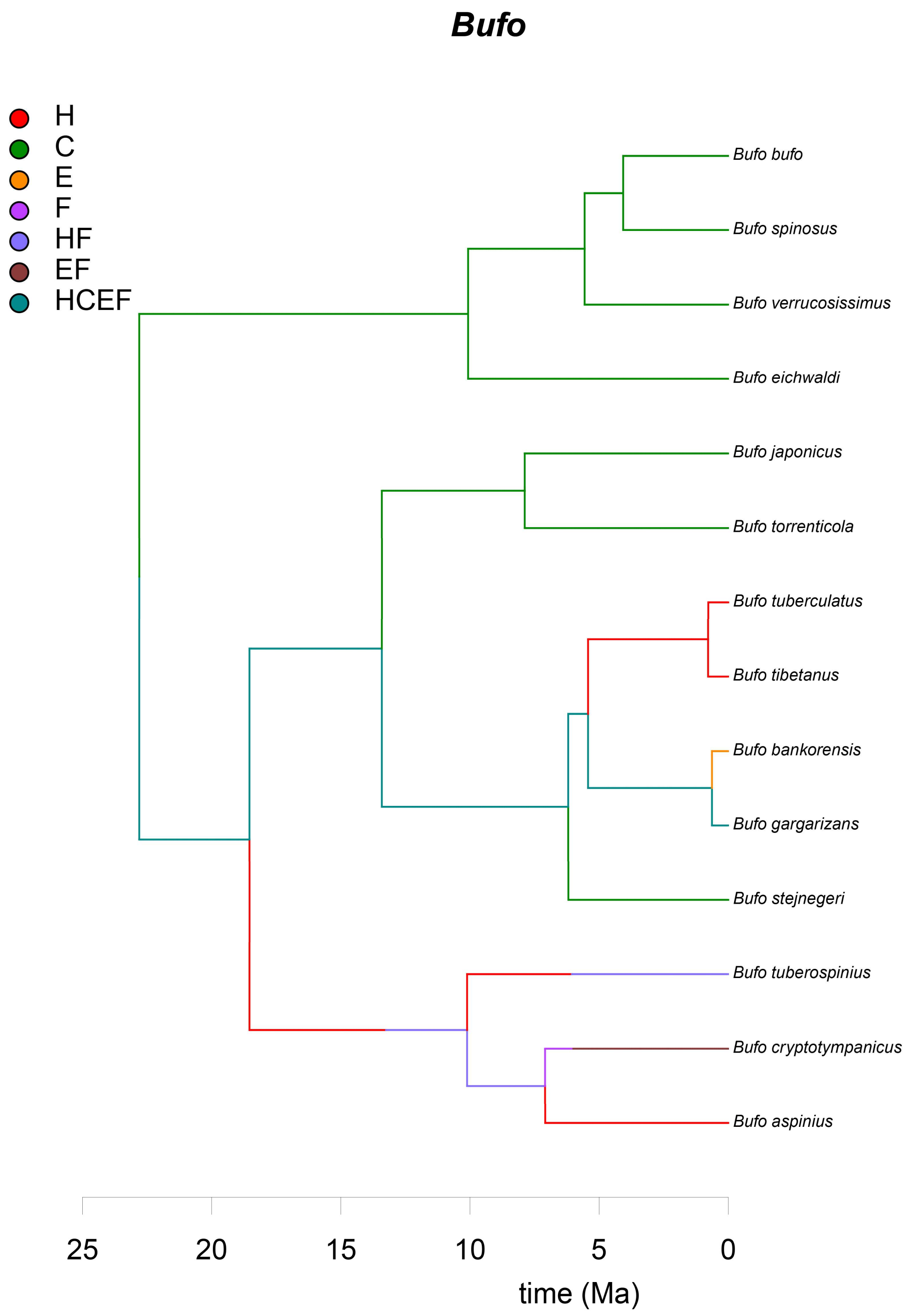

Fig. S5. Marginal maximum *a posteriori* reconstruction of the evolutionary history of geographic range on the maximum clade credibility tree of *Bufo* using RevBayes. Labels for geographic regions follow Fig. S2.

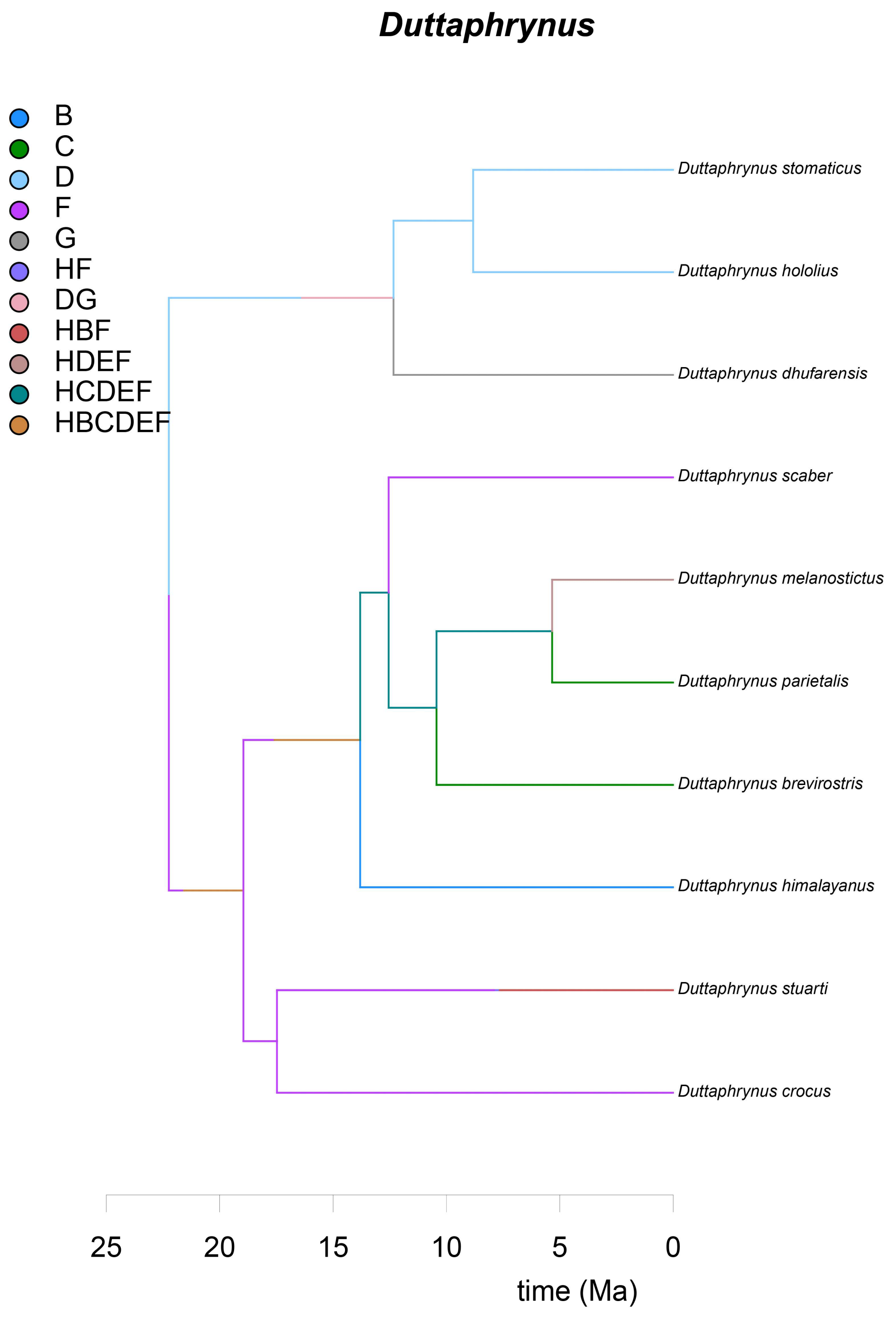

Fig. S6. Marginal maximum *a posteriori* reconstruction of the evolutionary history of geographic range on the maximum clade credibility tree of *Duttaphrynus* using RevBayes. Labels for geographic regions follow Fig. S2.

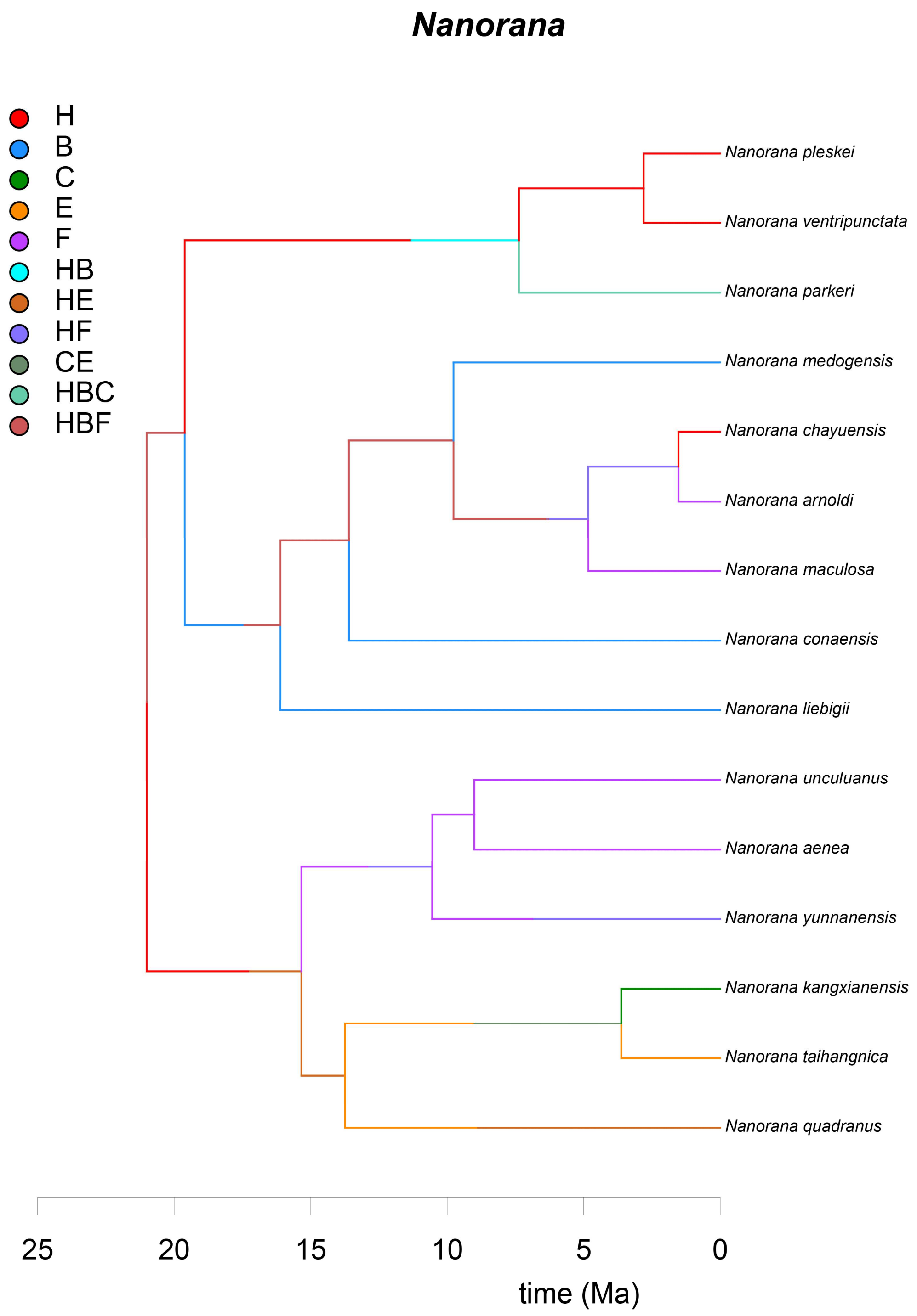

Fig. S7. Marginal maximum *a posteriori* reconstruction of the evolutionary history of geographic range on the maximum clade credibility tree of *Nanorana* using RevBayes. Labels for geographic regions follow Fig. S2.

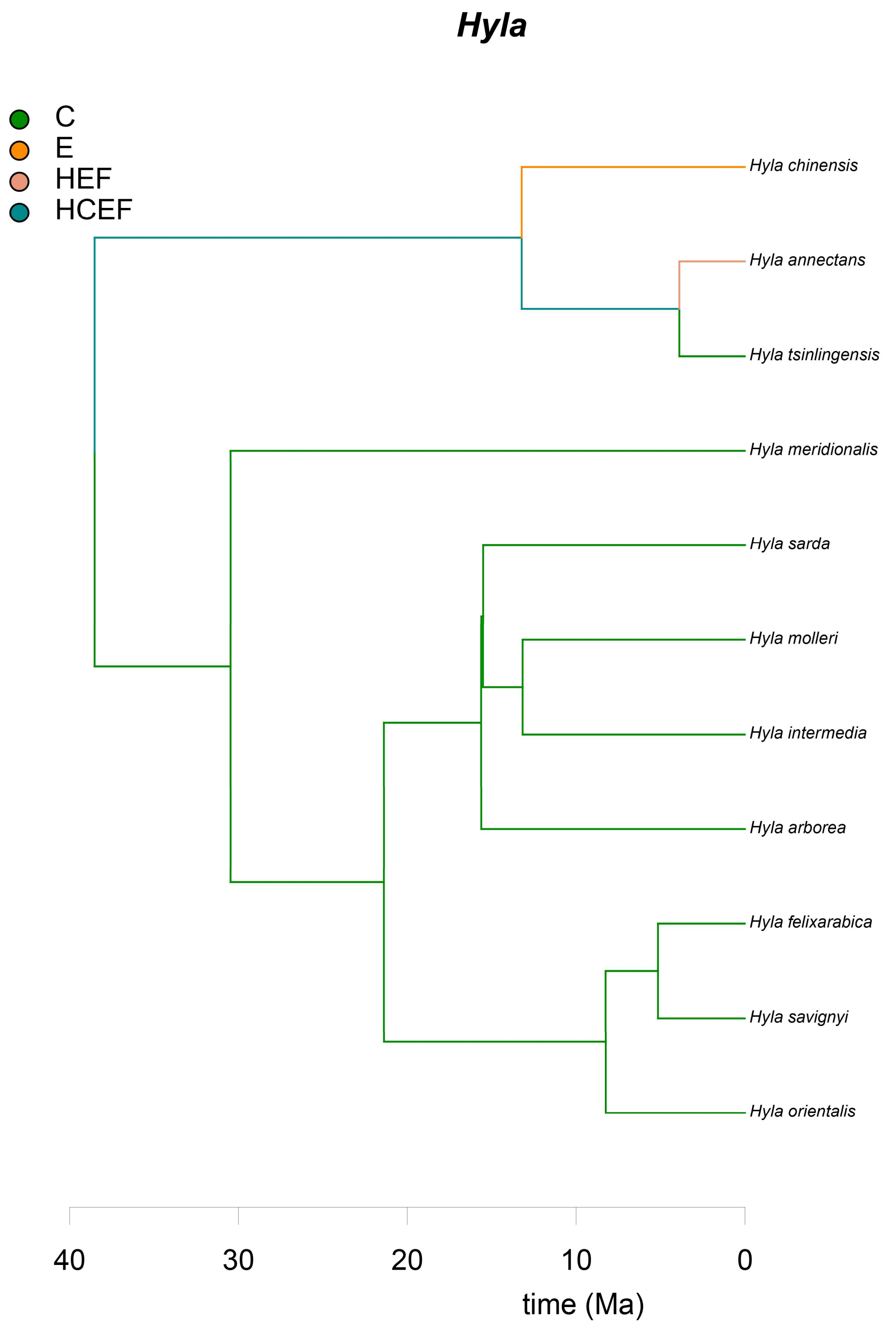

Fig. S8. Marginal maximum *a posteriori* reconstruction of the evolutionary history of geographic range on the maximum clade credibility tree of *Hyla* using RevBayes. Labels for geographic regions follow Fig. S2.

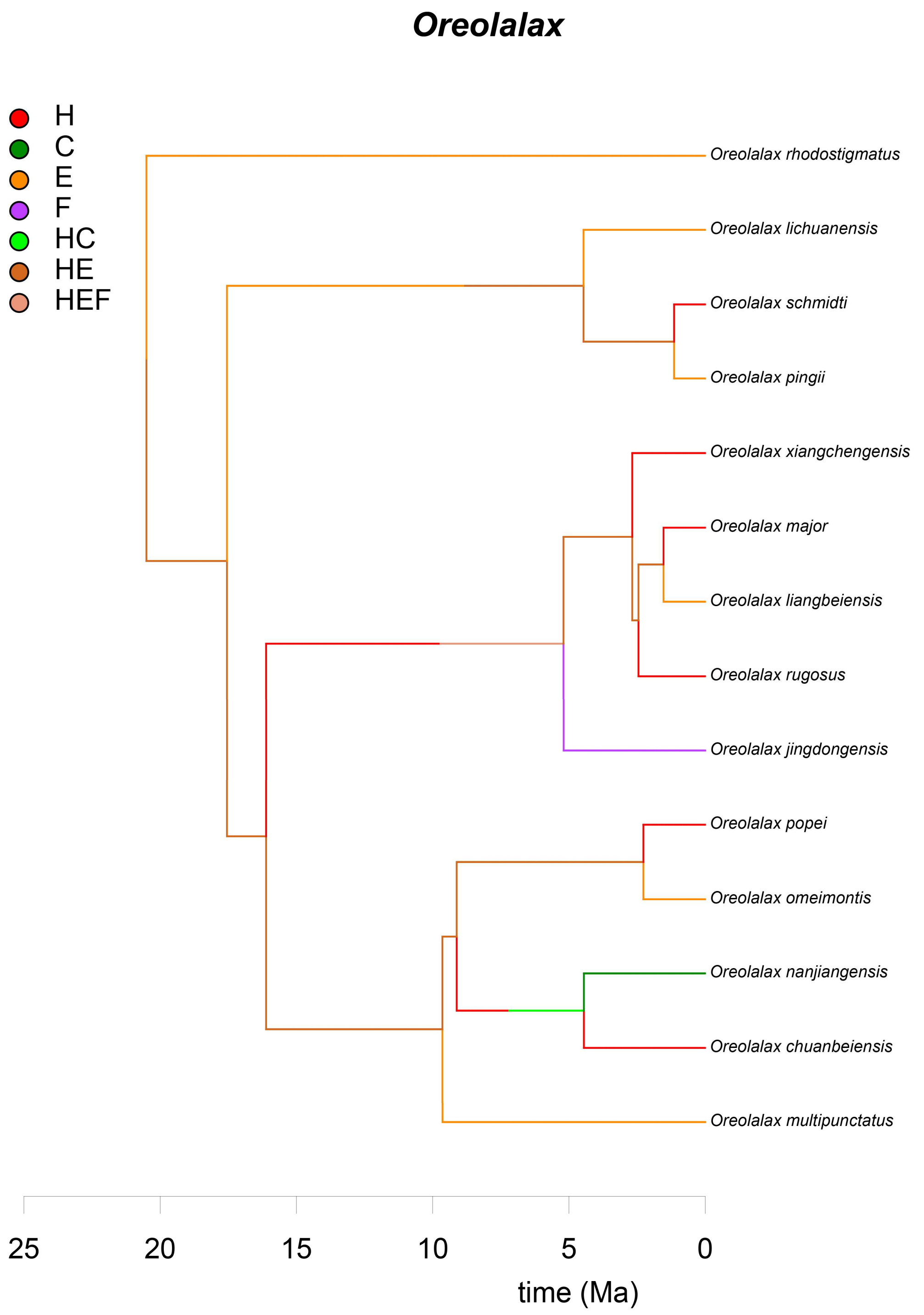

Fig. S9. Marginal maximum *a posteriori* reconstruction of the evolutionary history of geographic range on the maximum clade credibility tree of *Oreolalax* using RevBayes. Labels for geographic regions follow Fig. S2.

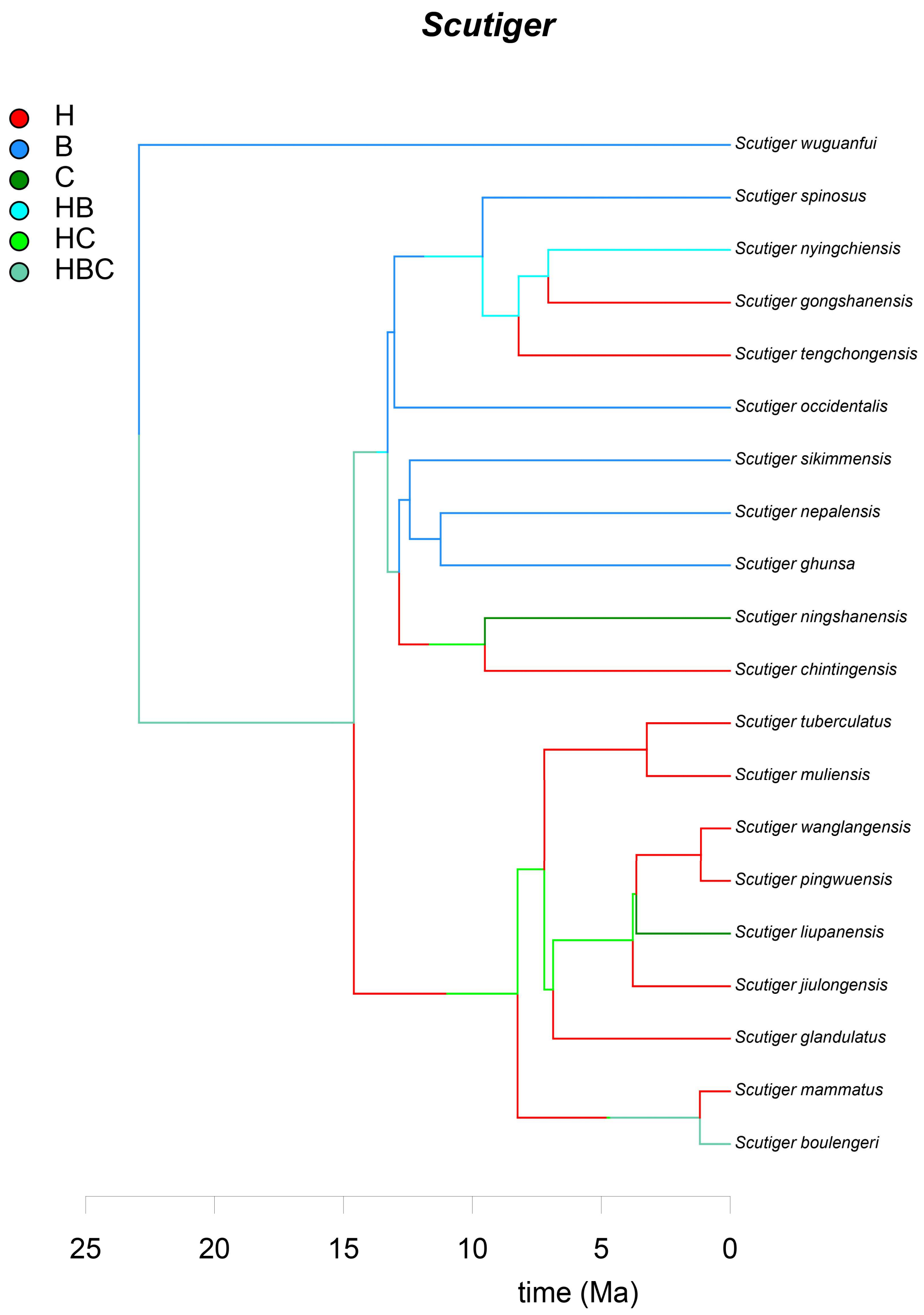

Fig. S10. Marginal maximum *a posteriori* reconstruction of the evolutionary history of geographic range on the maximum clade credibility tree of *Scutiger* using RevBayes. Labels for geographic regions follow Fig. S2.

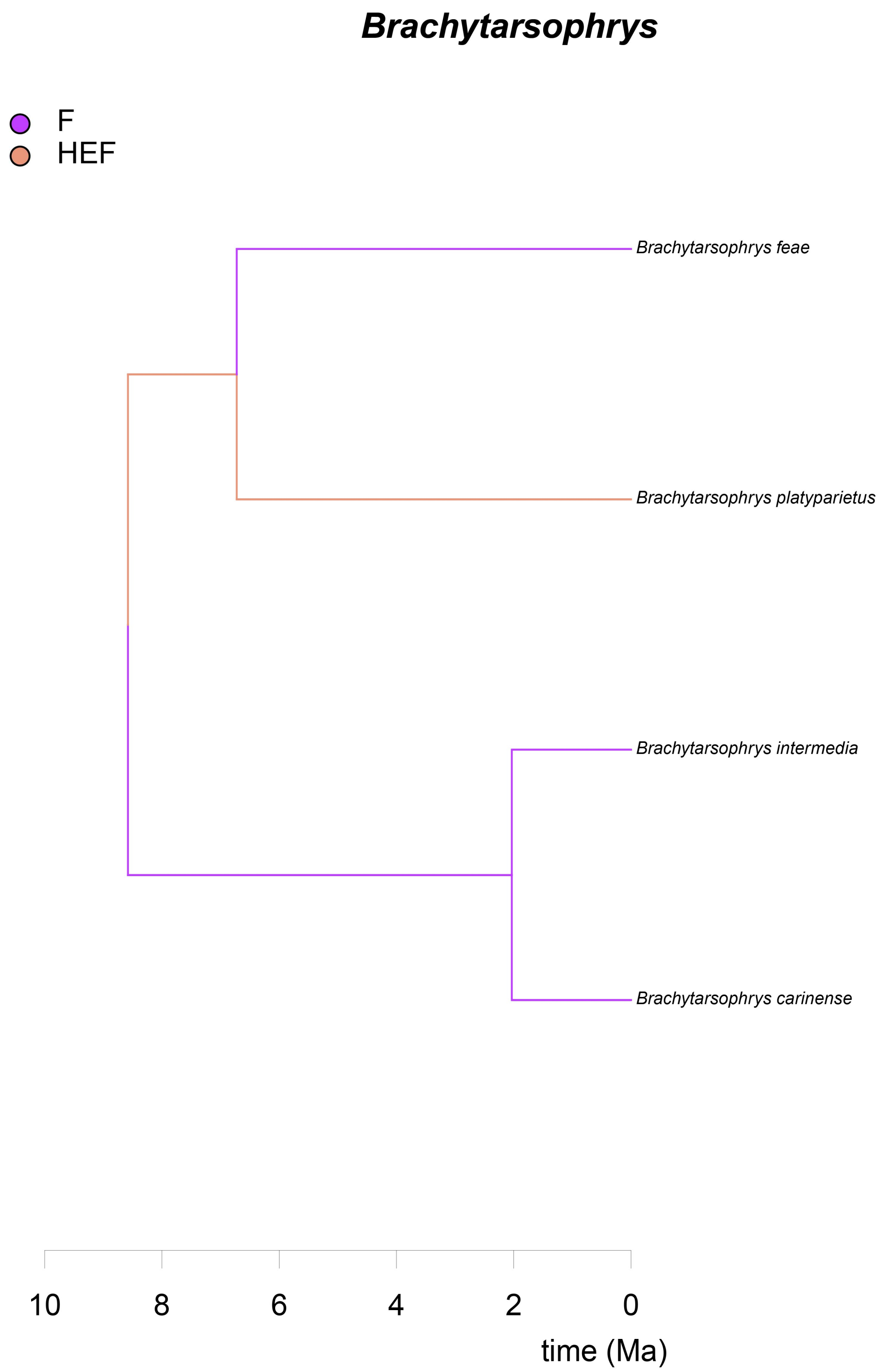

Fig. S11. Marginal maximum *a posteriori* reconstruction of the evolutionary history of geographic range on the maximum clade credibility tree of *Brachytarsophrys* using RevBayes. Labels for geographic regions follow Fig. S2.

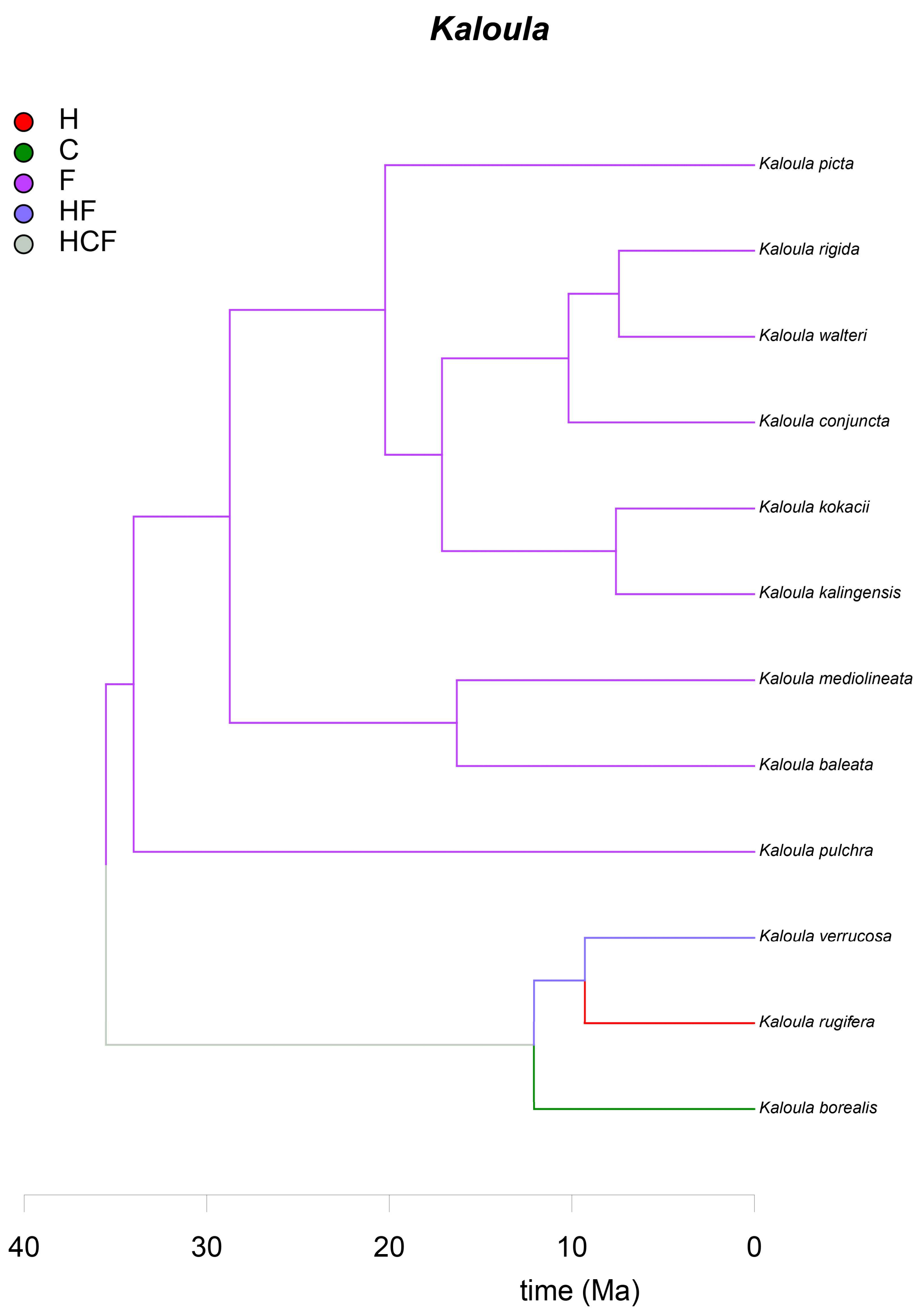

Fig. S12. Marginal maximum *a posteriori* reconstruction of the evolutionary history of geographic range on the maximum clade credibility tree of *Kaloula* using RevBayes. Labels for geographic regions follow Fig. S2.

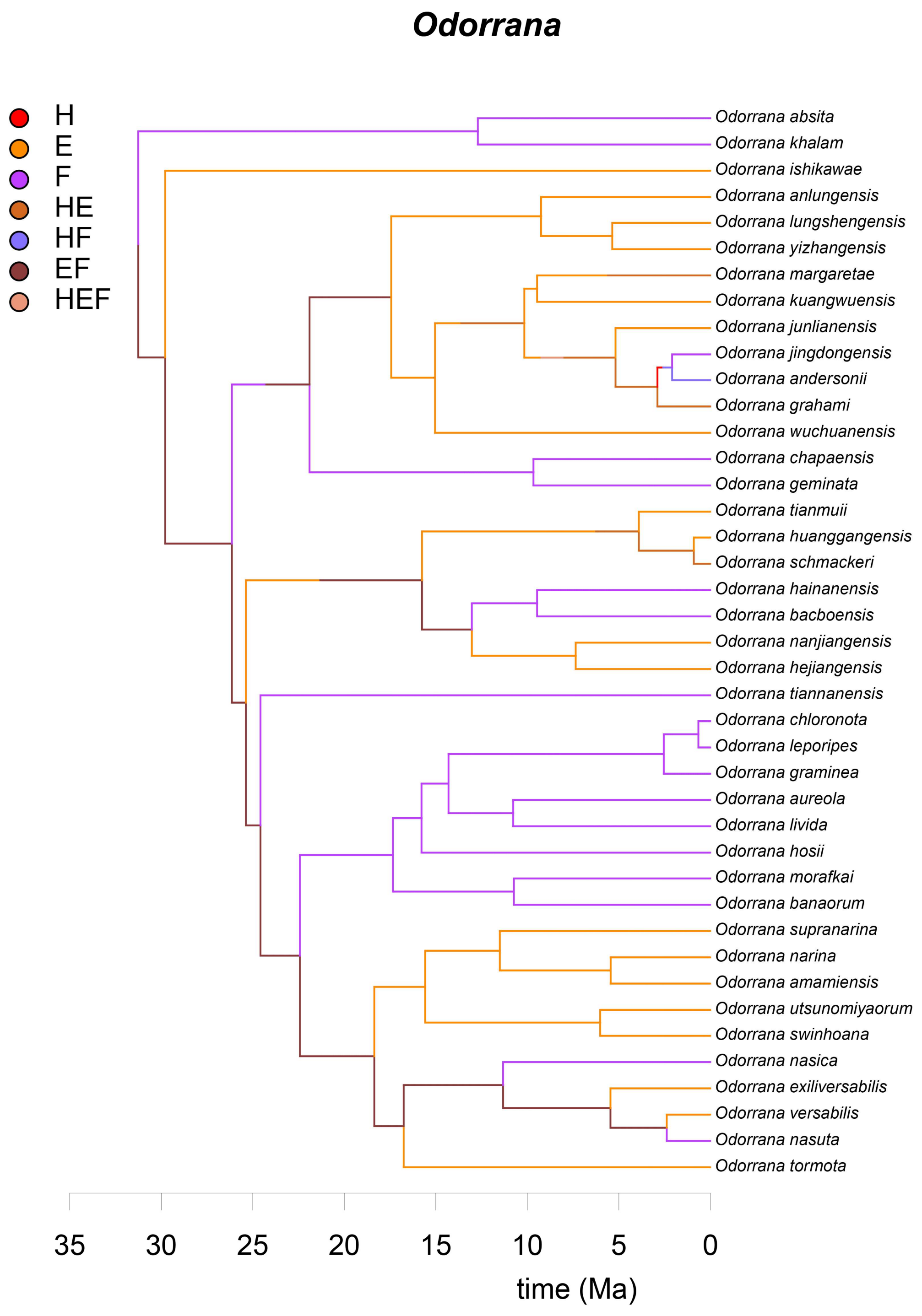

Fig. S13. Marginal maximum *a posteriori* reconstruction of the evolutionary history of geographic range on the maximum clade credibility tree of *Odorrana* using RevBayes. Labels for geographic regions follow Fig. S2.

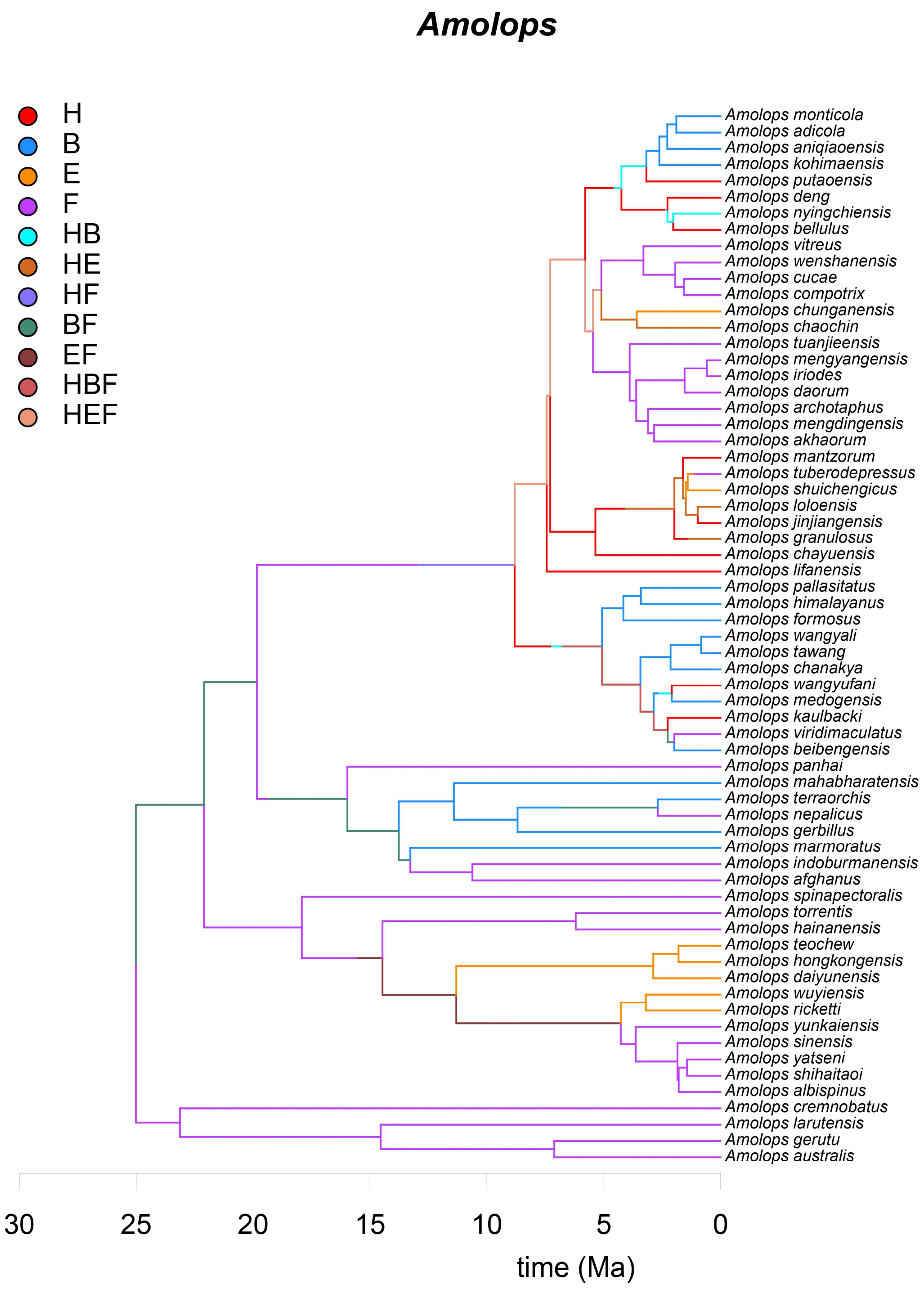

Fig. S14. Marginal maximum *a posteriori* reconstruction of the evolutionary history of geographic range on the maximum clade credibility tree of *Amolops* using RevBayes. Labels for geographic regions follow Fig. S2.

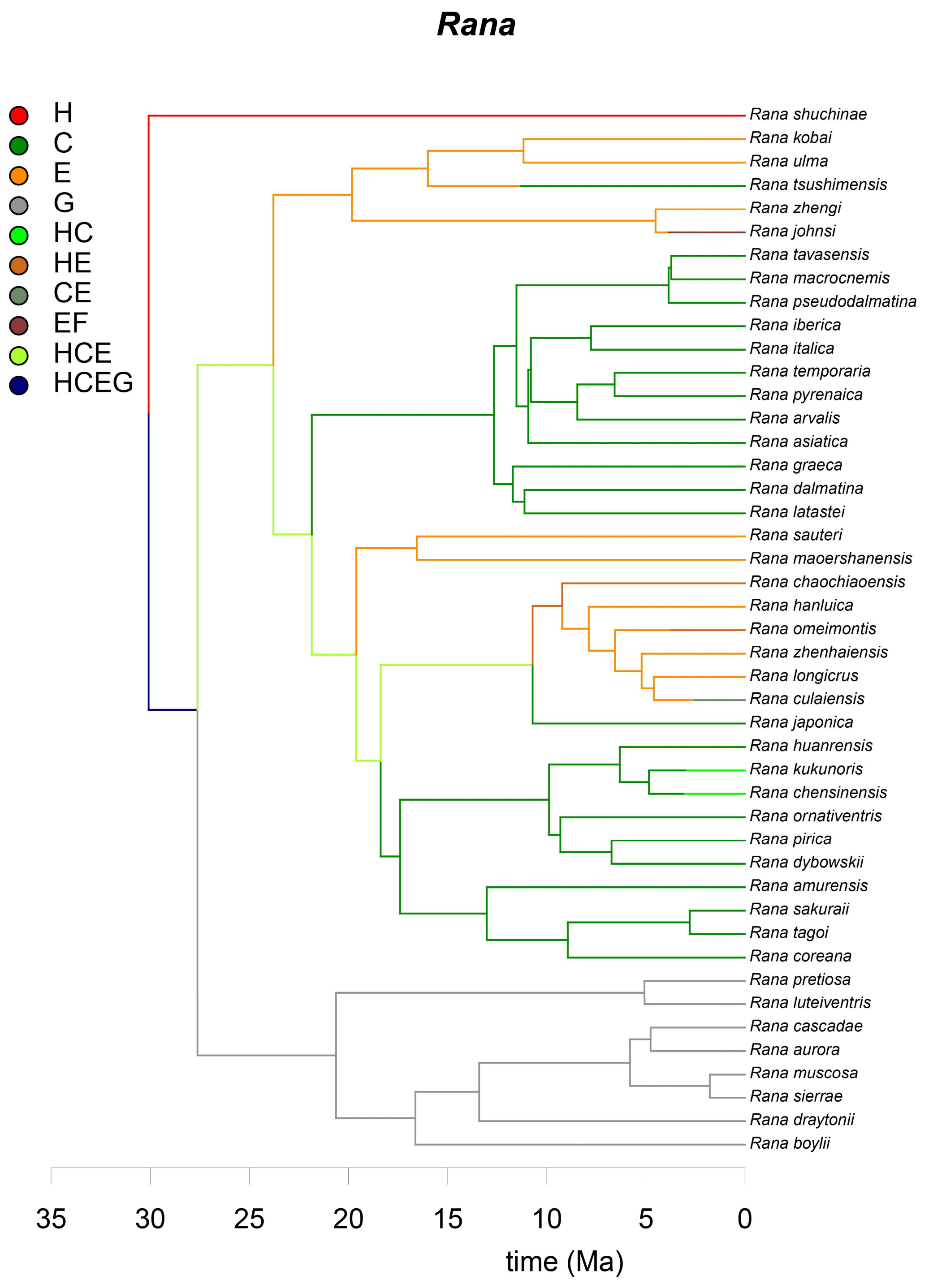

Fig. S15. Marginal maximum *a posteriori* reconstruction of the evolutionary history of geographic range on the maximum clade credibility tree of *Rana* using RevBayes. Labels for geographic regions follow Fig. S2.

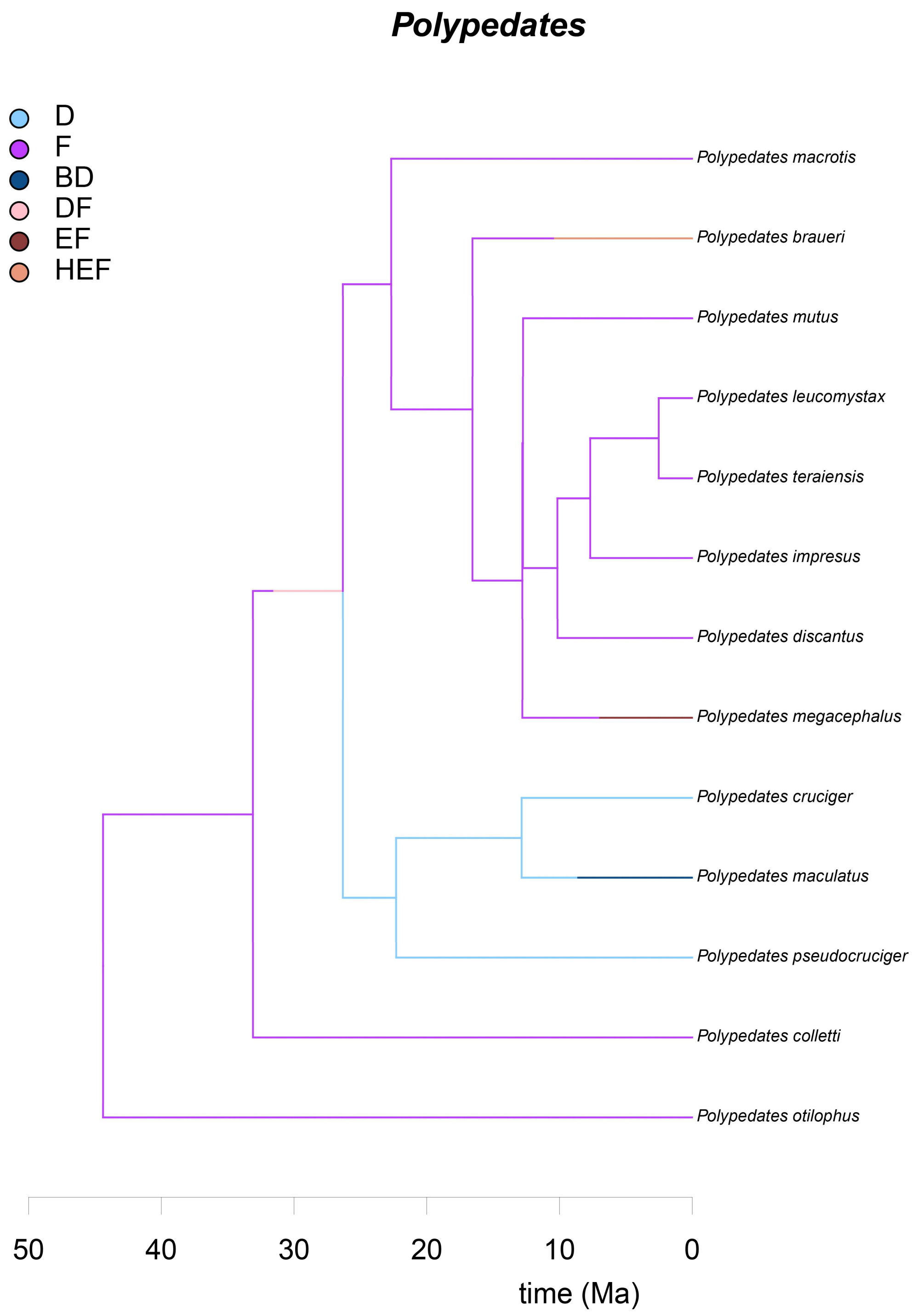

Fig. S16. Marginal maximum *a posteriori* reconstruction of the evolutionary history of geographic range on the maximum clade credibility tree of *Polypedates* using RevBayes. Labels for geographic regions follow Fig. S2.

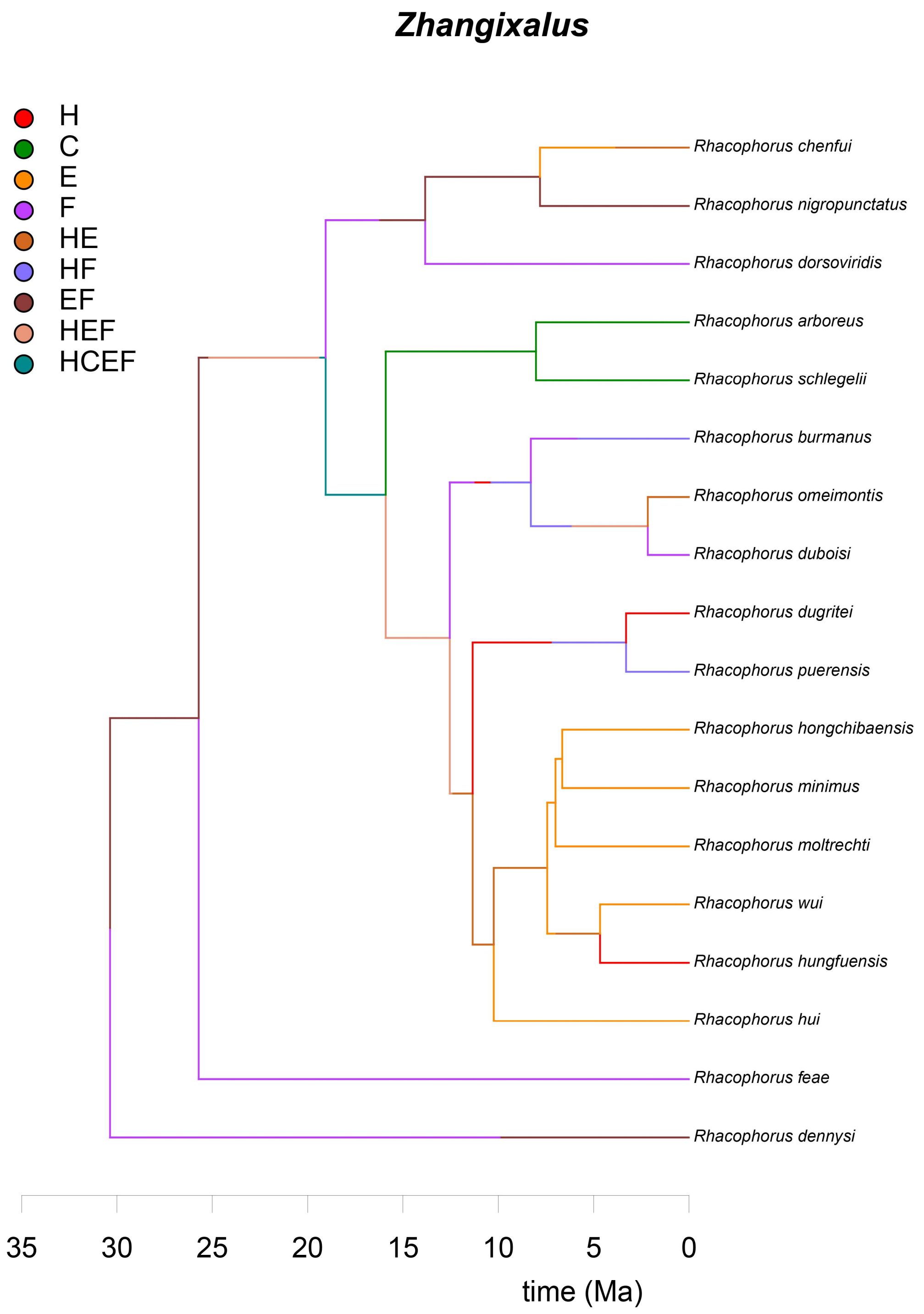

Fig. S17. Marginal maximum *a posteriori* reconstruction of the evolutionary history of geographic range on the maximum clade credibility tree of *Zhangixalus* using RevBayes. Labels for geographic regions follow Fig. S2.

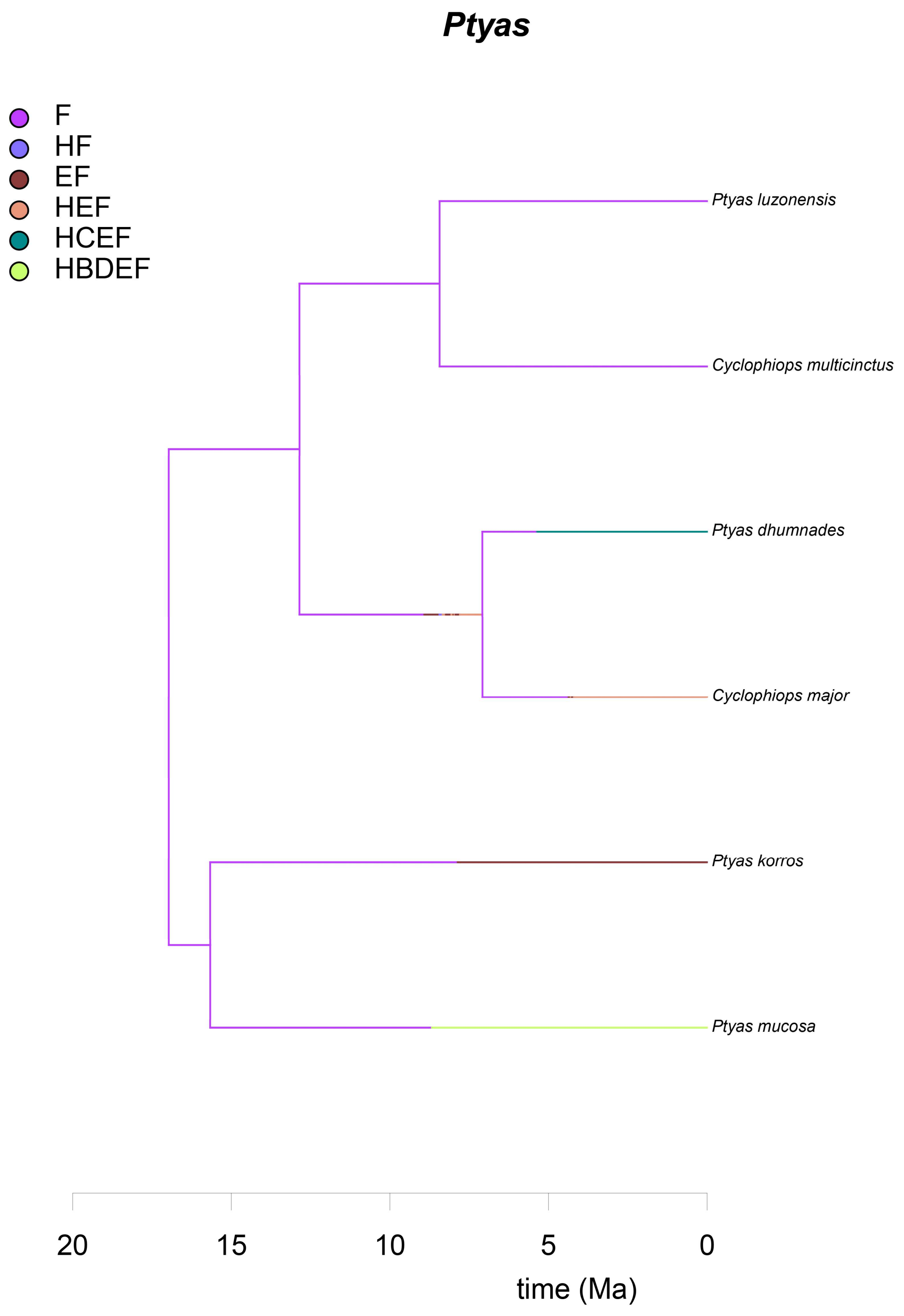

Fig. S18. Marginal maximum *a posteriori* reconstruction of the evolutionary history of geographic range on the maximum clade credibility tree of *Ptyas* using RevBayes. Labels for geographic regions follow Fig. S2.

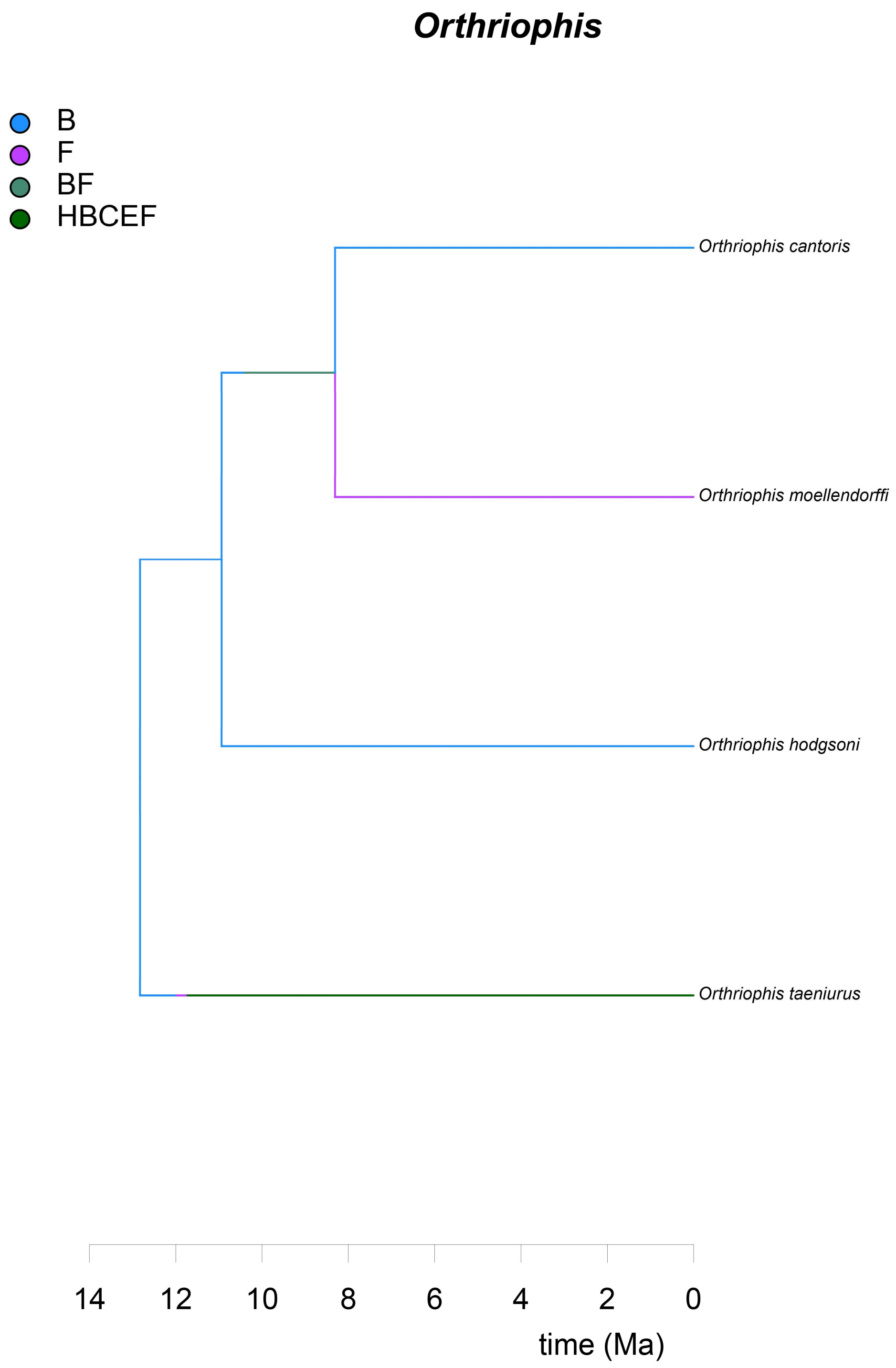

Fig. S19. Marginal maximum *a posteriori* reconstruction of the evolutionary history of geographic range on the maximum clade credibility tree of *Orthriophis* using RevBayes. Labels for geographic regions follow Fig. S2.

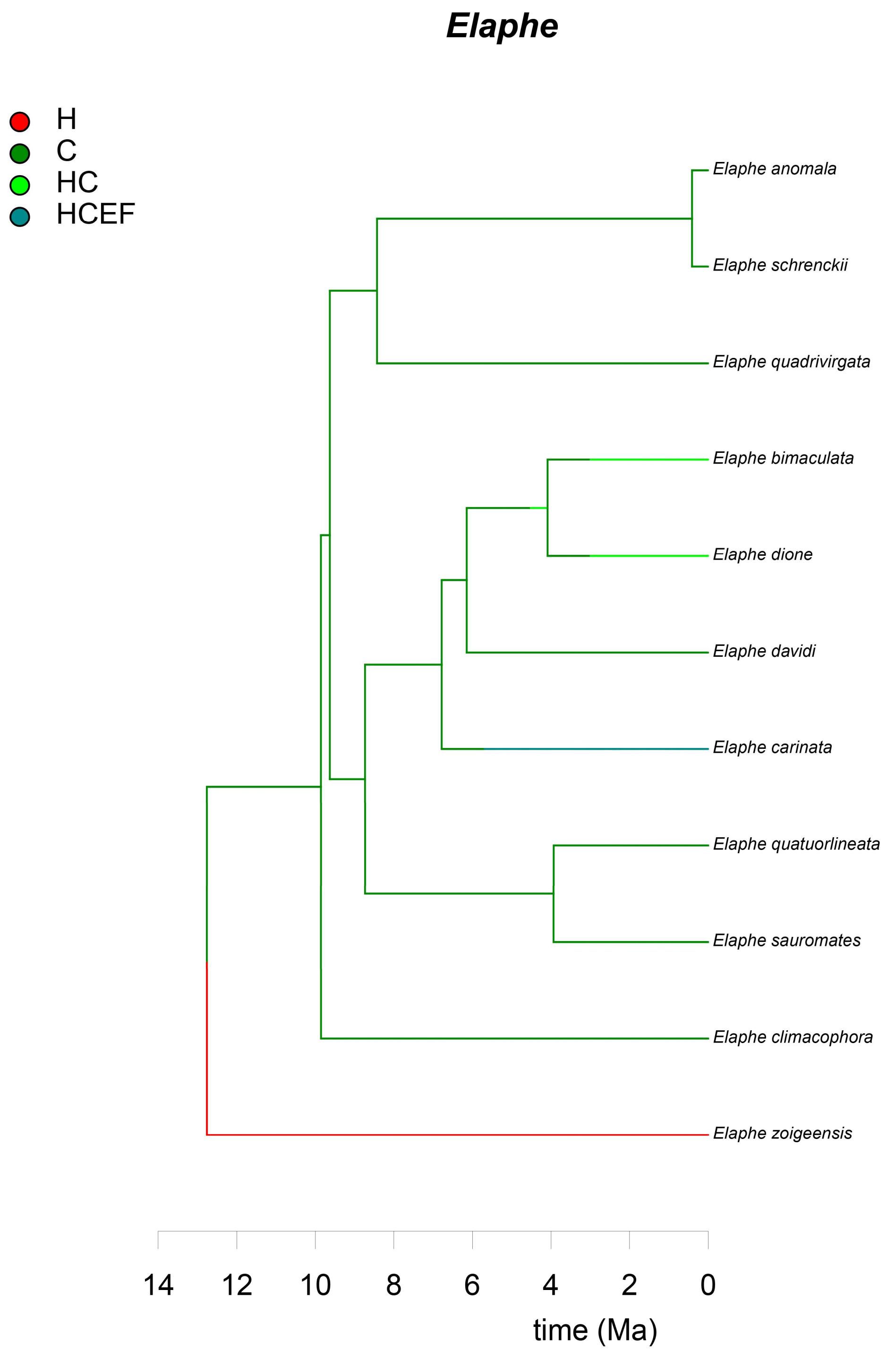

Fig. S20. Marginal maximum *a posteriori* reconstruction of the evolutionary history of geographic range on the maximum clade credibility tree of *Elaphe* using RevBayes. Labels for geographic regions follow Fig. S2.

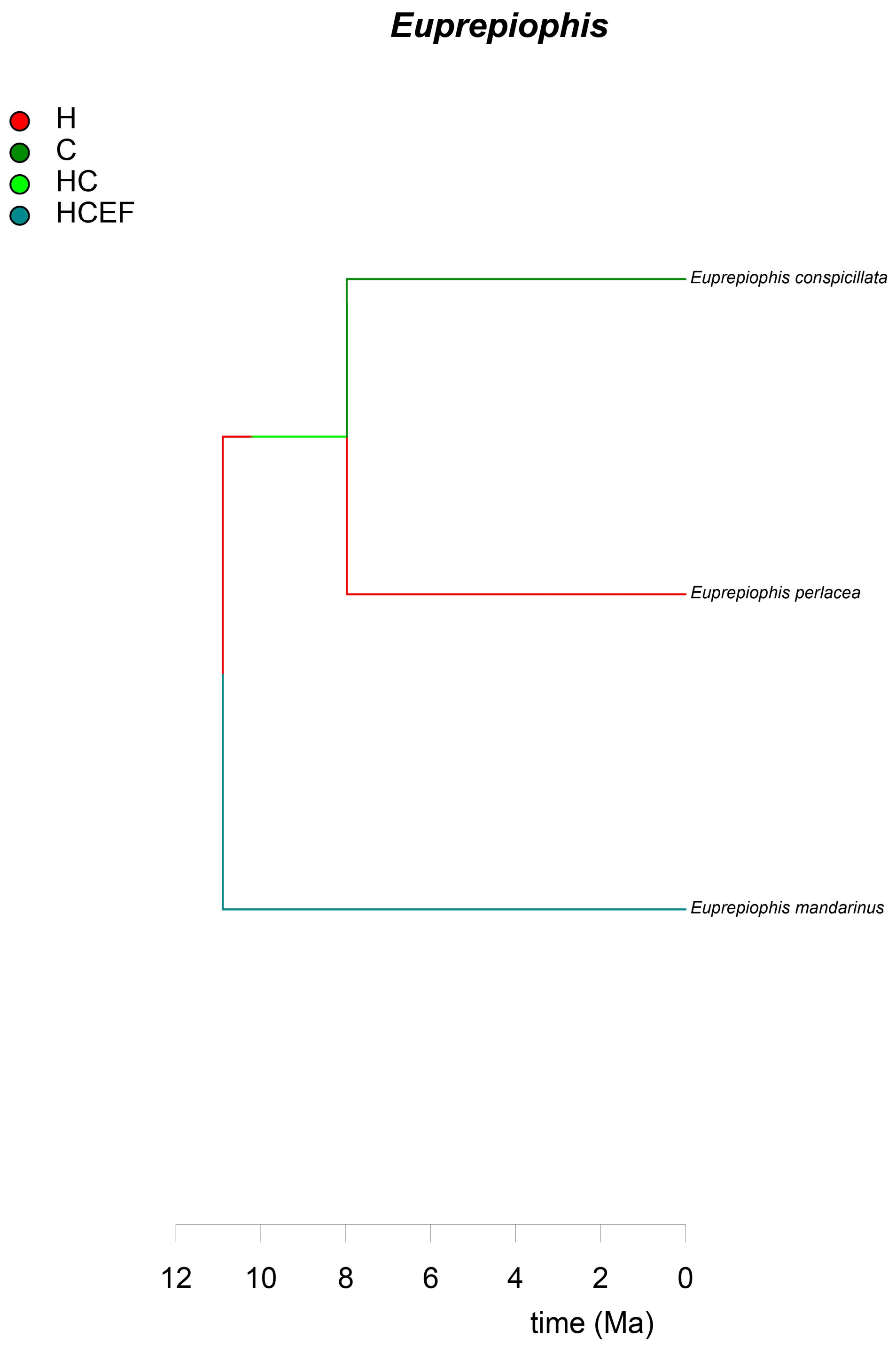

Fig. S21. Marginal maximum *a posteriori* reconstruction of the evolutionary history of geographic range on the maximum clade credibility tree of *Euprepiophis* using RevBayes. Labels for geographic regions follow Fig. S2.

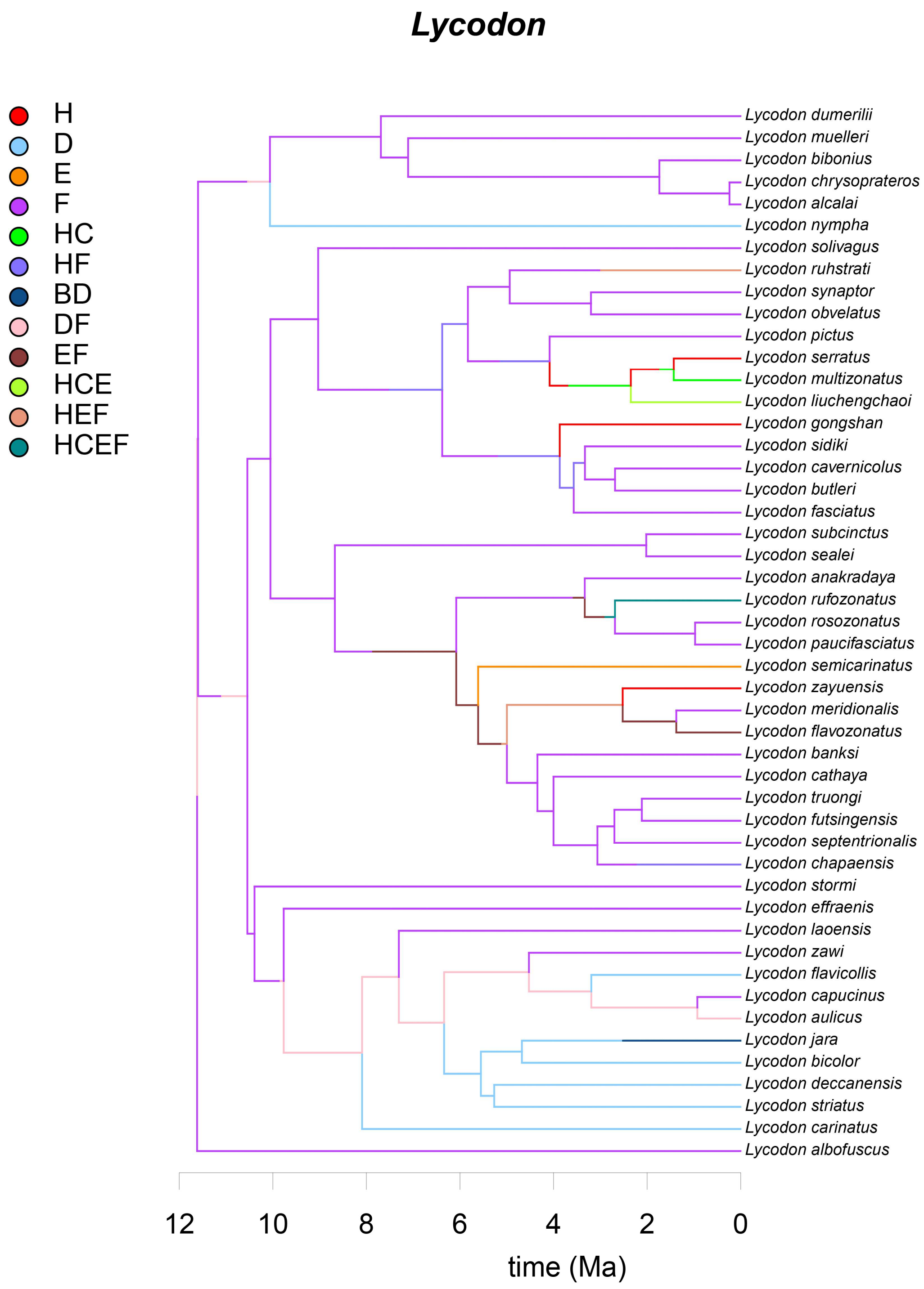

Fig. S22. Marginal maximum *a posteriori* reconstruction of the evolutionary history of geographic range on the maximum clade credibility tree of *Lycodon* using RevBayes. Labels for geographic regions follow Fig. S2.

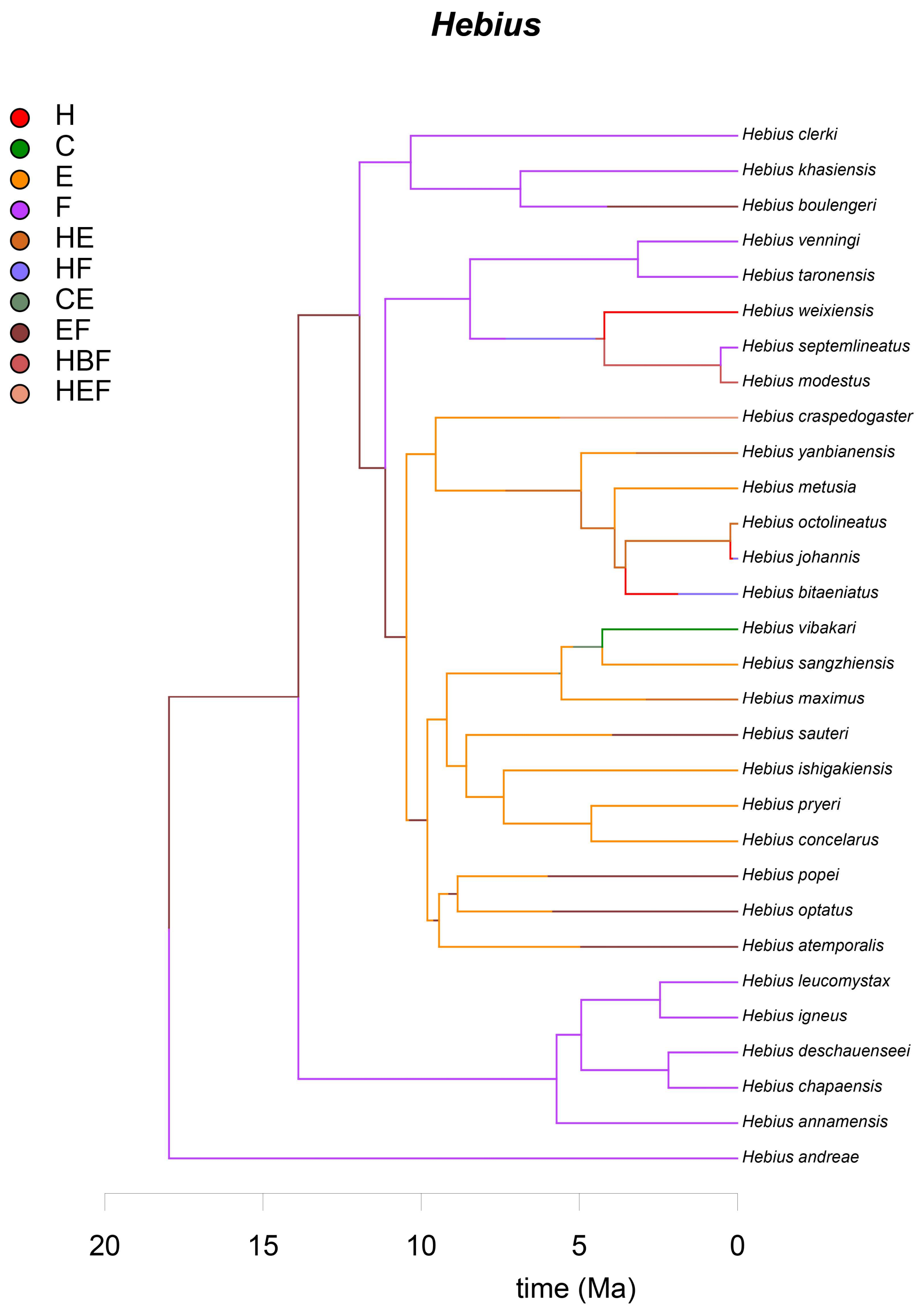

Fig. S23. Marginal maximum *a posteriori* reconstruction of the evolutionary history of geographic range on the maximum clade credibility tree of *Hebius* using RevBayes. Labels for geographic regions follow Fig. S2.

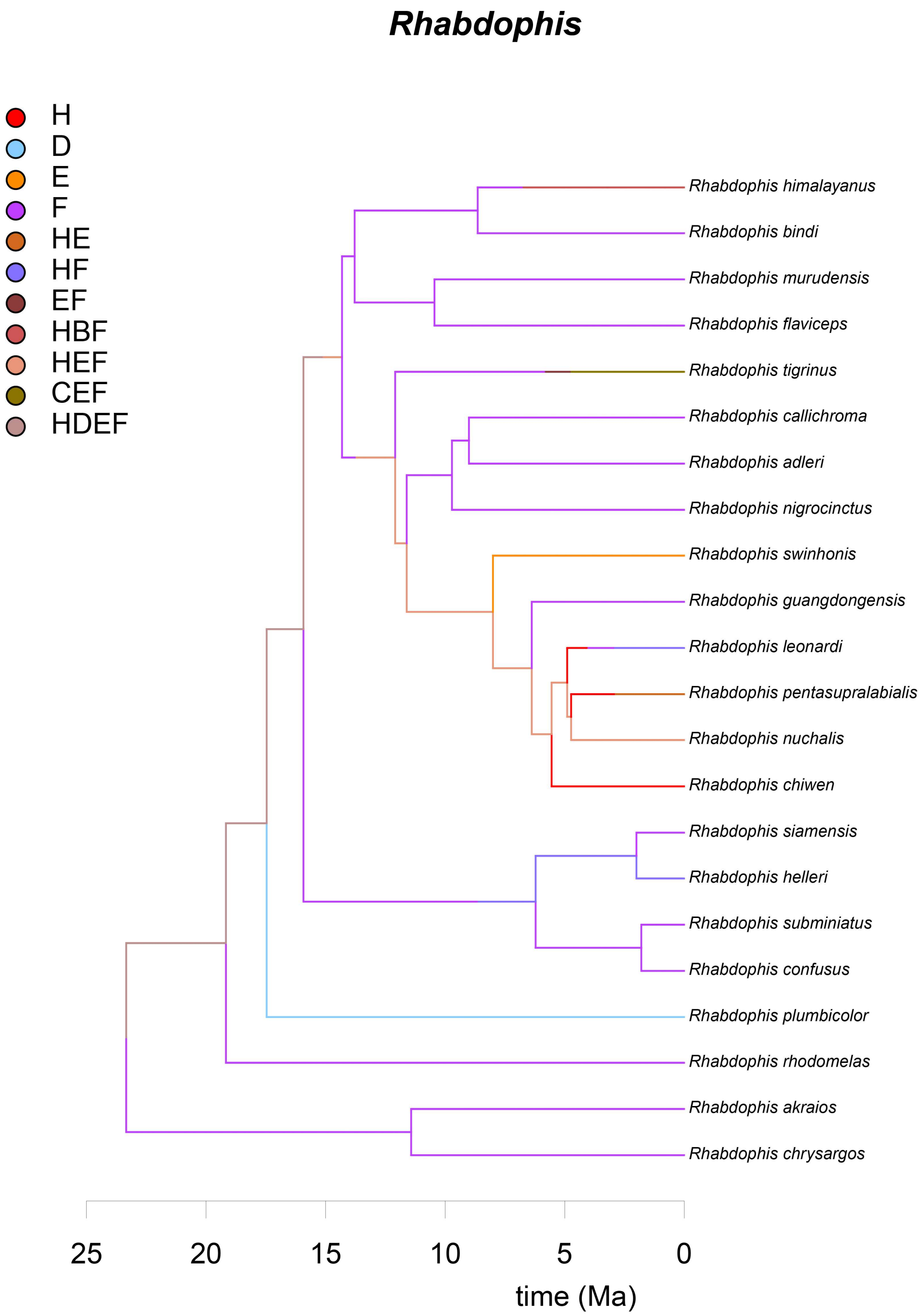

Fig. S24. Marginal maximum *a posteriori* reconstruction of the evolutionary history of geographic range on the maximum clade credibility tree of *Rhabdophis* using RevBayes. Labels for geographic regions follow Fig. S2.

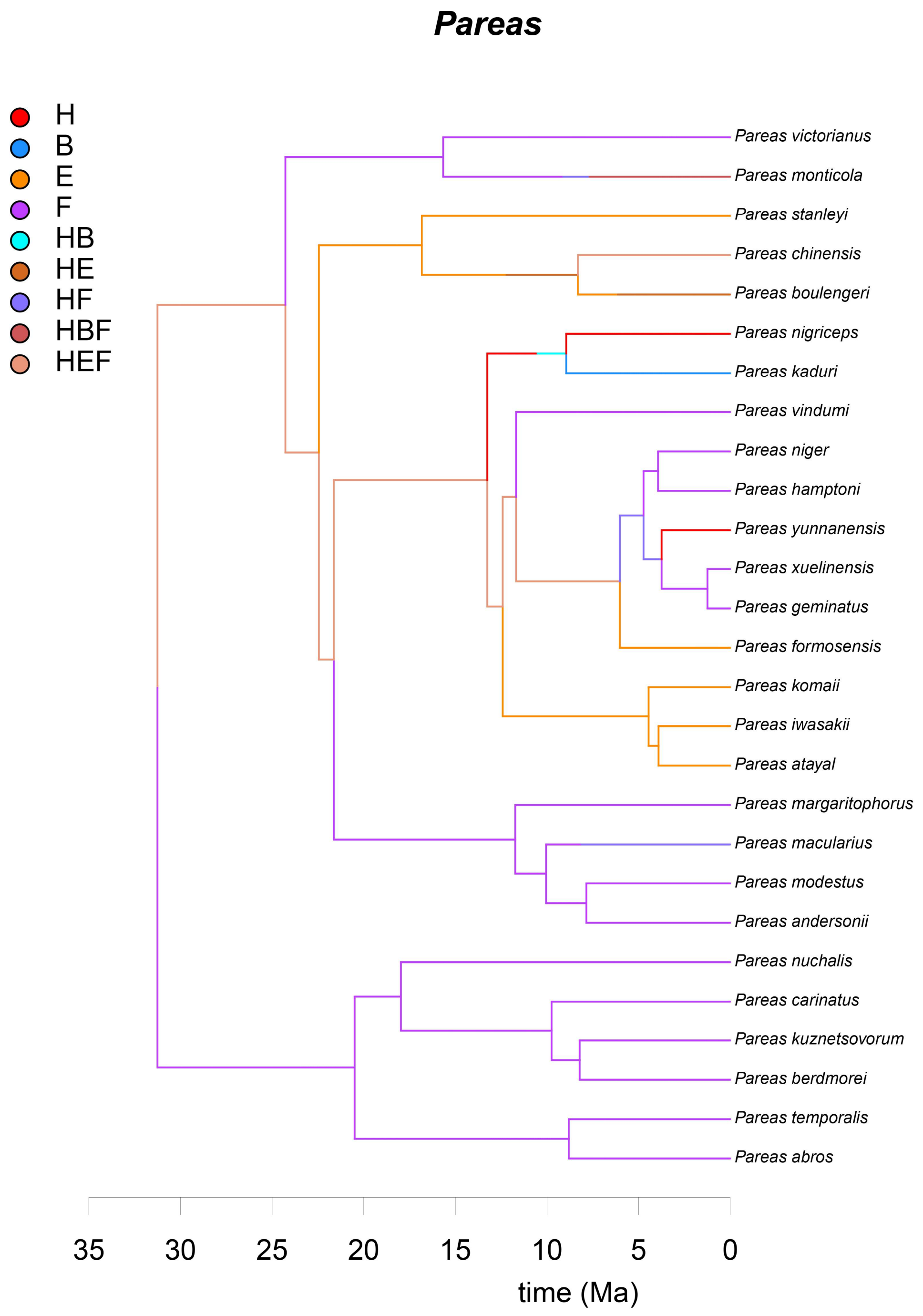

Fig. S25. Marginal maximum *a posteriori* reconstruction of the evolutionary history of geographic range on the maximum clade credibility tree of *Pareas* using RevBayes. Labels for geographic regions follow Fig. S2.

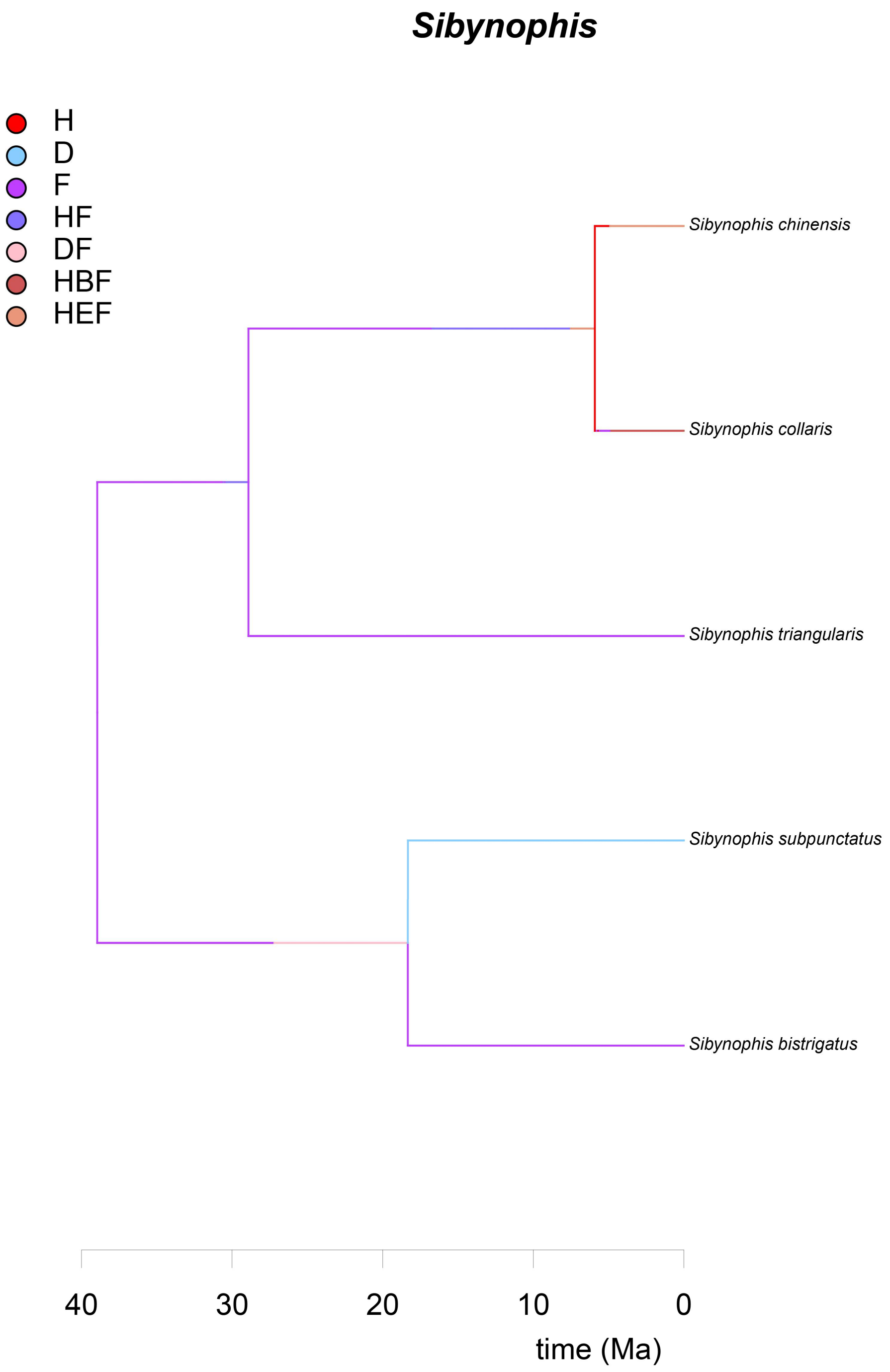

Fig. S26. Marginal maximum *a posteriori* reconstruction of the evolutionary history of geographic range on the maximum clade credibility tree of *Sibynophis* using RevBayes. Labels for geographic regions follow Fig. S2.

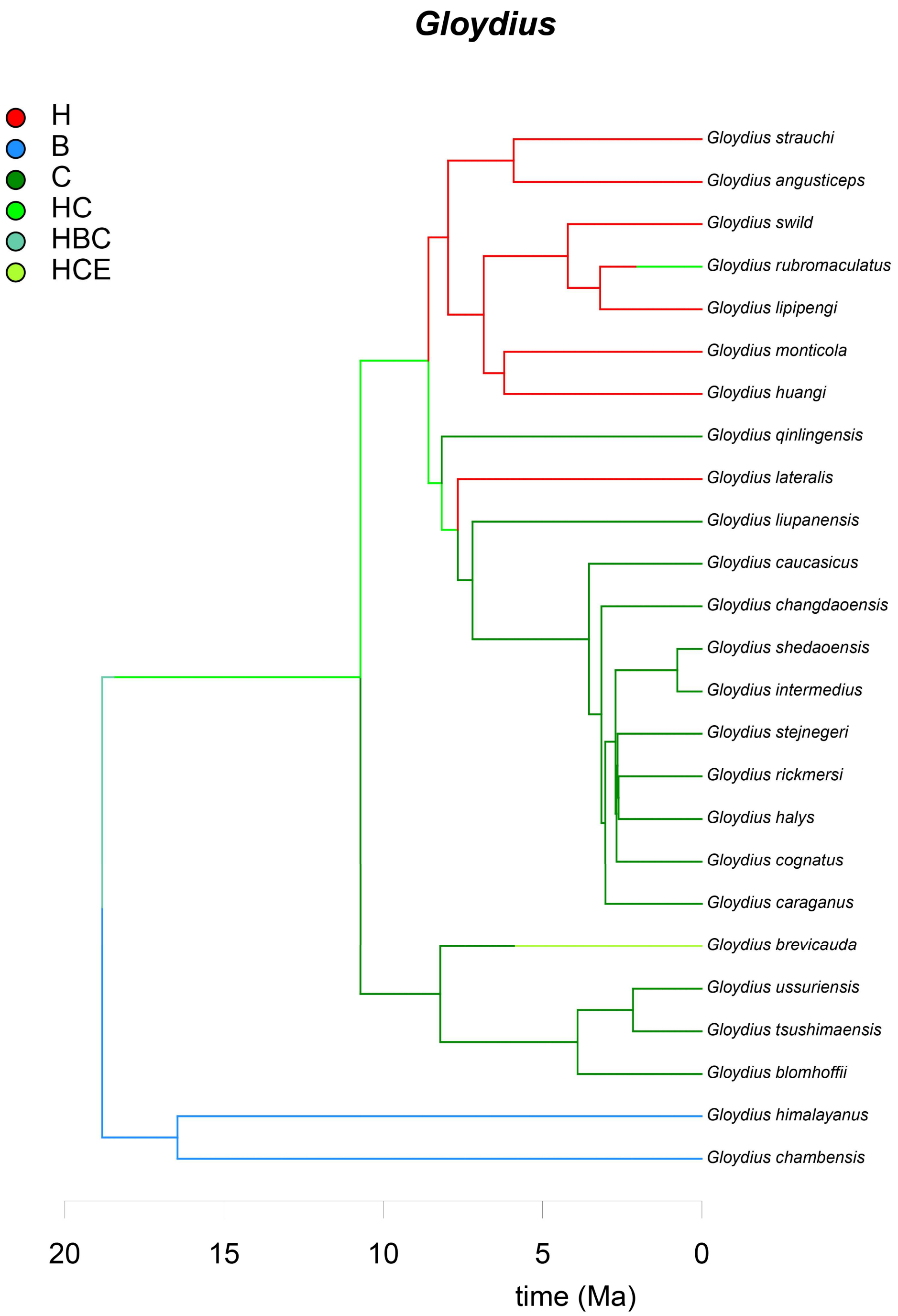

Fig. S27. Marginal maximum *a posteriori* reconstruction of the evolutionary history of geographic range on the maximum clade credibility tree of *Gloydius* using RevBayes. Labels for geographic regions follow Fig. S2.

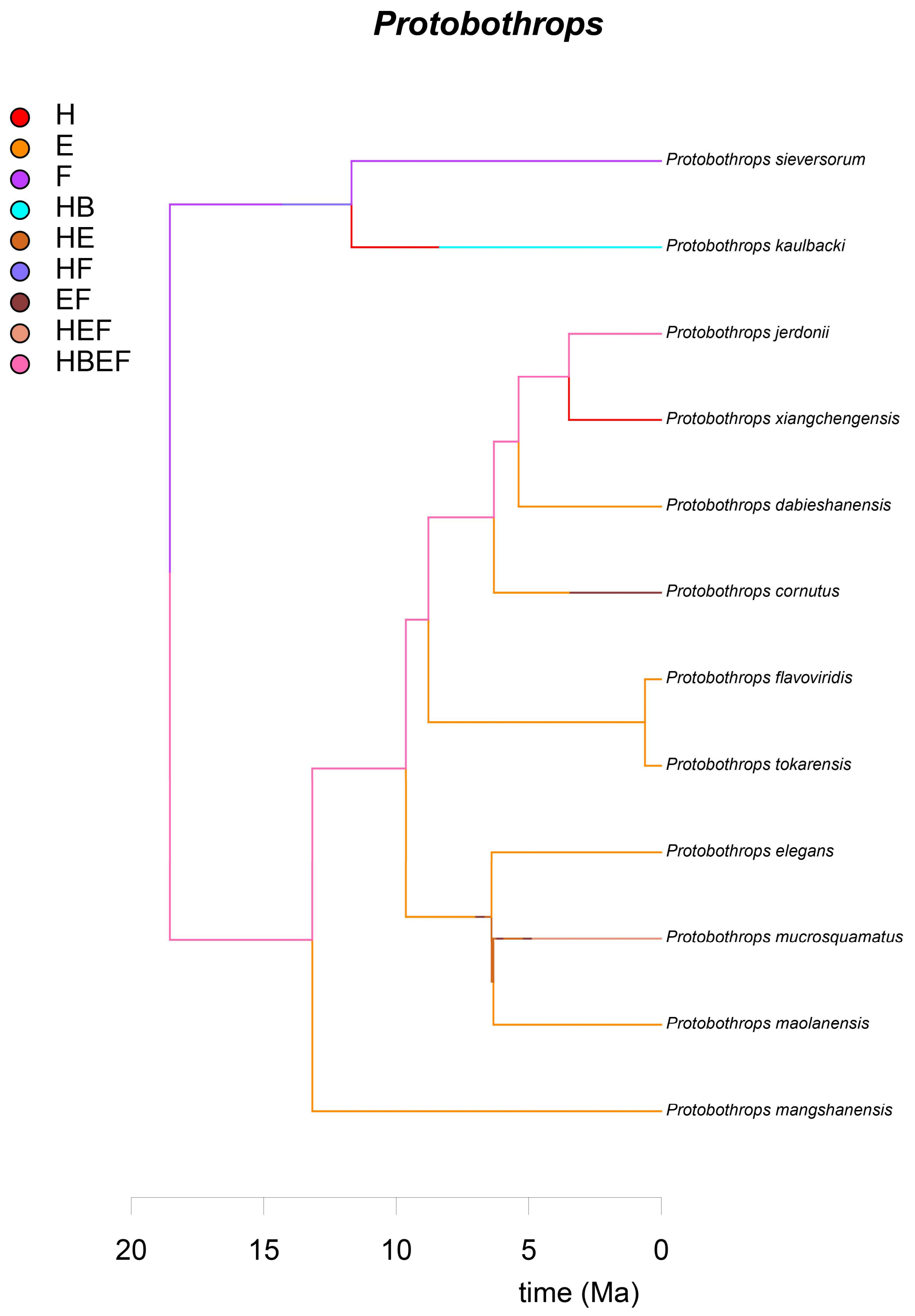

Fig. S28. Marginal maximum *a posteriori* reconstruction of the evolutionary history of geographic range on the maximum clade credibility tree of *Protobothrops* using RevBayes. Labels for geographic regions follow Fig. S2.

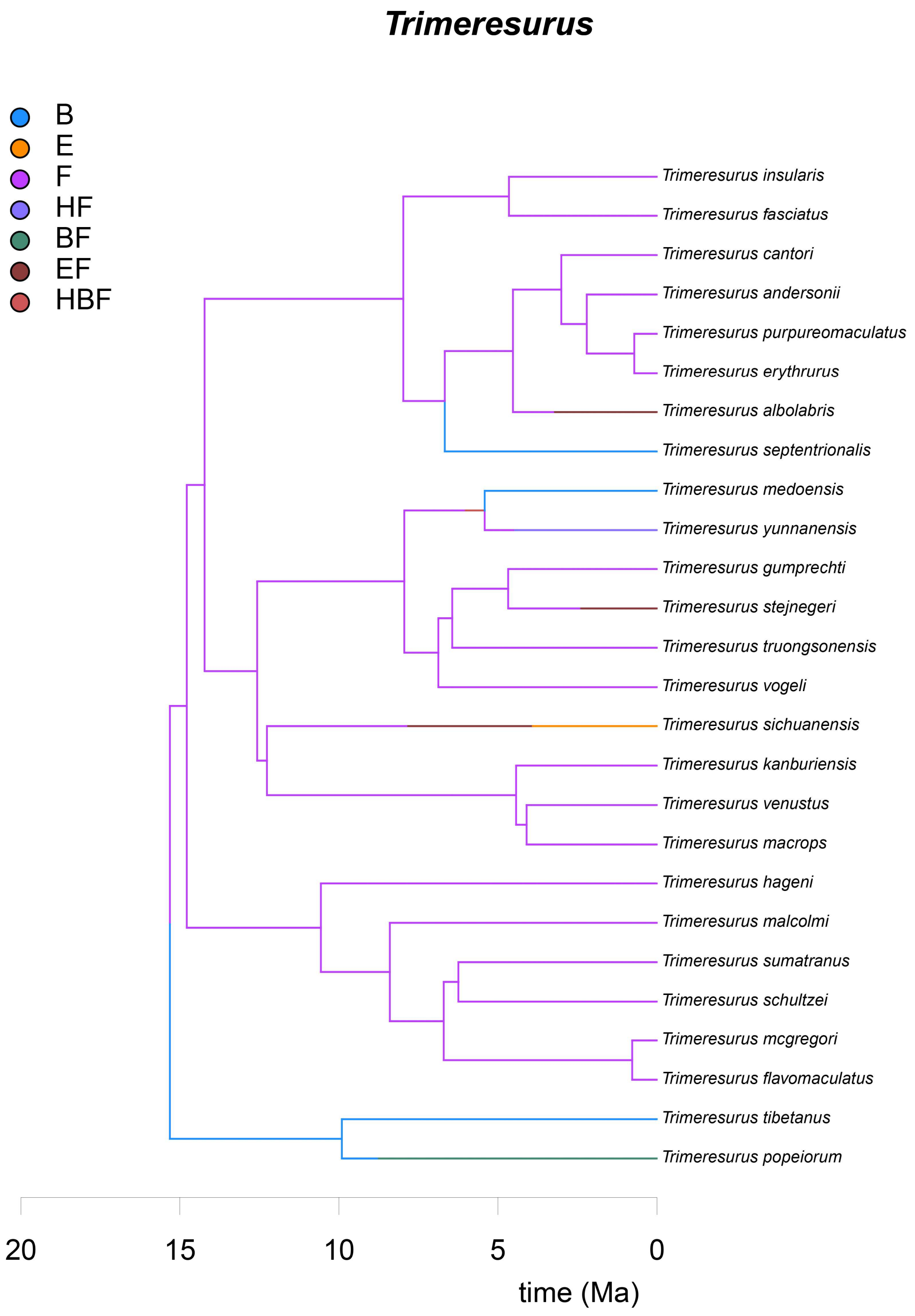

Fig. S29. Marginal maximum *a posteriori* reconstruction of the evolutionary history of geographic range on the maximum clade credibility tree of *Trimeresurus* using RevBayes. Labels for geographic regions follow Fig. S2.

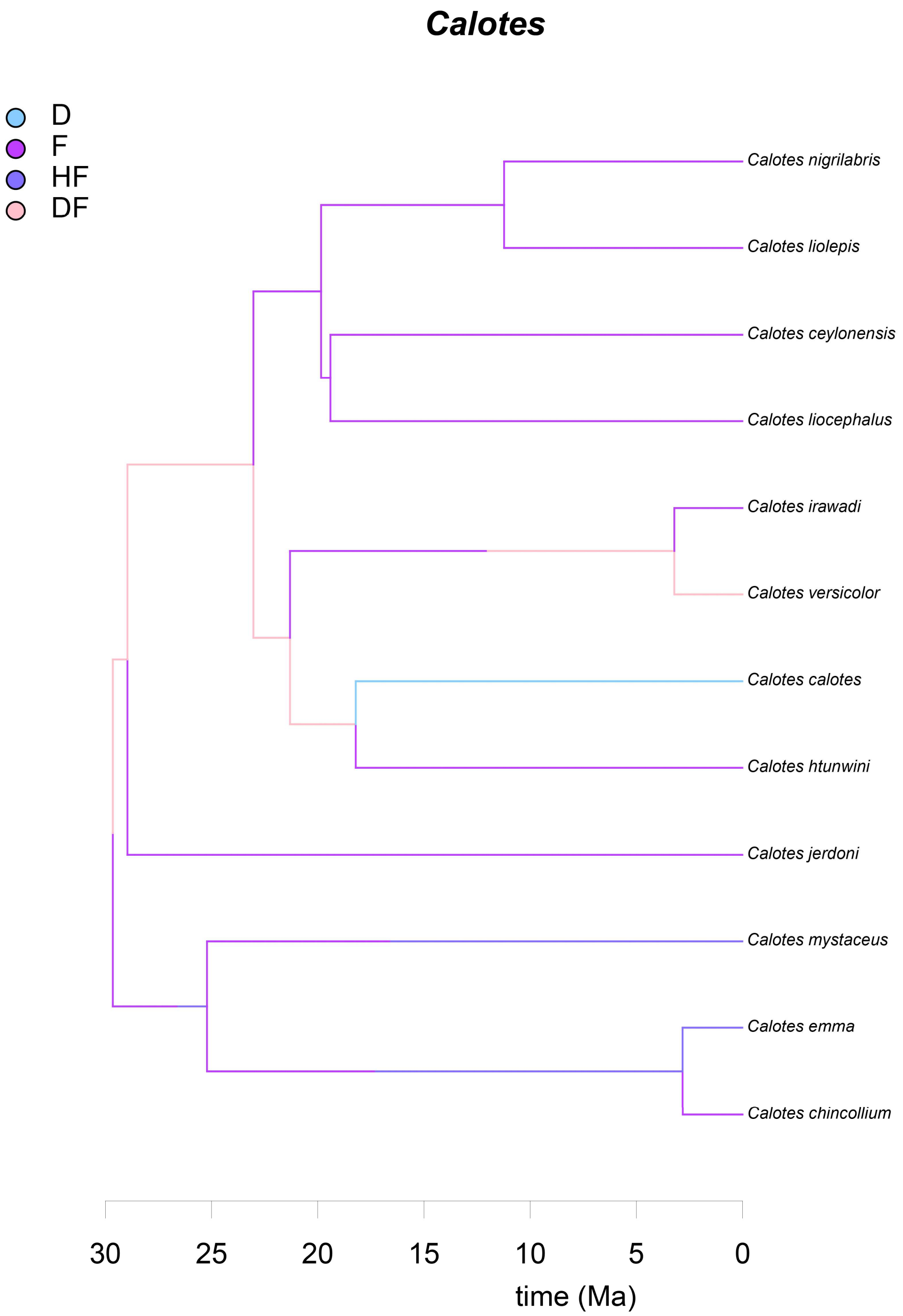

Fig. S30. Marginal maximum *a posteriori* reconstruction of the evolutionary history of geographic range on the maximum clade credibility tree of *Calotes* using RevBayes. Labels for geographic regions follow Fig. S2.

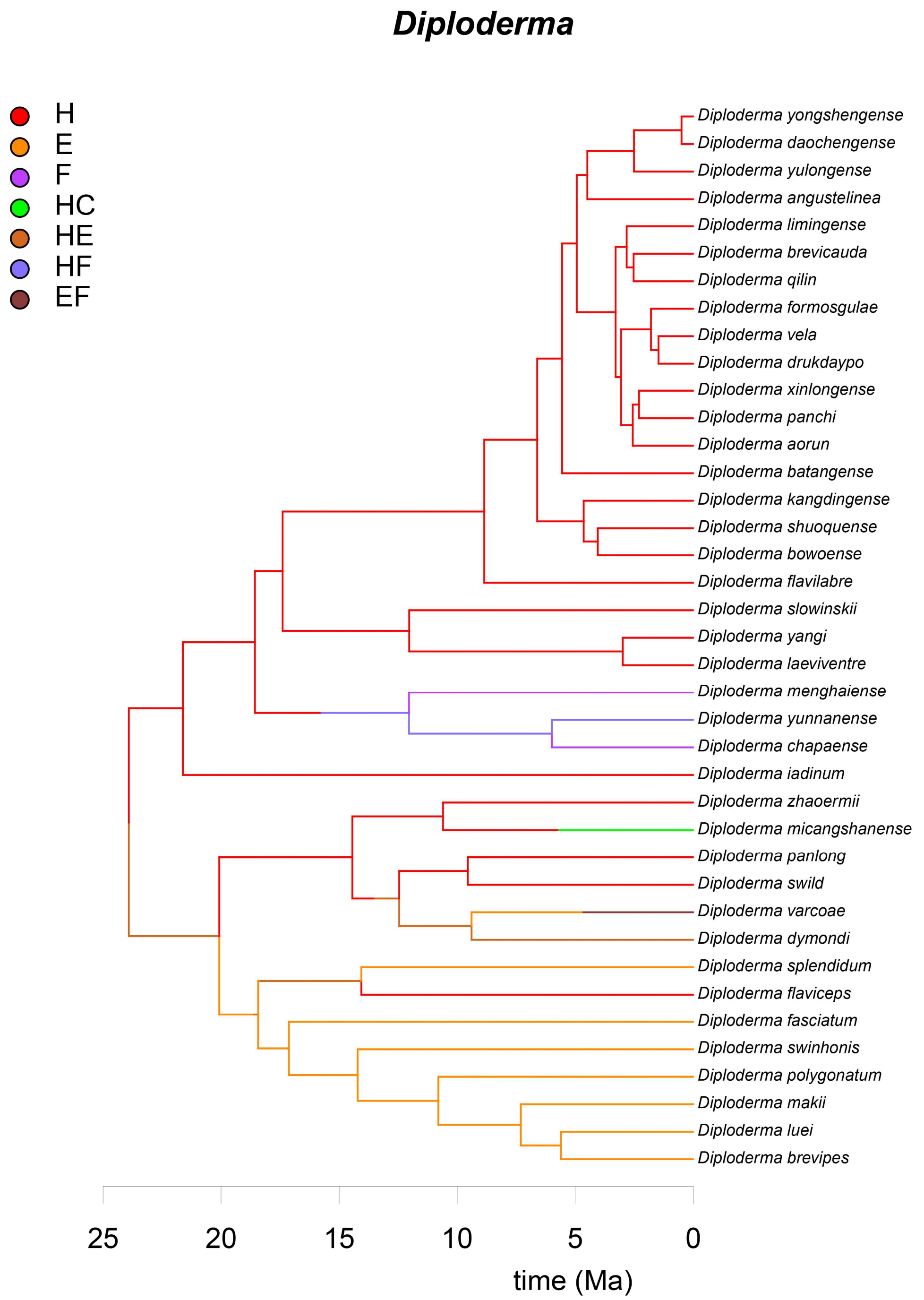

Fig. S31. Marginal maximum *a posteriori* reconstruction of the evolutionary history of geographic range on the maximum clade credibility tree of *Diploderma* using RevBayes. Labels for geographic regions follow Fig. S2.

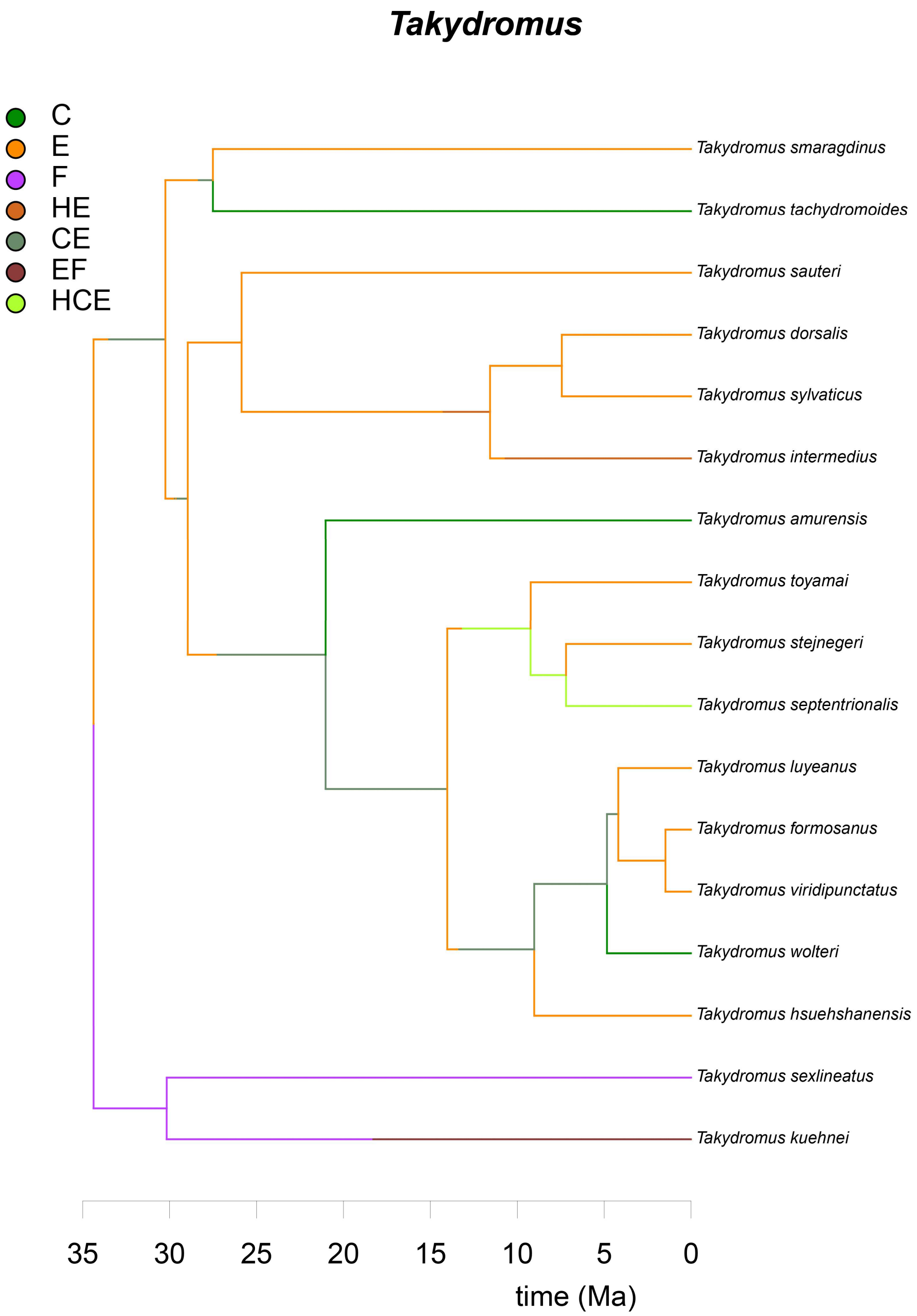

Fig. S32. Marginal maximum *a posteriori* reconstruction of the evolutionary history of geographic range on the maximum clade credibility tree of *Takydromus* using RevBayes. Labels for geographic regions follow Fig. S2.

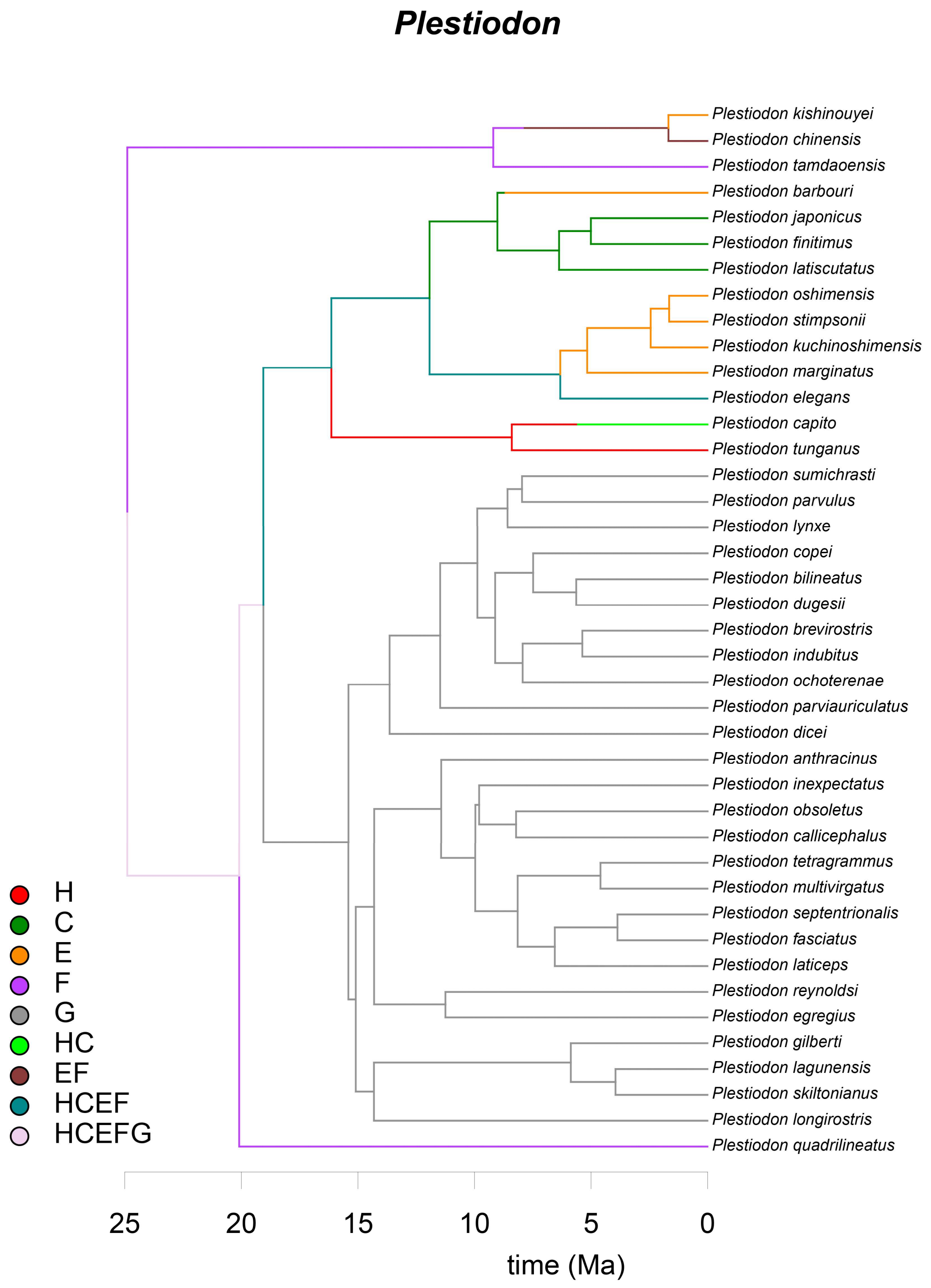

Fig. S33. Marginal maximum *a posteriori* reconstruction of the evolutionary history of geographic range on the maximum clade credibility tree of *Plestiodon* using RevBayes. Labels for geographic regions follow Fig. S2.

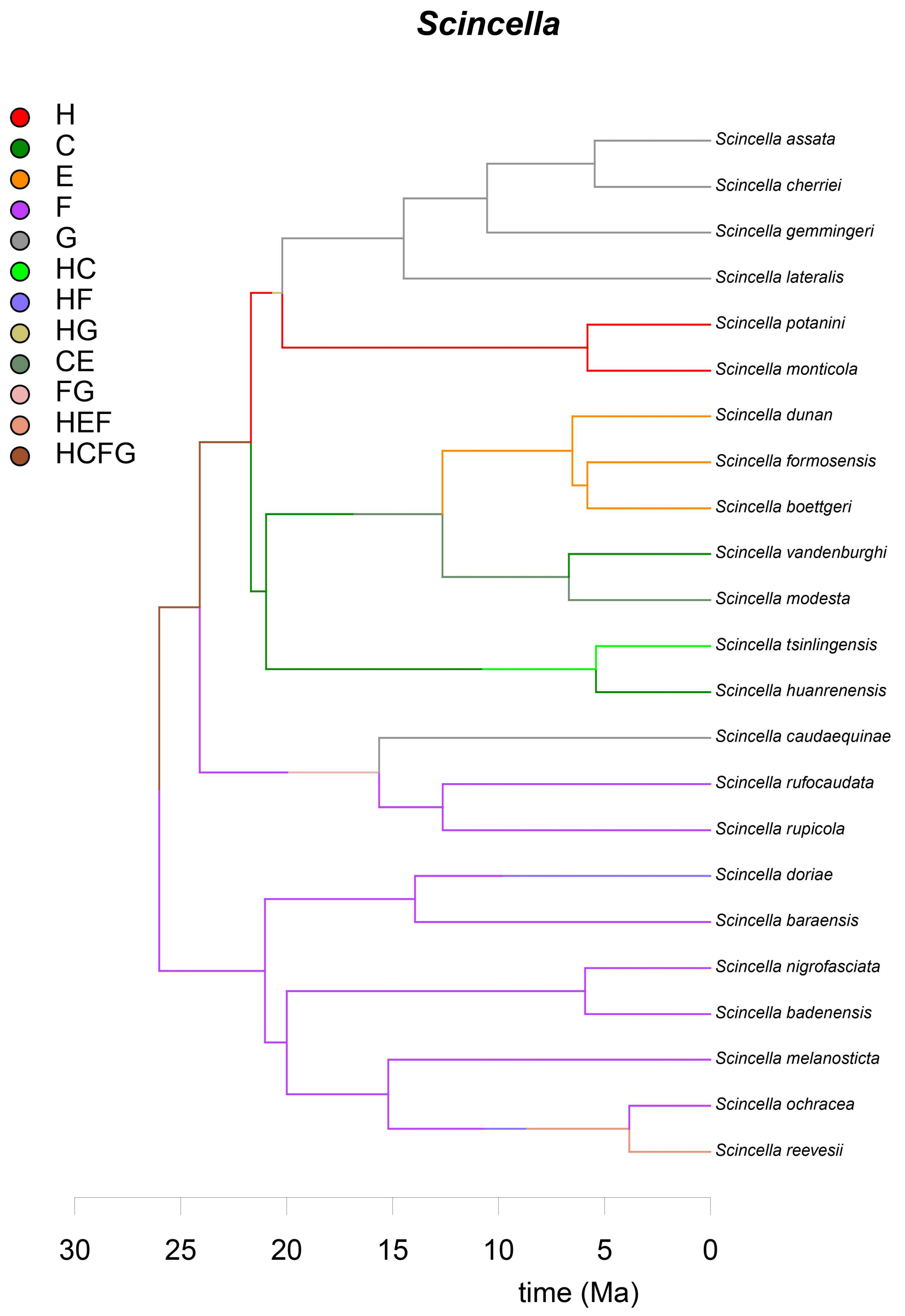

Fig. S34. Marginal maximum *a posteriori* reconstruction of the evolutionary history of geographic range on the maximum clade credibility tree of *Scincella* using RevBayes. Labels for geographic regions follow Fig. S2.

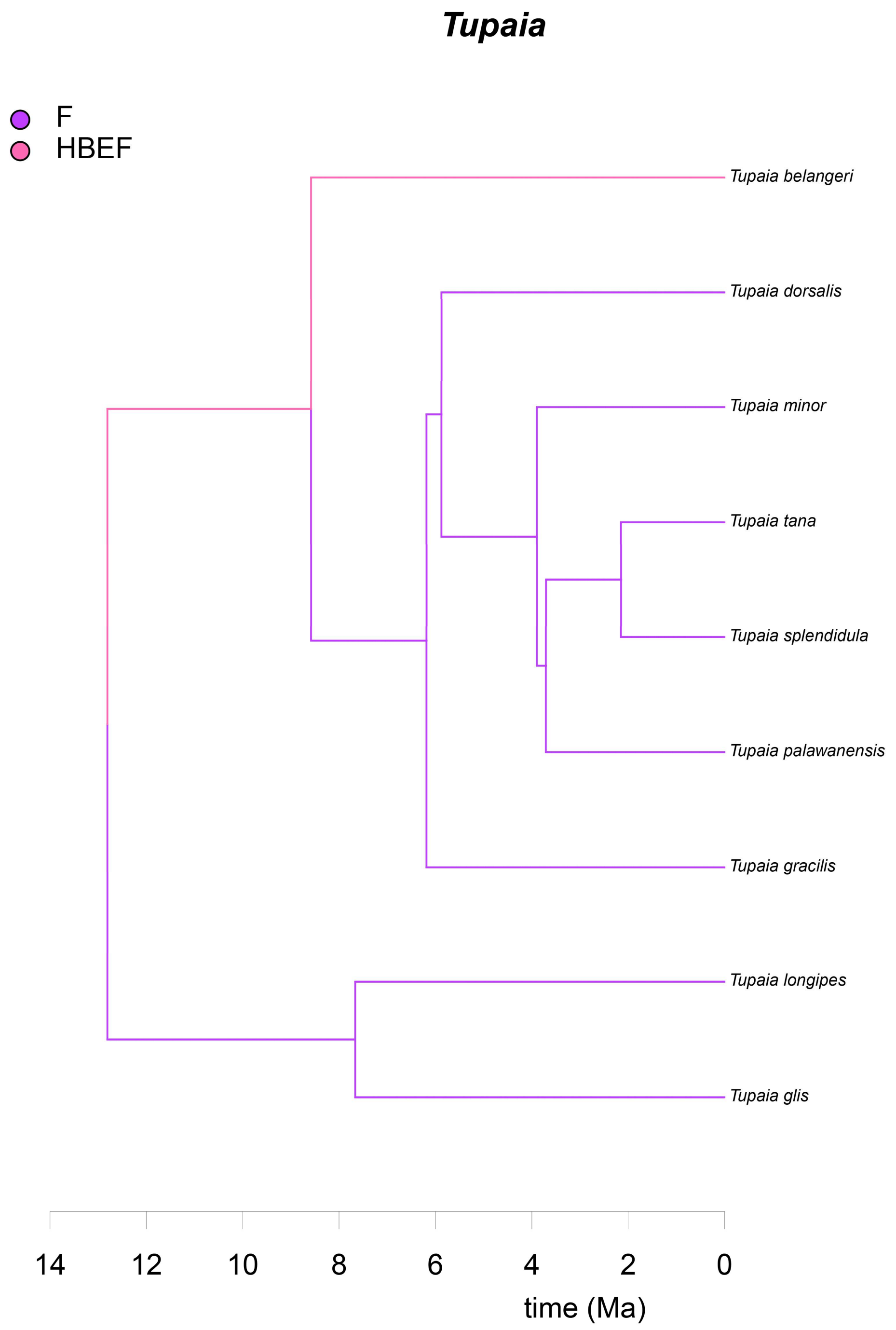

Fig. S35. Marginal maximum *a posteriori* reconstruction of the evolutionary history of geographic range on the maximum clade credibility tree of *Tupaia* using RevBayes. Labels for geographic regions follow Fig. S2.

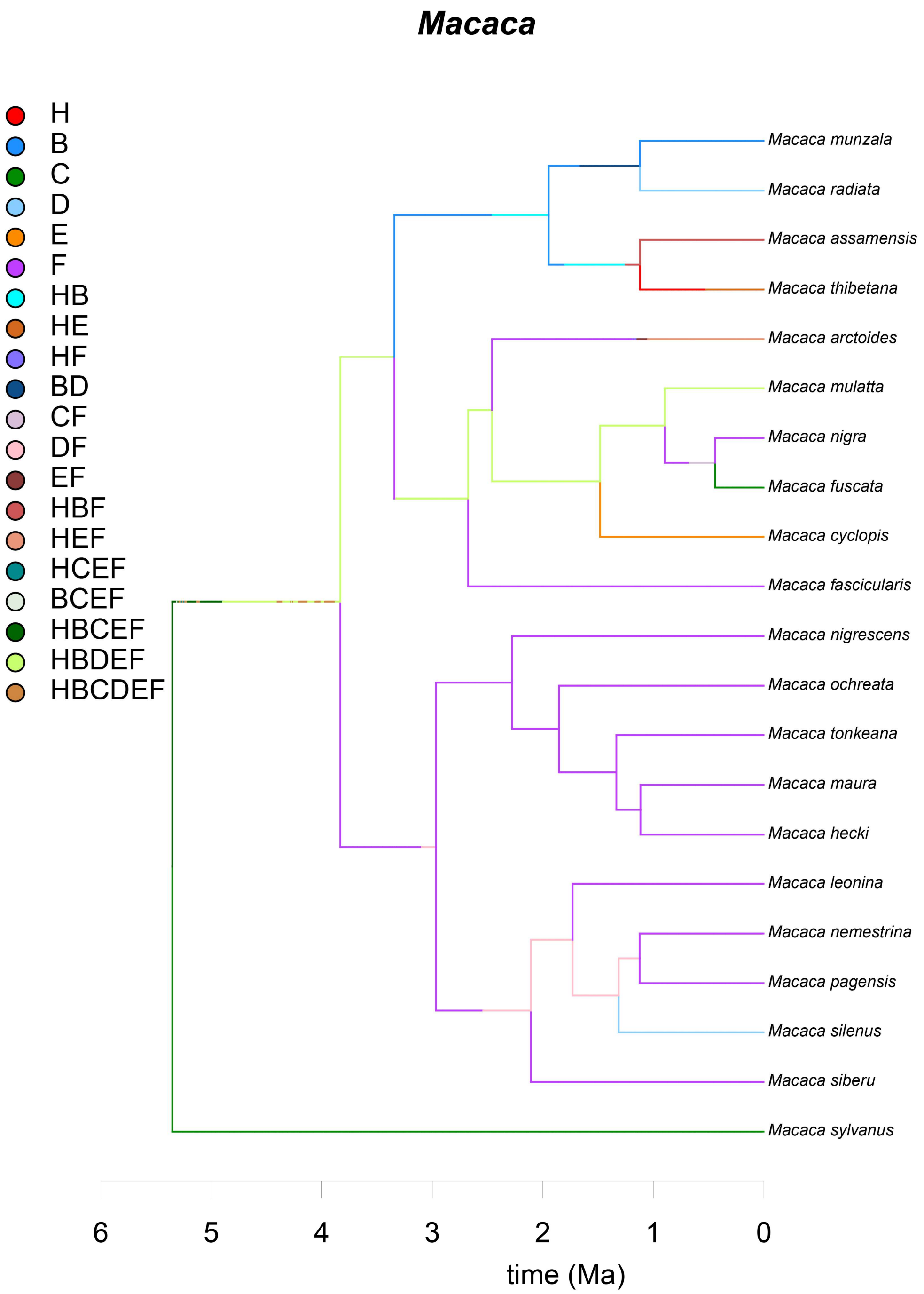

Fig. S36. Marginal maximum *a posteriori* reconstruction of the evolutionary history of geographic range on the maximum clade credibility tree of *Macaca* using RevBayes. Labels for geographic regions follow Fig. S2.

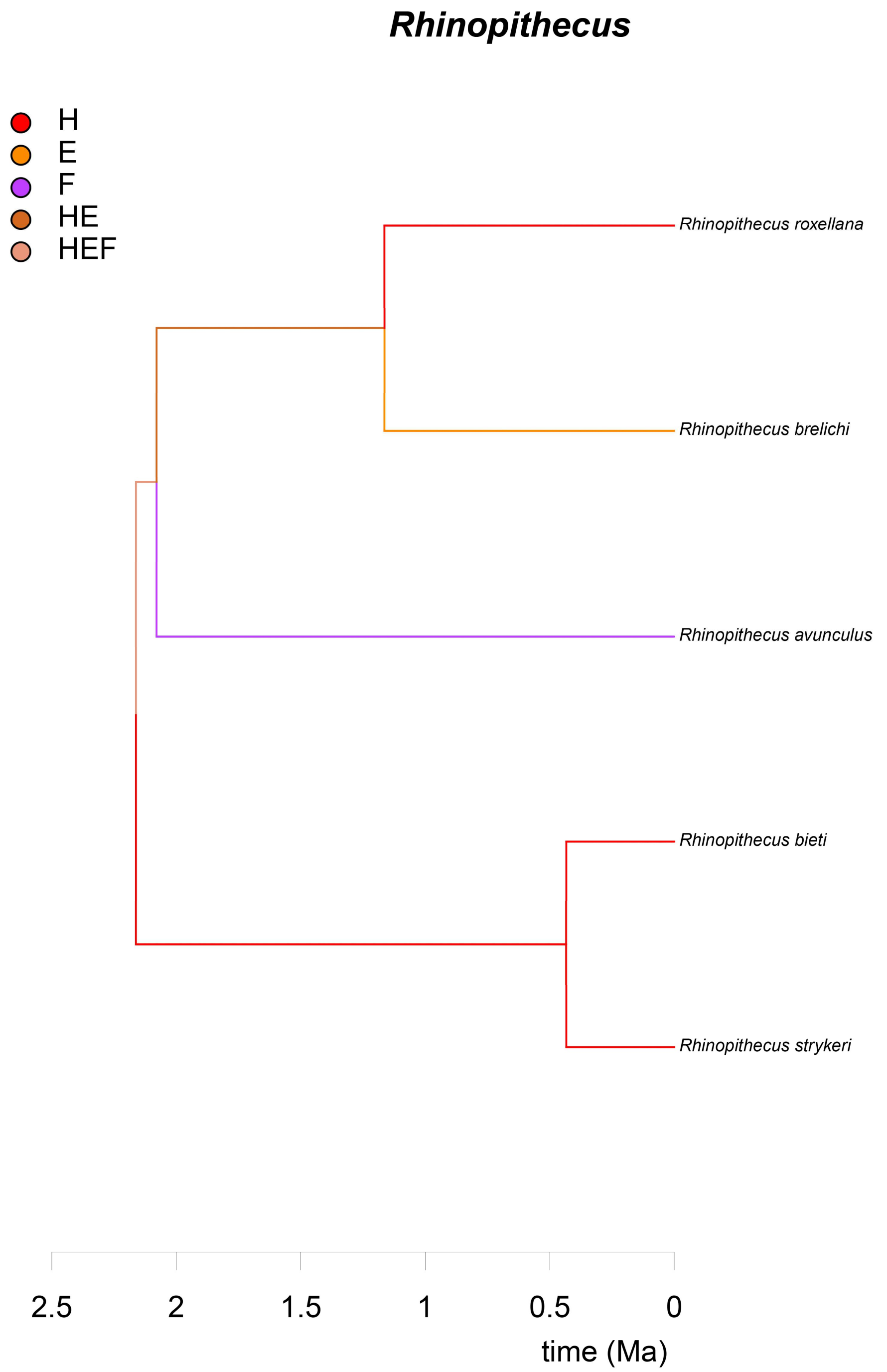

Fig. S37. Marginal maximum *a posteriori* reconstruction of the evolutionary history of geographic range on the maximum clade credibility tree of *Rhinopithecus* using RevBayes. Labels for geographic regions follow Fig. S2.

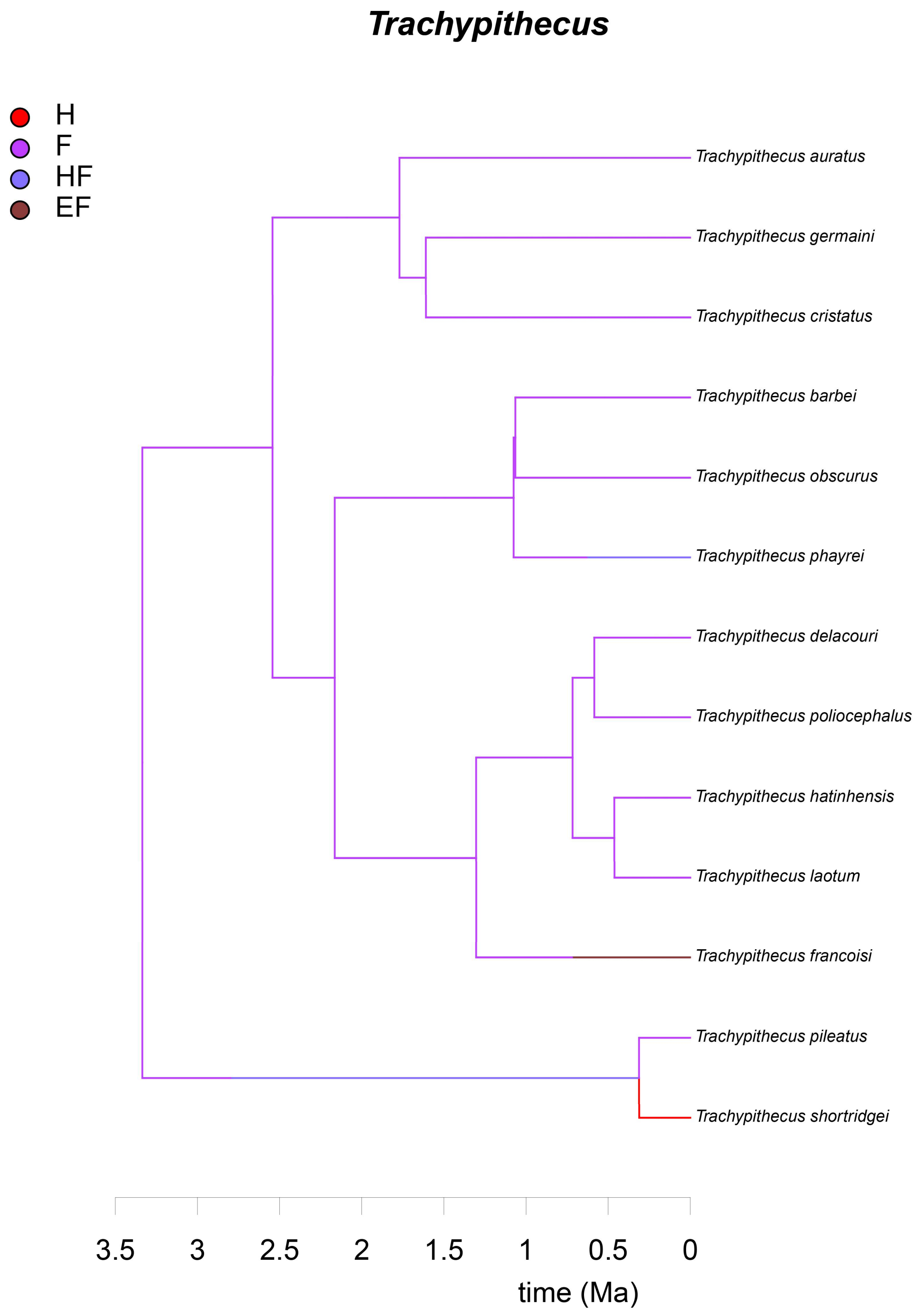

Fig. S38. Marginal maximum *a posteriori* reconstruction of the evolutionary history of geographic range on the maximum clade credibility tree of *Trachypithecus* using RevBayes. Labels for geographic regions follow Fig. S2.

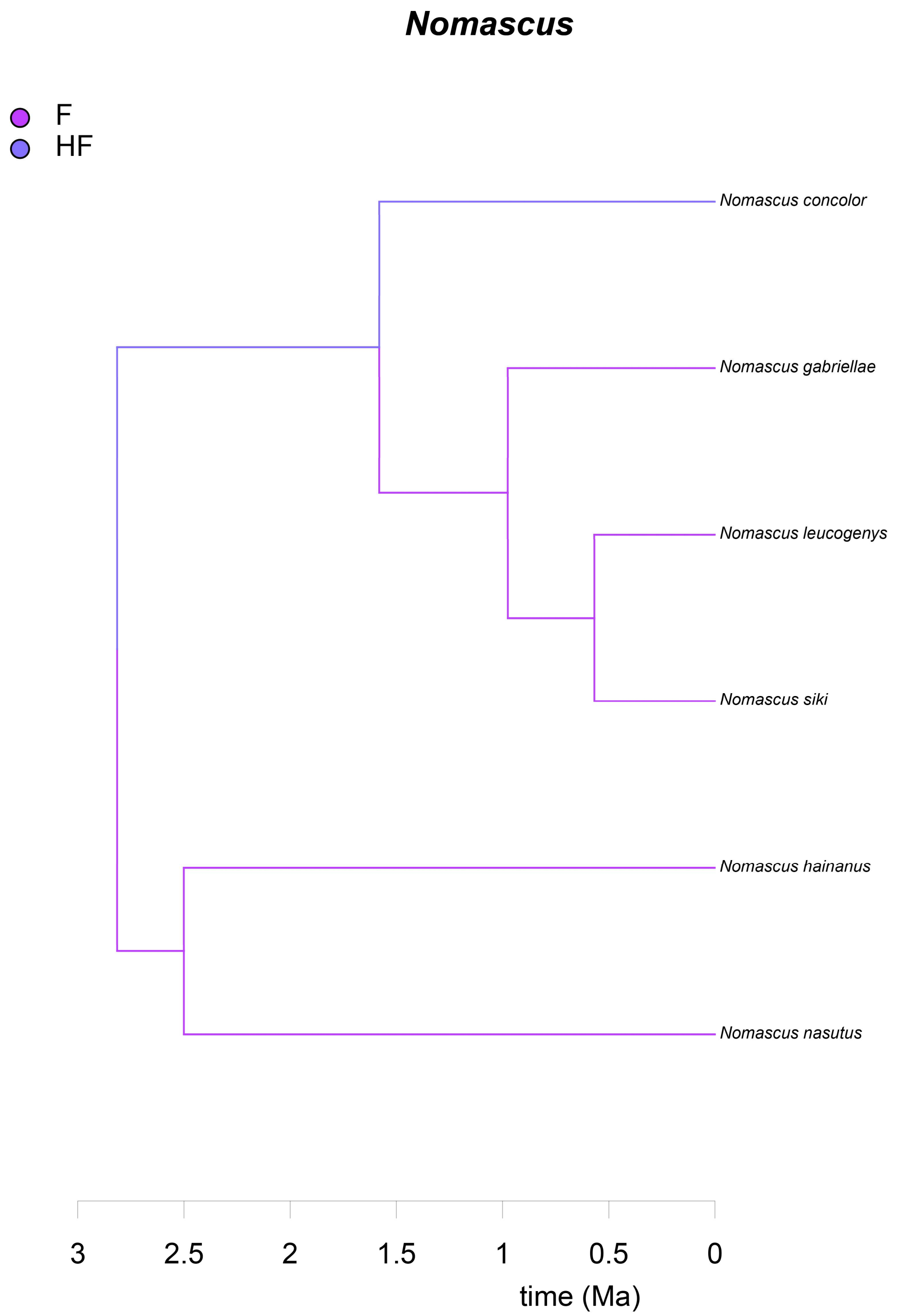

Fig. S39. Marginal maximum *a posteriori* reconstruction of the evolutionary history of geographic range on the maximum clade credibility tree of *Nomascus* using RevBayes. Labels for geographic regions follow Fig. S2.

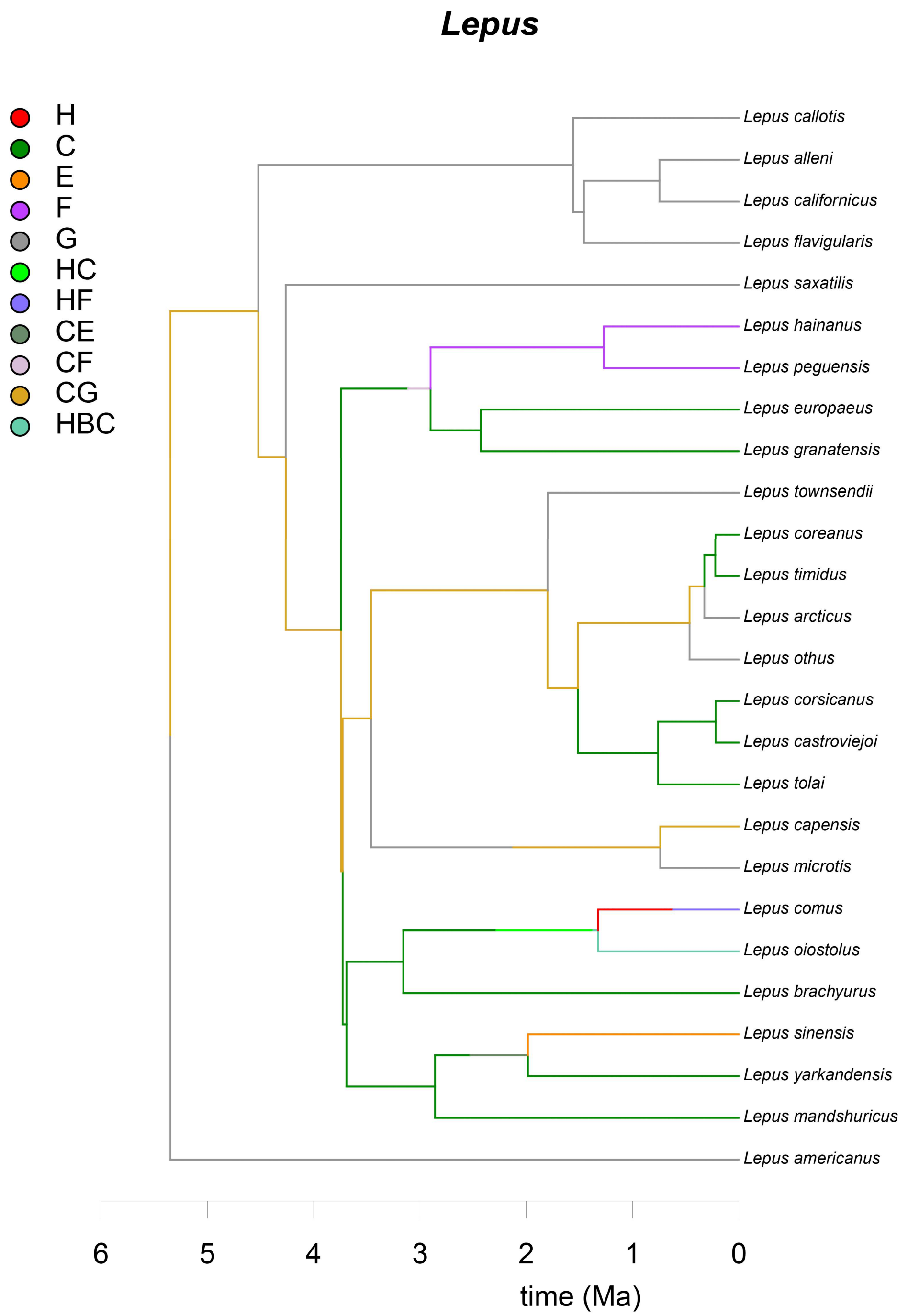

Fig. S40. Marginal maximum *a posteriori* reconstruction of the evolutionary history of geographic range on the maximum clade credibility tree of *Lepus* using RevBayes. Labels for geographic regions follow Fig. S2.

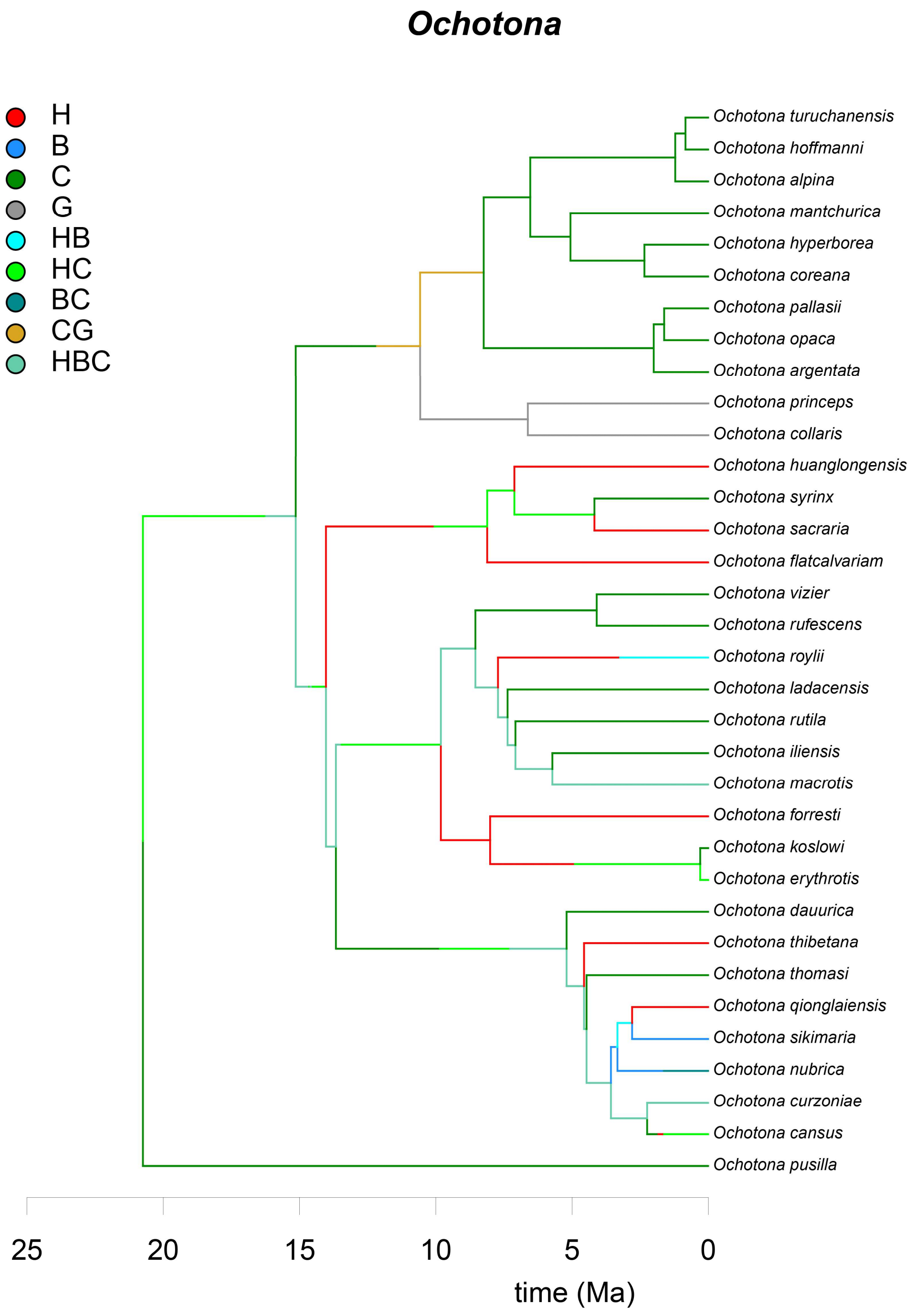

Fig. S41. Marginal maximum *a posteriori* reconstruction of the evolutionary history of geographic range on the maximum clade credibility tree of *Ochotona* using RevBayes. Labels for geographic regions follow Fig. S2.

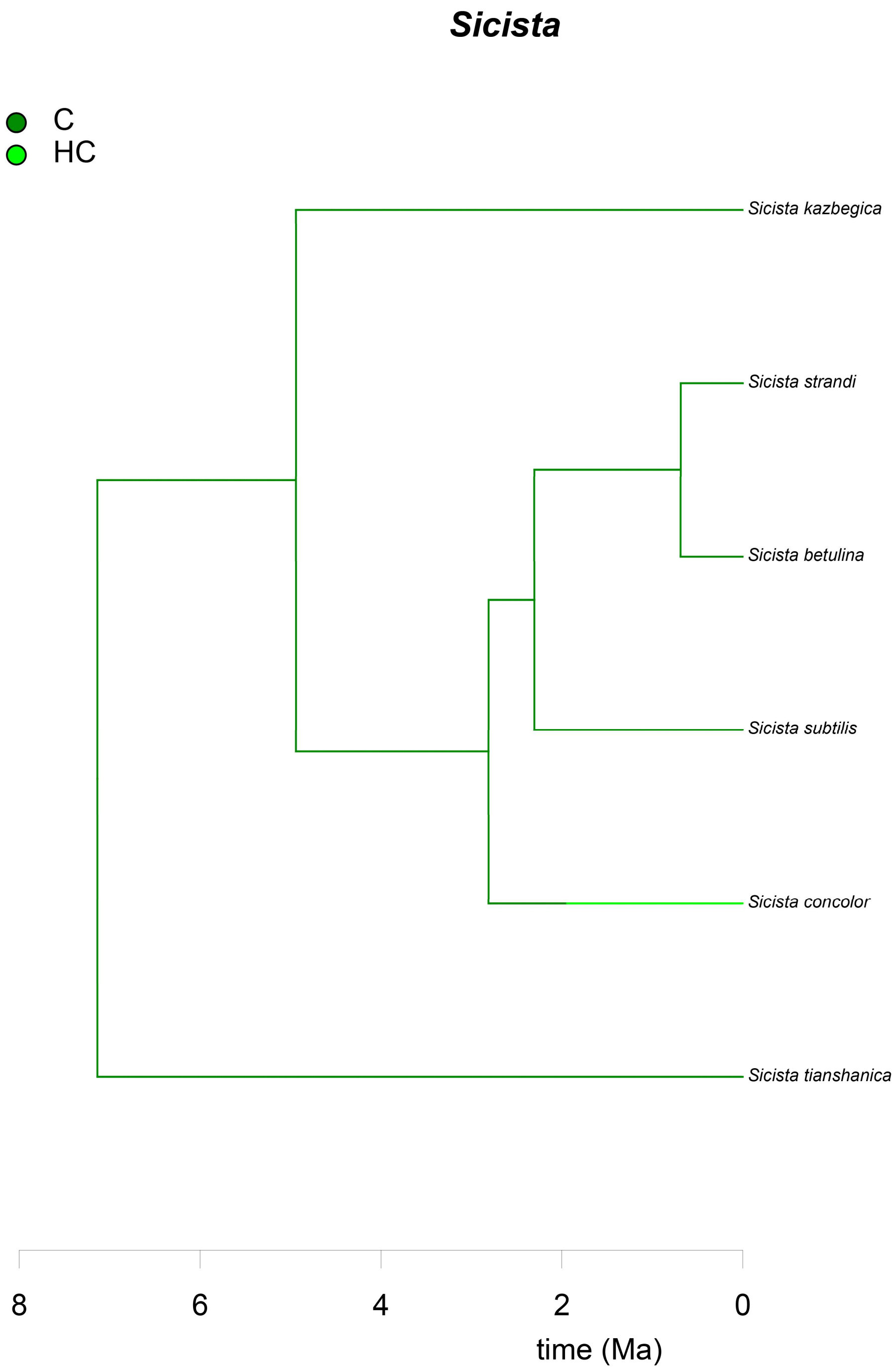

Fig. S42. Marginal maximum *a posteriori* reconstruction of the evolutionary history of geographic range on the maximum clade credibility tree of *Sicista* using RevBayes. Labels for geographic regions follow Fig. S2.

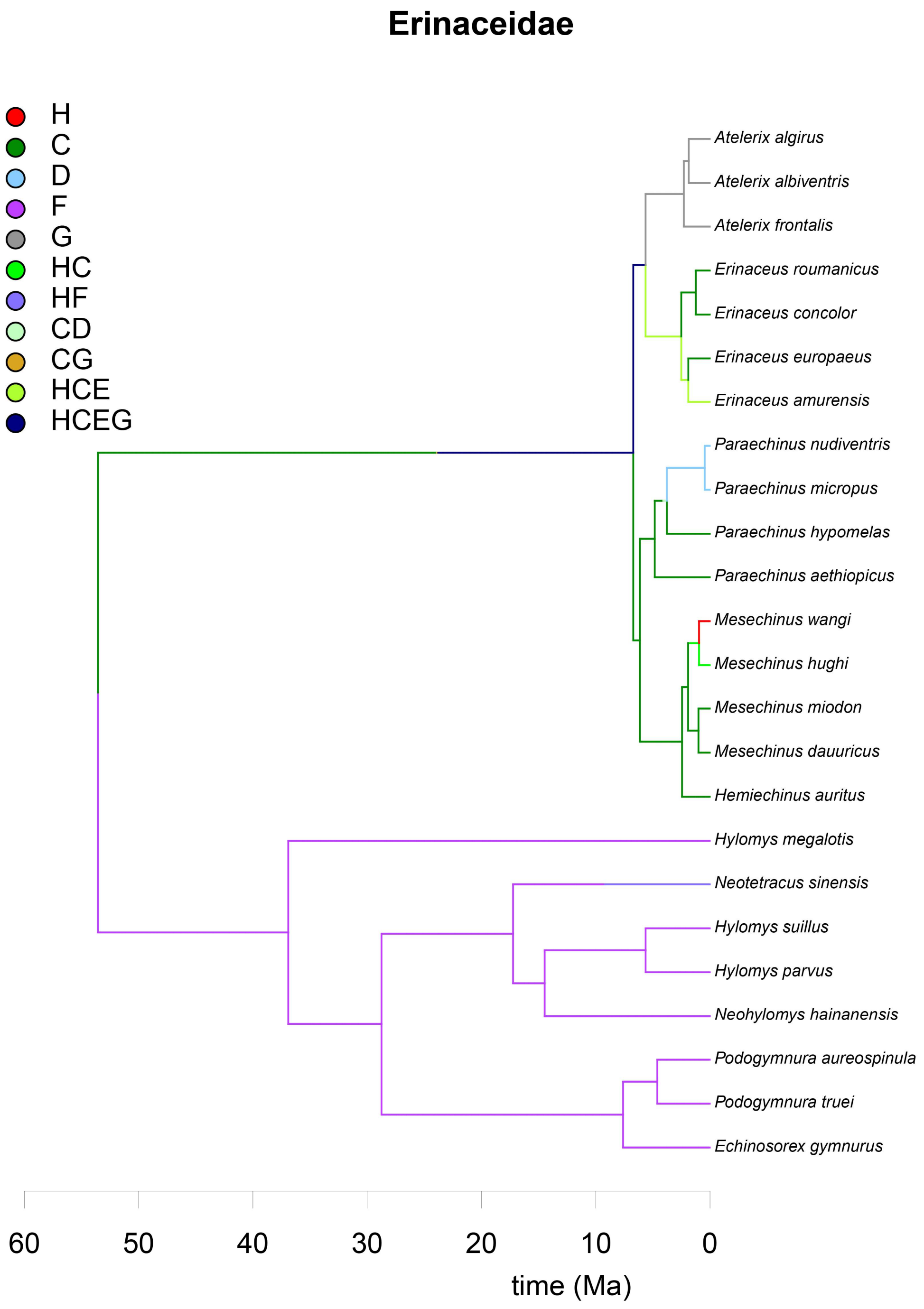

Fig. S43. Marginal maximum *a posteriori* reconstruction of the evolutionary history of geographic range on the maximum clade credibility tree of Erinaceidae using RevBayes. Labels for geographic regions follow Fig. S2.

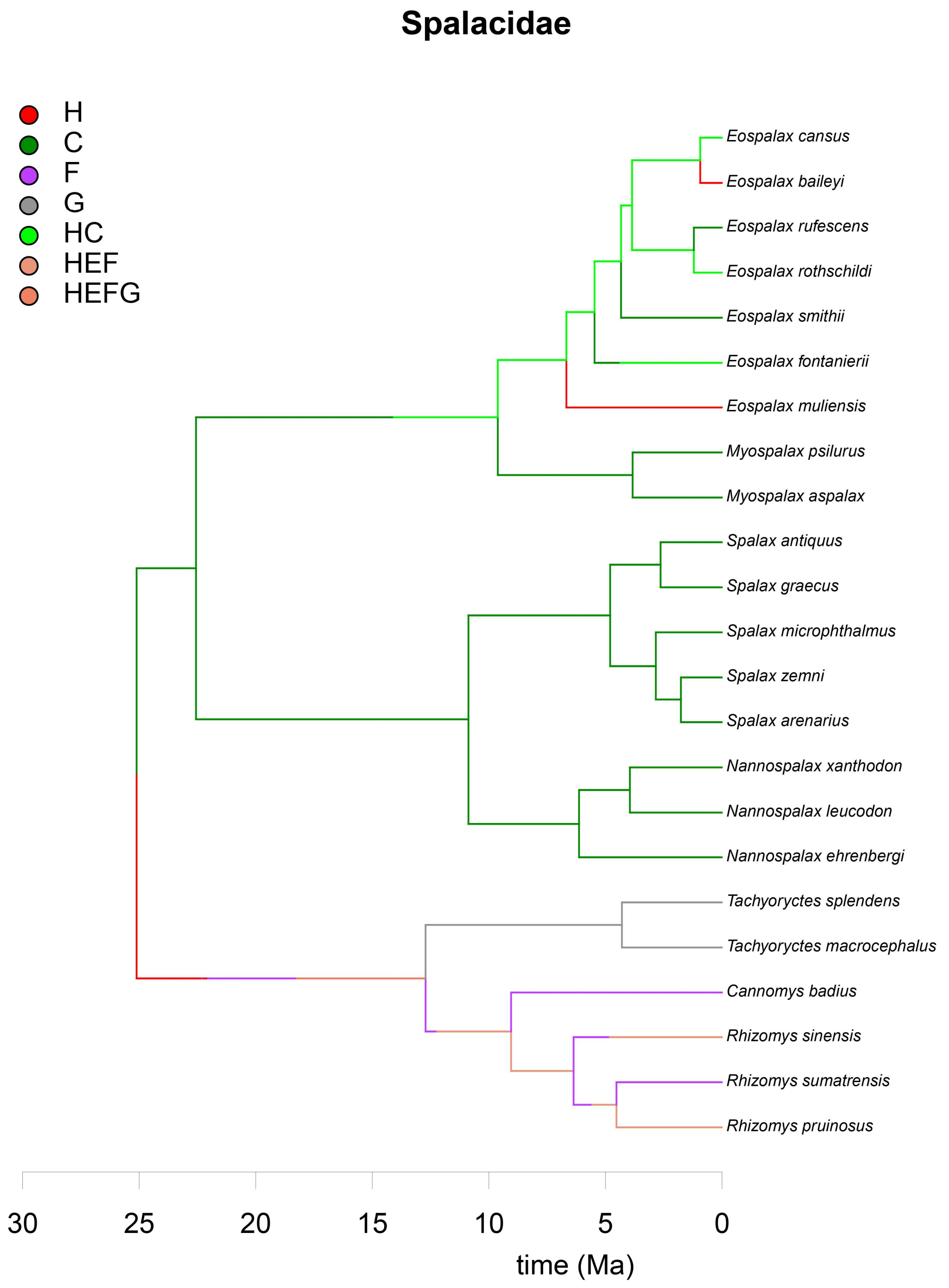

Fig. S44. Marginal maximum *a posteriori* reconstruction of the evolutionary history of geographic range on the maximum clade credibility tree of Spalacidae using RevBayes. Labels for geographic regions follow Fig. S2.

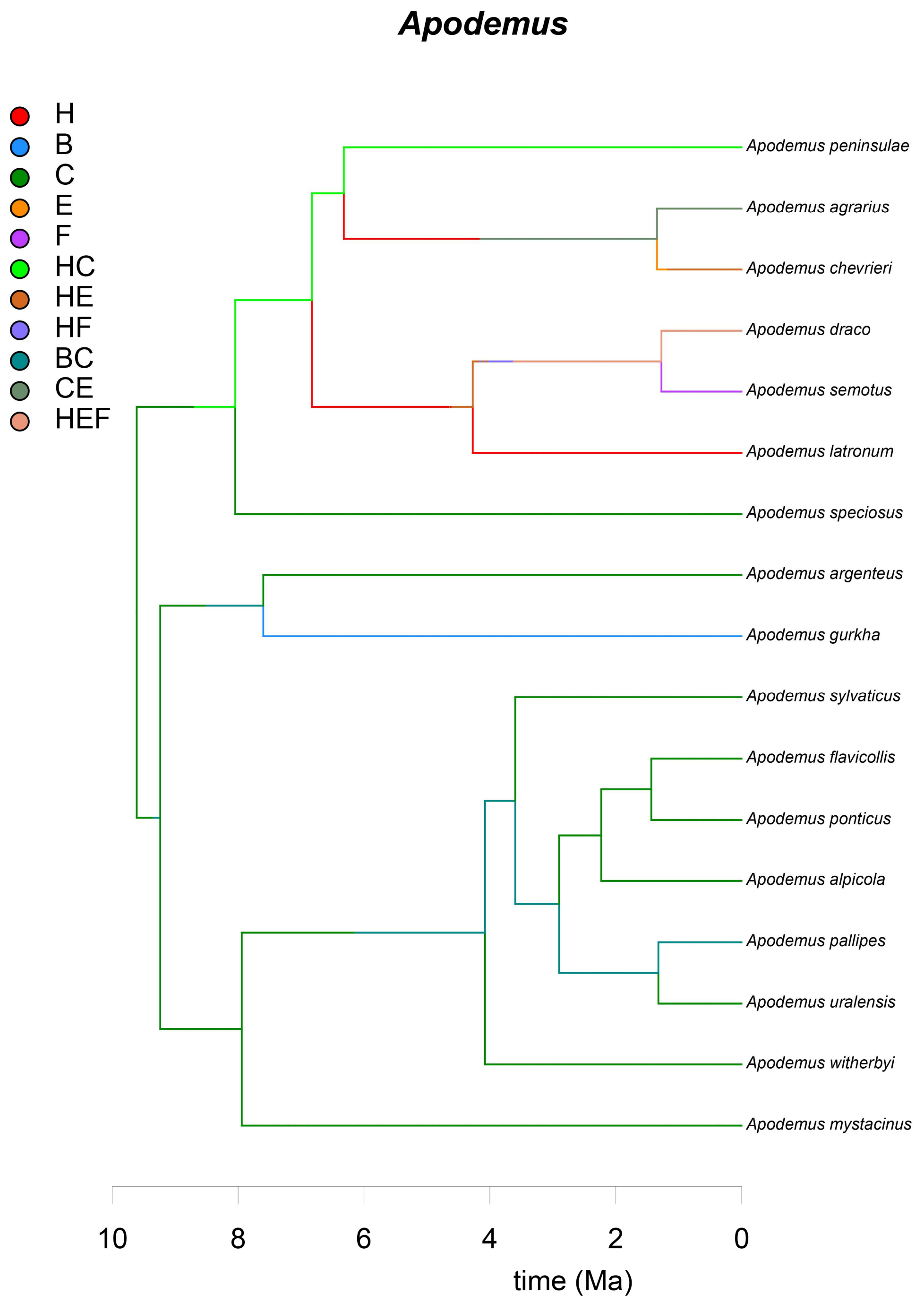

Fig. S45. Marginal maximum *a posteriori* reconstruction of the evolutionary history of geographic range on the maximum clade credibility tree of *Apodemus* using RevBayes. Labels for geographic regions follow Fig. S2.

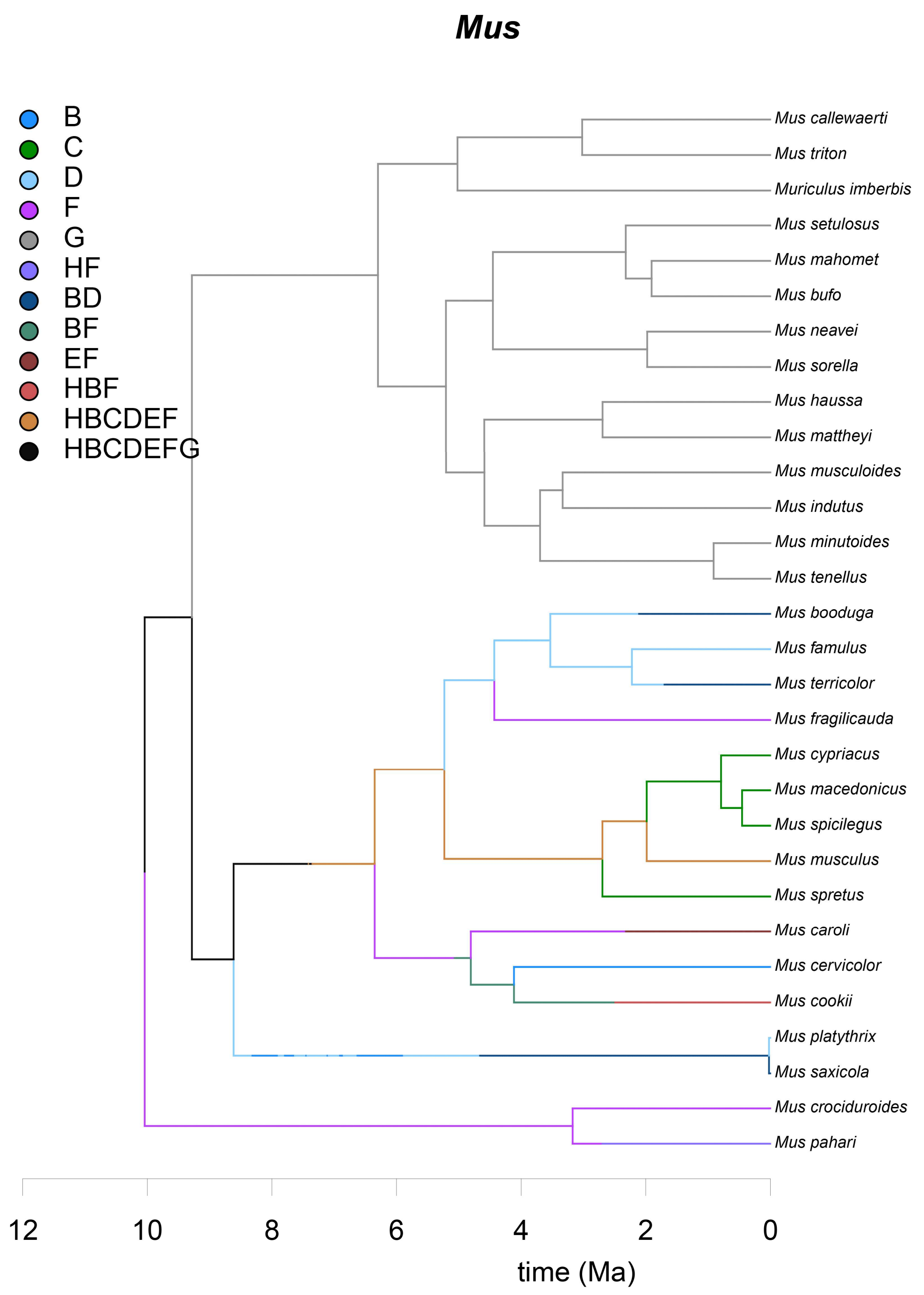

Fig. S46. Marginal maximum *a posteriori* reconstruction of the evolutionary history of geographic range on the maximum clade credibility tree of *Mus* using RevBayes. Labels for geographic regions follow Fig. S2.

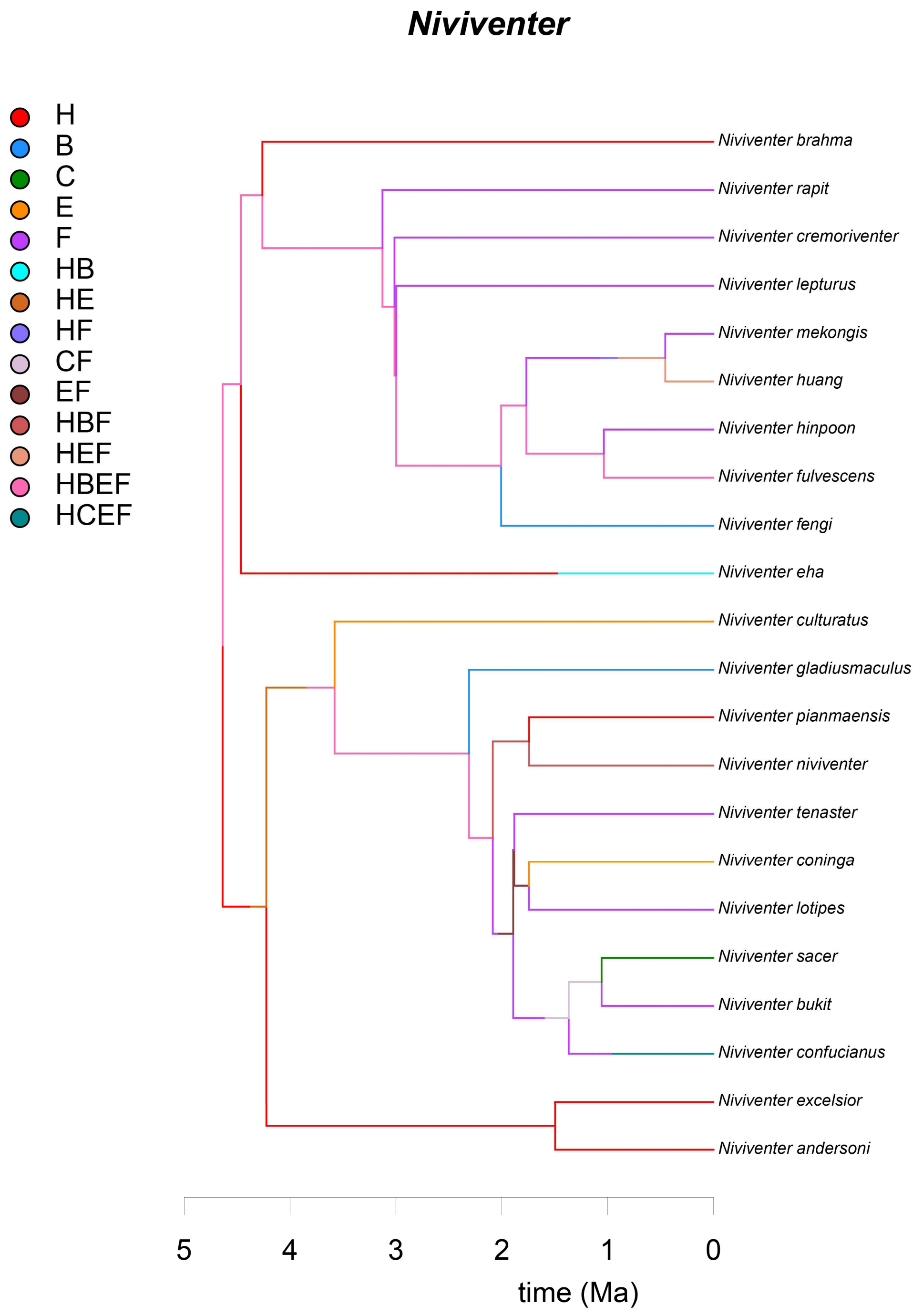

Fig. S47. Marginal maximum *a posteriori* reconstruction of the evolutionary history of geographic range on the maximum clade credibility tree of *Niviventer* using RevBayes. Labels for geographic regions follow Fig. S2.

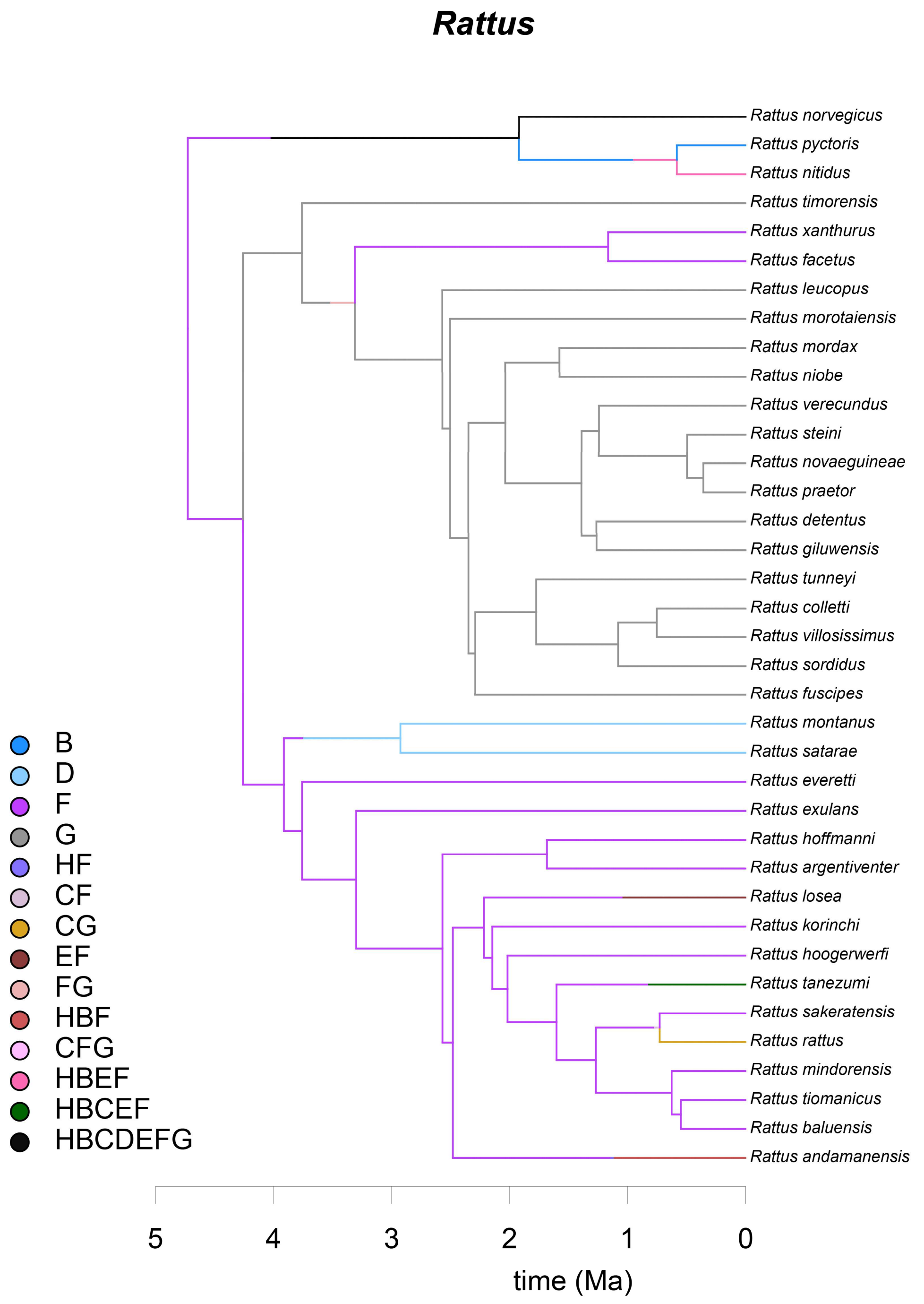

Fig. S48. Marginal maximum *a posteriori* reconstruction of the evolutionary history of geographic range on the maximum clade credibility tree of *Rattus* using RevBayes. Labels for geographic regions follow Fig. S2.

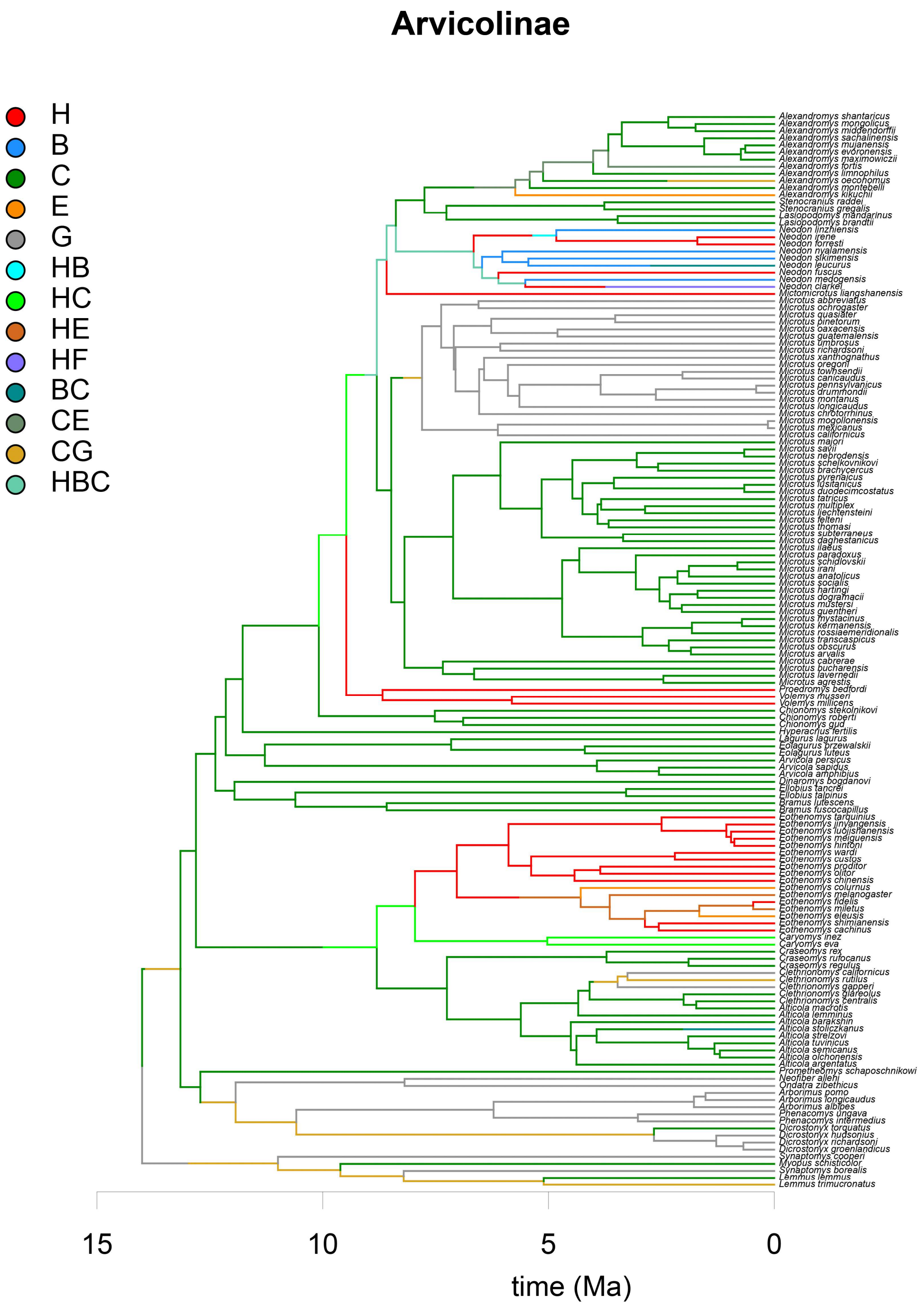

Fig. S49. Marginal maximum *a posteriori* reconstruction of the evolutionary history of geographic range on the maximum clade credibility tree of Arvicolinae using RevBayes. Labels for geographic regions follow Fig. S2.

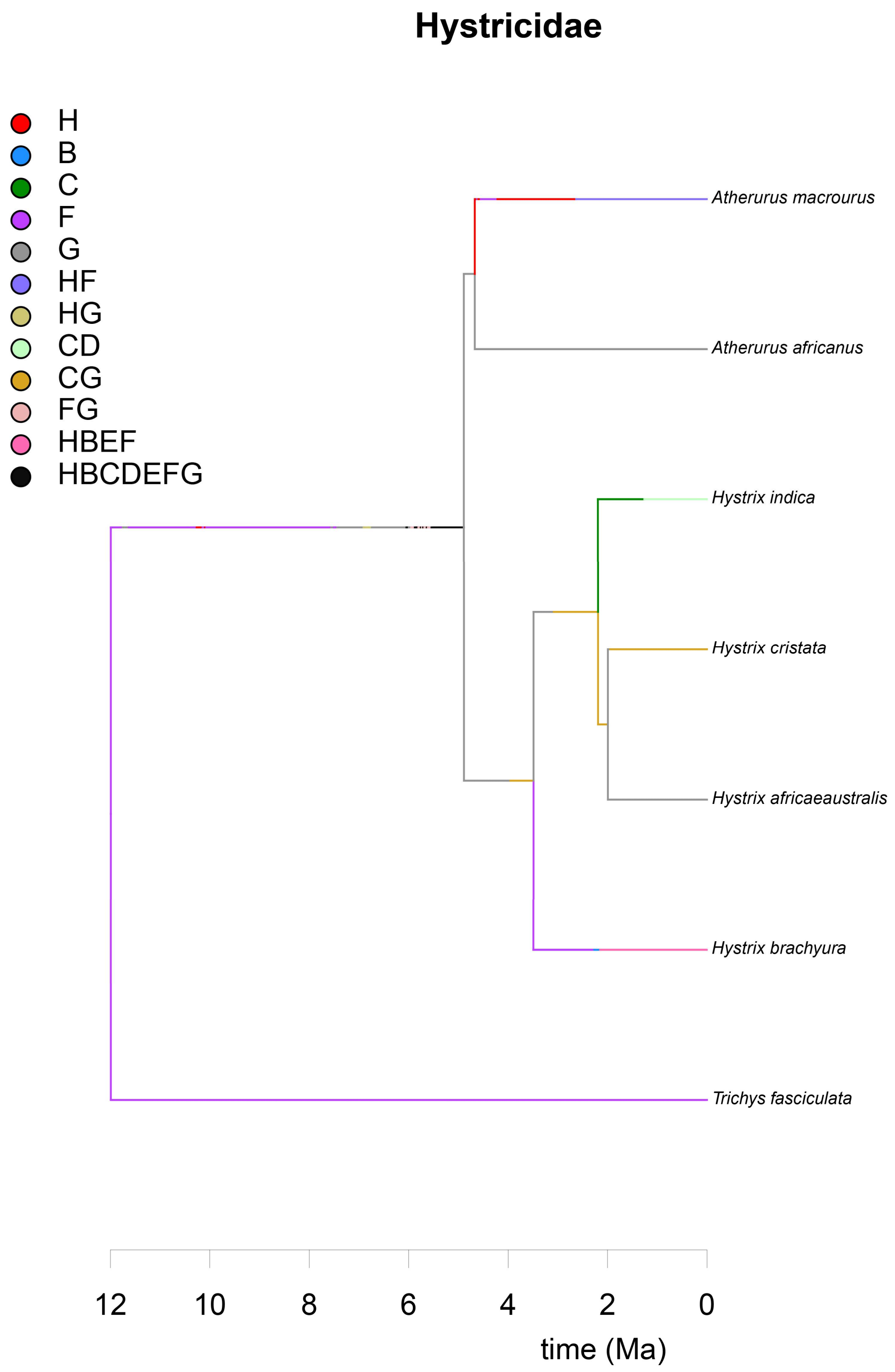

Fig. S50. Marginal maximum *a posteriori* reconstruction of the evolutionary history of geographic range on the maximum clade credibility tree of Hystricidae using RevBayes. Labels for geographic regions follow Fig. S2.

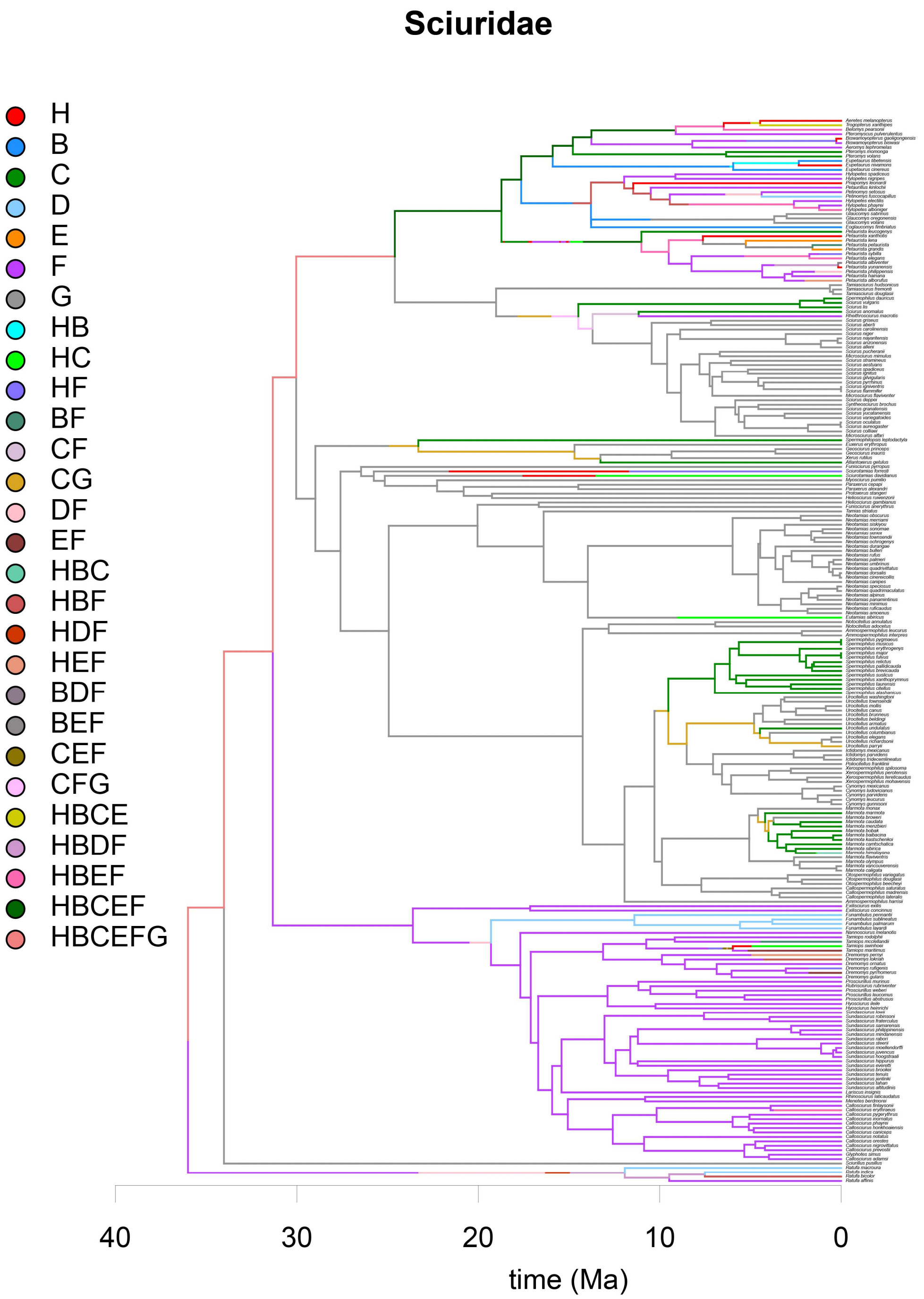

Fig. S51. Marginal maximum *a posteriori* reconstruction of the evolutionary history of geographic range on the maximum clade credibility tree of Sciuridae using RevBayes. Labels for geographic regions follow Fig. S2.

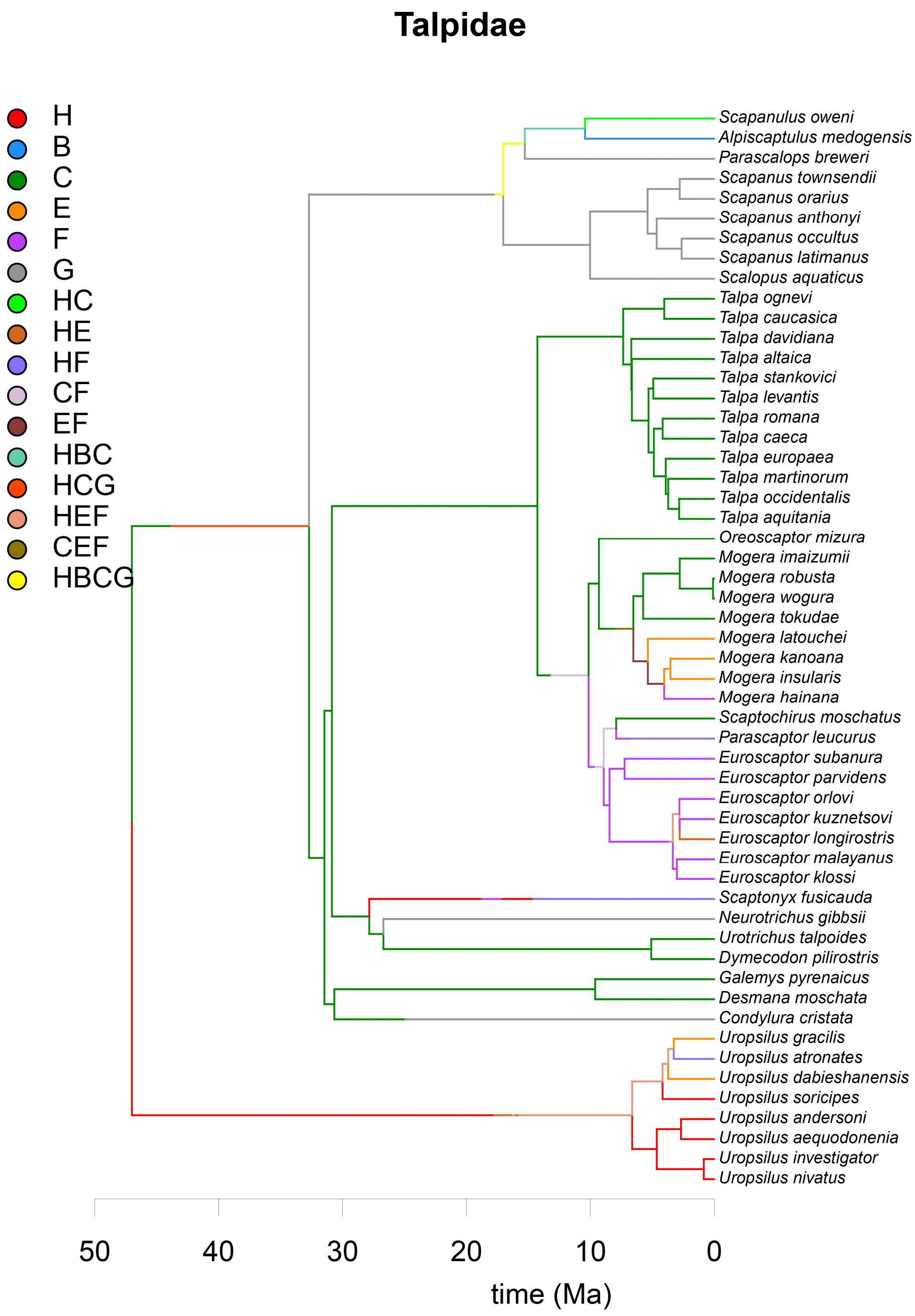

Fig. S52. Marginal maximum *a posteriori* reconstruction of the evolutionary history of geographic range on the maximum clade credibility tree of Talpidae using RevBayes. Labels for geographic regions follow Fig. S2.

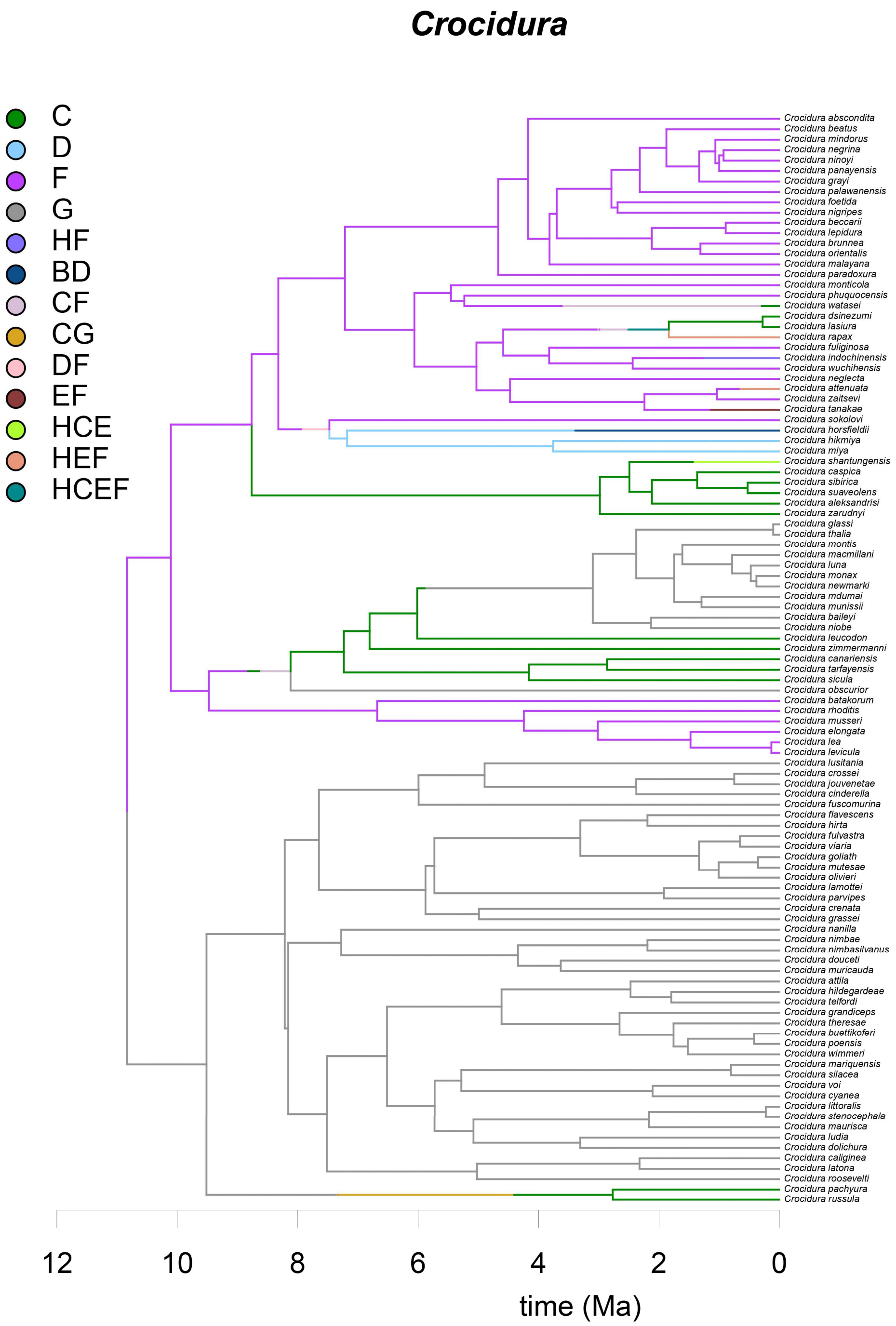

Fig. S53. Marginal maximum *a posteriori* reconstruction of the evolutionary history of geographic range on the maximum clade credibility tree of *Crocidura* using RevBayes. Labels for geographic regions follow Fig. S2.

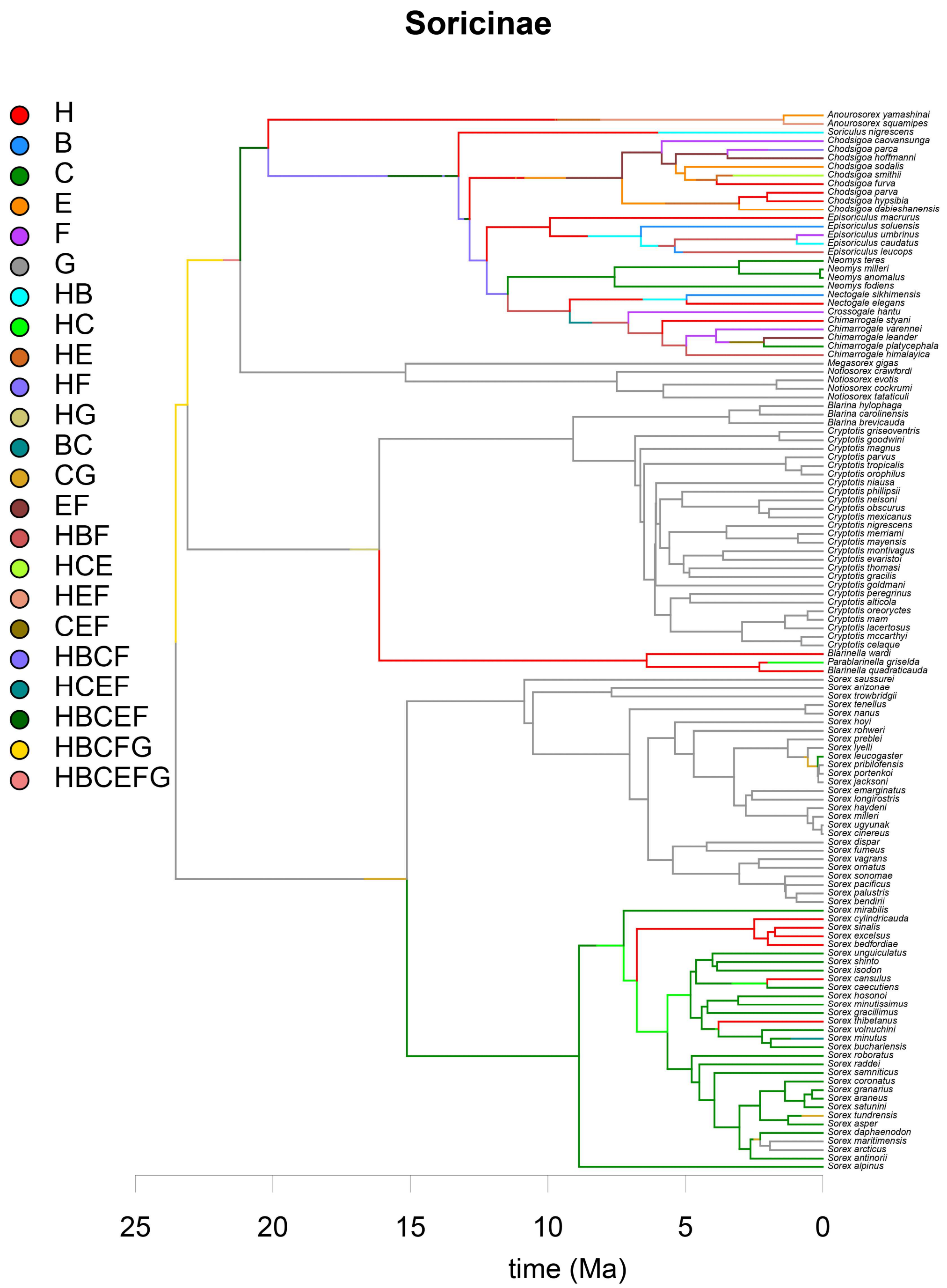

Fig. S54. Marginal maximum *a posteriori* reconstruction of the evolutionary history of geographic range on the maximum clade credibility tree of Soricinae using RevBayes. Labels for geographic regions follow Fig. S2.

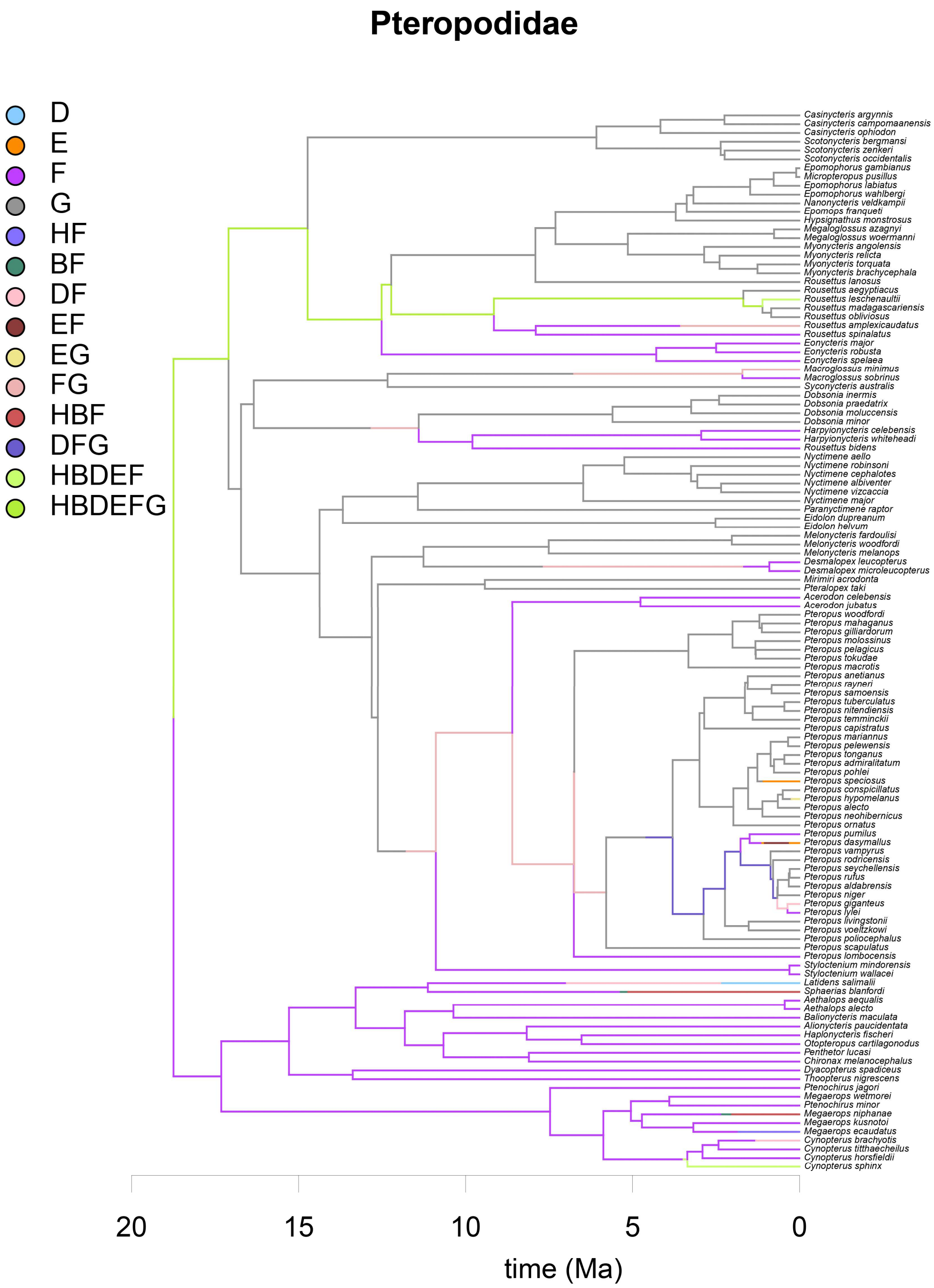

Fig. S55. Marginal maximum *a posteriori* reconstruction of the evolutionary history of geographic range on the maximum clade credibility tree of Pteropodidae using RevBayes. Labels for geographic regions follow Fig. S2.

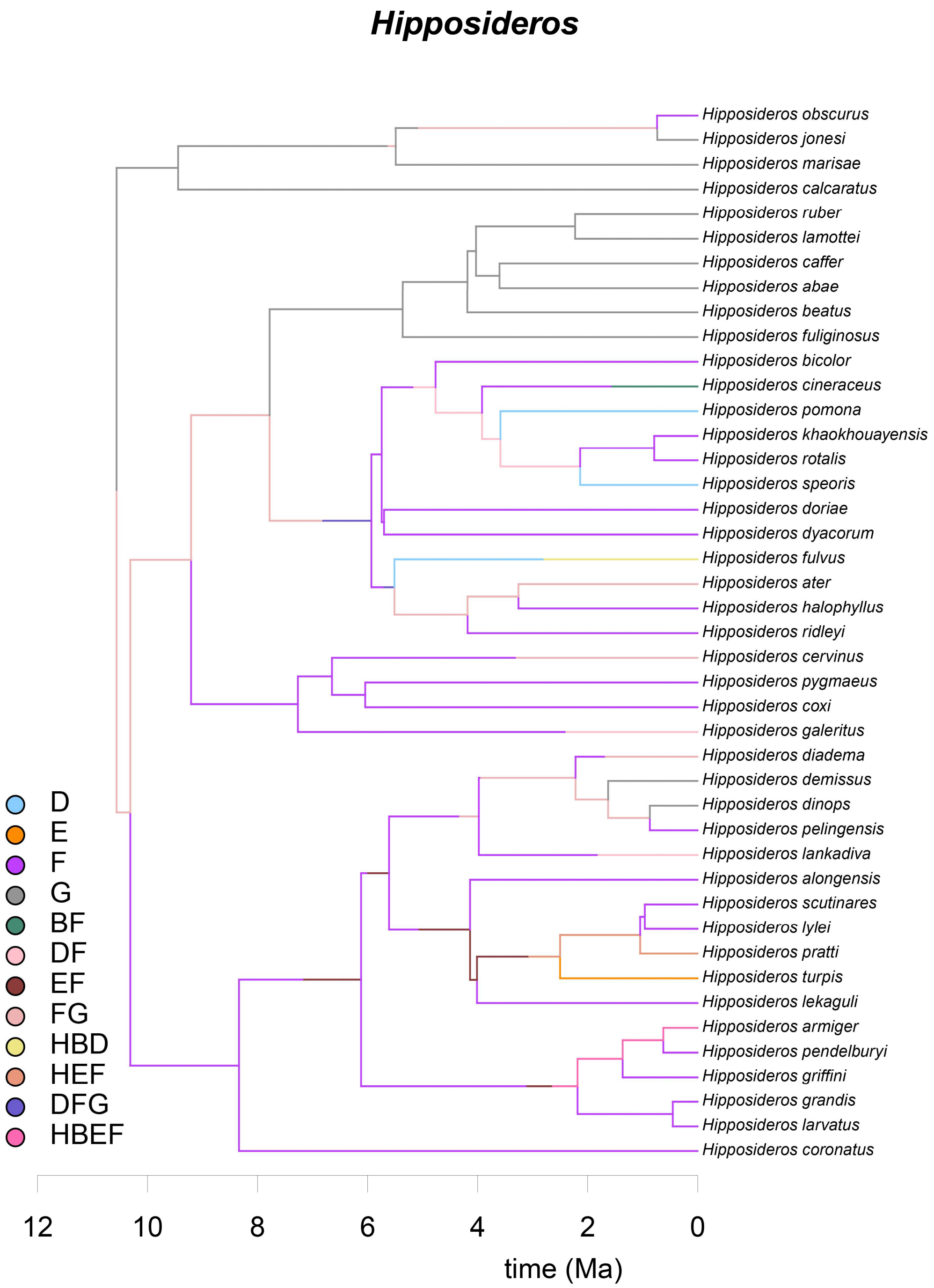

Fig. S56. Marginal maximum *a posteriori* reconstruction of the evolutionary history of geographic range on the maximum clade credibility tree of *Hipposideros* using RevBayes. Labels for geographic regions follow Fig. S2.

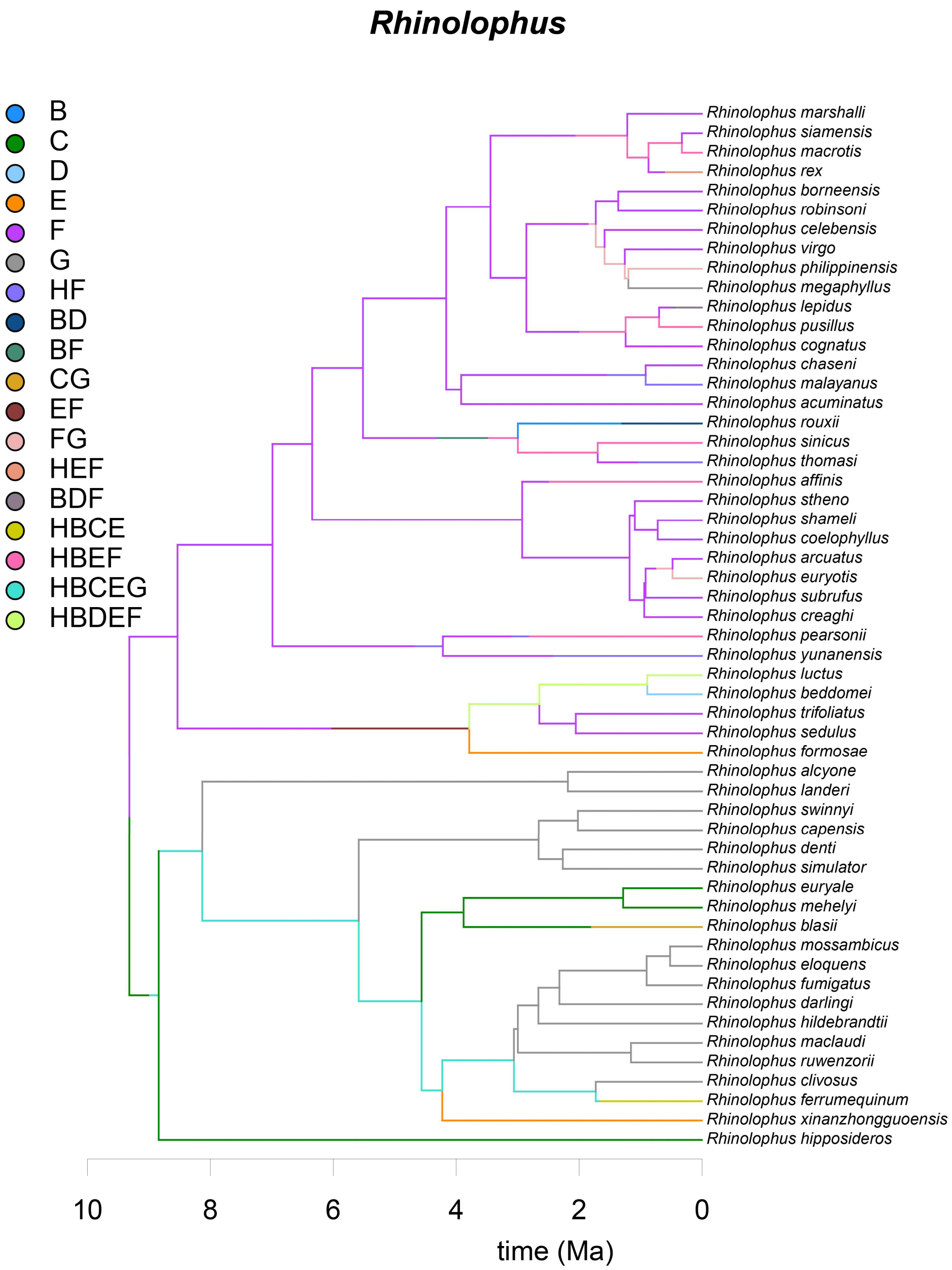

Fig. S57. Marginal maximum *a posteriori* reconstruction of the evolutionary history of geographic range on the maximum clade credibility tree of *Rhinolophus* using RevBayes. Labels for geographic regions follow Fig. S2.

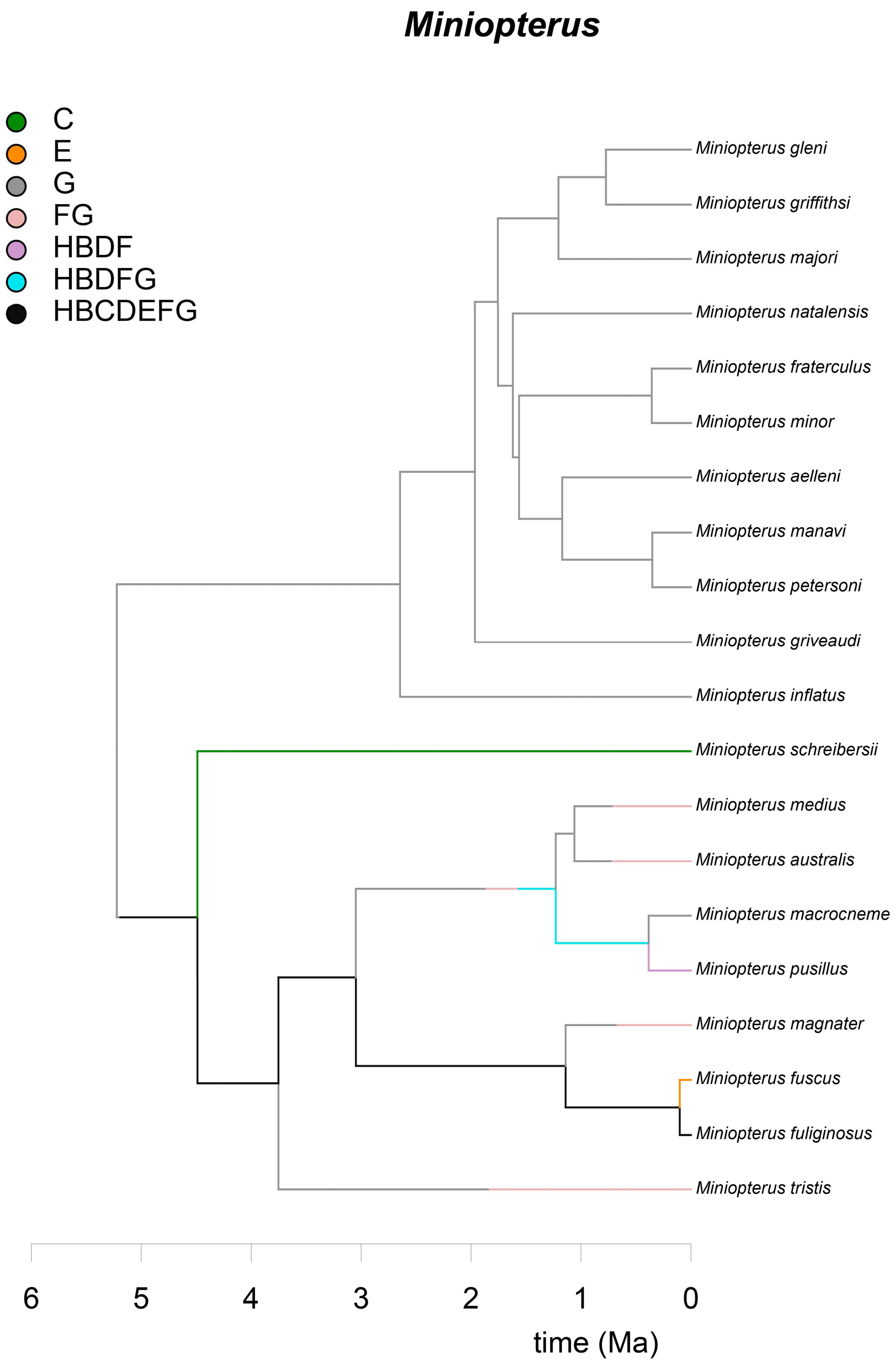

Fig. S58. Marginal maximum *a posteriori* reconstruction of the evolutionary history of geographic range on the maximum clade credibility tree of *Miniopterus* using RevBayes. Labels for geographic regions follow Fig. S2.

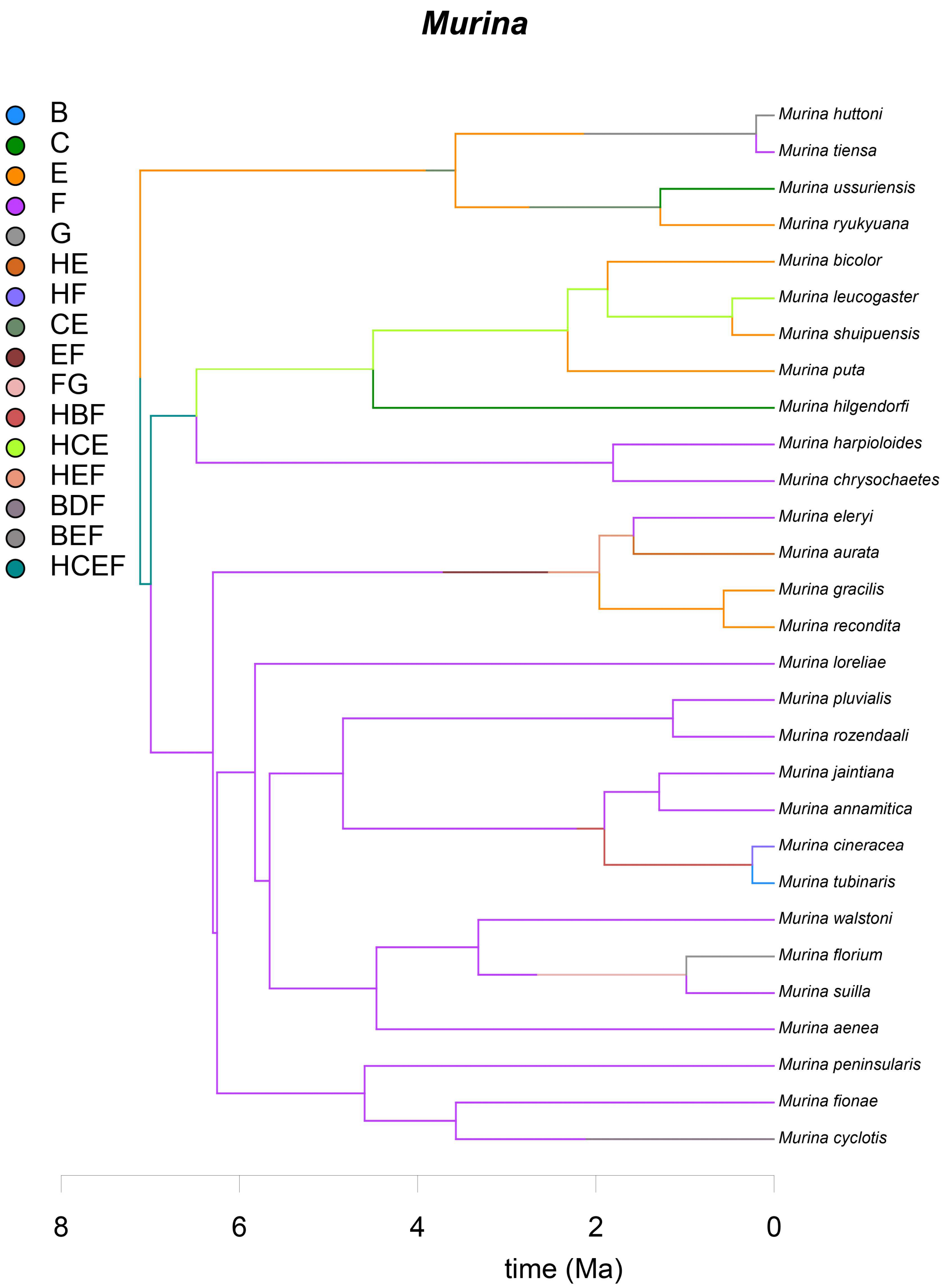

Fig. S59. Marginal maximum *a posteriori* reconstruction of the evolutionary history of geographic range on the maximum clade credibility tree of *Murina* using RevBayes. Labels for geographic regions follow Fig. S2.

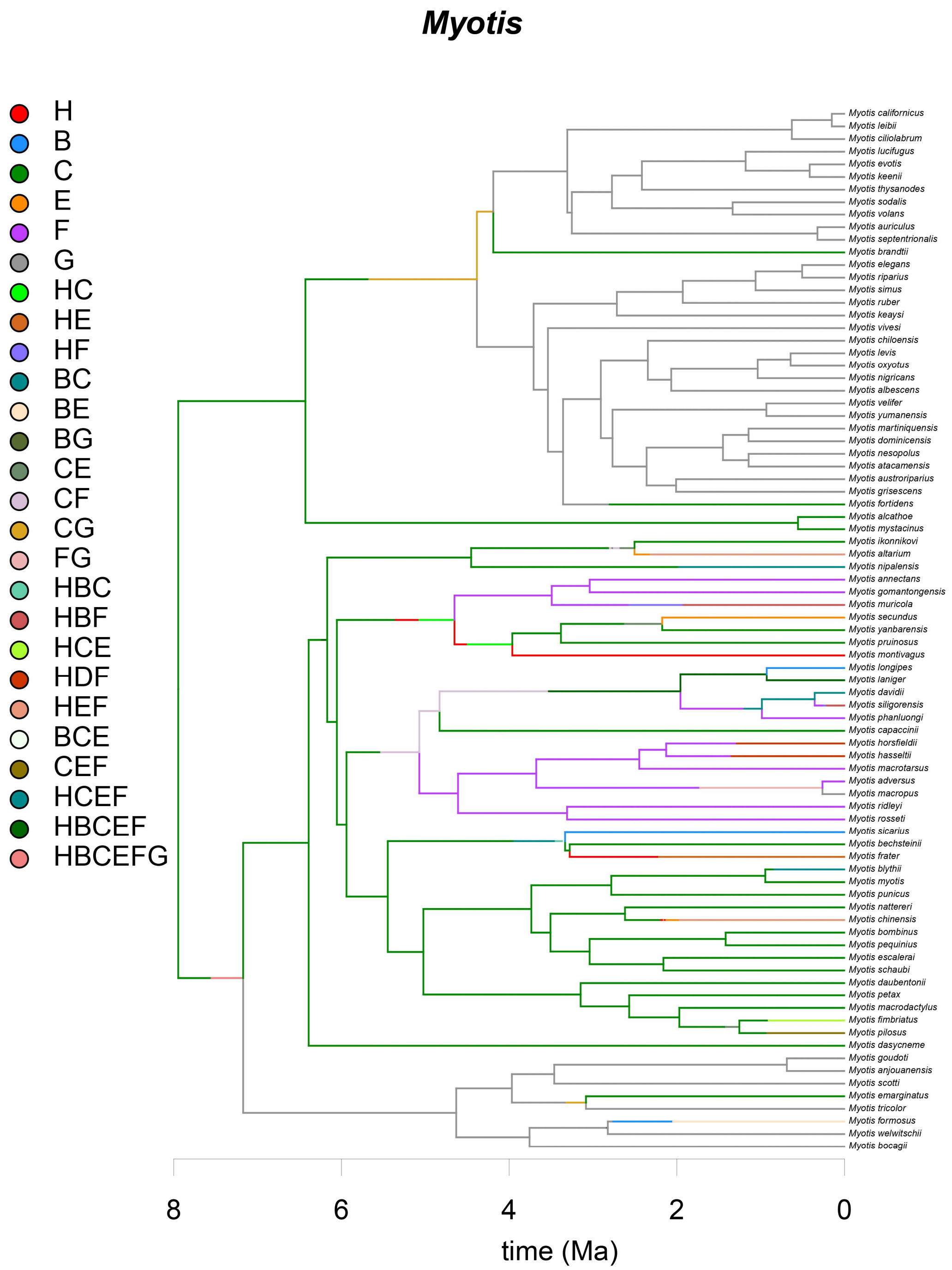

Fig. S60. Marginal maximum *a posteriori* reconstruction of the evolutionary history of geographic range on the maximum clade credibility tree of *Myotis* using RevBayes. Labels for geographic regions follow Fig. S2.

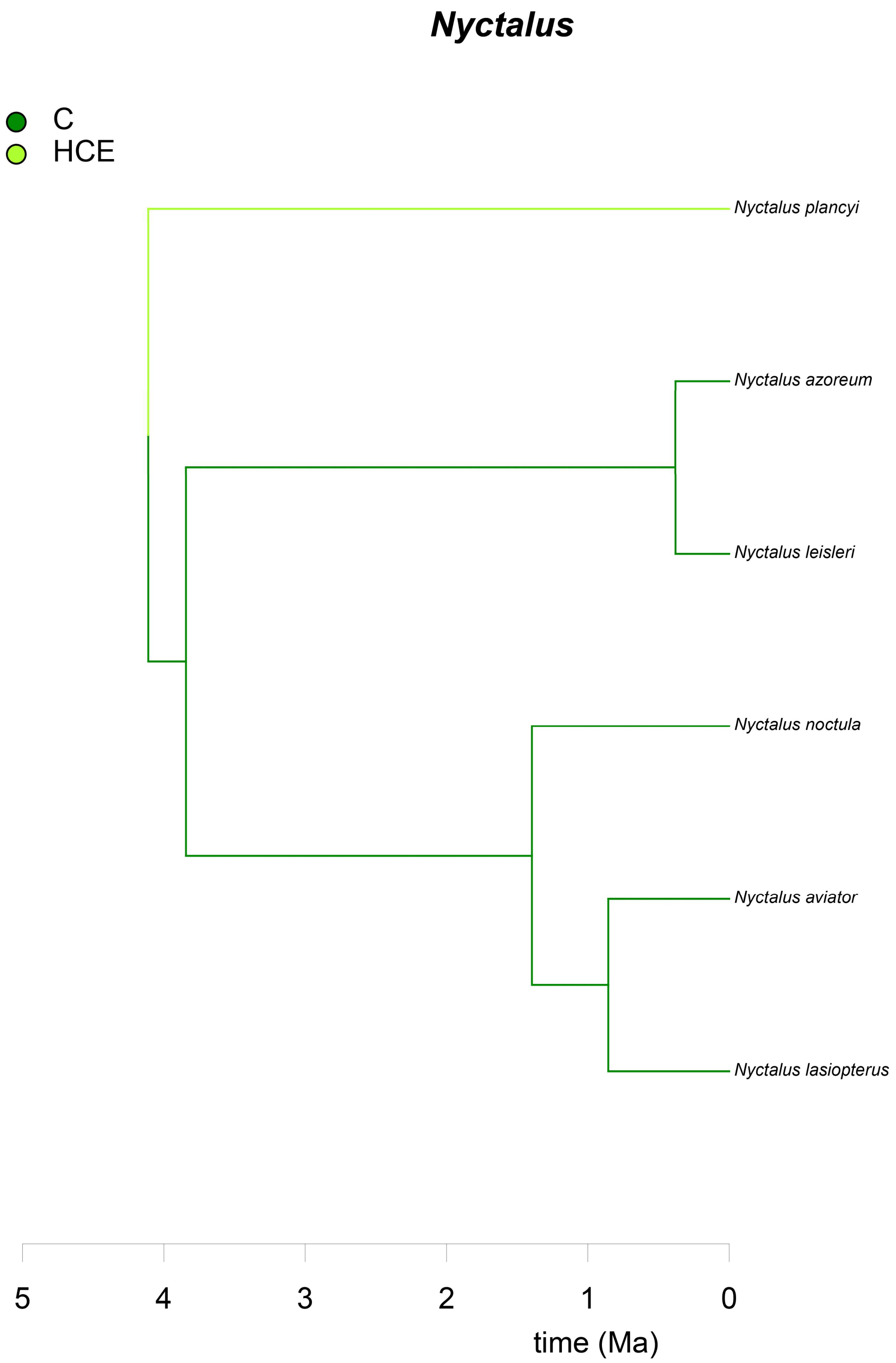

Fig. S61. Marginal maximum *a posteriori* reconstruction of the evolutionary history of geographic range on the maximum clade credibility tree of *Nyctalus* using RevBayes. Labels for geographic regions follow Fig. S2.

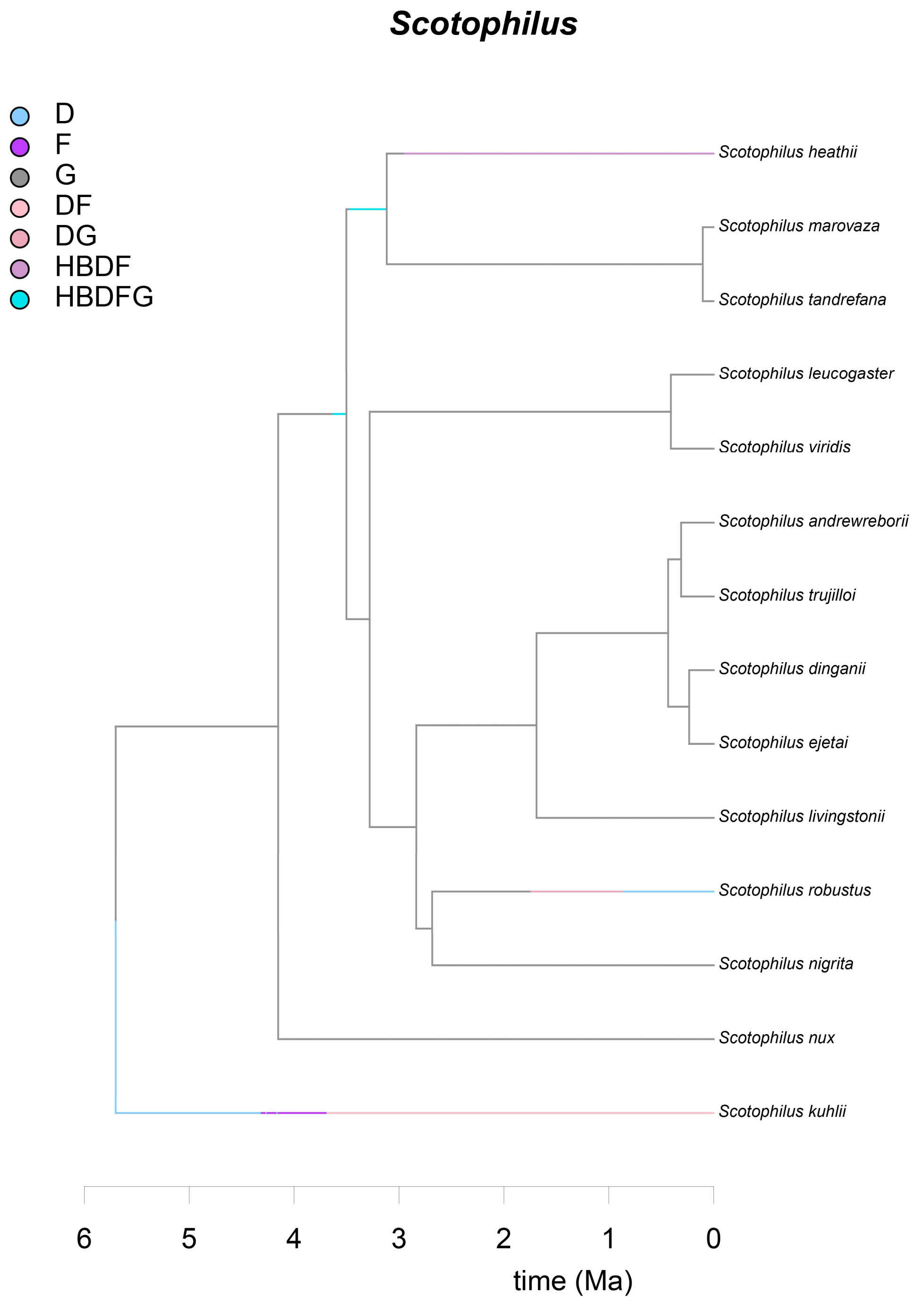

Fig. S62. Marginal maximum *a posteriori* reconstruction of the evolutionary history of geographic range on the maximum clade credibility tree of *Scotophilus* using RevBayes. Labels for geographic regions follow Fig. S2.

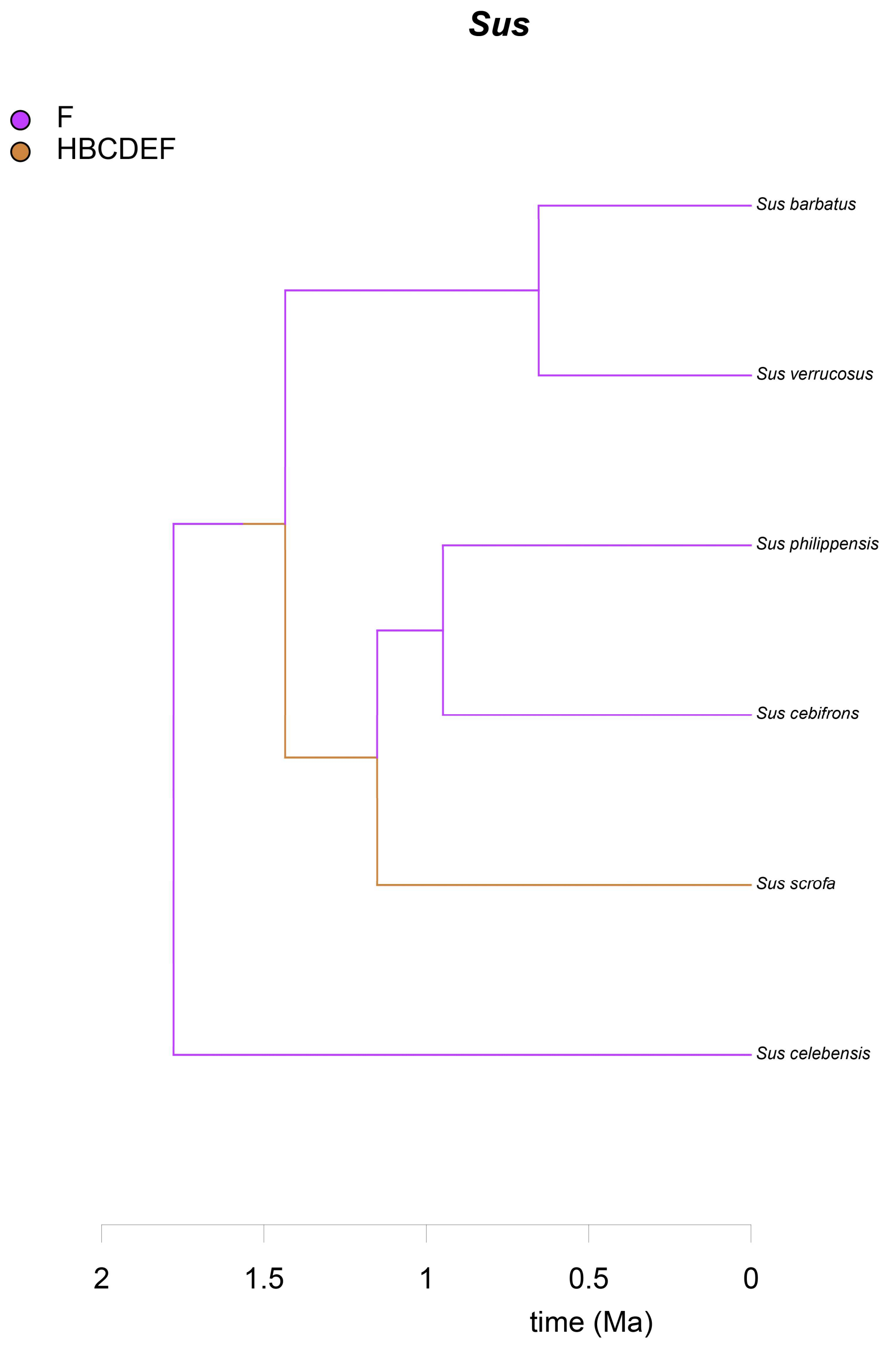

Fig. S63. Marginal maximum *a posteriori* reconstruction of the evolutionary history of geographic range on the maximum clade credibility tree of *Sus* using RevBayes. Labels for geographic regions follow Fig. S2.

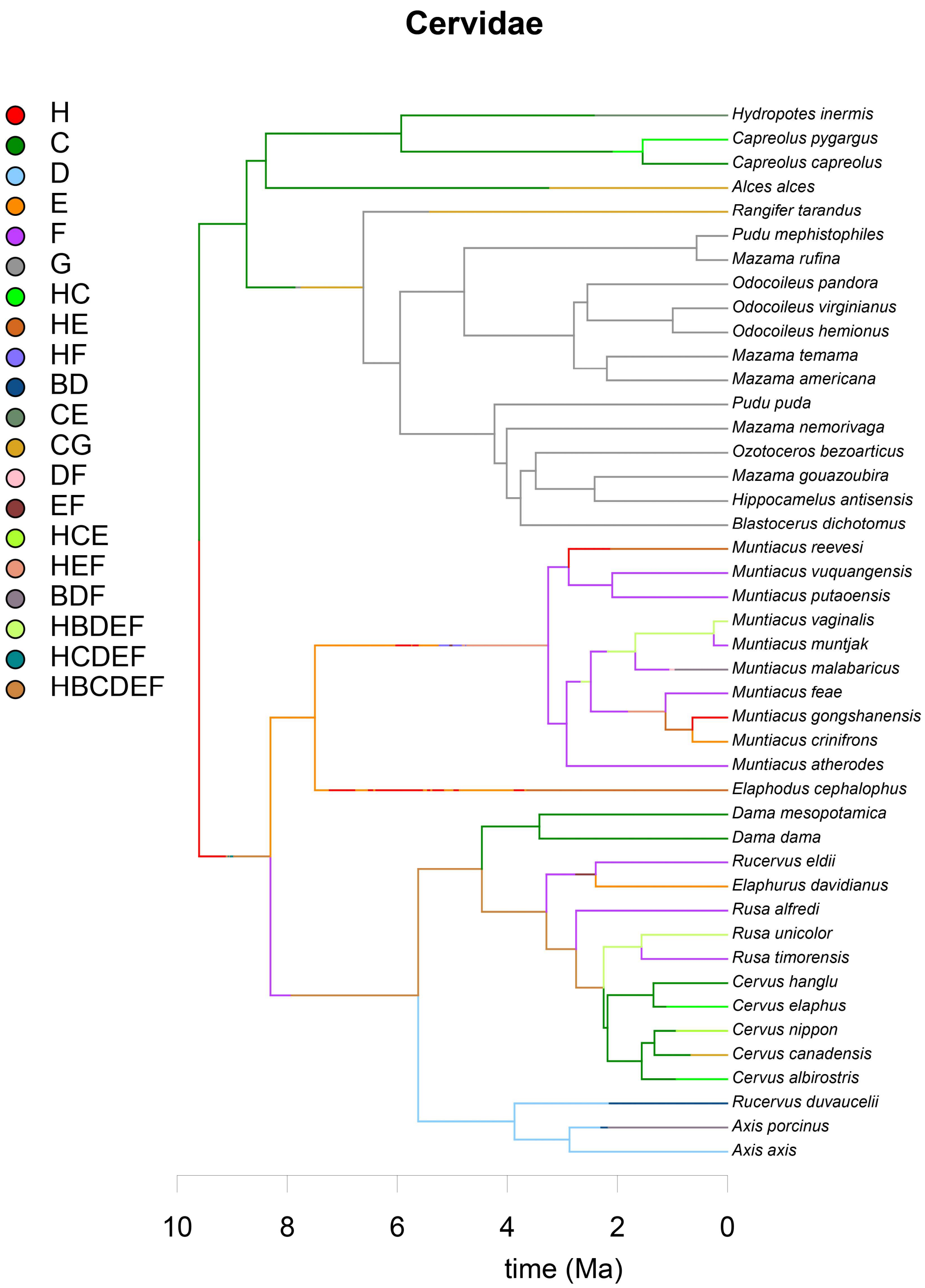

Fig. S64. Marginal maximum *a posteriori* reconstruction of the evolutionary history of geographic range on the maximum clade credibility tree of Cervidae using RevBayes. Labels for geographic regions follow Fig. S2.

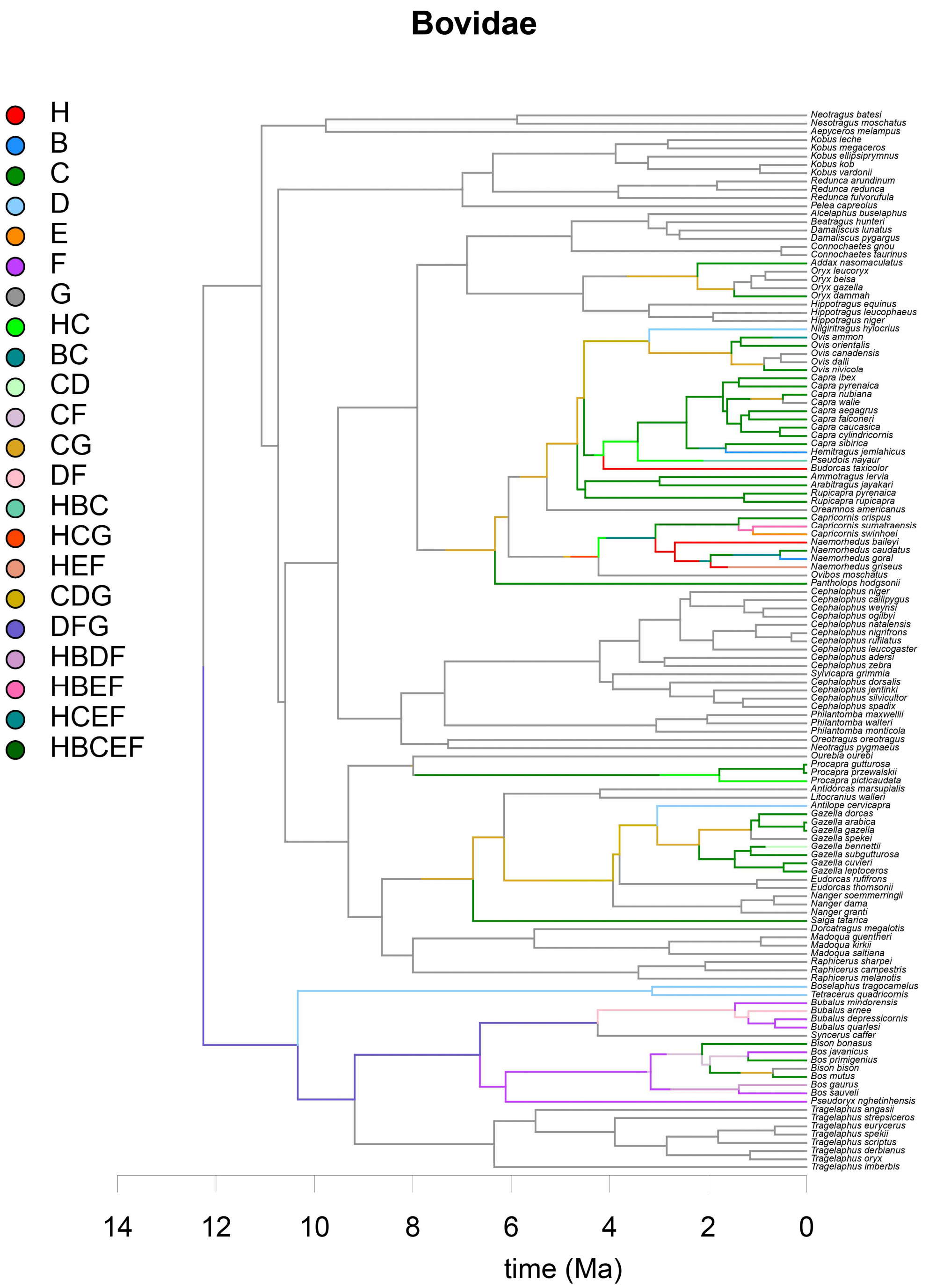

Fig. S65. Marginal maximum *a posteriori* reconstruction of the evolutionary history of geographic range on the maximum clade credibility tree of Bovidae using RevBayes. Labels for geographic regions follow Fig. S2.

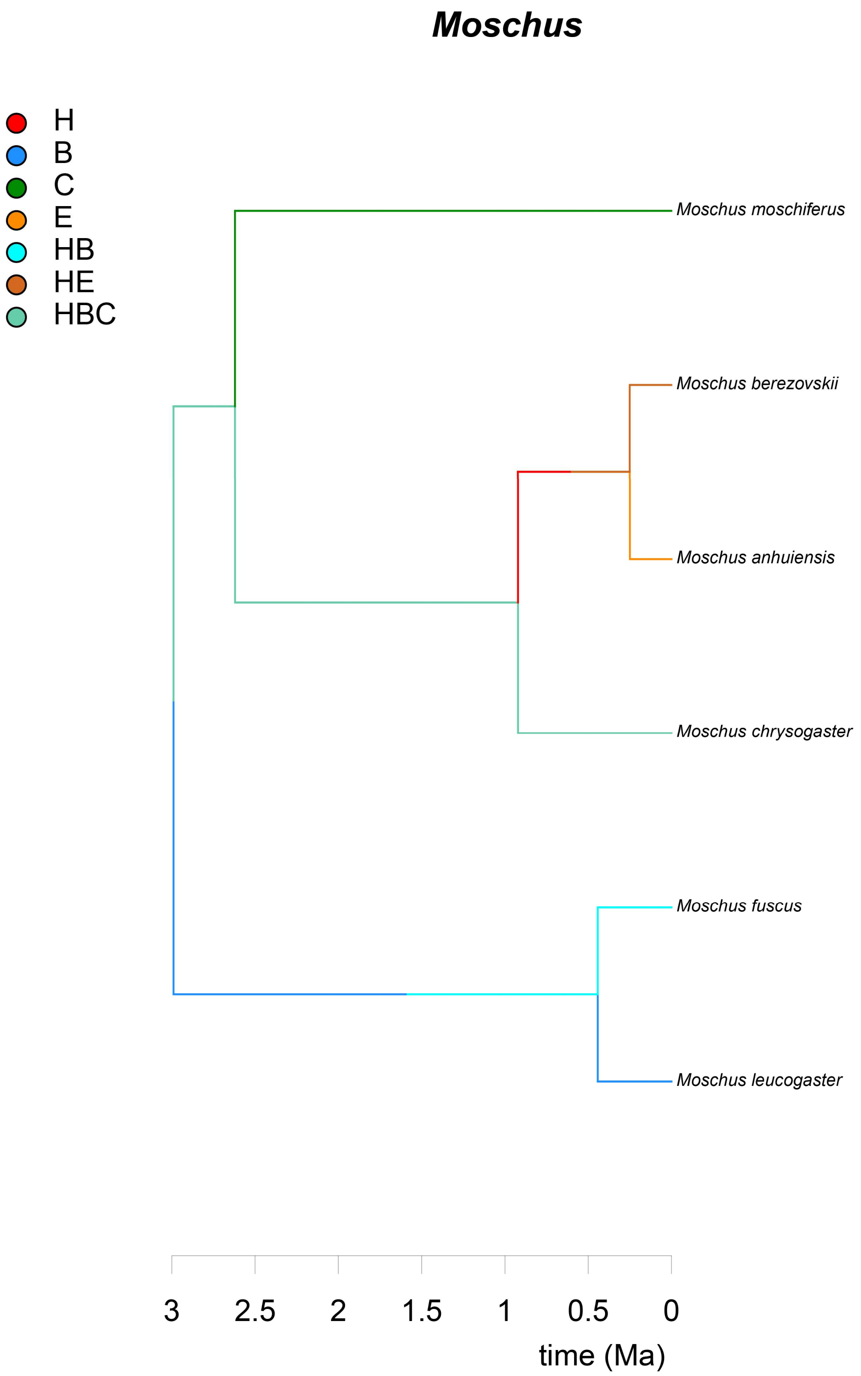

Fig. S66. Marginal maximum *a posteriori* reconstruction of the evolutionary history of geographic range on the maximum clade credibility tree of *Moschus* using RevBayes. Labels for geographic regions follow Fig. S2.

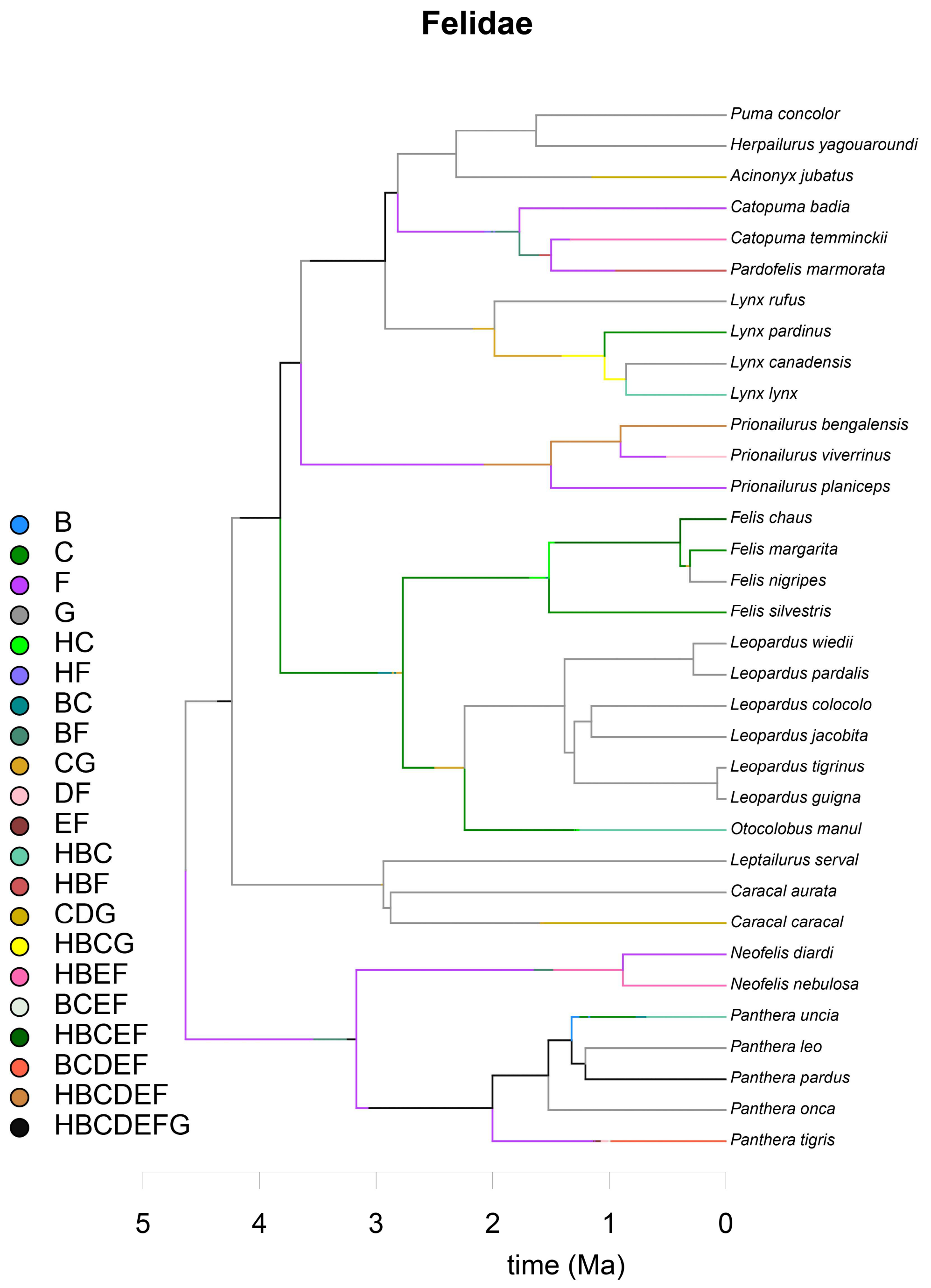

Fig. S67. Marginal maximum *a posteriori* reconstruction of the evolutionary history of geographic range on the maximum clade credibility tree of Felidae using RevBayes. Labels for geographic regions follow Fig. S2.

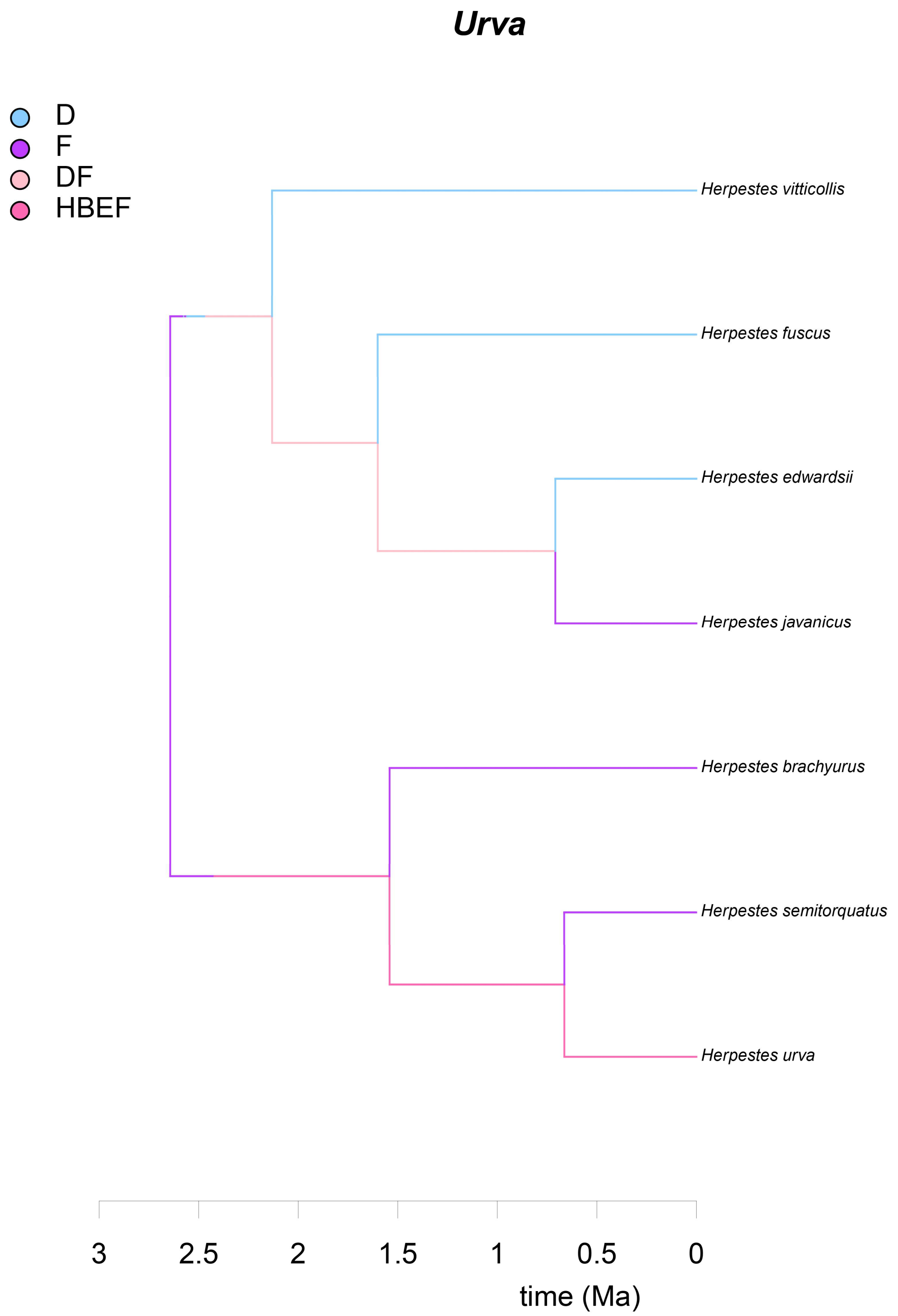

Fig. S68. Marginal maximum *a posteriori* reconstruction of the evolutionary history of geographic range on the maximum clade credibility tree of *Urva* using RevBayes. Labels for geographic regions follow Fig. S2.

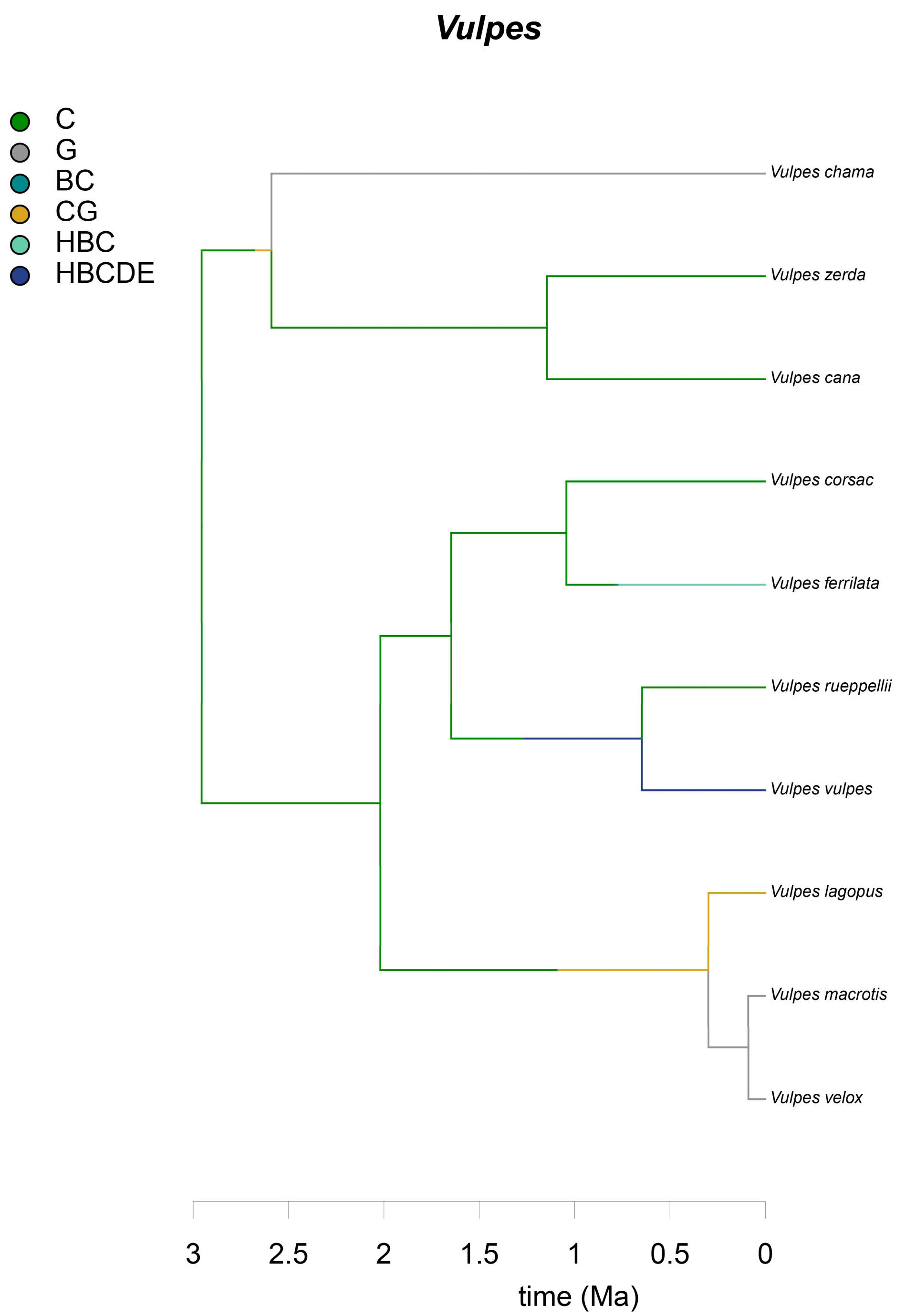

Fig. S69. Marginal maximum *a posteriori* reconstruction of the evolutionary history of geographic range on the maximum clade credibility tree of *Vulpes* using RevBayes. Labels for geographic regions follow Fig. S2.

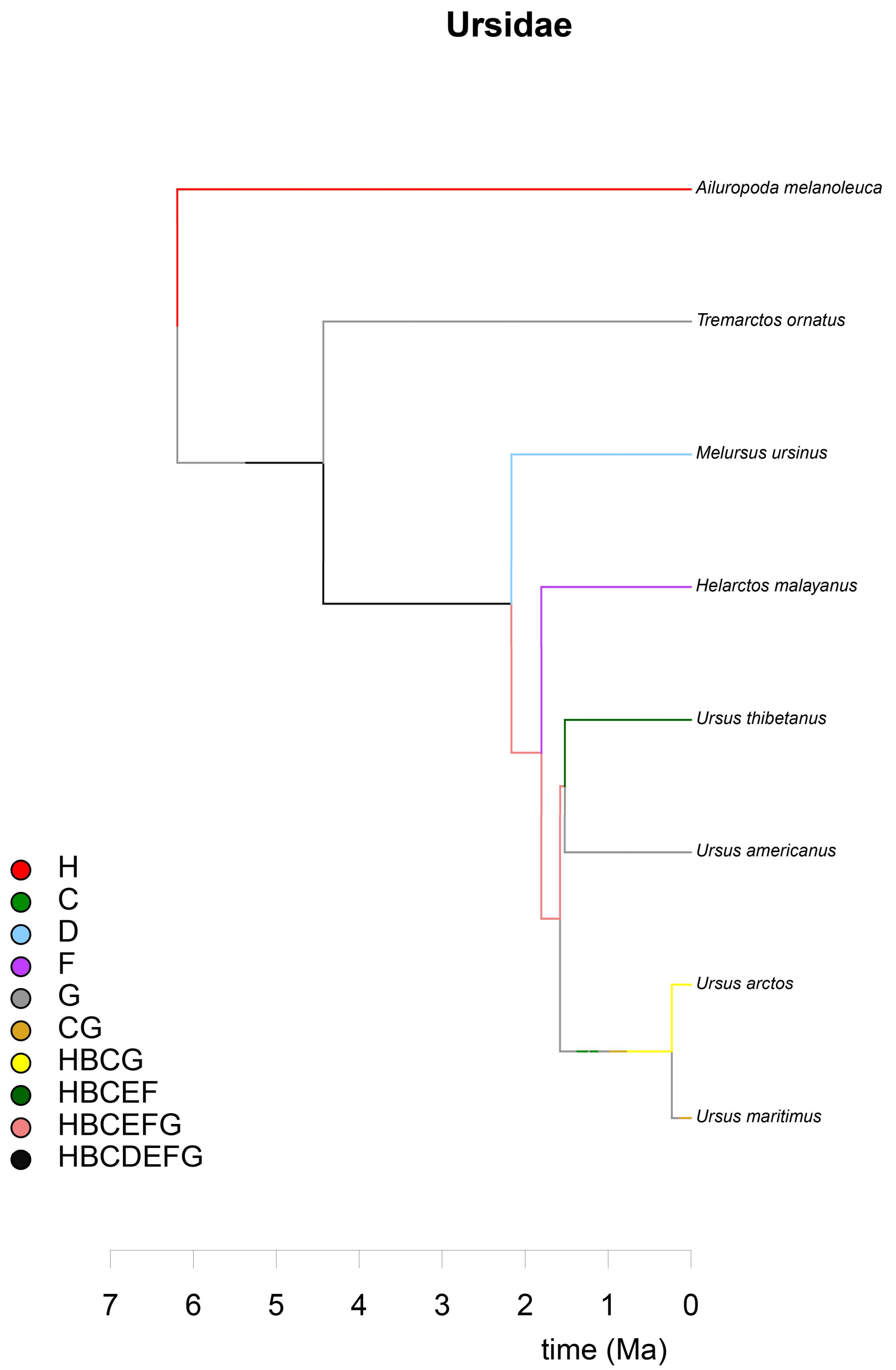

Fig. S70. Marginal maximum *a posteriori* reconstruction of the evolutionary history of geographic range on the maximum clade credibility tree of Ursidae using RevBayes. Labels for geographic regions follow Fig. S2.

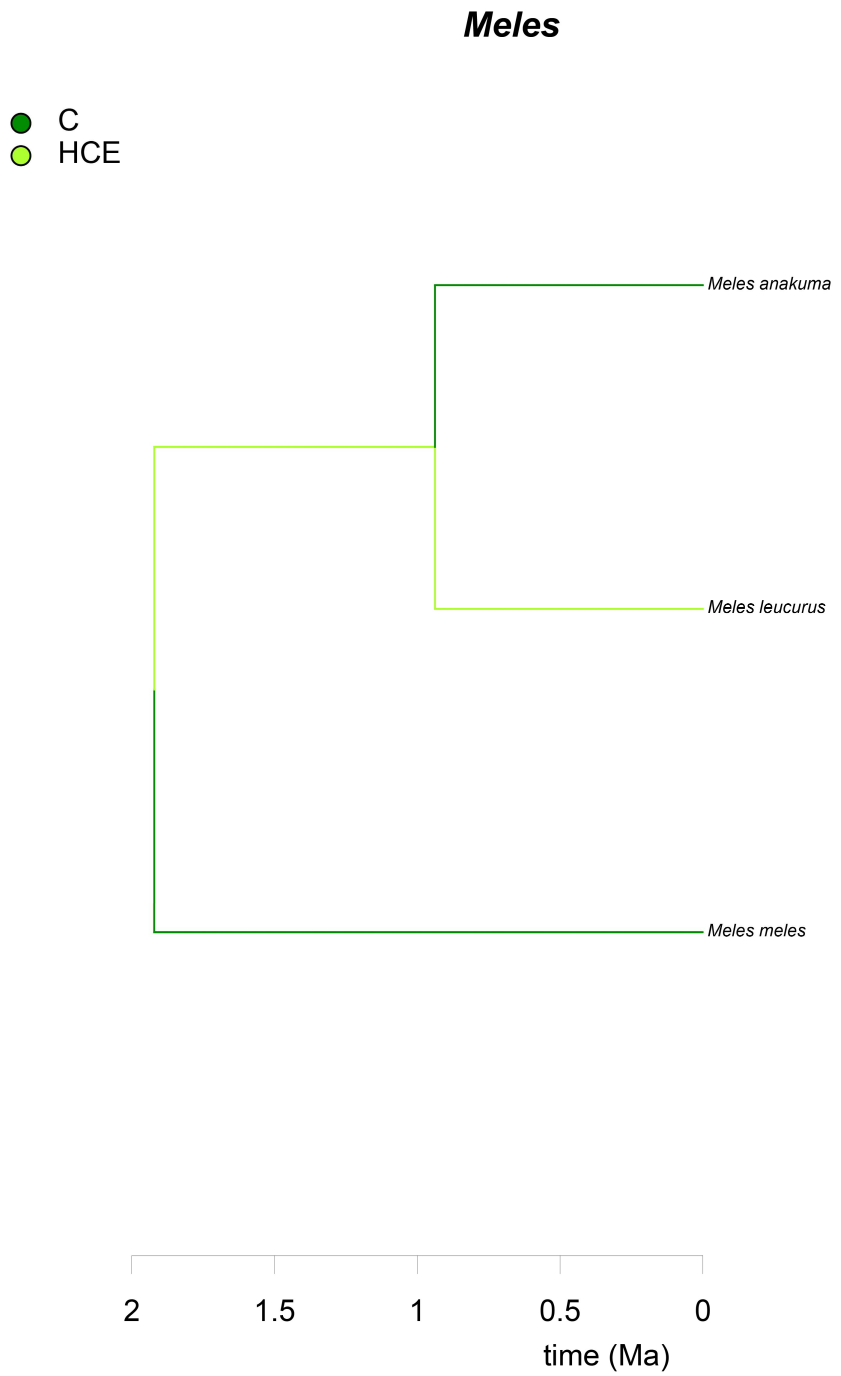

Fig. S71. Marginal maximum *a posteriori* reconstruction of the evolutionary history of geographic range on the maximum clade credibility tree of *Meles* using RevBayes. Labels for geographic regions follow Fig. S2.

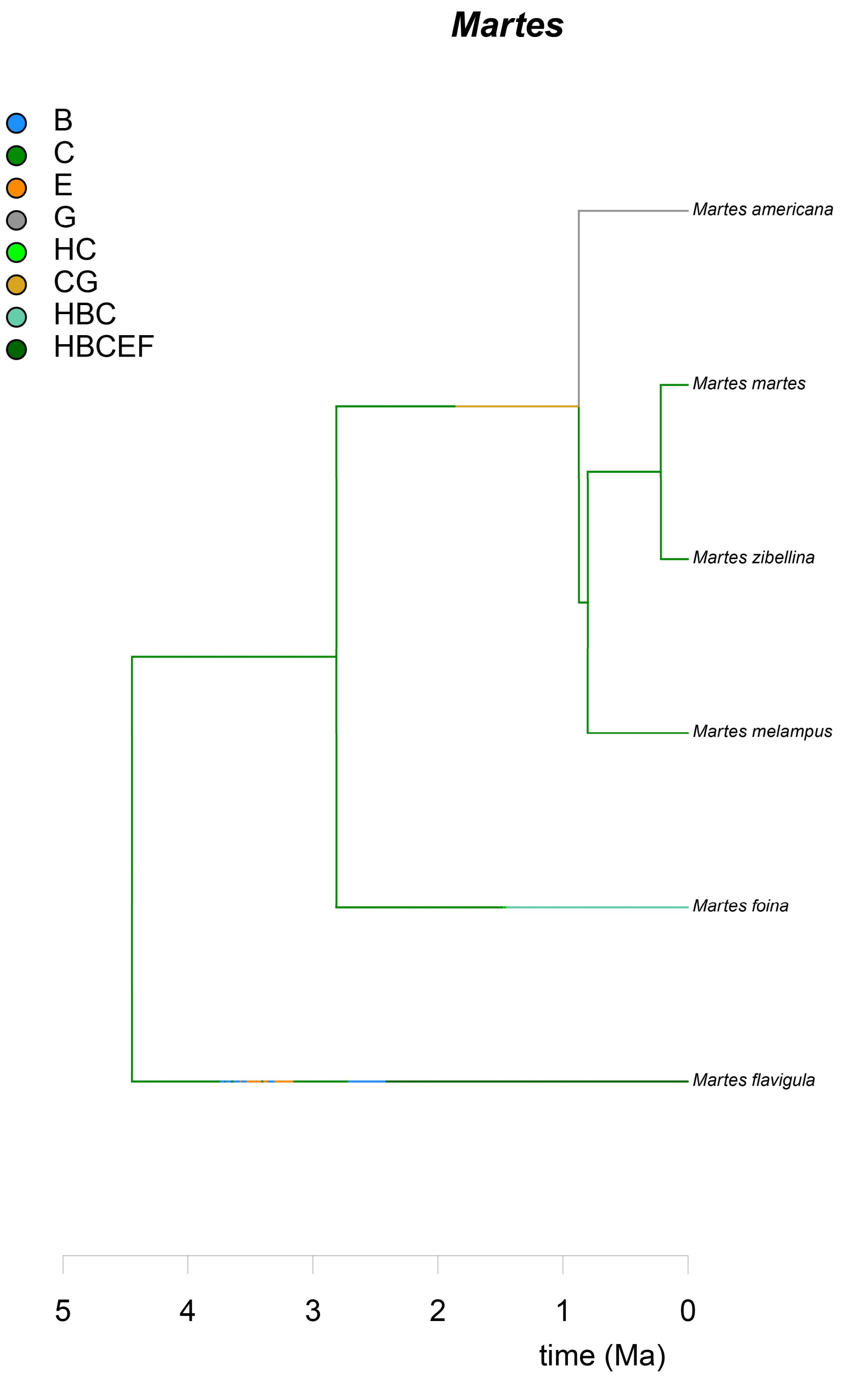

Fig. S72. Marginal maximum *a posteriori* reconstruction of the evolutionary history of geographic range on the maximum clade credibility tree of *Martes* using RevBayes. Labels for geographic regions follow Fig. S2.

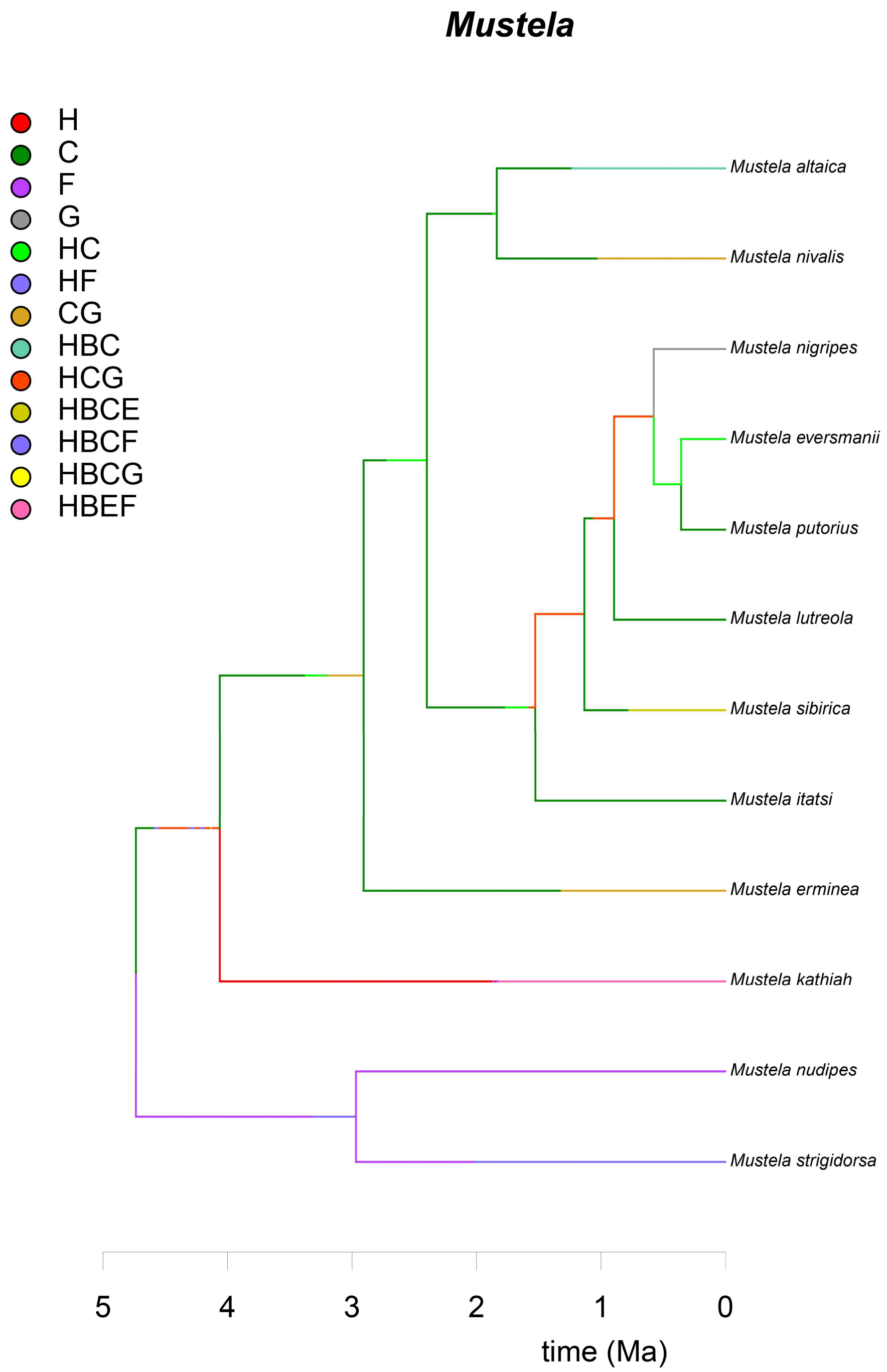

Fig. S73. Marginal maximum *a posteriori* reconstruction of the evolutionary history of geographic range on the maximum clade credibility tree of *Mustela* using RevBayes. Labels for geographic regions follow Fig. S2.

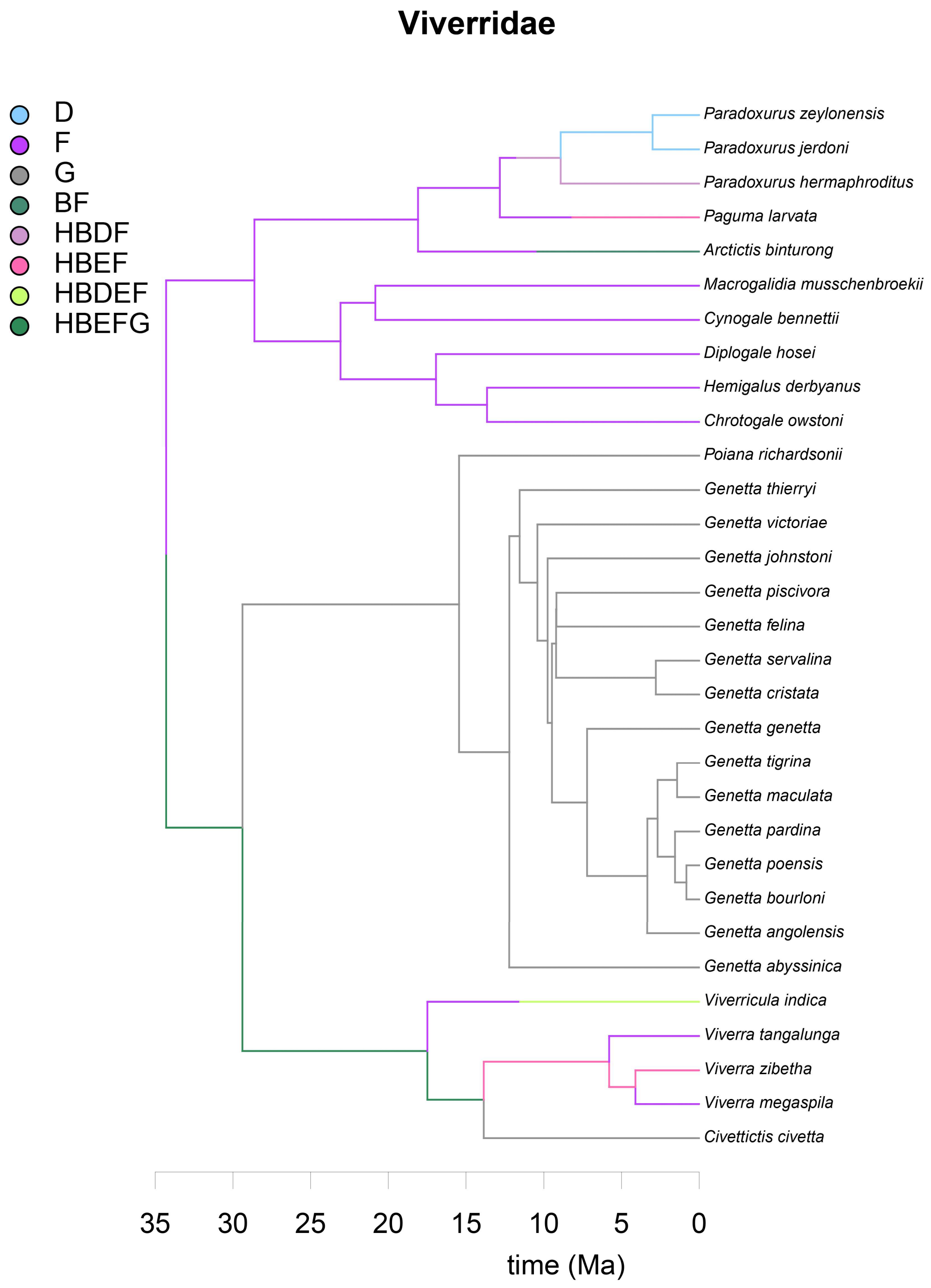

Fig. S74. Marginal maximum *a posteriori* reconstruction of the evolutionary history of geographic range on the maximum clade credibility tree of Viverridae using RevBayes. Labels for geographic regions follow Fig. S2.

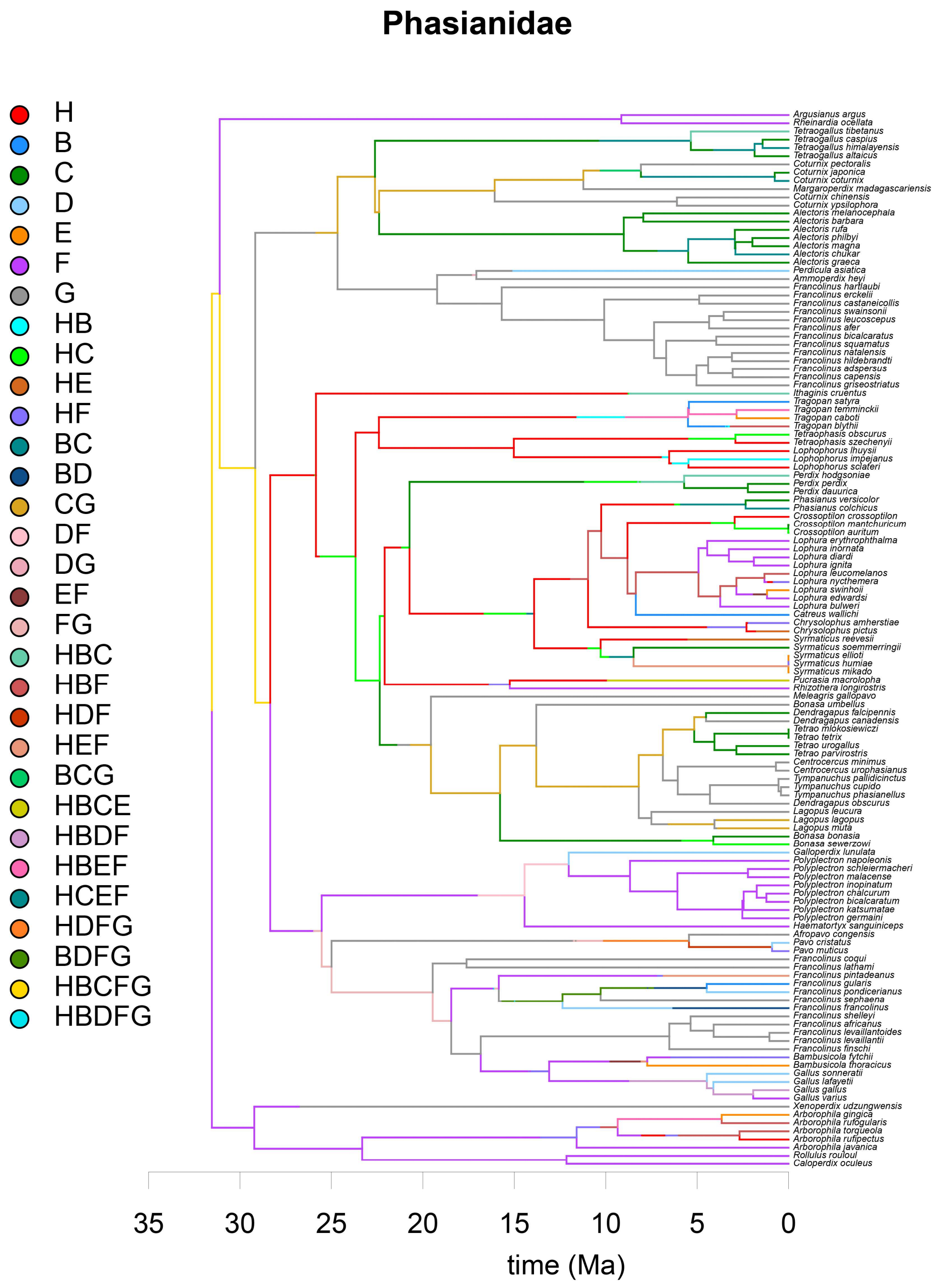

Fig. S75. Marginal maximum *a posteriori* reconstruction of the evolutionary history of geographic range on the maximum clade credibility tree of Phasianidae using RevBayes. Labels for geographic regions follow Fig. S2.

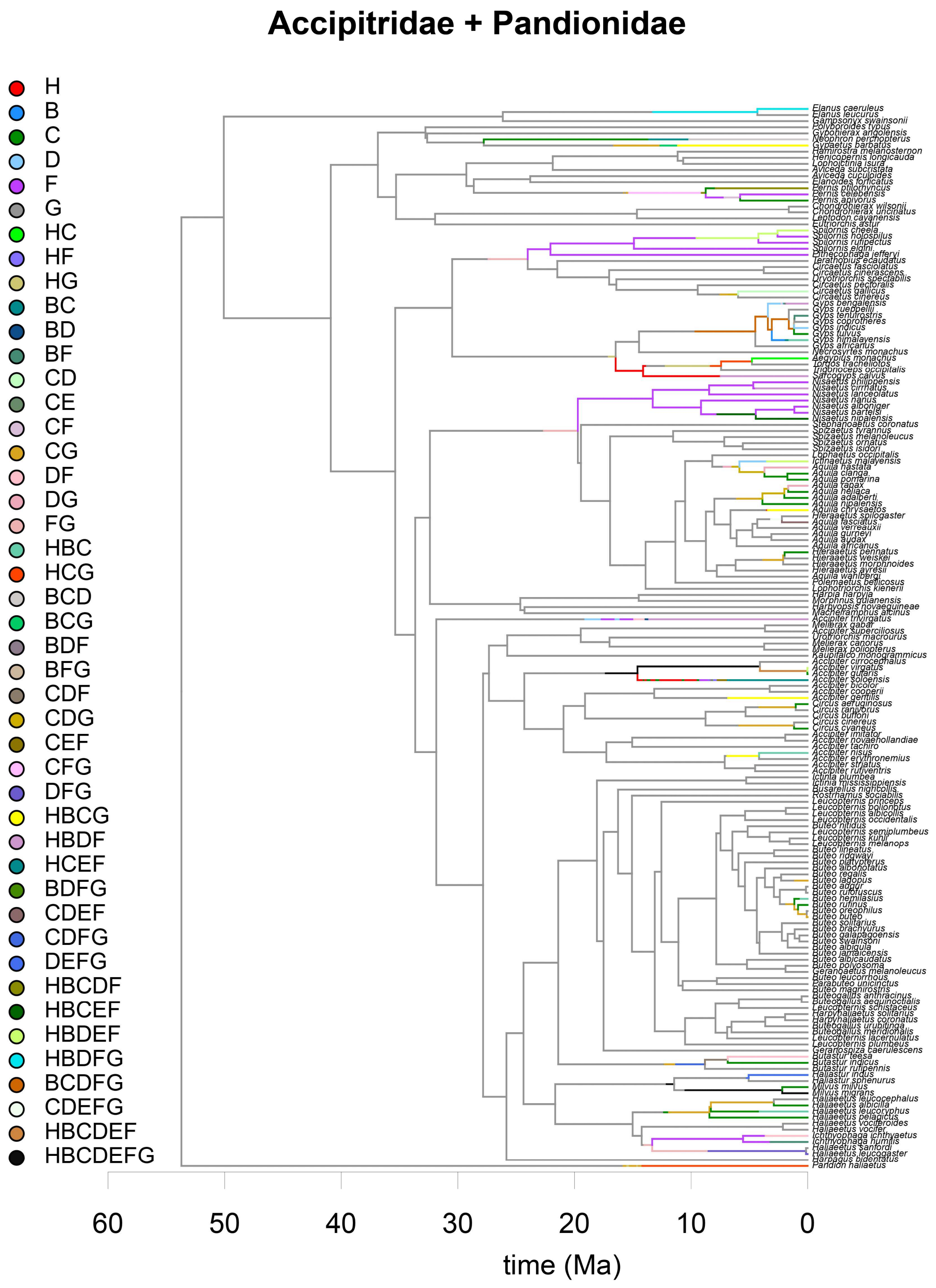

Fig. S76. Marginal maximum *a posteriori* reconstruction of the evolutionary history of geographic range on the maximum clade credibility tree of Accipitridae + Pandionidae using RevBayes. Labels for geographic regions follow Fig. S2.

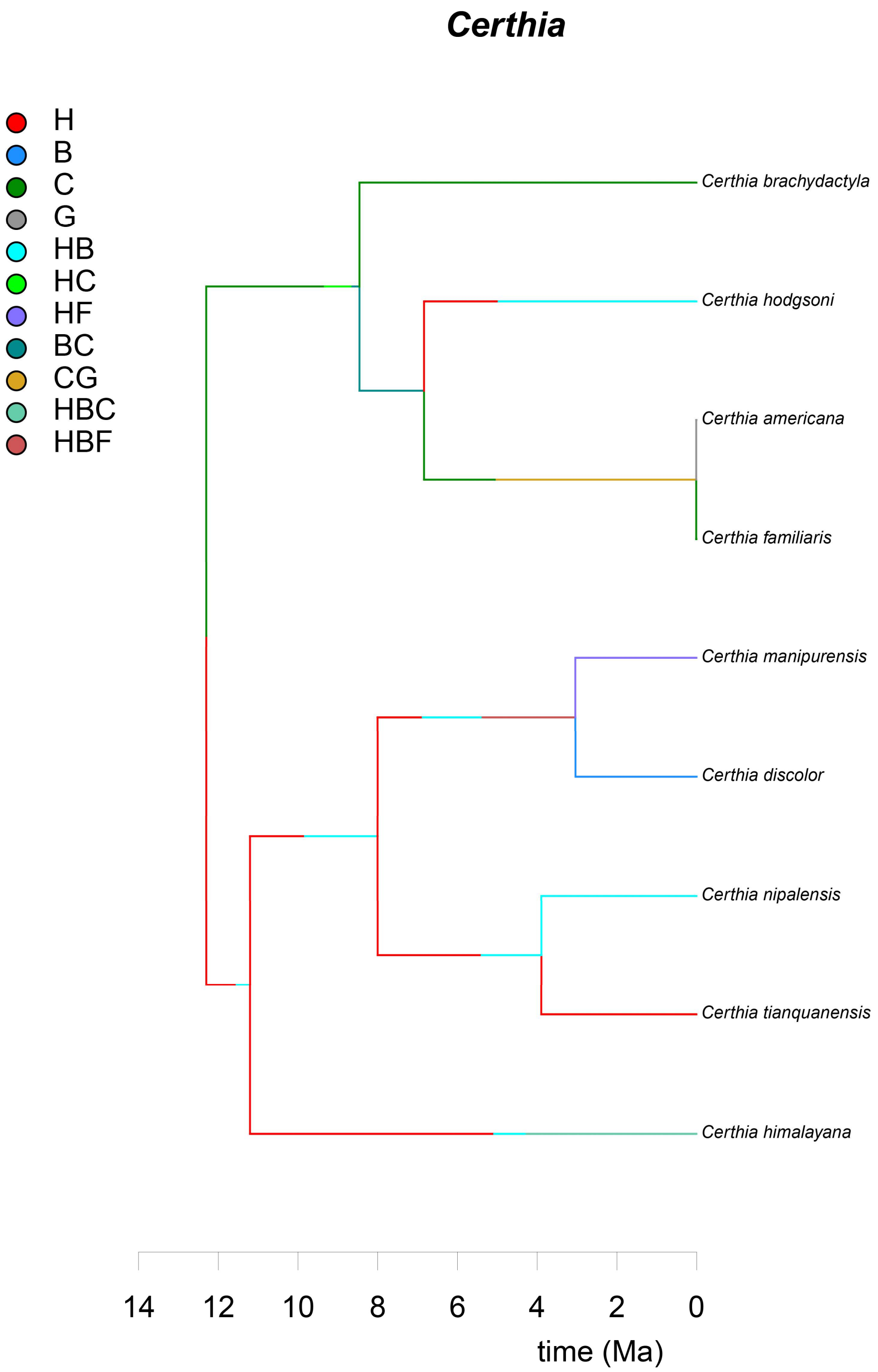

Fig. S77. Marginal maximum *a posteriori* reconstruction of the evolutionary history of geographic range on the maximum clade credibility tree of *Certhia* using RevBayes. Labels for geographic regions follow Fig. S2.

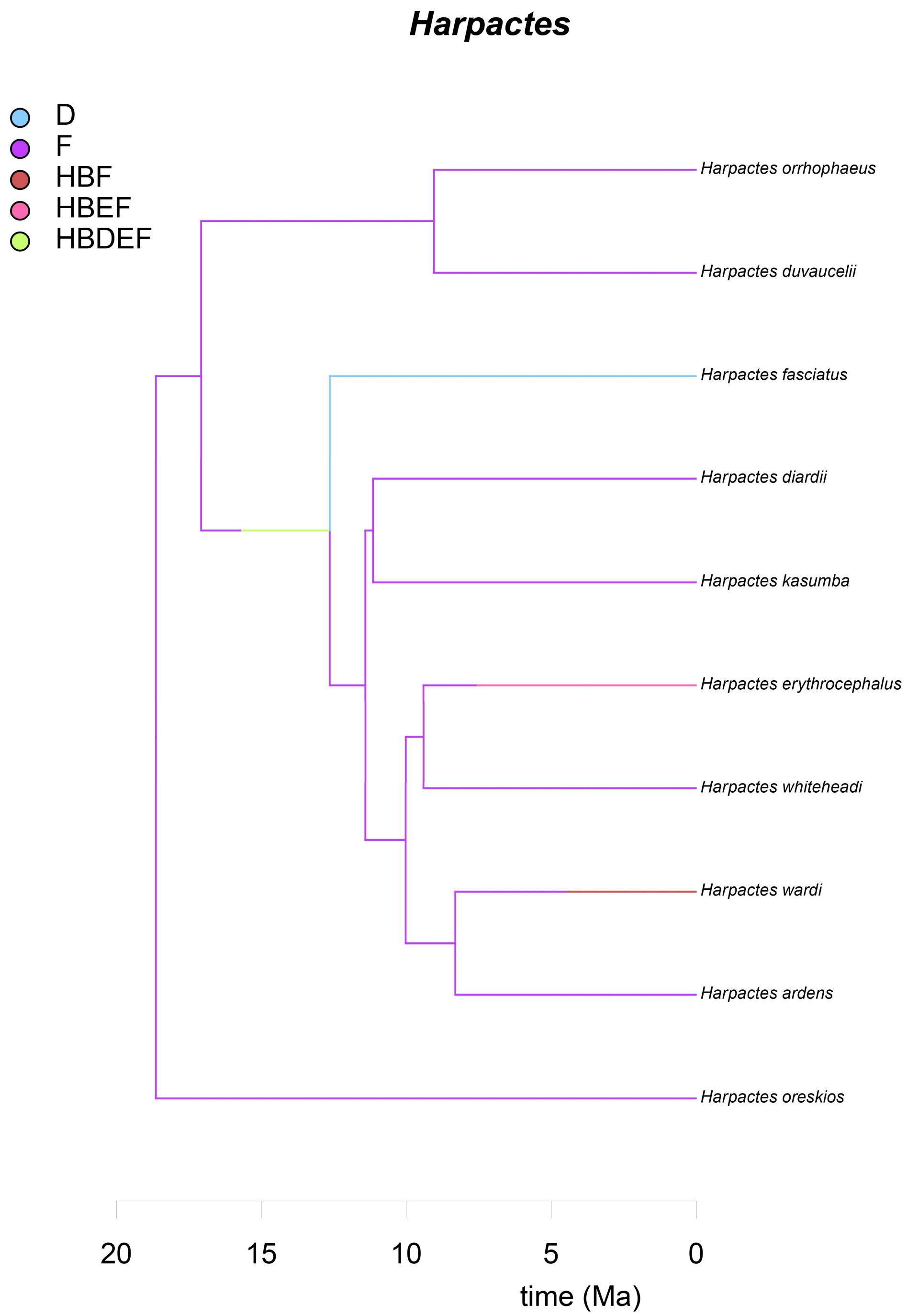

Fig. S78. Marginal maximum *a posteriori* reconstruction of the evolutionary history of geographic range on the maximum clade credibility tree of *Harpactes* using RevBayes. Labels for geographic regions follow Fig. S2.

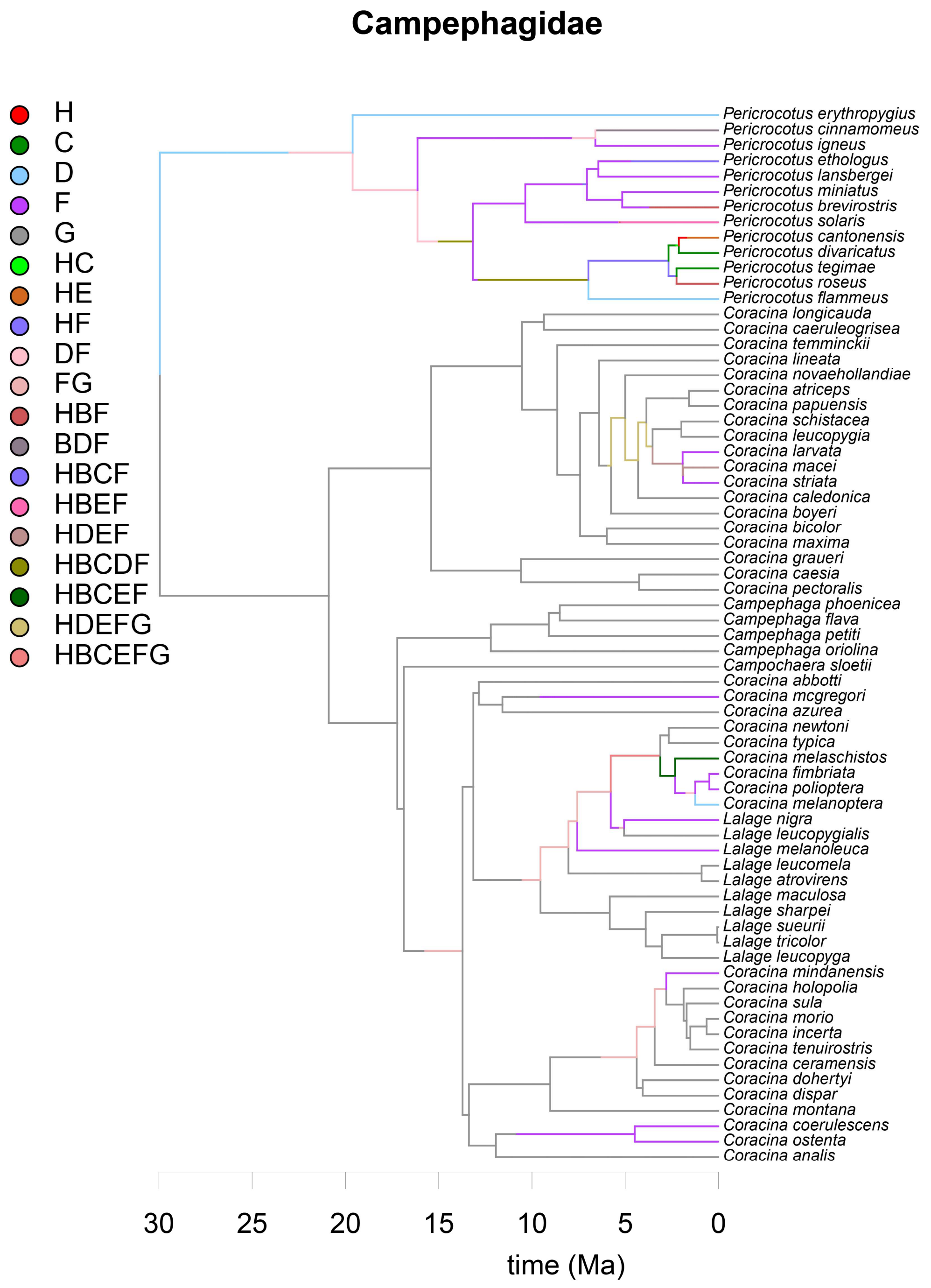

Fig. S79. Marginal maximum *a posteriori* reconstruction of the evolutionary history of geographic range on the maximum clade credibility tree of Campephagidae using RevBayes. Labels for geographic regions follow Fig. S2.

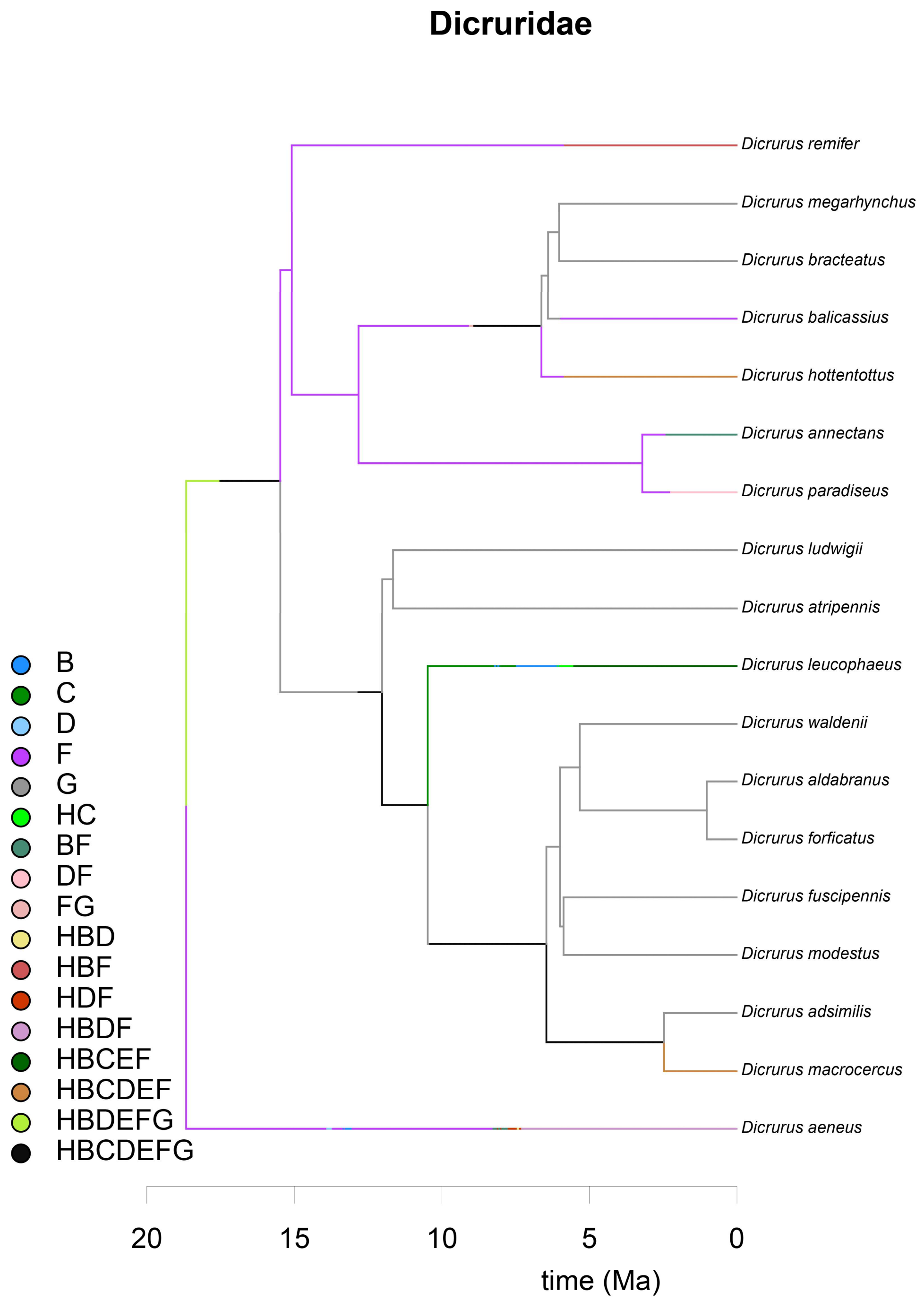

Fig. S80. Marginal maximum *a posteriori* reconstruction of the evolutionary history of geographic range on the maximum clade credibility tree of Dicruridae using RevBayes. Labels for geographic regions follow Fig. S2.

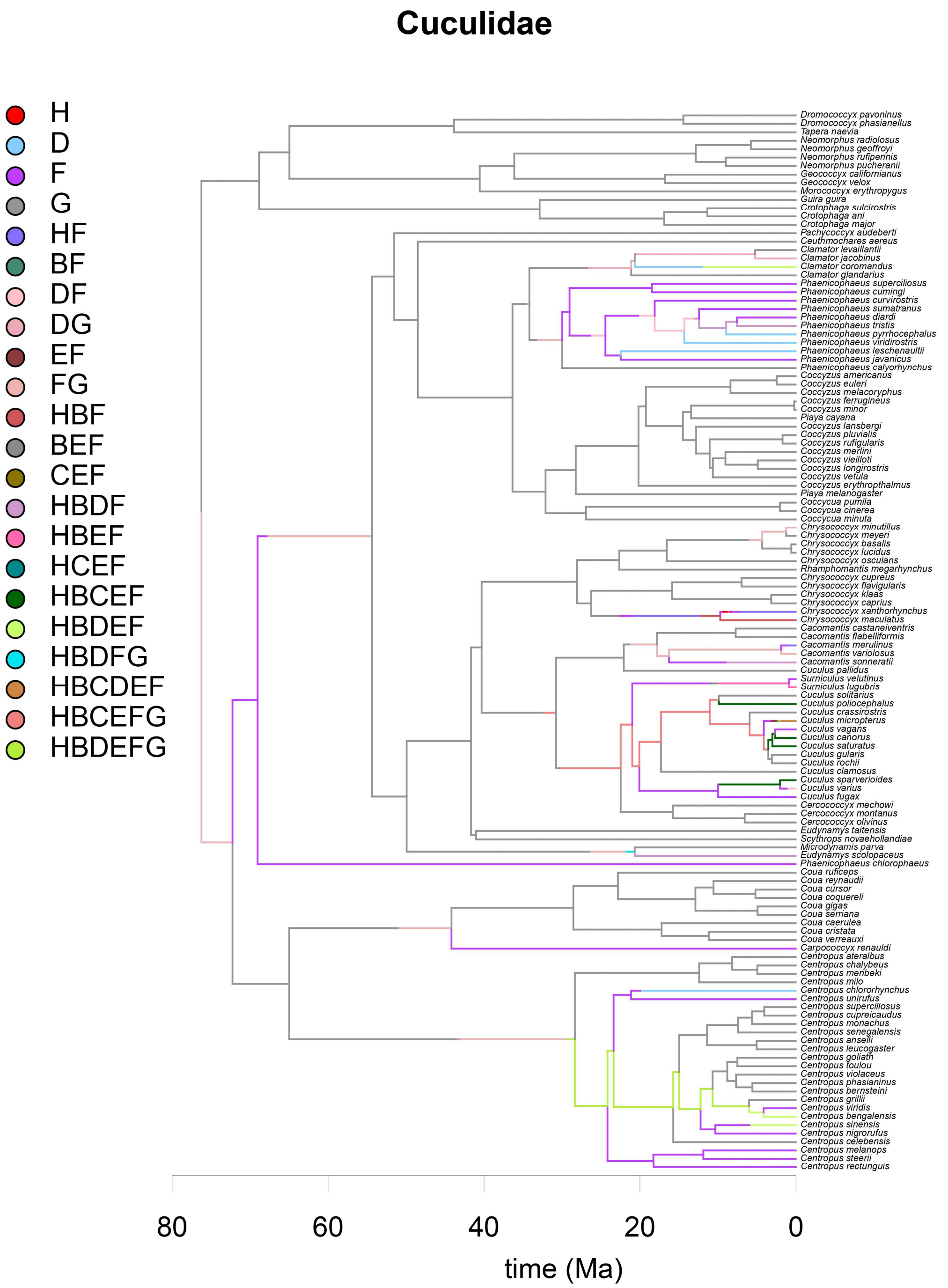

Fig. S81. Marginal maximum *a posteriori* reconstruction of the evolutionary history of geographic range on the maximum clade credibility tree of Cuculidae using RevBayes. Labels for geographic regions follow Fig. S2.

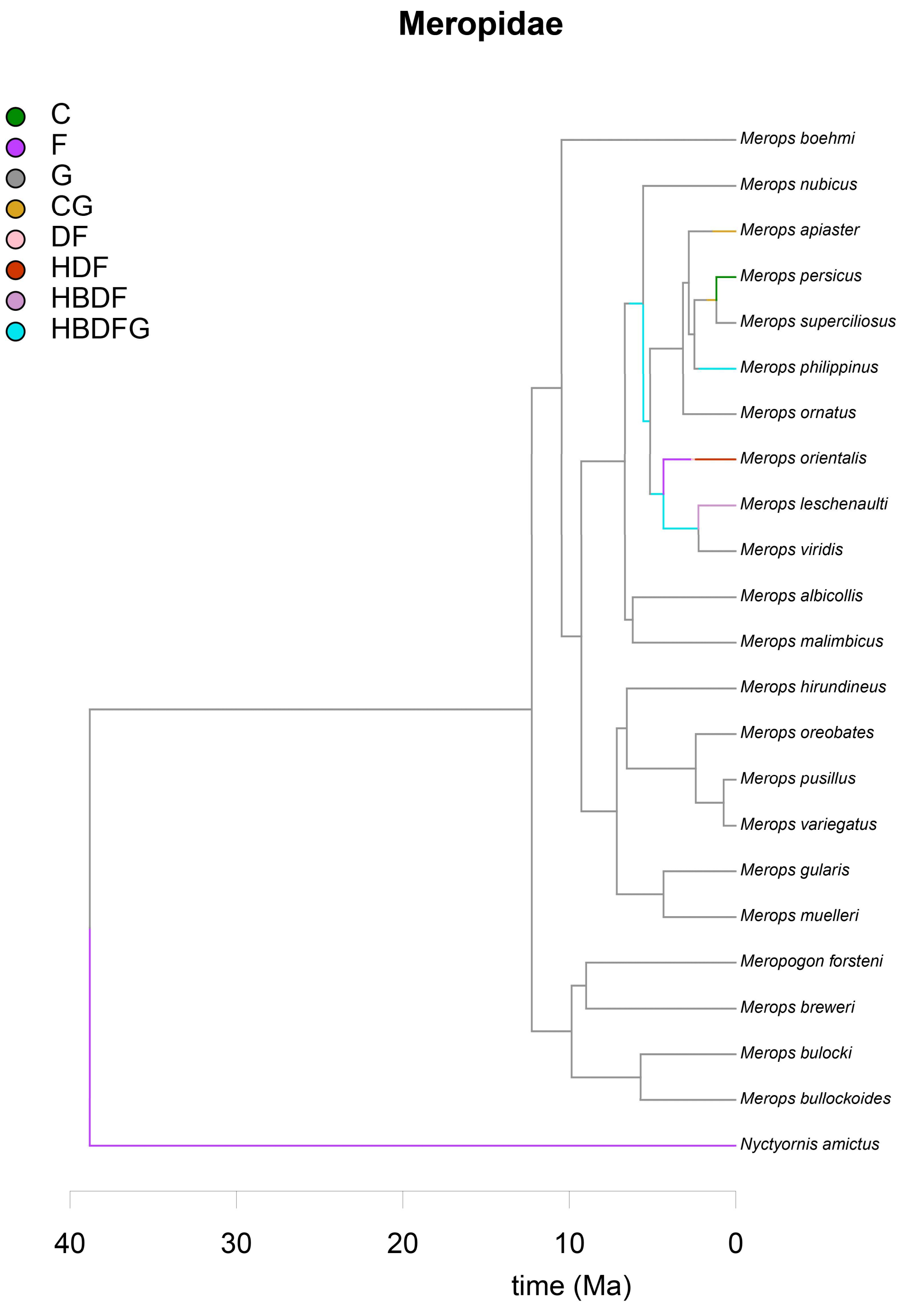

Fig. S82. Marginal maximum *a posteriori* reconstruction of the evolutionary history of geographic range on the maximum clade credibility tree of Meropidae using RevBayes. Labels for geographic regions follow Fig. S2.

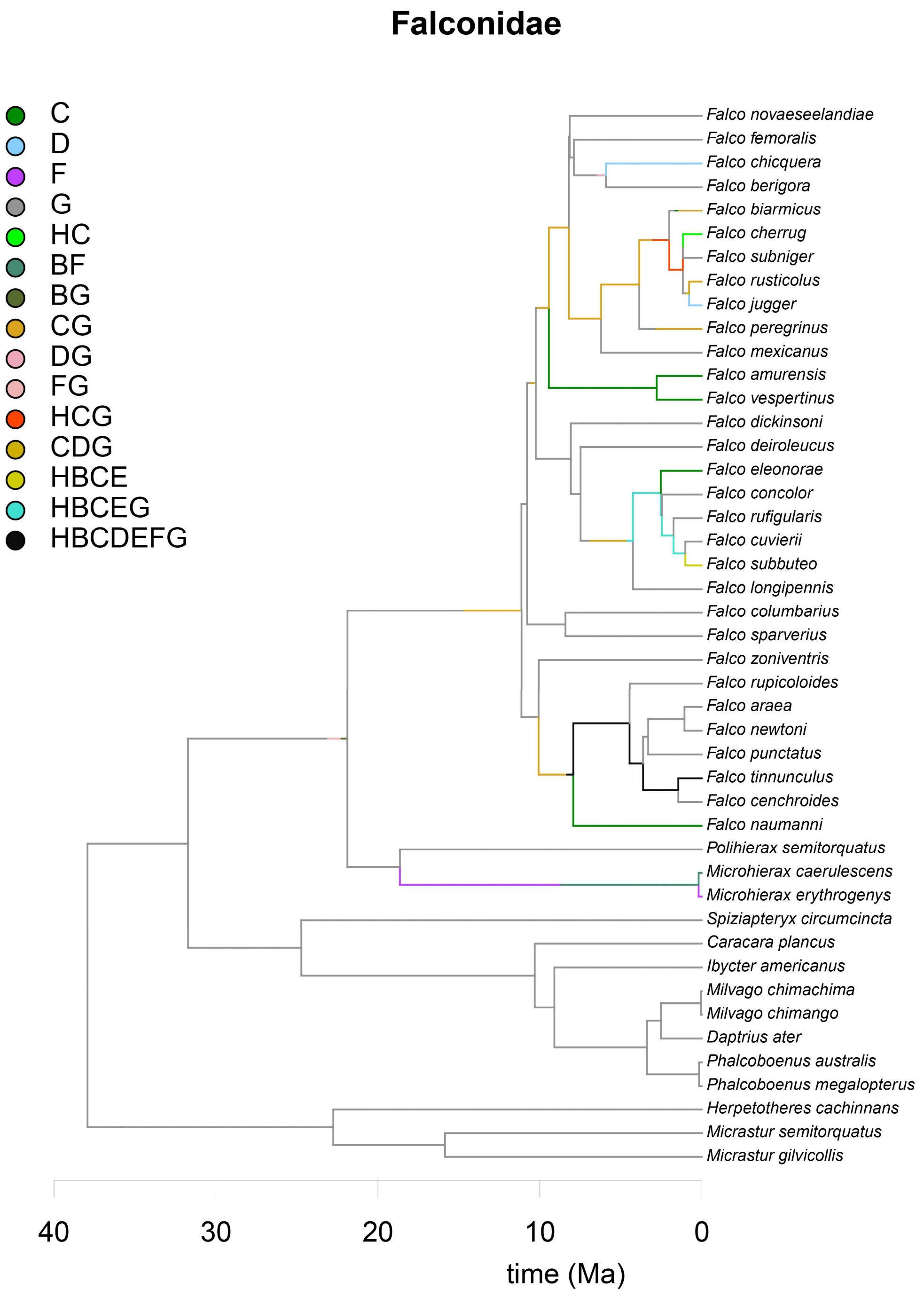

Fig. S83. Marginal maximum *a posteriori* reconstruction of the evolutionary history of geographic range on the maximum clade credibility tree of Falconidae using RevBayes. Labels for geographic regions follow Fig. S2.

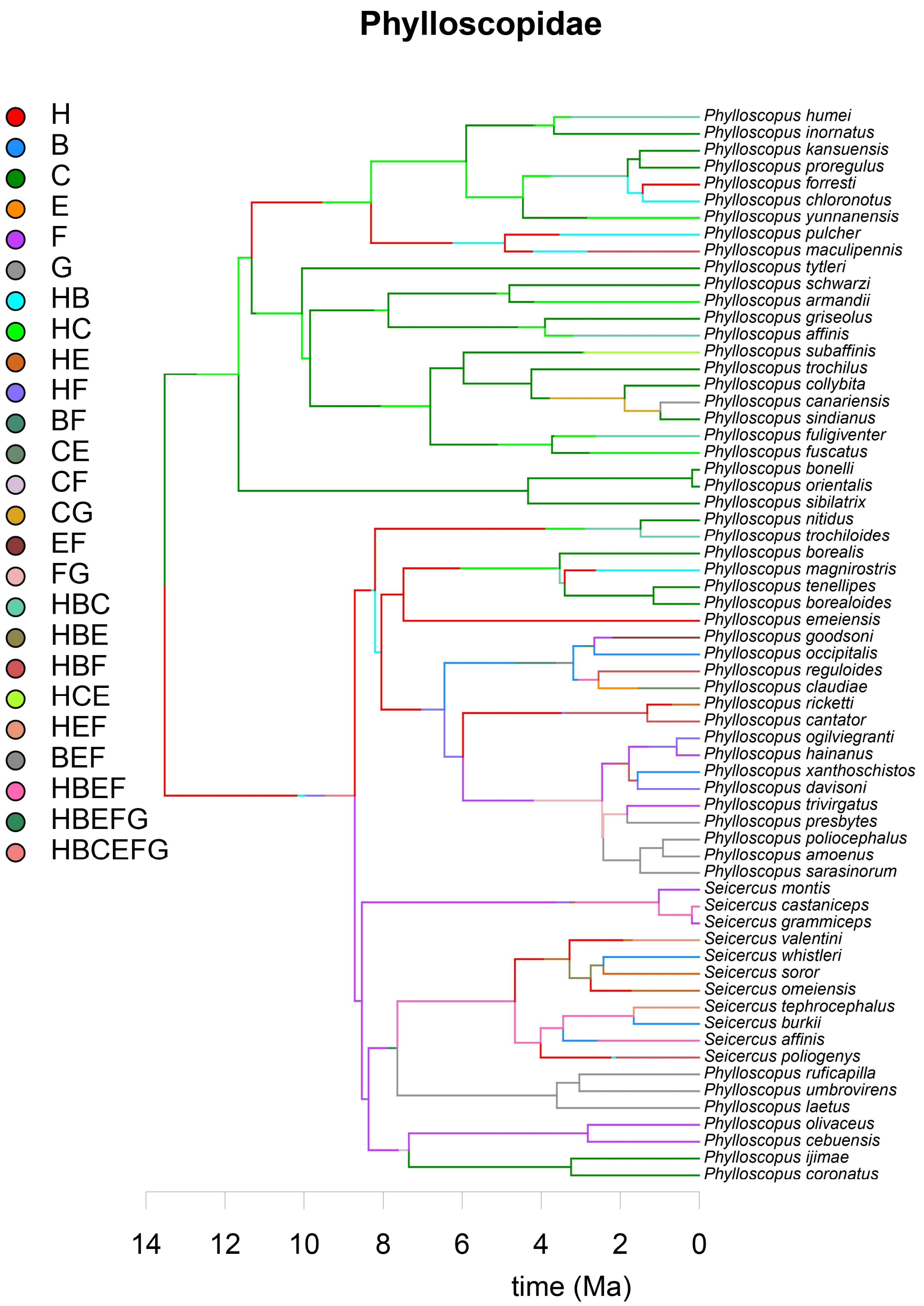

Fig. S84. Marginal maximum *a posteriori* reconstruction of the evolutionary history of geographic range on the maximum clade credibility tree of Phylloscopidae using RevBayes. Labels for geographic regions follow Fig. S2.

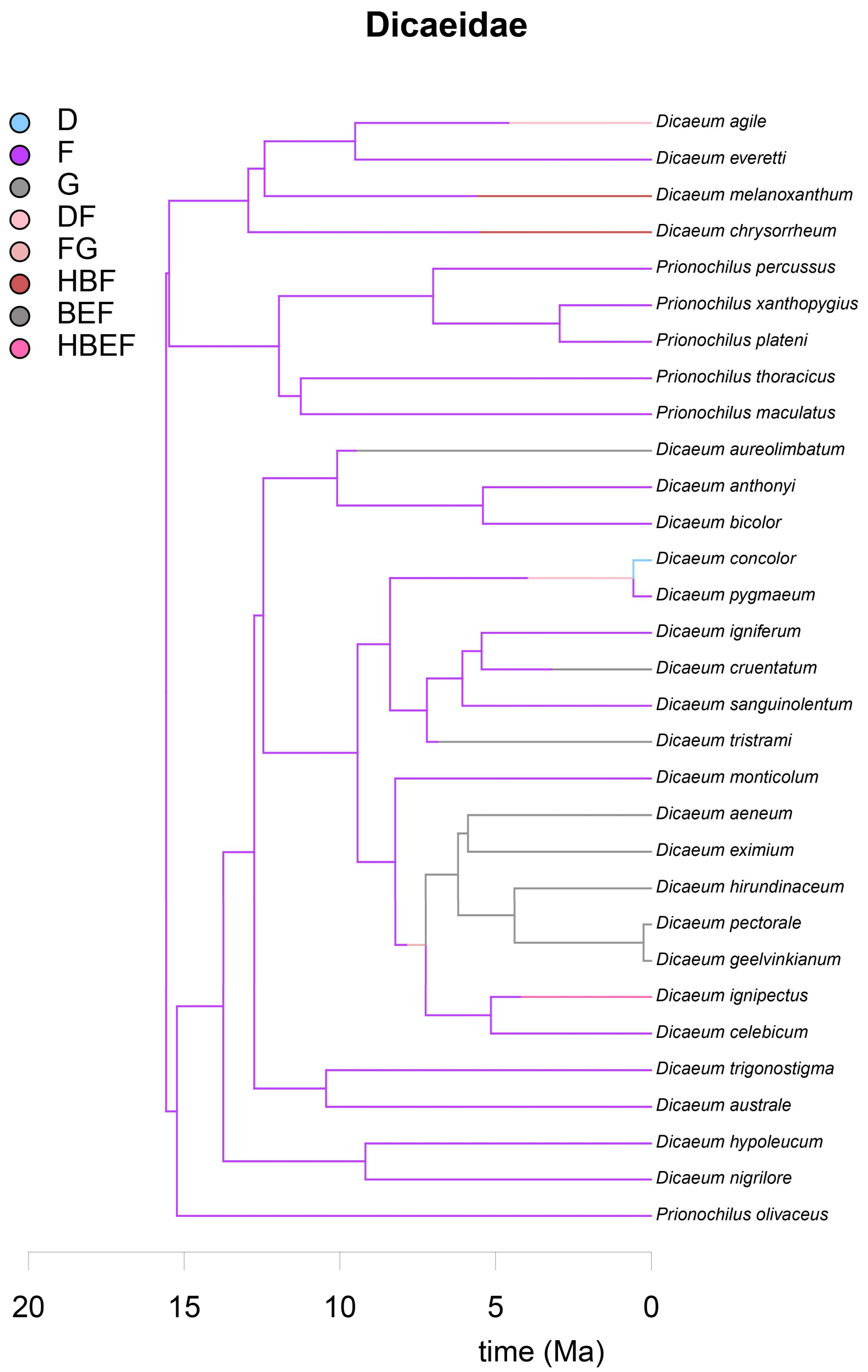

Fig. S85. Marginal maximum *a posteriori* reconstruction of the evolutionary history of geographic range on the maximum clade credibility tree of Dicaeidae using RevBayes. Labels for geographic regions follow Fig. S2.

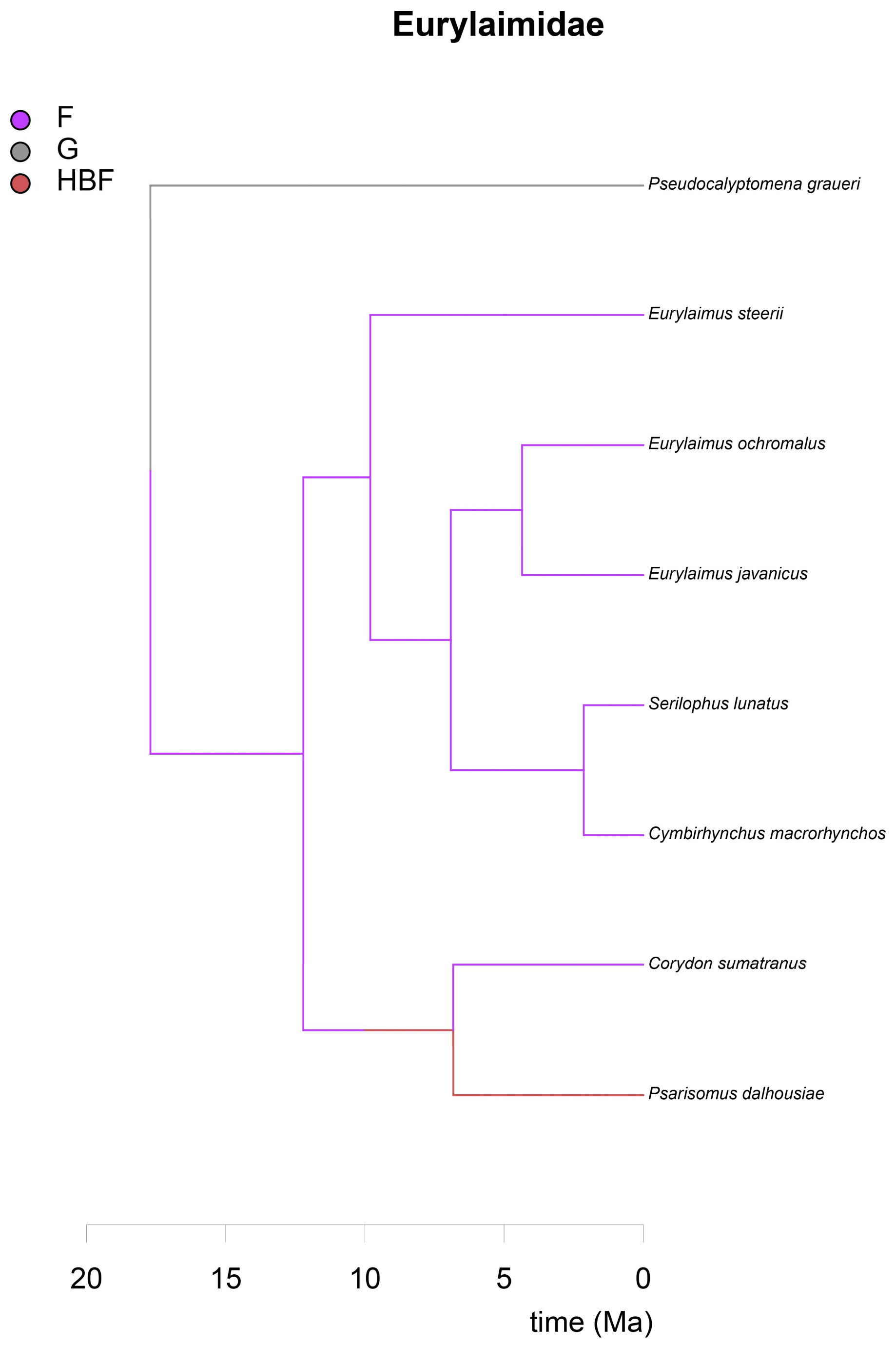

Fig. S86. Marginal maximum *a posteriori* reconstruction of the evolutionary history of geographic range on the maximum clade credibility tree of Eurylaimidae using RevBayes. Labels for geographic regions follow Fig. S2.

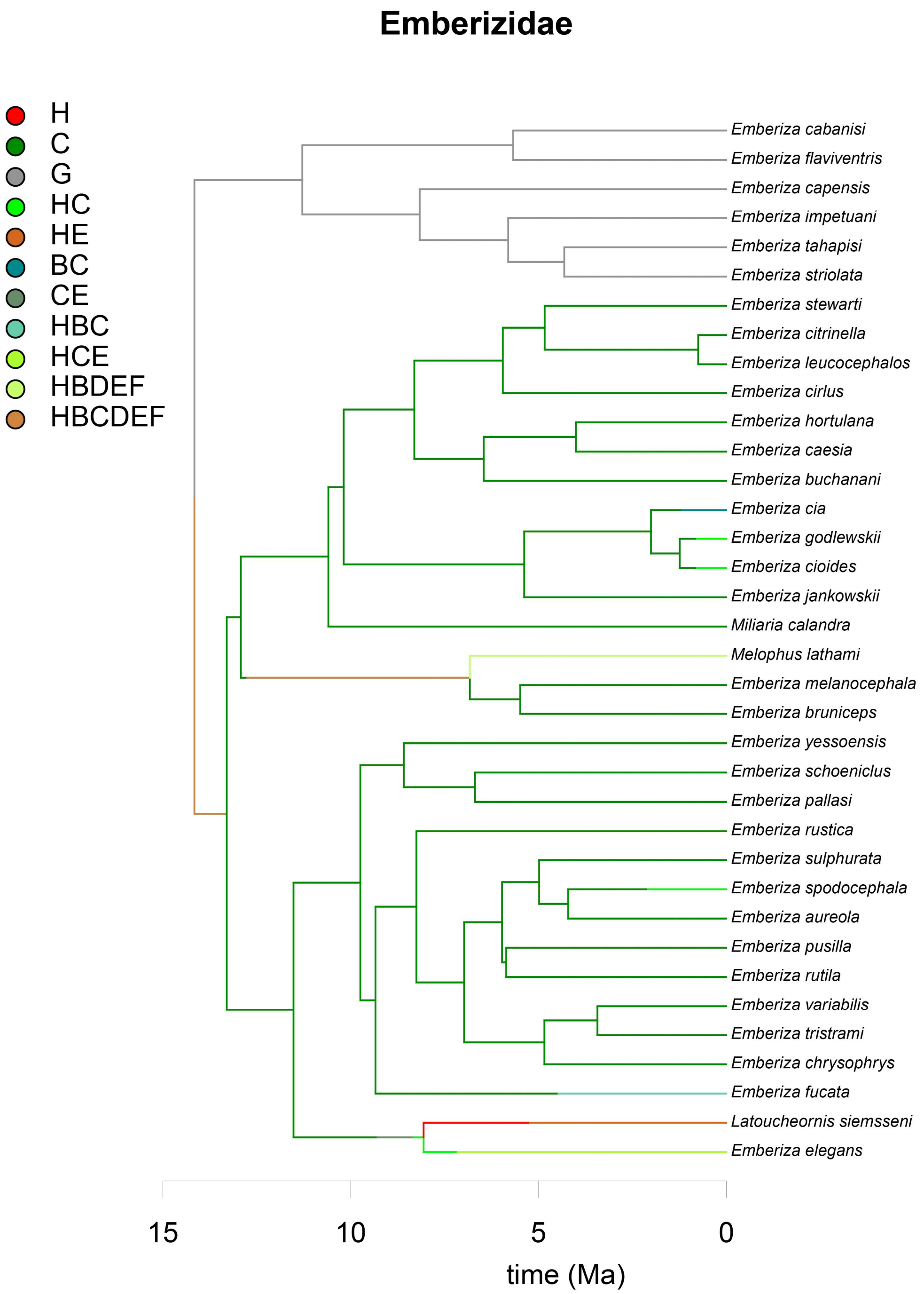

Fig. S87. Marginal maximum *a posteriori* reconstruction of the evolutionary history of geographic range on the maximum clade credibility tree of Emberizidae using RevBayes. Labels for geographic regions follow Fig. S2.

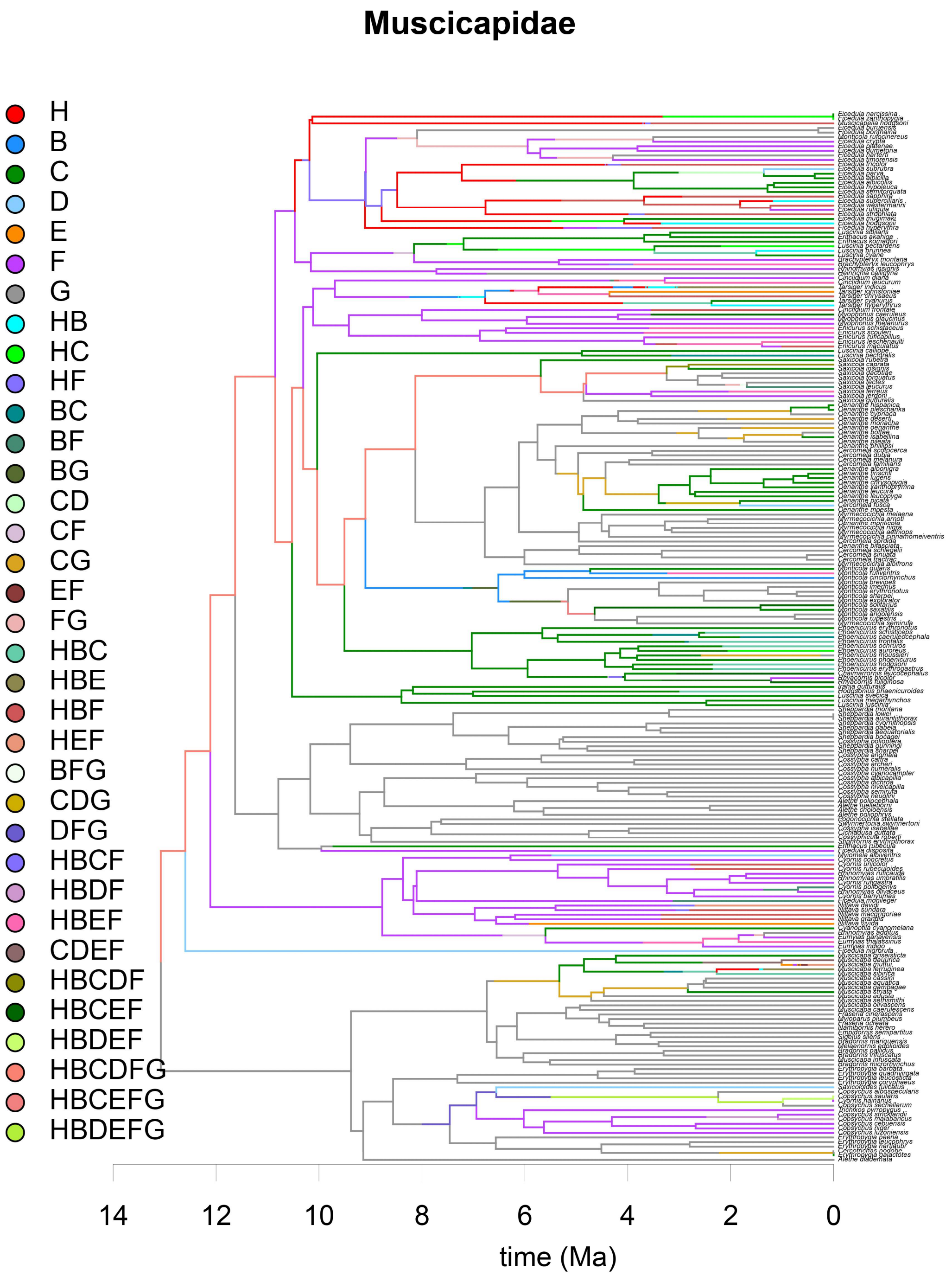

Fig. S88. Marginal maximum *a posteriori* reconstruction of the evolutionary history of geographic range on the maximum clade credibility tree of Muscicapidae using RevBayes. Labels for geographic regions follow Fig. S2.

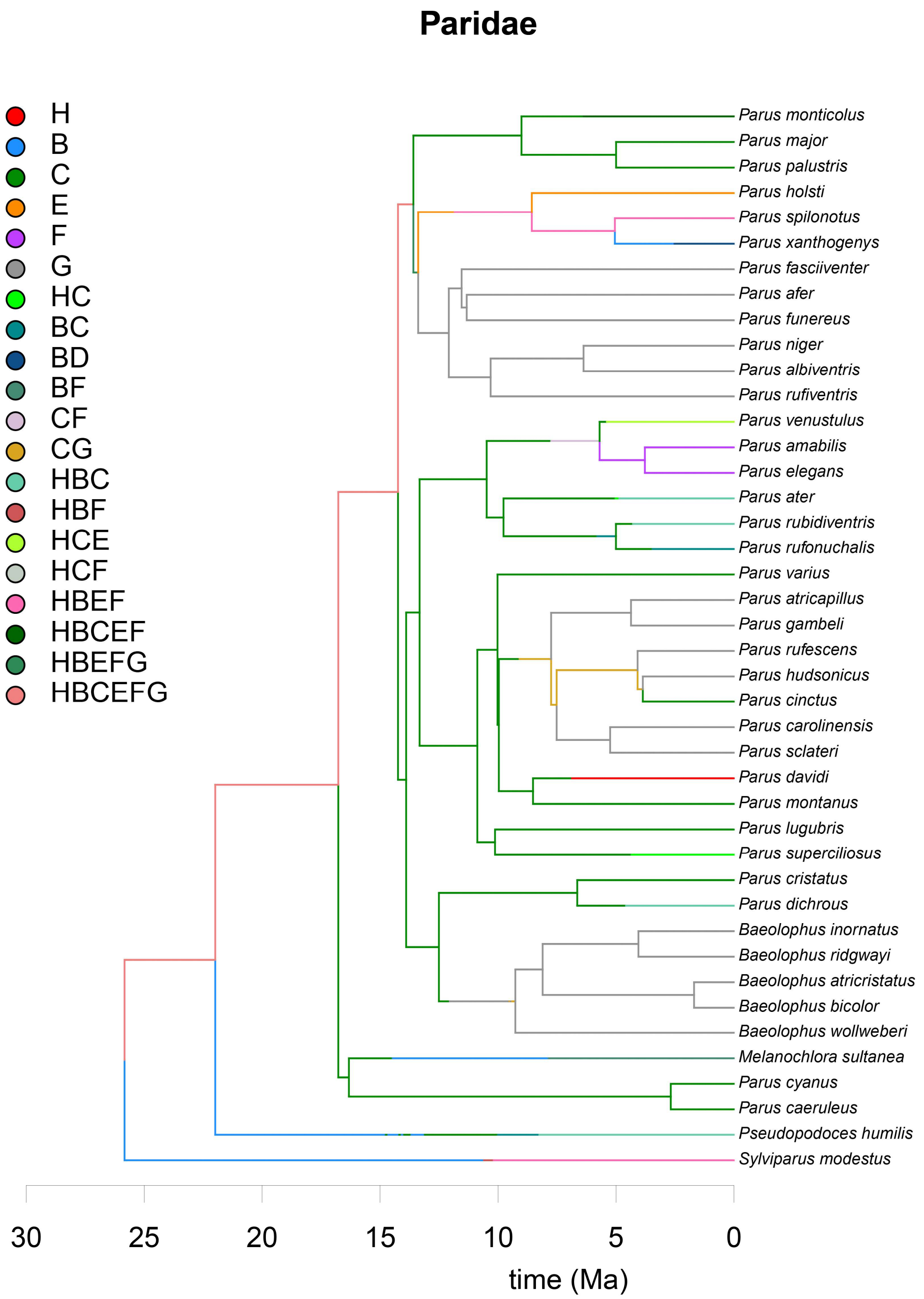

Fig. S89. Marginal maximum *a posteriori* reconstruction of the evolutionary history of geographic range on the maximum clade credibility tree of Paridae using RevBayes. Labels for geographic regions follow Fig. S2.

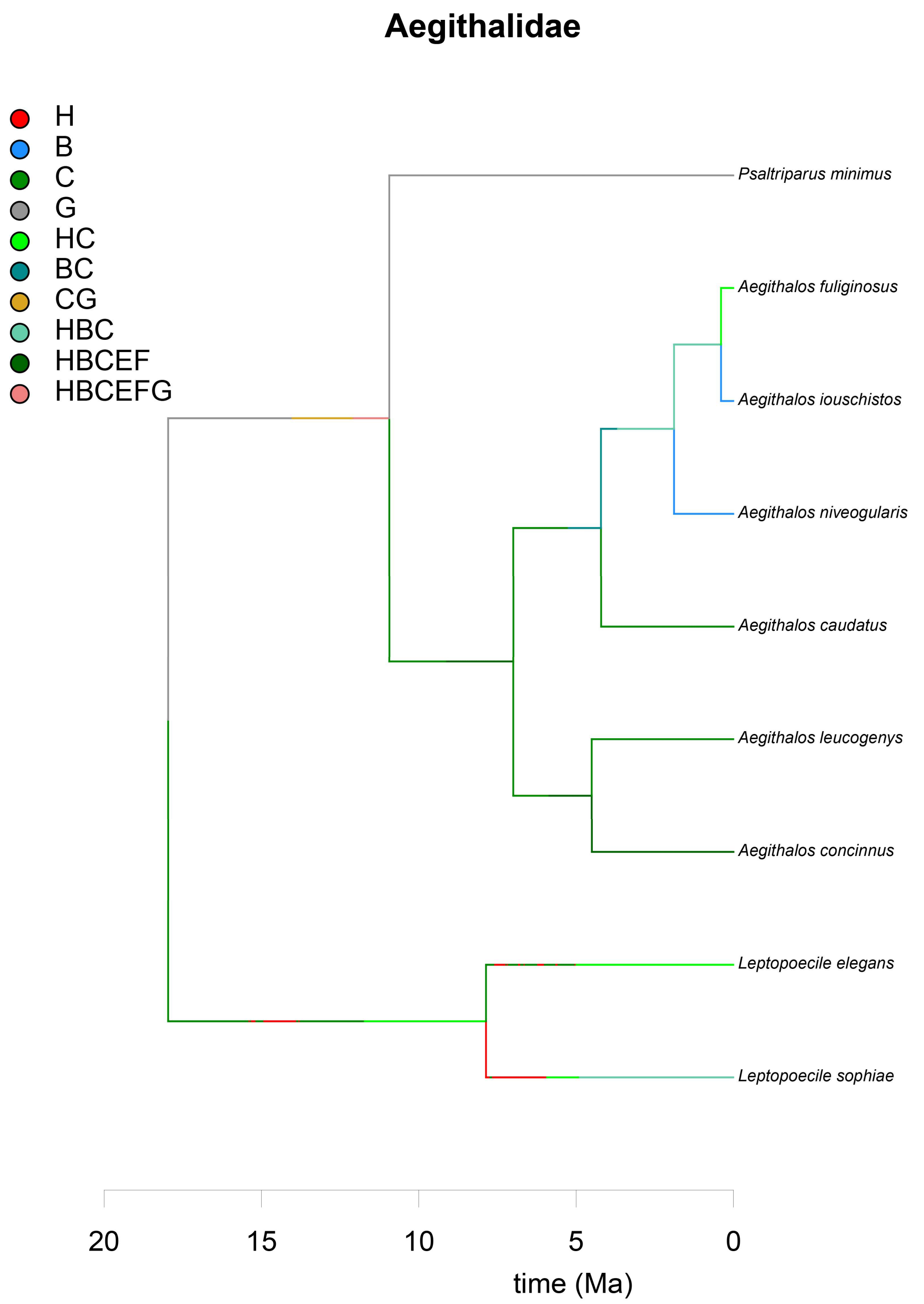

Fig. S90. Marginal maximum *a posteriori* reconstruction of the evolutionary history of geographic range on the maximum clade credibility tree of Aegithalidae using RevBayes. Labels for geographic regions follow Fig. S2.

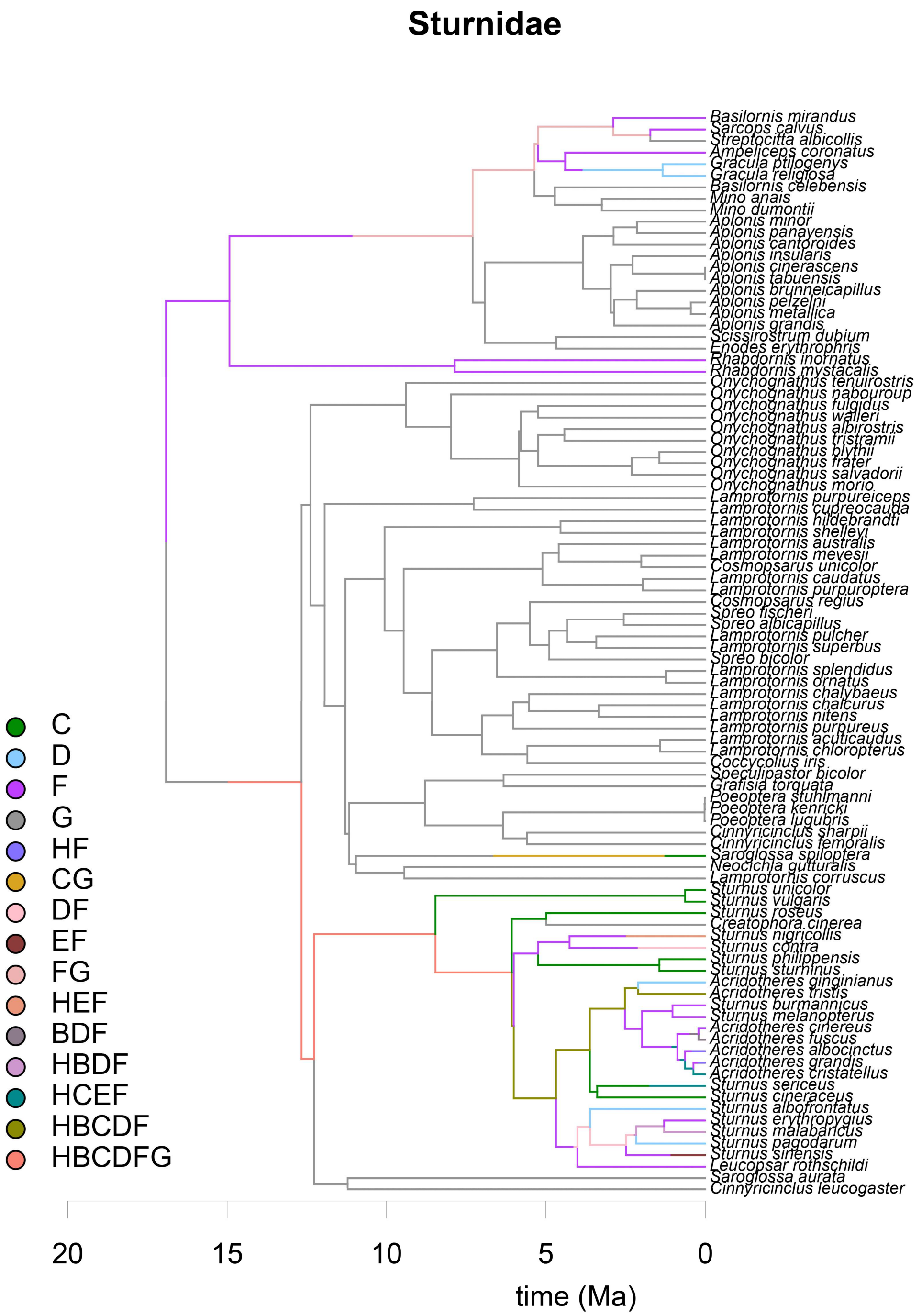

Fig. S91. Marginal maximum *a posteriori* reconstruction of the evolutionary history of geographic range on the maximum clade credibility tree of Sturnidae using RevBayes. Labels for geographic regions follow Fig. S2.

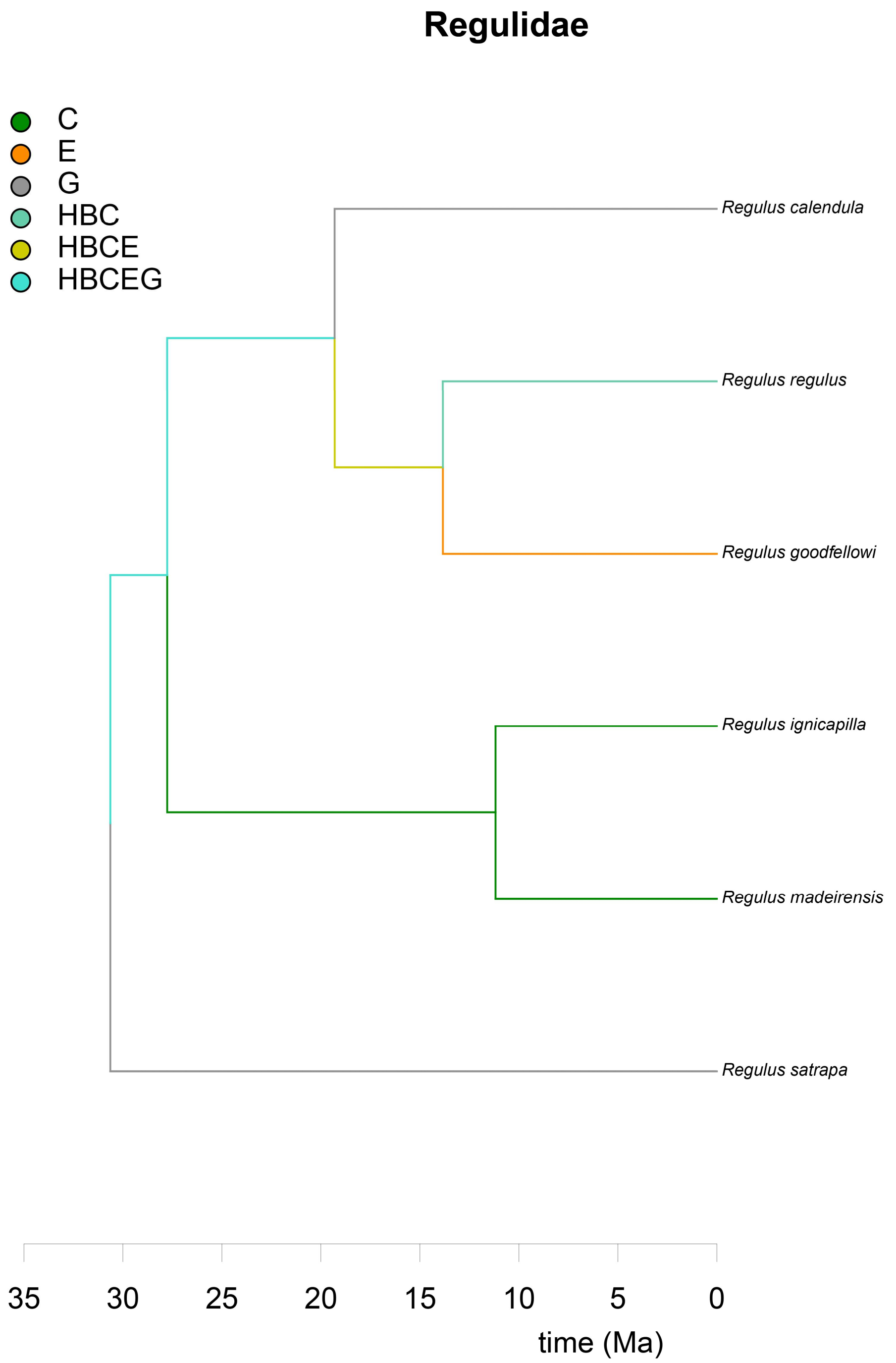

Fig. S92. Marginal maximum *a posteriori* reconstruction of the evolutionary history of geographic range on the maximum clade credibility tree of Regulidae using RevBayes. Labels for geographic regions follow Fig. S2.

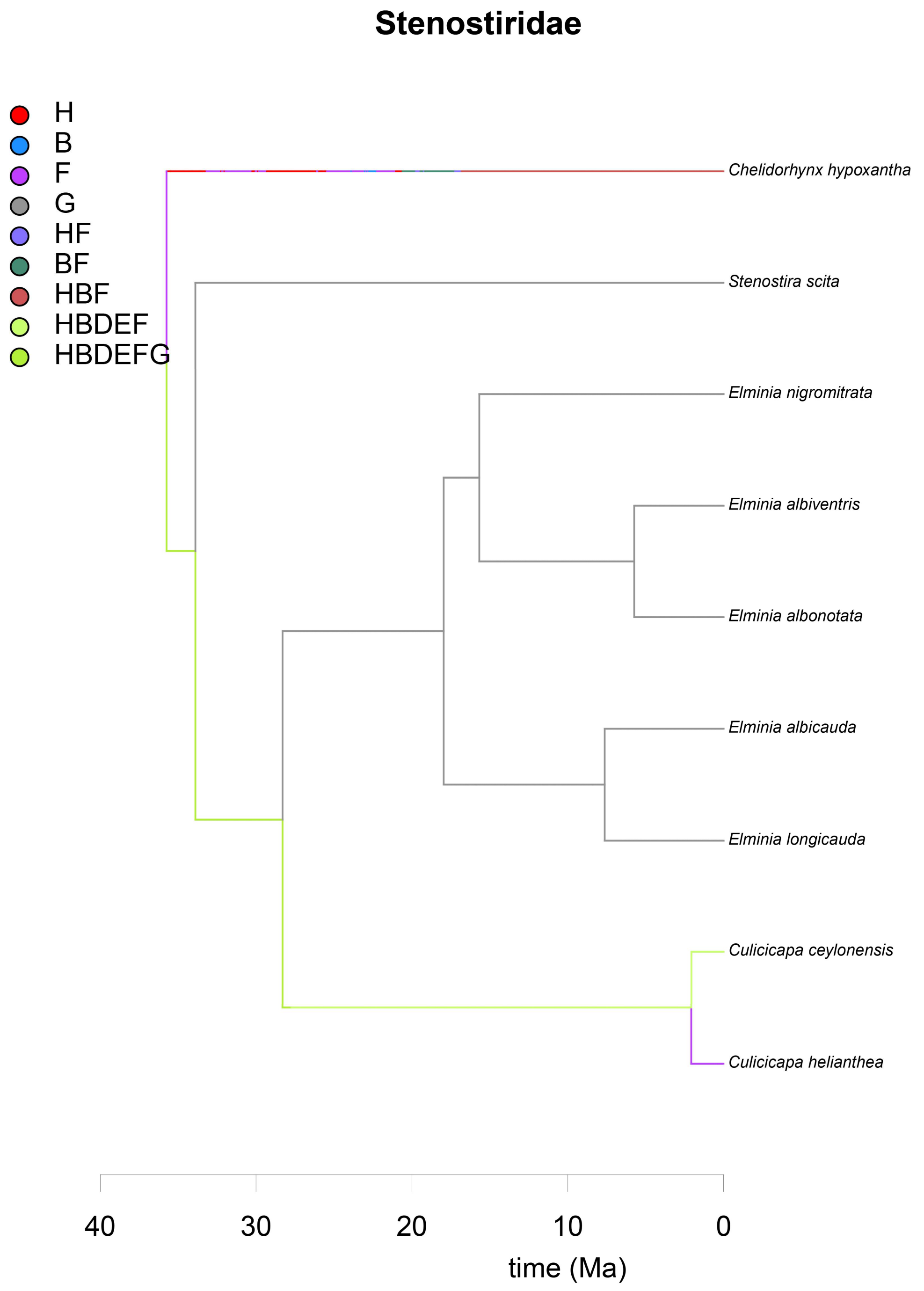

Fig. S93. Marginal maximum *a posteriori* reconstruction of the evolutionary history of geographic range on the maximum clade credibility tree of Stenostiridae using RevBayes. Labels for geographic regions follow Fig. S2.

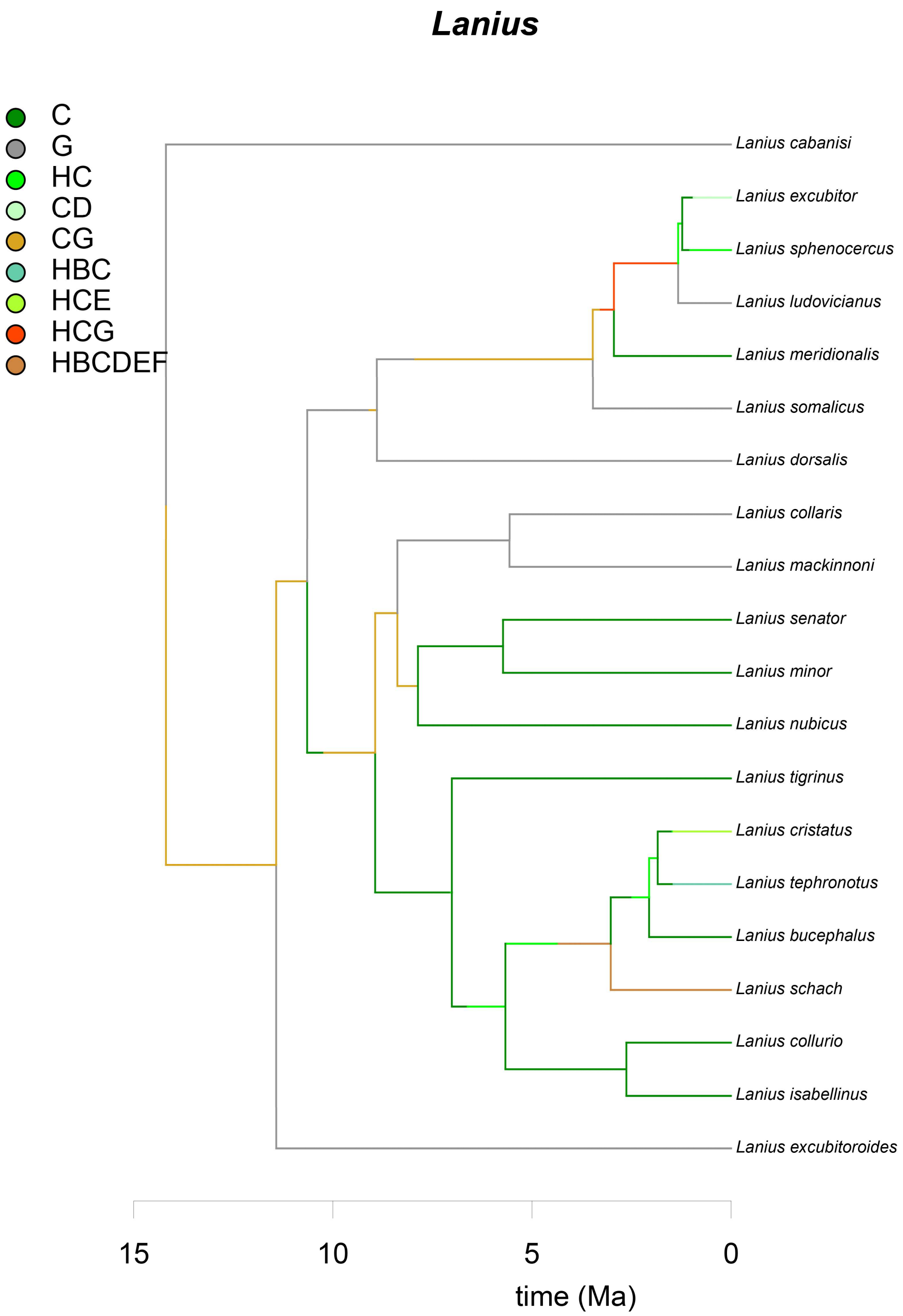

Fig. S94. Marginal maximum *a posteriori* reconstruction of the evolutionary history of geographic range on the maximum clade credibility tree of *Lanius* using RevBayes. Labels for geographic regions follow Fig. S2.

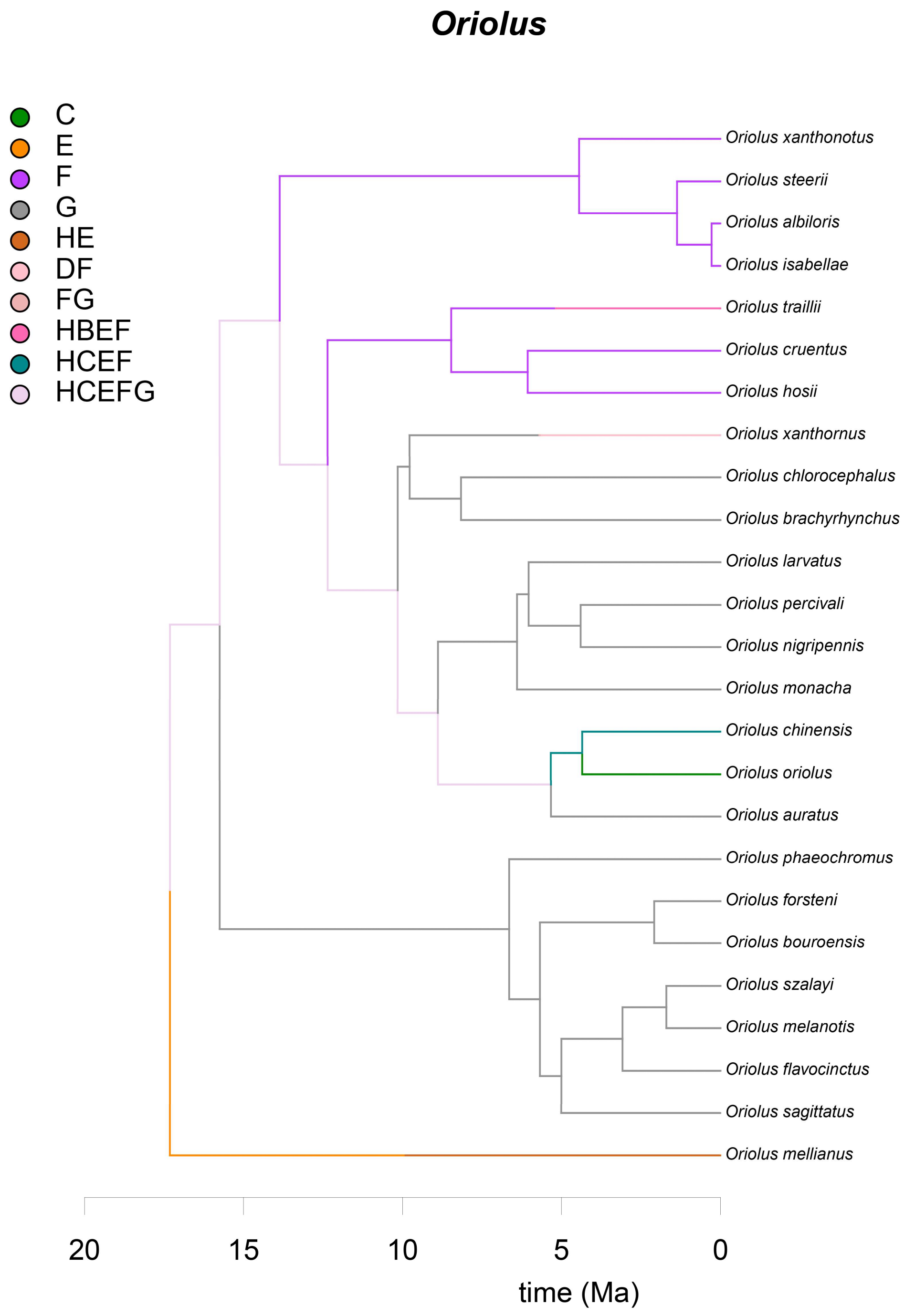

Fig. S95. Marginal maximum *a posteriori* reconstruction of the evolutionary history of geographic range on the maximum clade credibility tree of *Oriolus* using RevBayes. Labels for geographic regions follow Fig. S2.

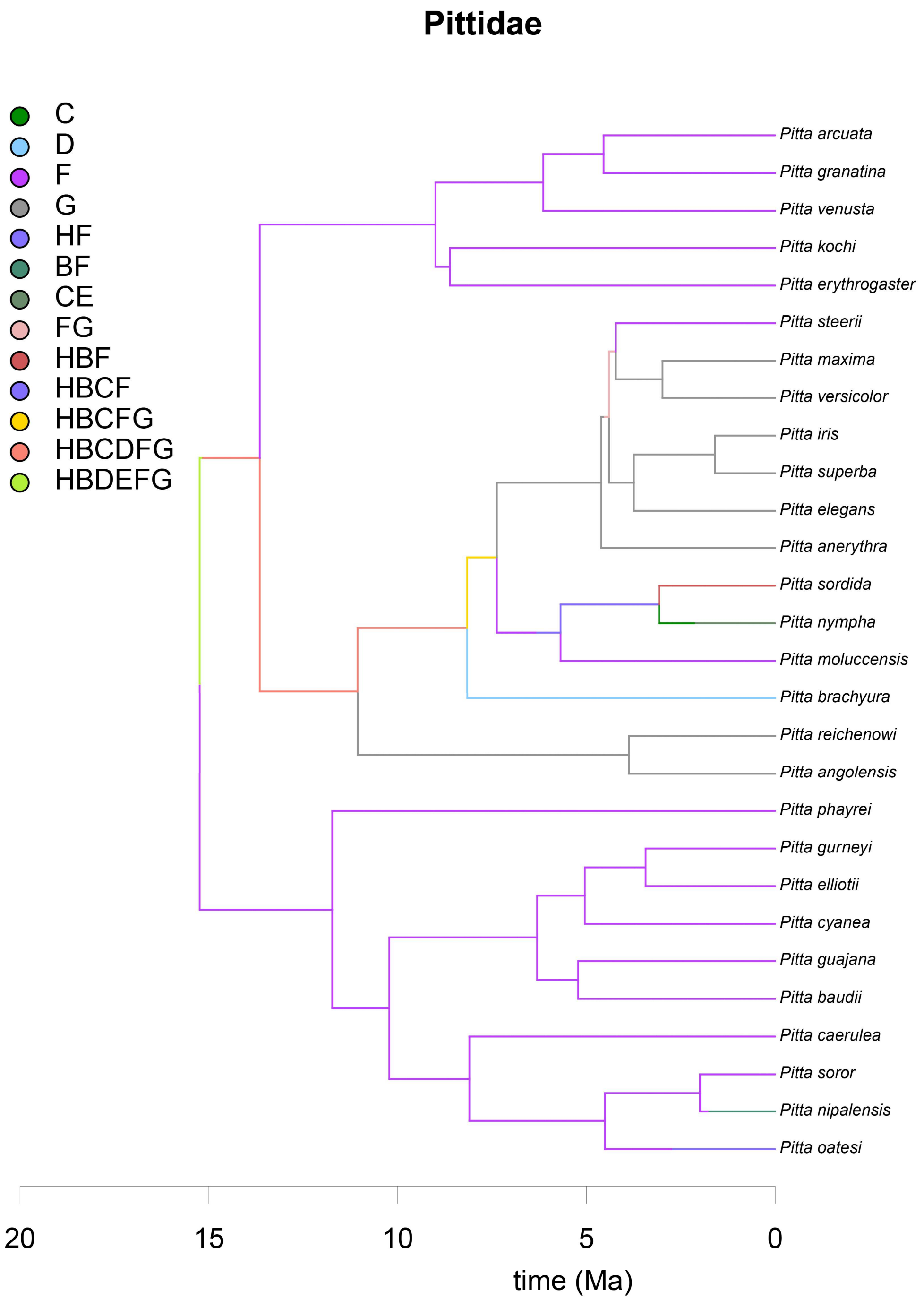

Fig. S96. Marginal maximum *a posteriori* reconstruction of the evolutionary history of geographic range on the maximum clade credibility tree of Pittidae using RevBayes. Labels for geographic regions follow Fig. S2.

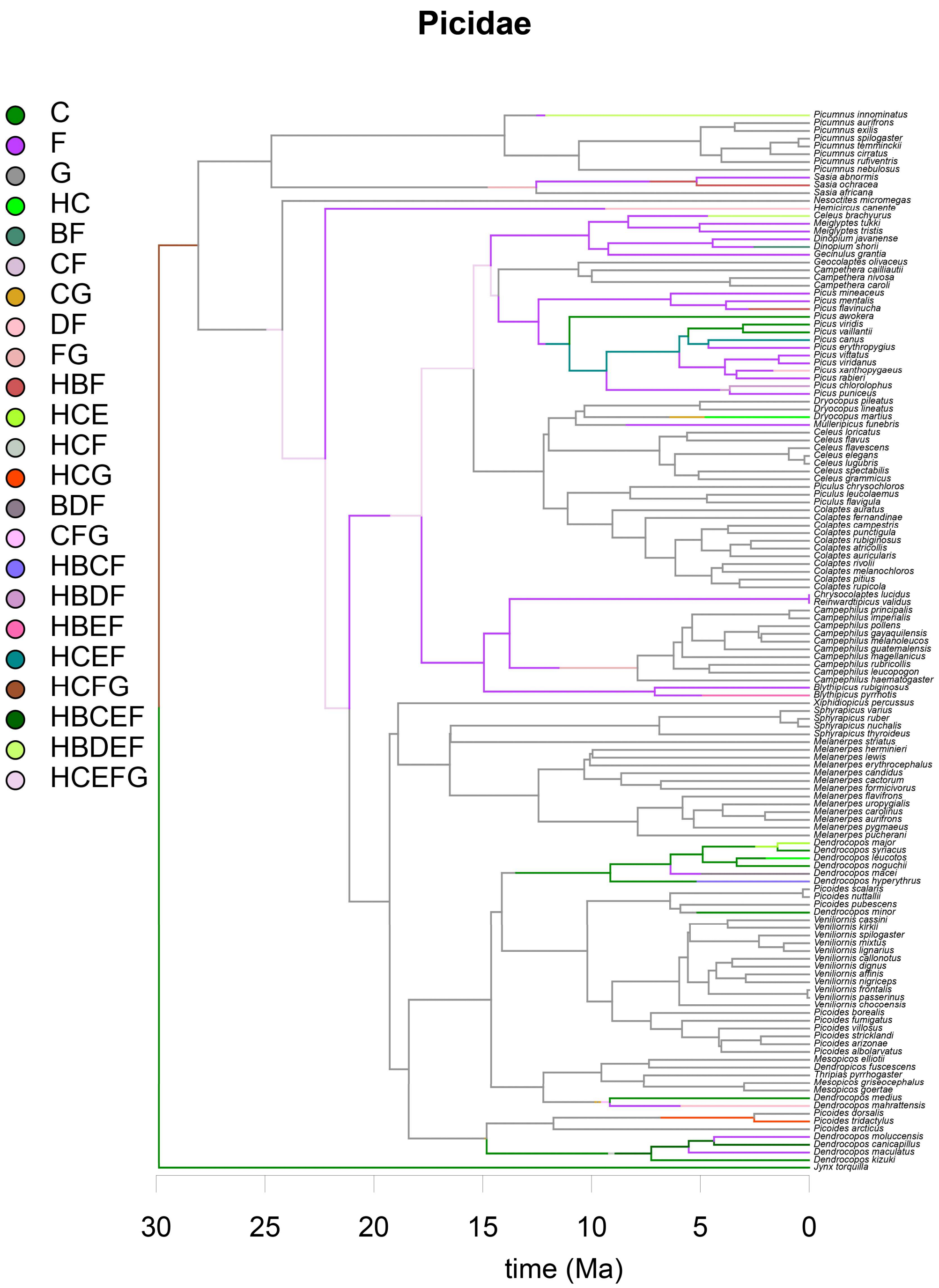

Fig. S97. Marginal maximum *a posteriori* reconstruction of the evolutionary history of geographic range on the maximum clade credibility tree of Picidae using RevBayes. Labels for geographic regions follow Fig. S2.

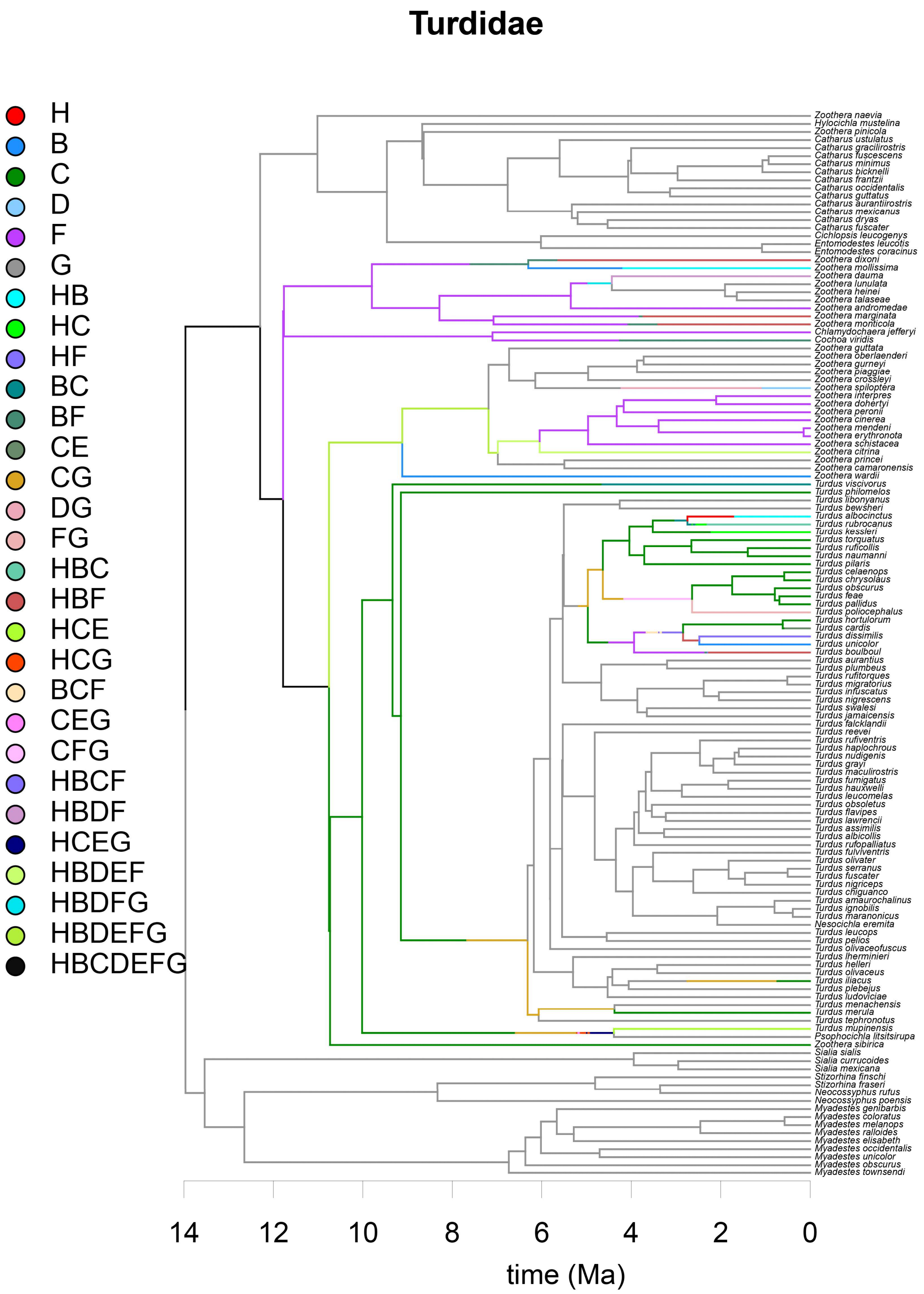

Fig. S98. Marginal maximum *a posteriori* reconstruction of the evolutionary history of geographic range on the maximum clade credibility tree of Turdidae using RevBayes. Labels for geographic regions follow Fig. S2.

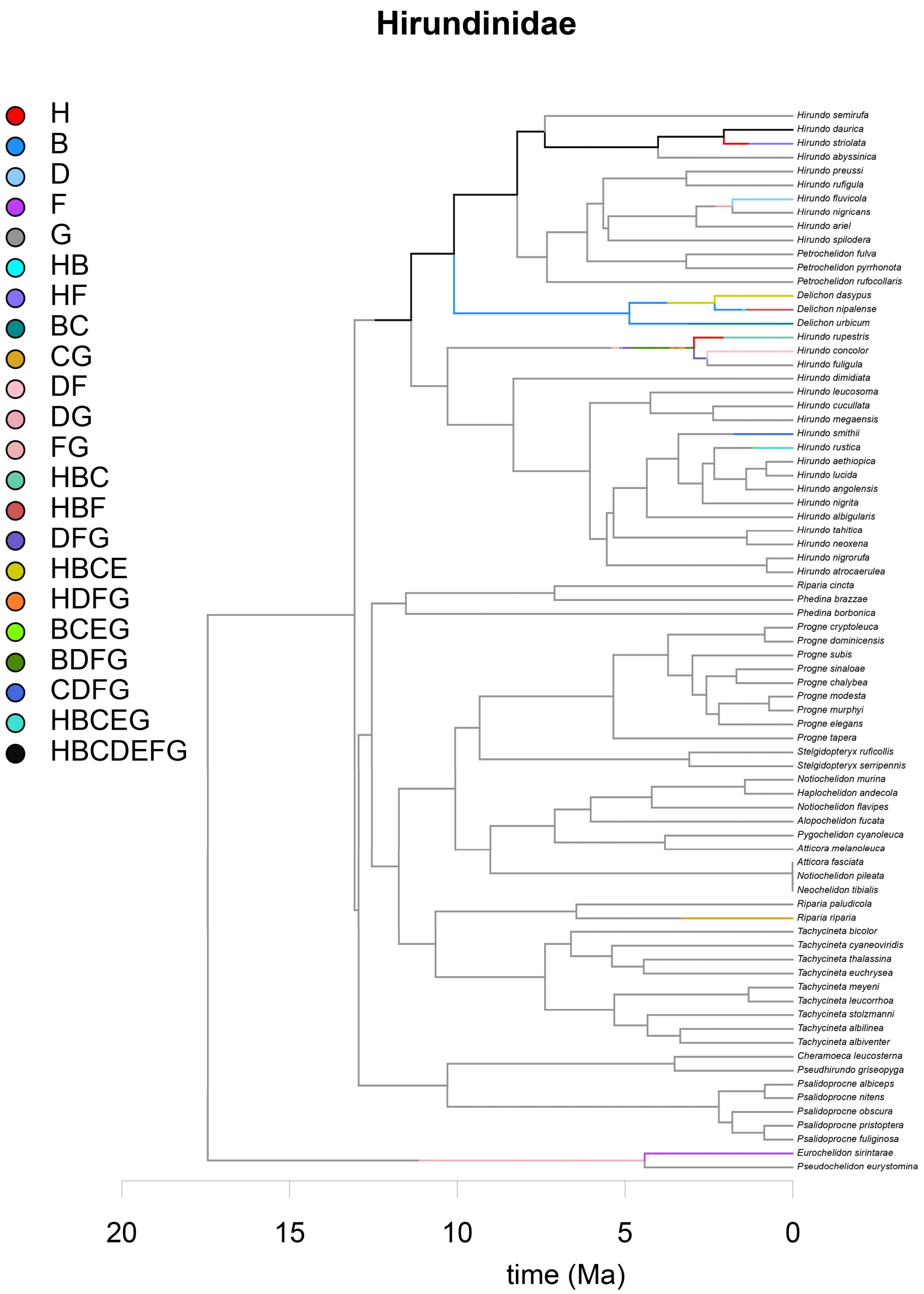

Fig. S99. Marginal maximum *a posteriori* reconstruction of the evolutionary history of geographic range on the maximum clade credibility tree of Hirundinidae using RevBayes. Labels for geographic regions follow Fig. S2.

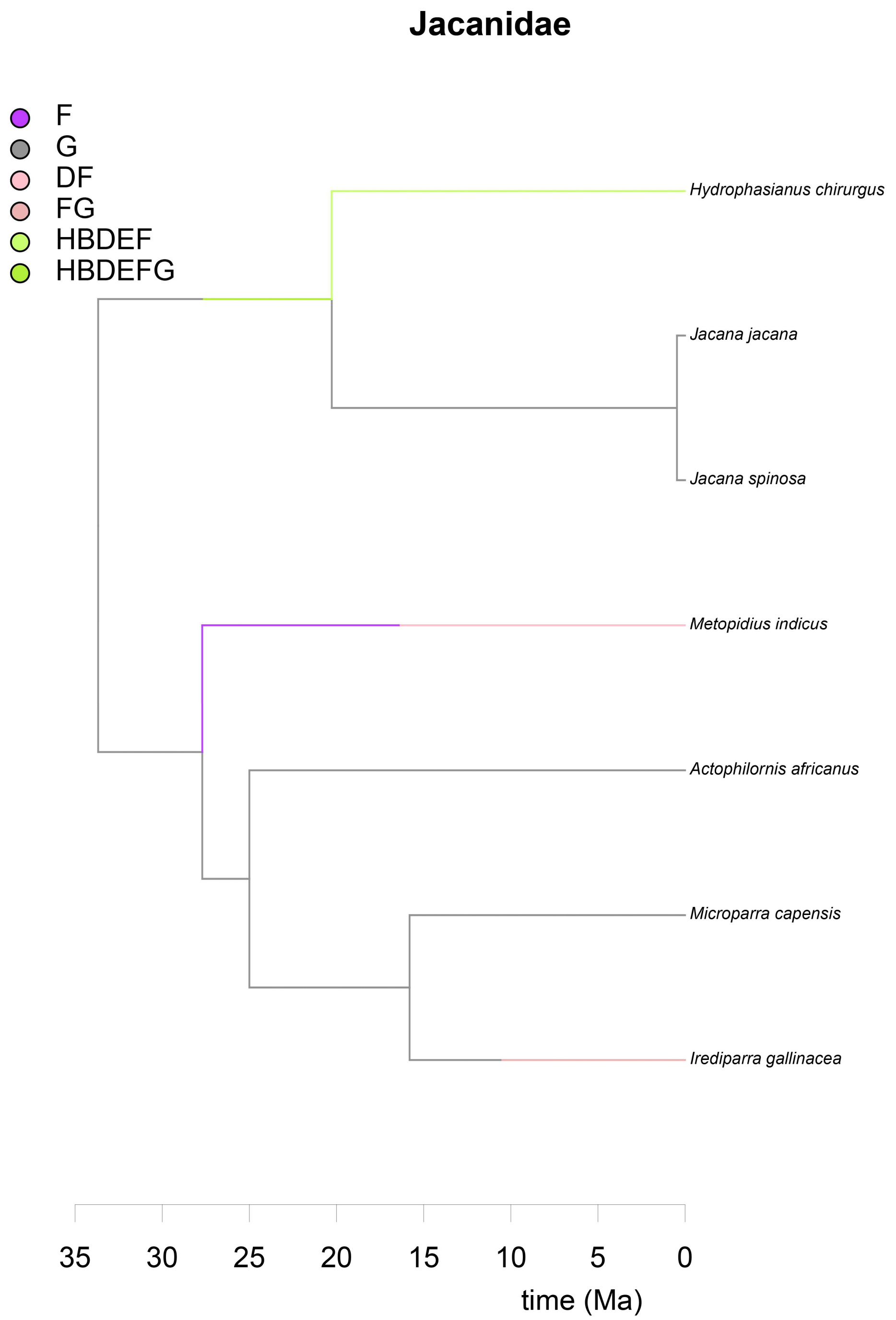

Fig. S100. Marginal maximum *a posteriori* reconstruction of the evolutionary history of geographic range on the maximum clade credibility tree of Jacanidae using RevBayes. Labels for geographic regions follow Fig. S2.

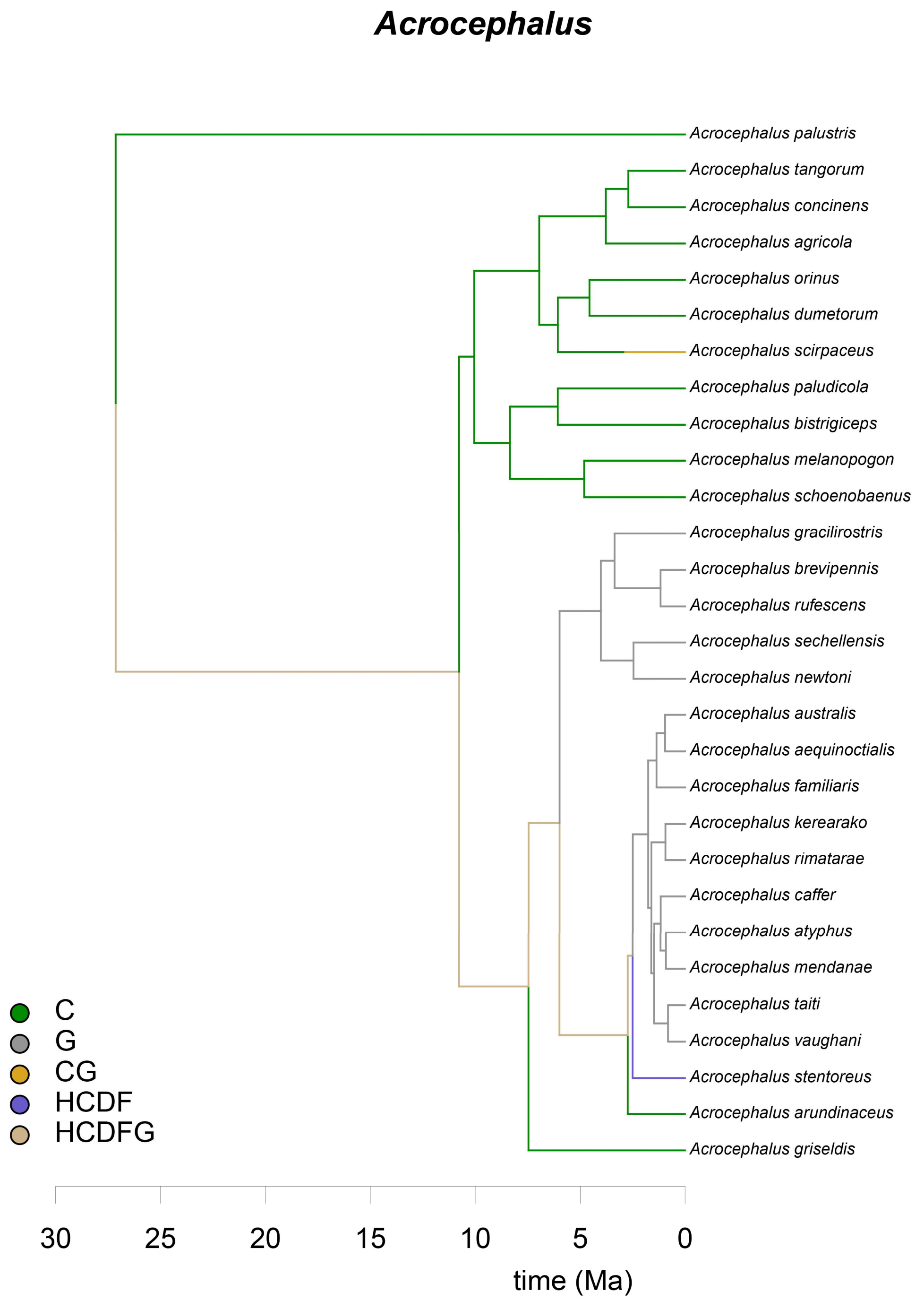

Fig. S101. Marginal maximum *a posteriori* reconstruction of the evolutionary history of geographic range on the maximum clade credibility tree of *Acrocephalus* using RevBayes. Labels for geographic regions follow Fig. S2.

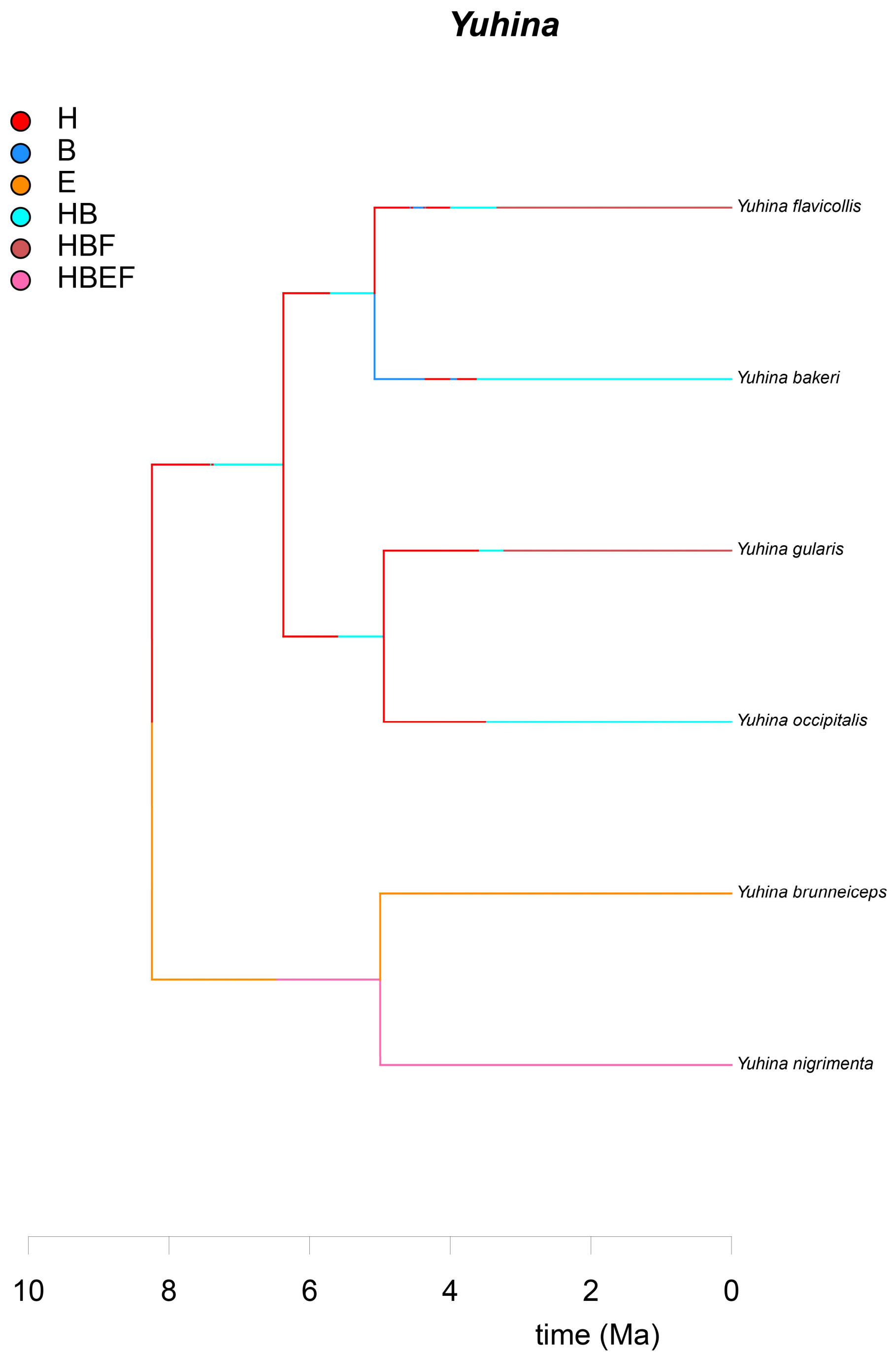

Fig. S102. Marginal maximum *a posteriori* reconstruction of the evolutionary history of geographic range on the maximum clade credibility tree of *Yuhina* using RevBayes. Labels for geographic regions follow Fig. S2.

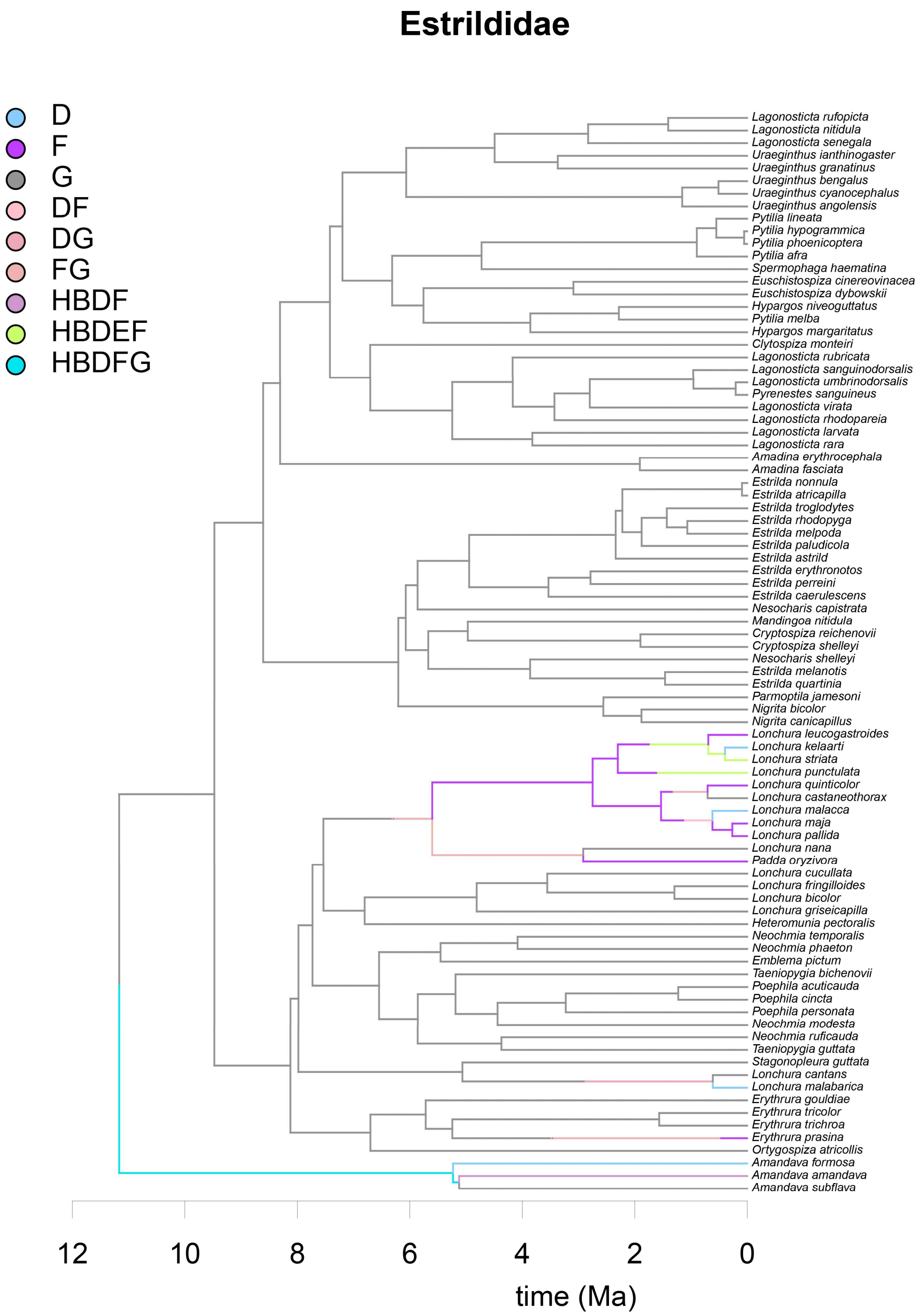

Fig. S103. Marginal maximum *a posteriori* reconstruction of the evolutionary history of geographic range on the maximum clade credibility tree of Estrildidae using RevBayes. Labels for geographic regions follow Fig. S2.

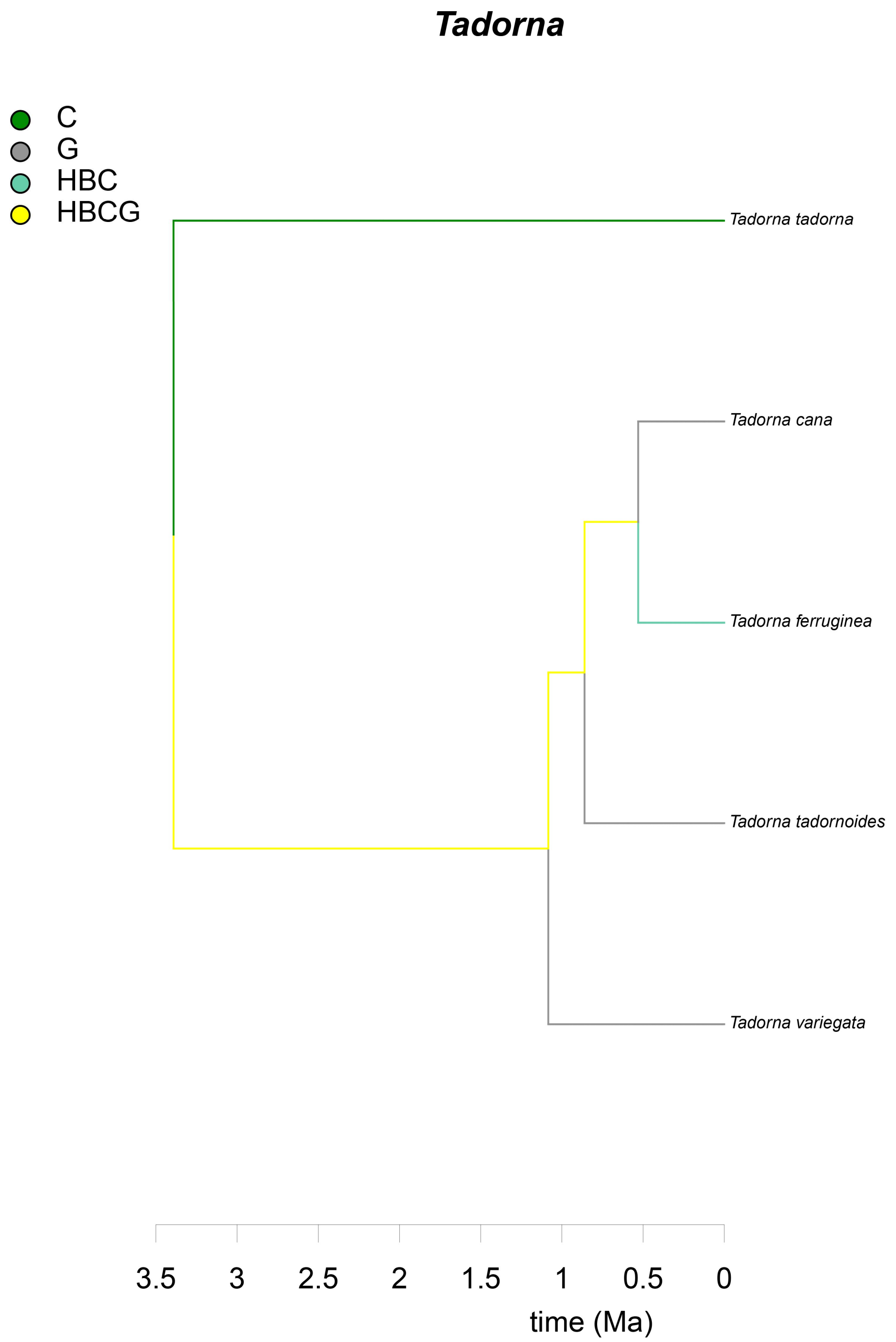

Fig. S104. Marginal maximum *a posteriori* reconstruction of the evolutionary history of geographic range on the maximum clade credibility tree of *Tadorna* using RevBayes. Labels for geographic regions follow Fig. S2.

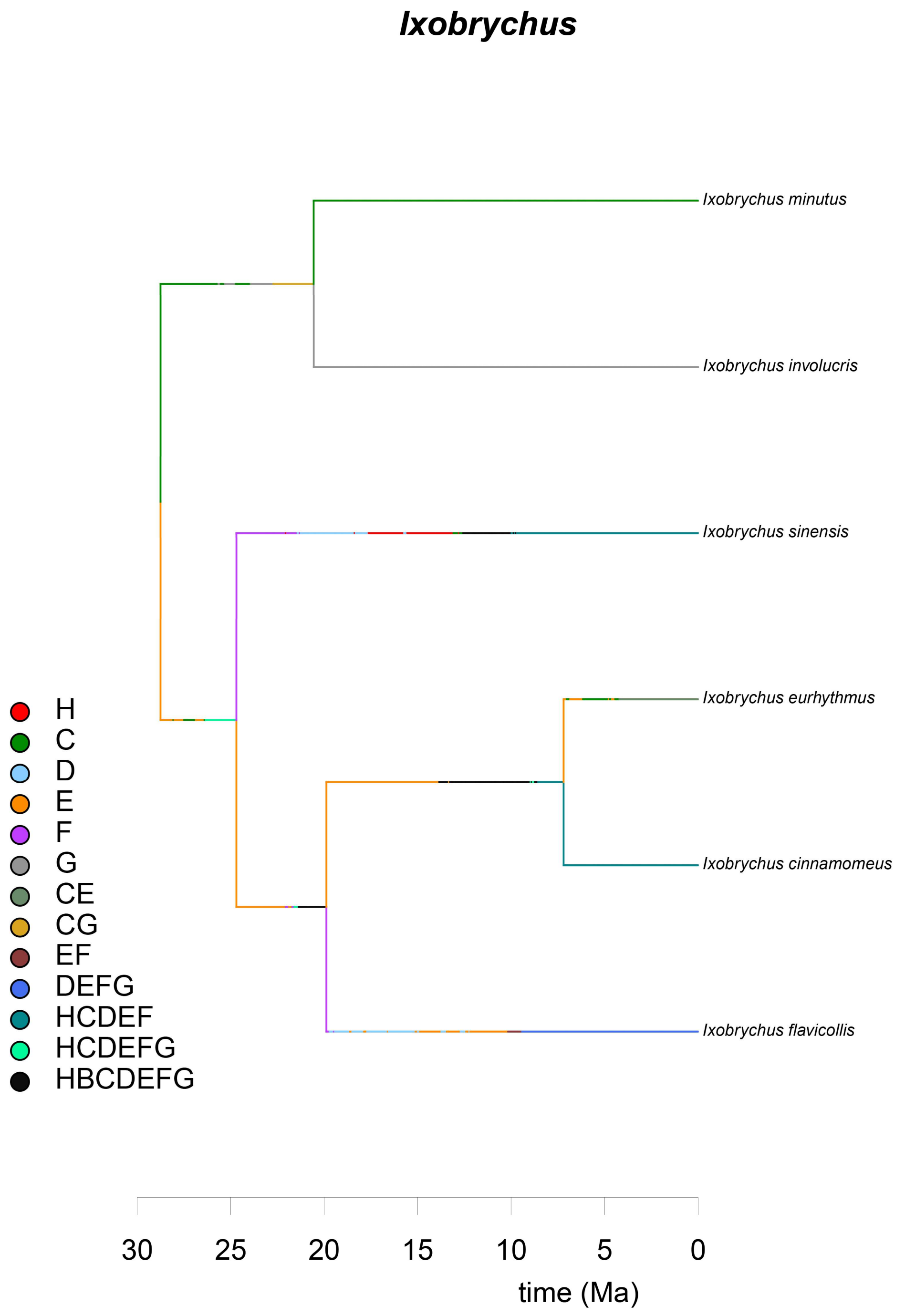

Fig. S105. Marginal maximum *a posteriori* reconstruction of the evolutionary history of geographic range on the maximum clade credibility tree of *Ixobrychus* using RevBayes. Labels for geographic regions follow Fig. S2.

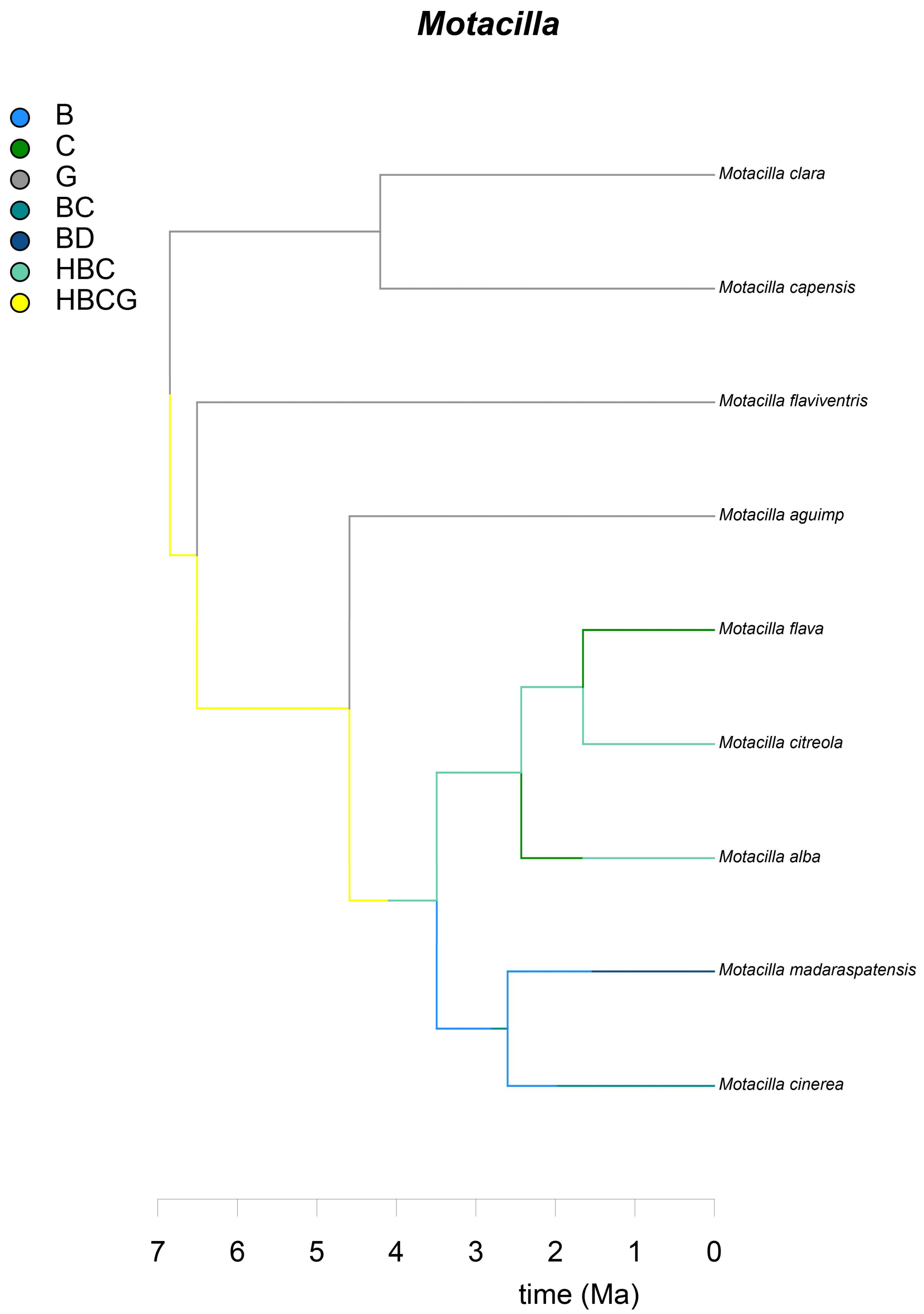

Fig. S106. Marginal maximum *a posteriori* reconstruction of the evolutionary history of geographic range on the maximum clade credibility tree of *Motacilla* using RevBayes. Labels for geographic regions follow Fig. S2.

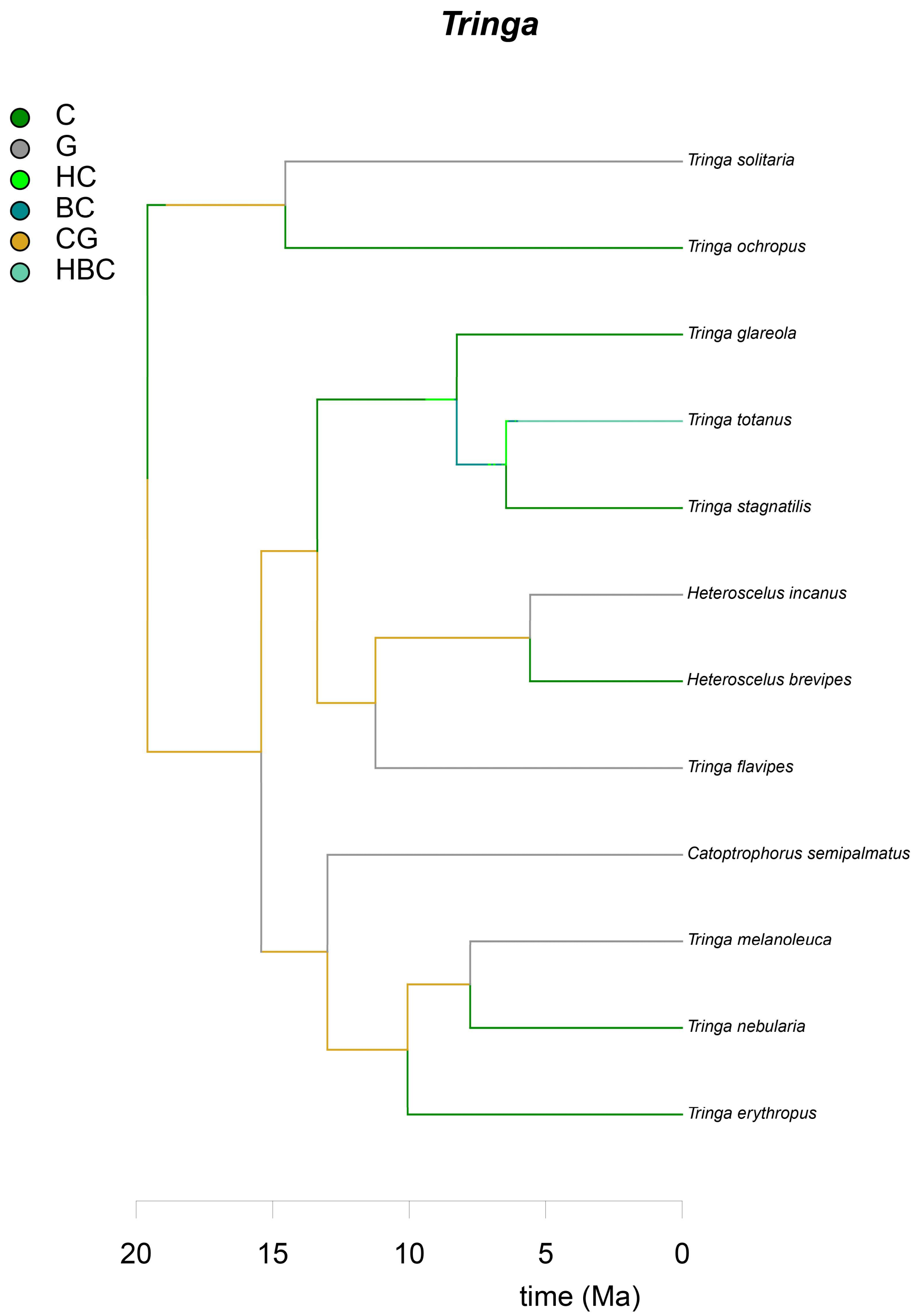

Fig. S107. Marginal maximum *a posteriori* reconstruction of the evolutionary history of geographic range on the maximum clade credibility tree of *Tringa* using RevBayes. Labels for geographic regions follow Fig. S2.

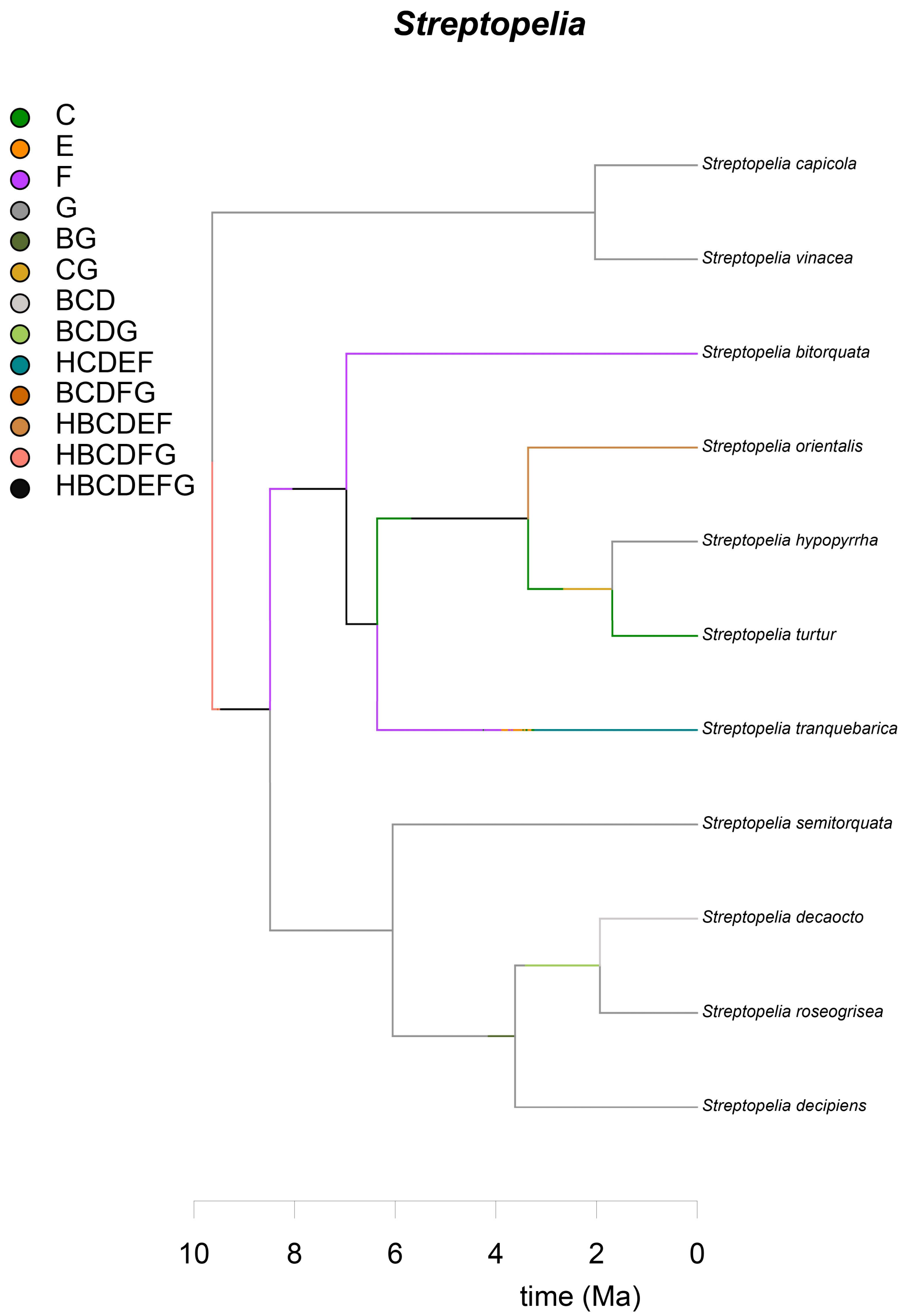

Fig. S108. Marginal maximum *a posteriori* reconstruction of the evolutionary history of geographic range on the maximum clade credibility tree of *Streptopelia* using RevBayes. Labels for geographic regions follow Fig. S2.

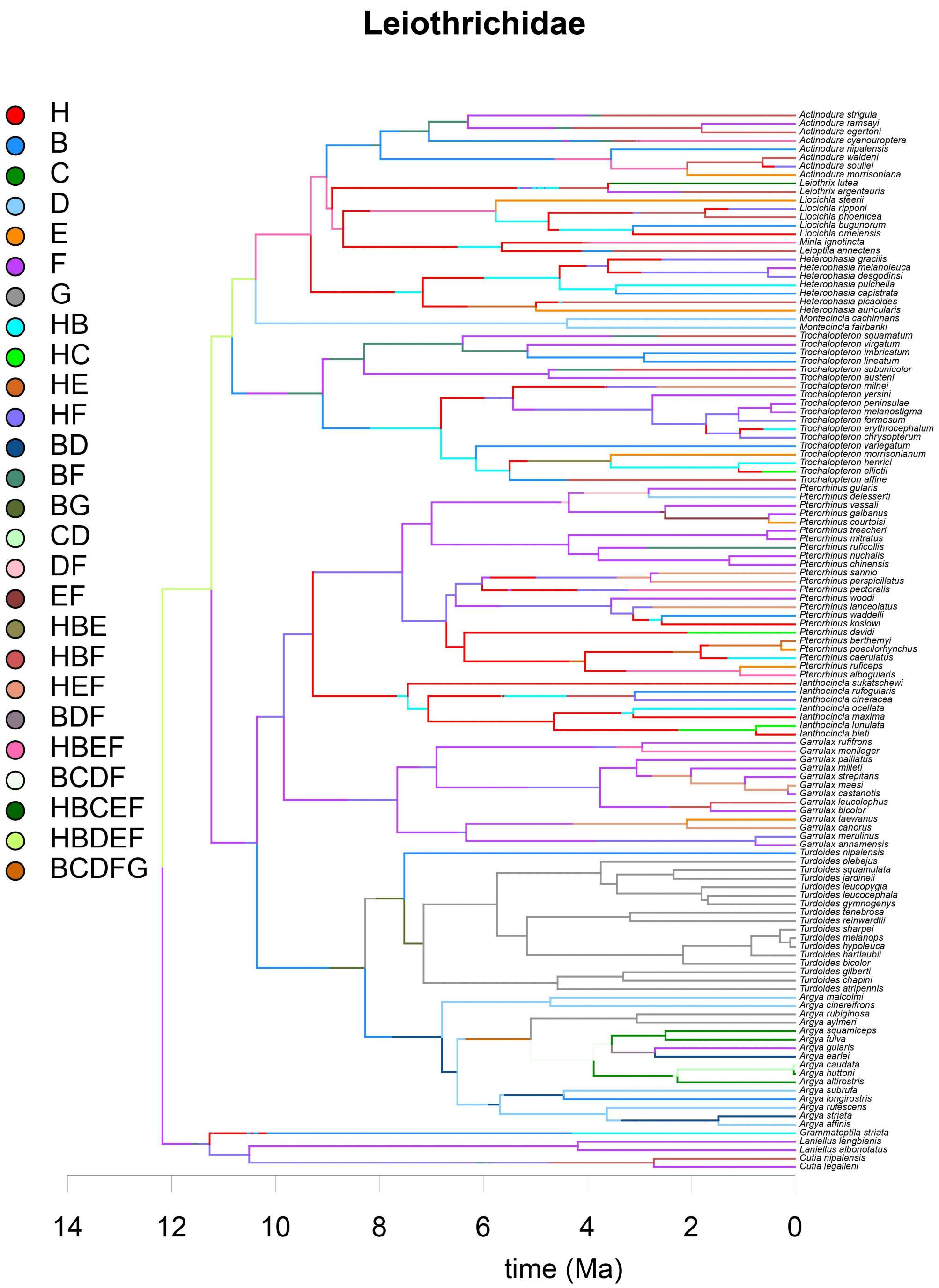

Fig. S109. Marginal maximum *a posteriori* reconstruction of the evolutionary history of geographic range on the maximum clade credibility tree of Leiothrichidae using RevBayes. Labels for geographic regions follow Fig. S2.

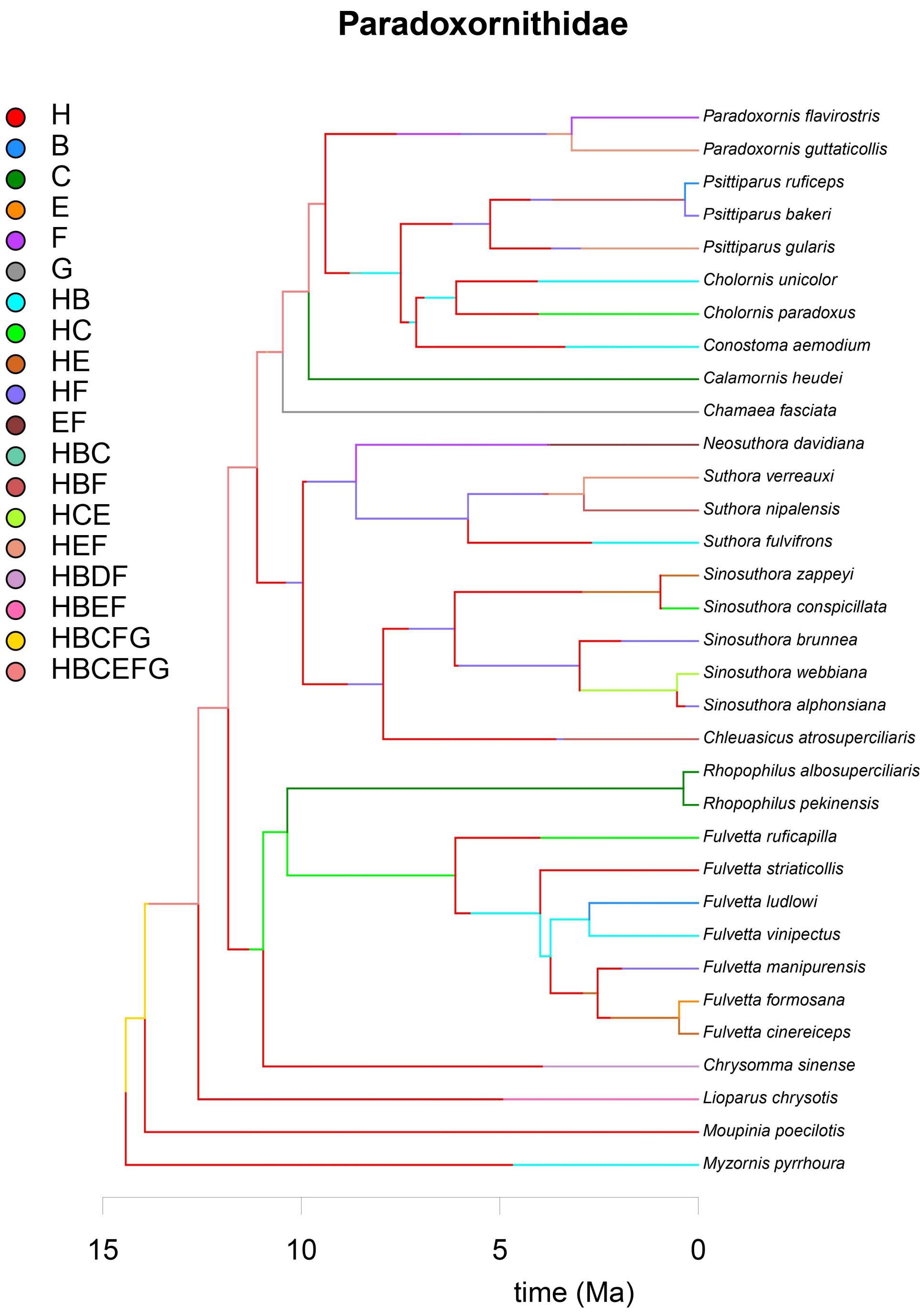

Fig. S110. Marginal maximum *a posteriori* reconstruction of the evolutionary history of geographic range on the maximum clade credibility tree of Paradoxornithidae using RevBayes. Labels for geographic regions follow Fig. S2.

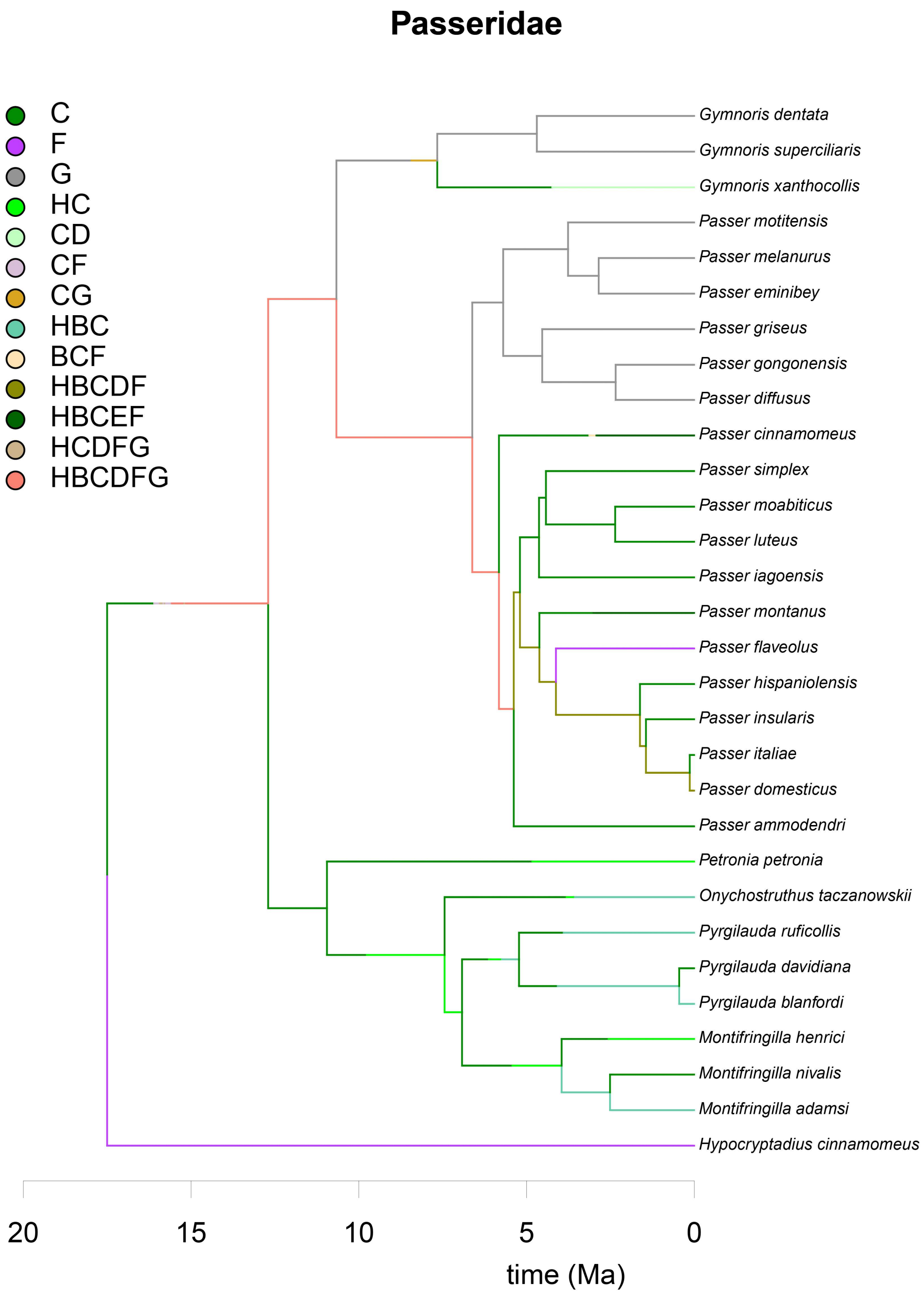

Fig. S111. Marginal maximum *a posteriori* reconstruction of the evolutionary history of geographic range on the maximum clade credibility tree of Passeridae using RevBayes. Labels for geographic regions follow Fig. S2.

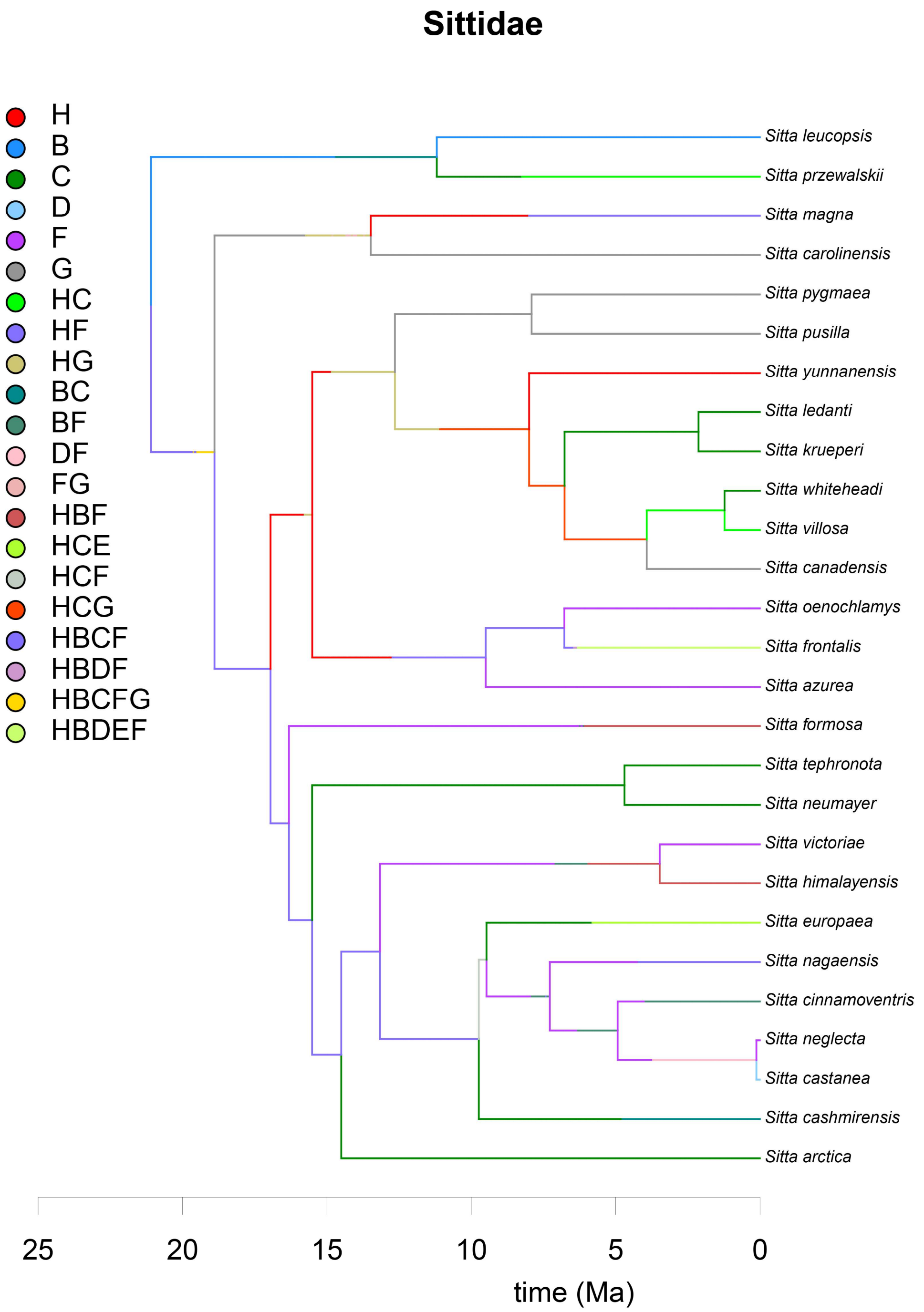

Fig. S112. Marginal maximum *a posteriori* reconstruction of the evolutionary history of geographic range on the maximum clade credibility tree of Sittidae using RevBayes. Labels for geographic regions follow Fig. S2.

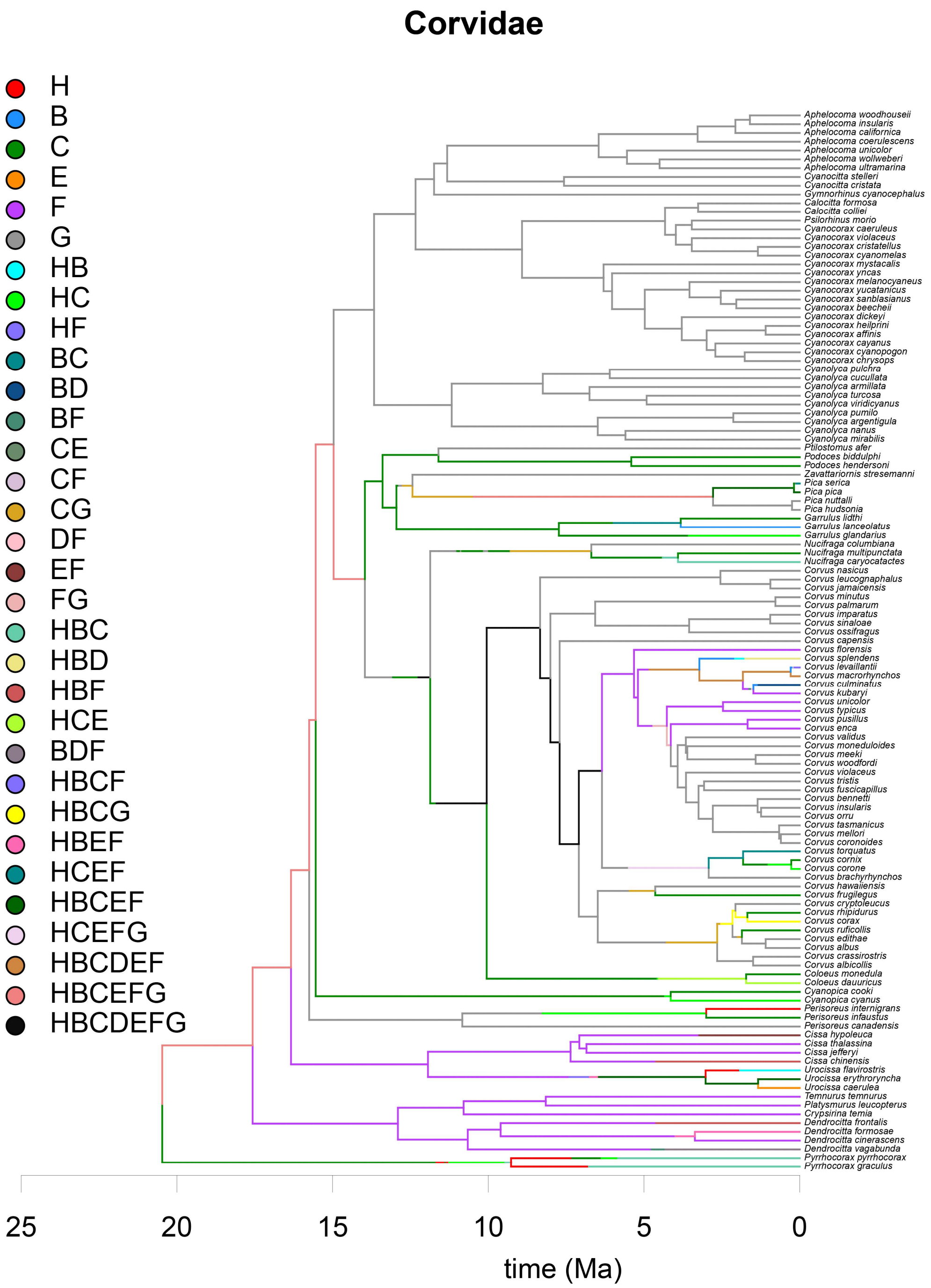

Fig. S113. Marginal maximum *a posteriori* reconstruction of the evolutionary history of geographic range on the maximum clade credibility tree of Corvidae using RevBayes. Labels for geographic regions follow Fig. S2.

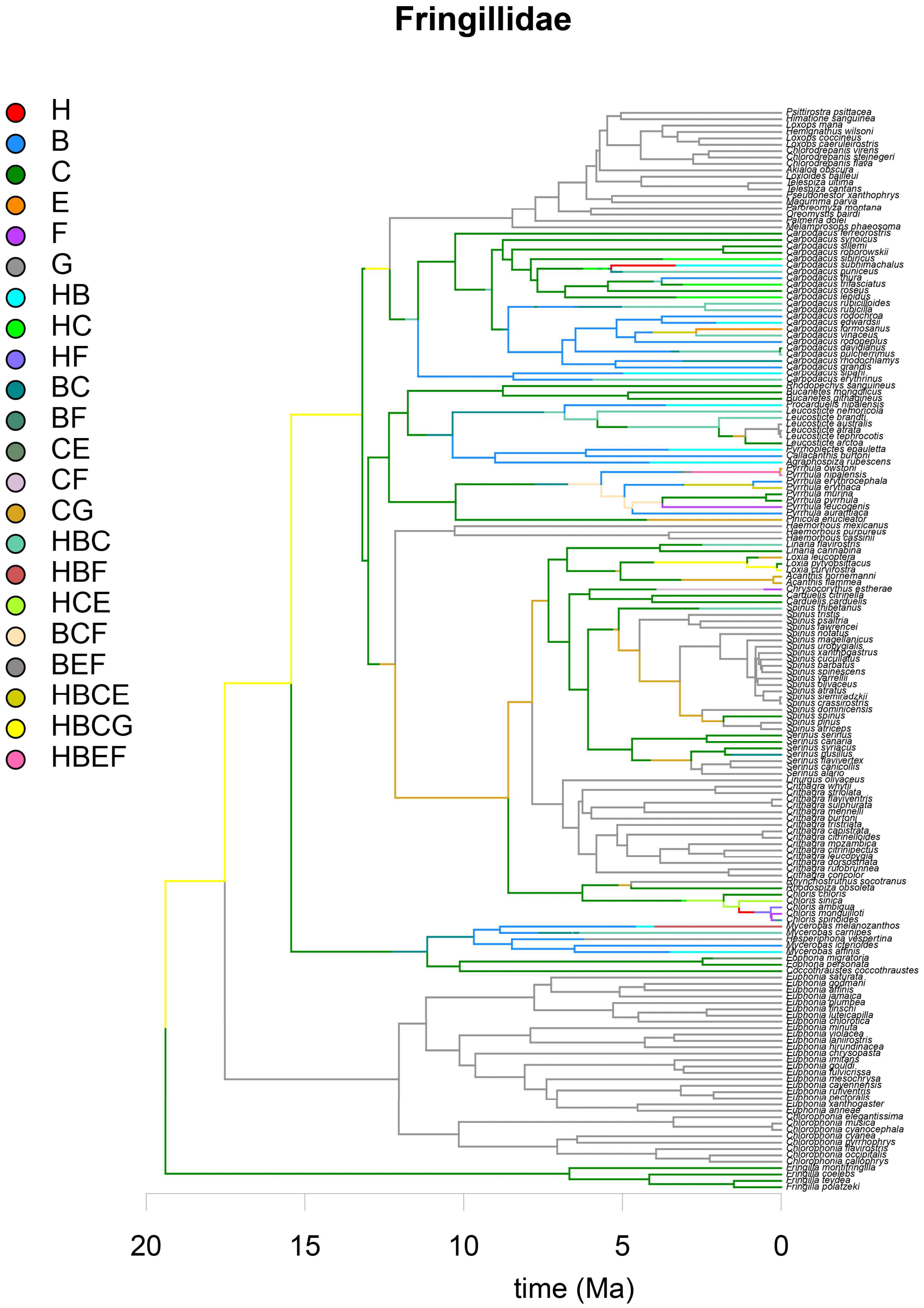

Fig. S114. Marginal maximum *a posteriori* reconstruction of the evolutionary history of geographic range on the maximum clade credibility tree of Fringillidae using RevBayes. Labels for geographic regions follow Fig. S2.

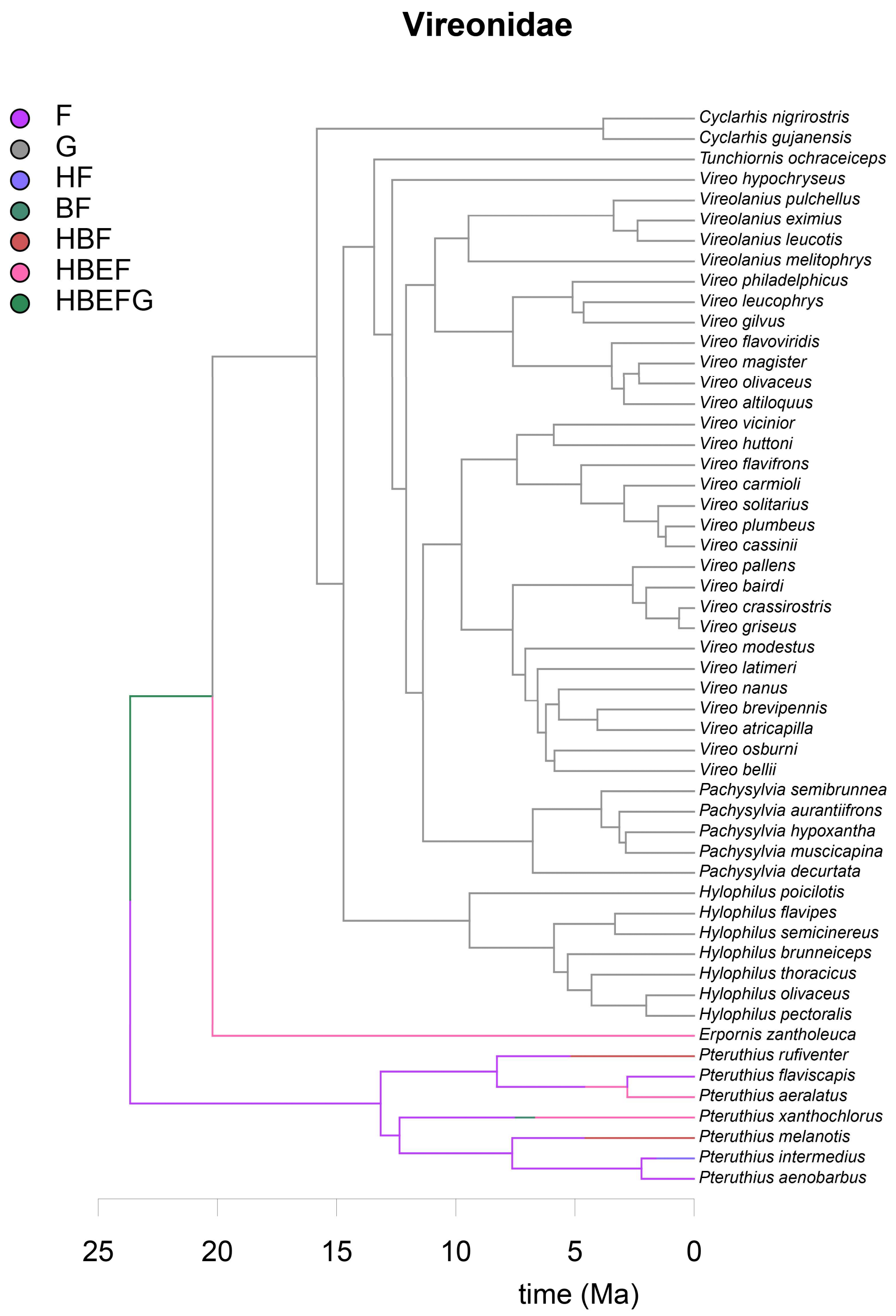

Fig. S115. Marginal maximum *a posteriori* reconstruction of the evolutionary history of geographic range on the maximum clade credibility tree of Vireonidae using RevBayes. Labels for geographic regions follow Fig. S2.

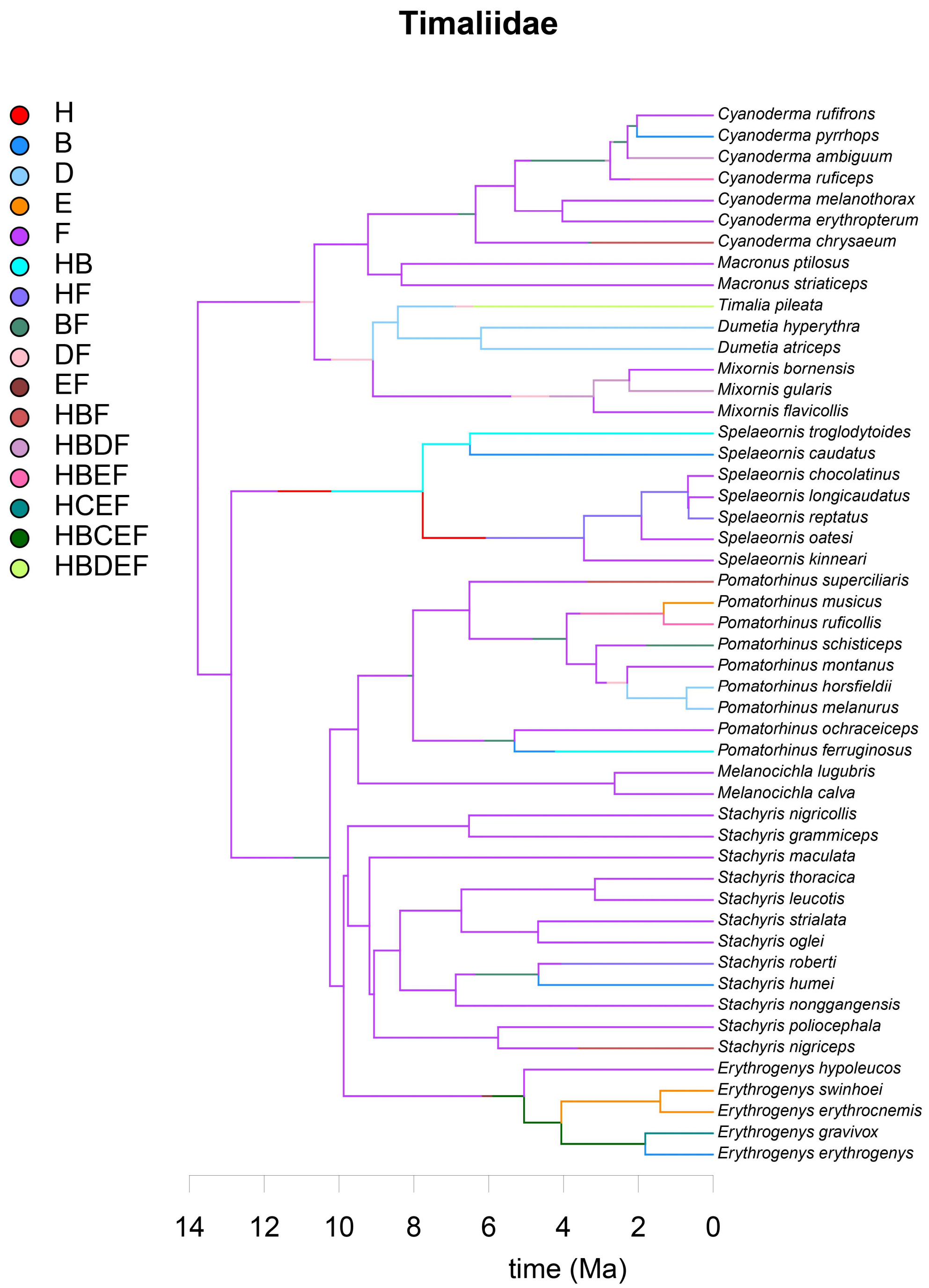

Fig. S116. Marginal maximum *a posteriori* reconstruction of the evolutionary history of geographic range on the maximum clade credibility tree of Timaliidae using RevBayes. Labels for geographic regions follow Fig. S2.

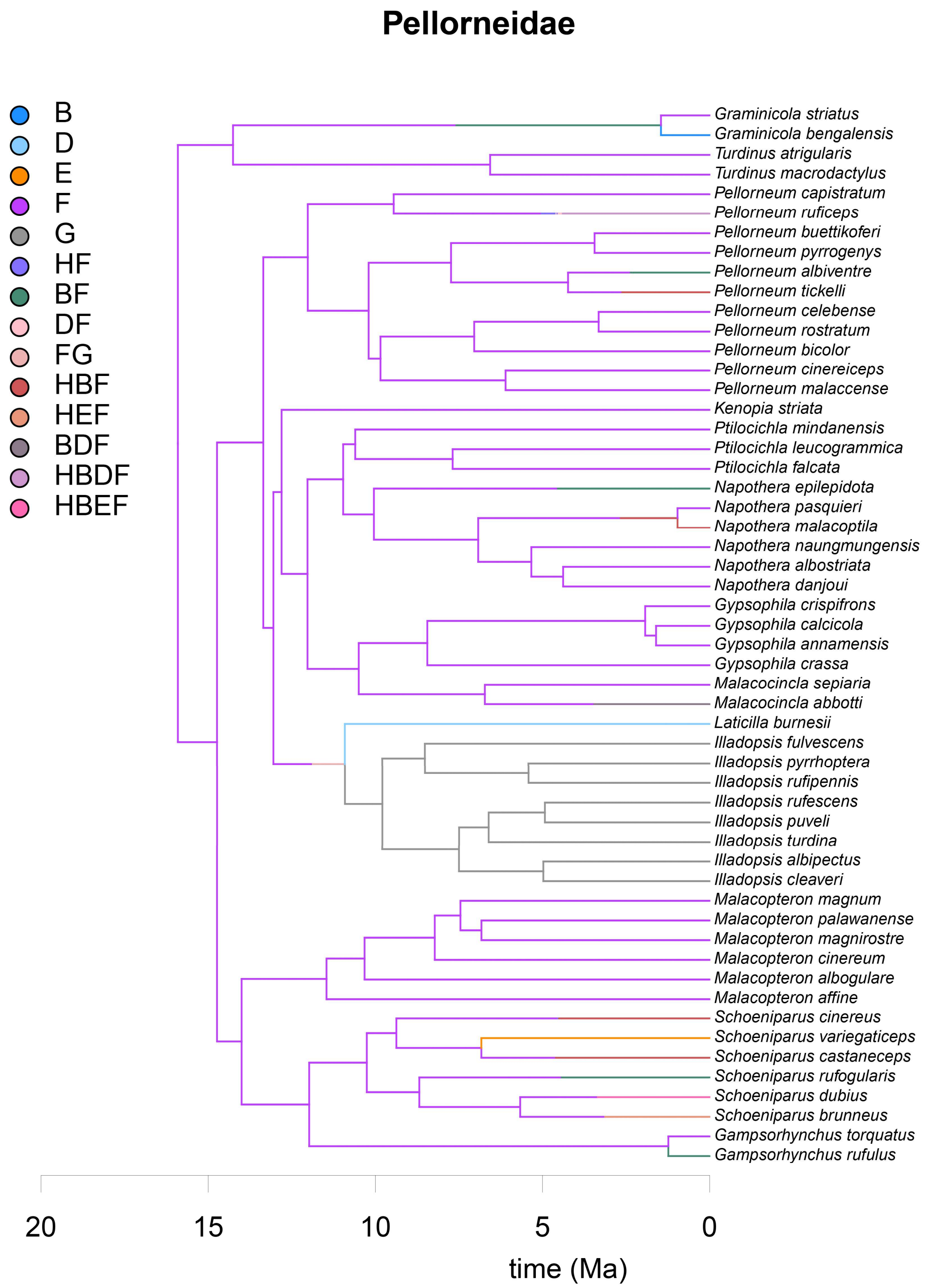

Fig. S117. Marginal maximum *a posteriori* reconstruction of the evolutionary history of geographic range on the maximum clade credibility tree of Pellorneidae using RevBayes. Labels for geographic regions follow Fig. S2.

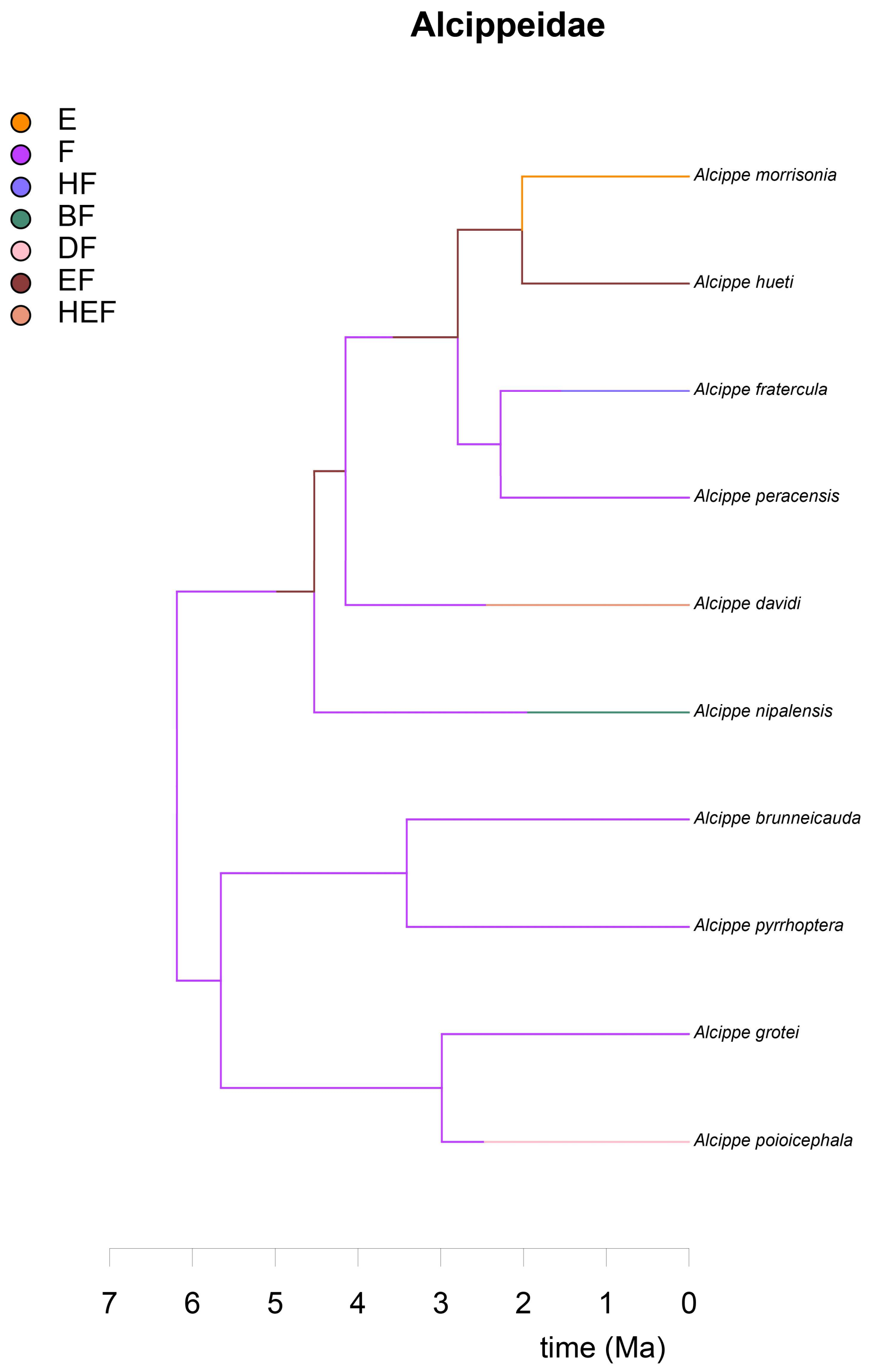

Fig. S118. Marginal maximum *a posteriori* reconstruction of the evolutionary history of geographic range on the maximum clade credibility tree of Alcippeidae using RevBayes. Labels for geographic regions follow Fig. S2.

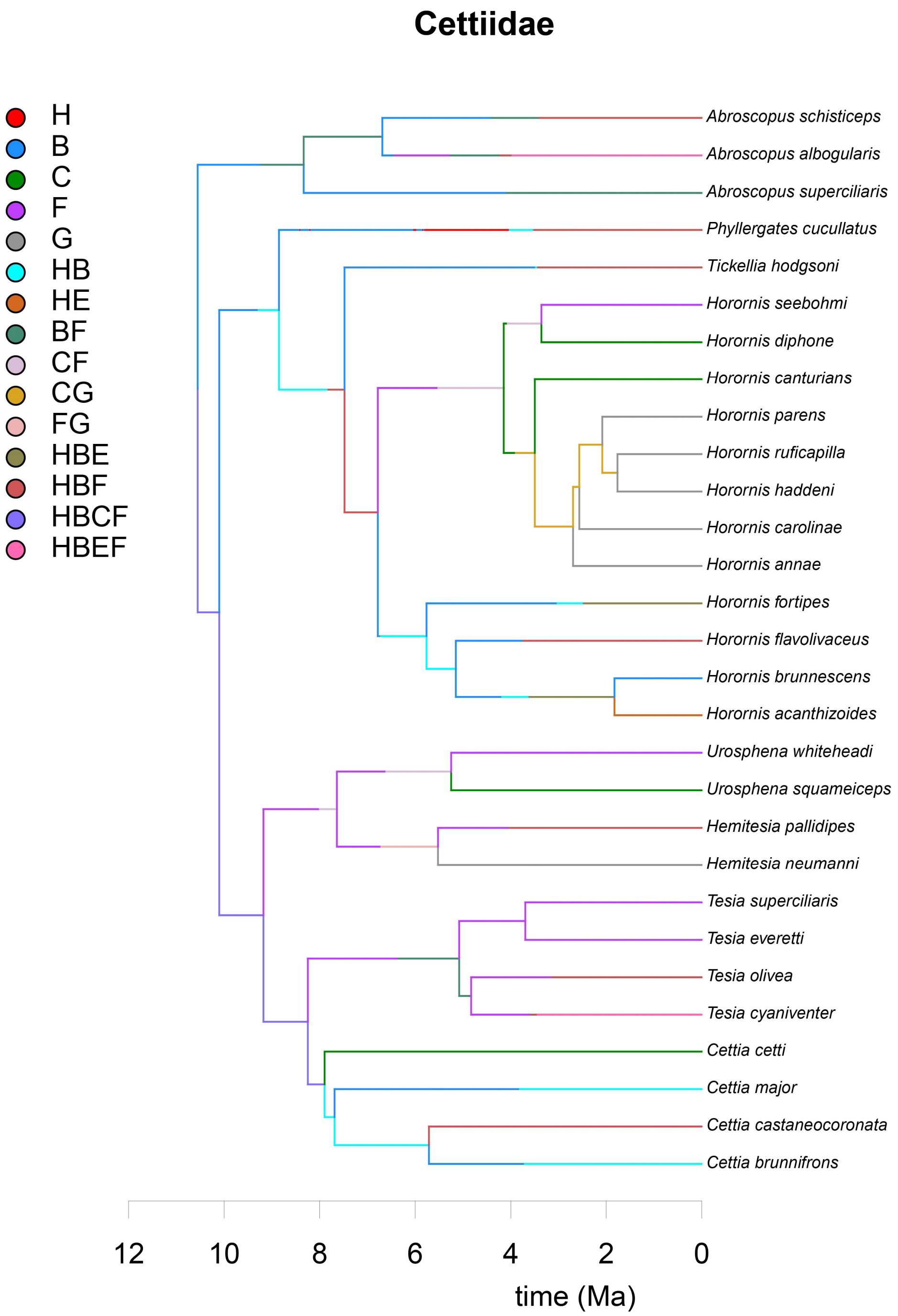

Fig. S119. Marginal maximum *a posteriori* reconstruction of the evolutionary history of geographic range on the maximum clade credibility tree of Cettiidae using RevBayes. Labels for geographic regions follow Fig. S2.

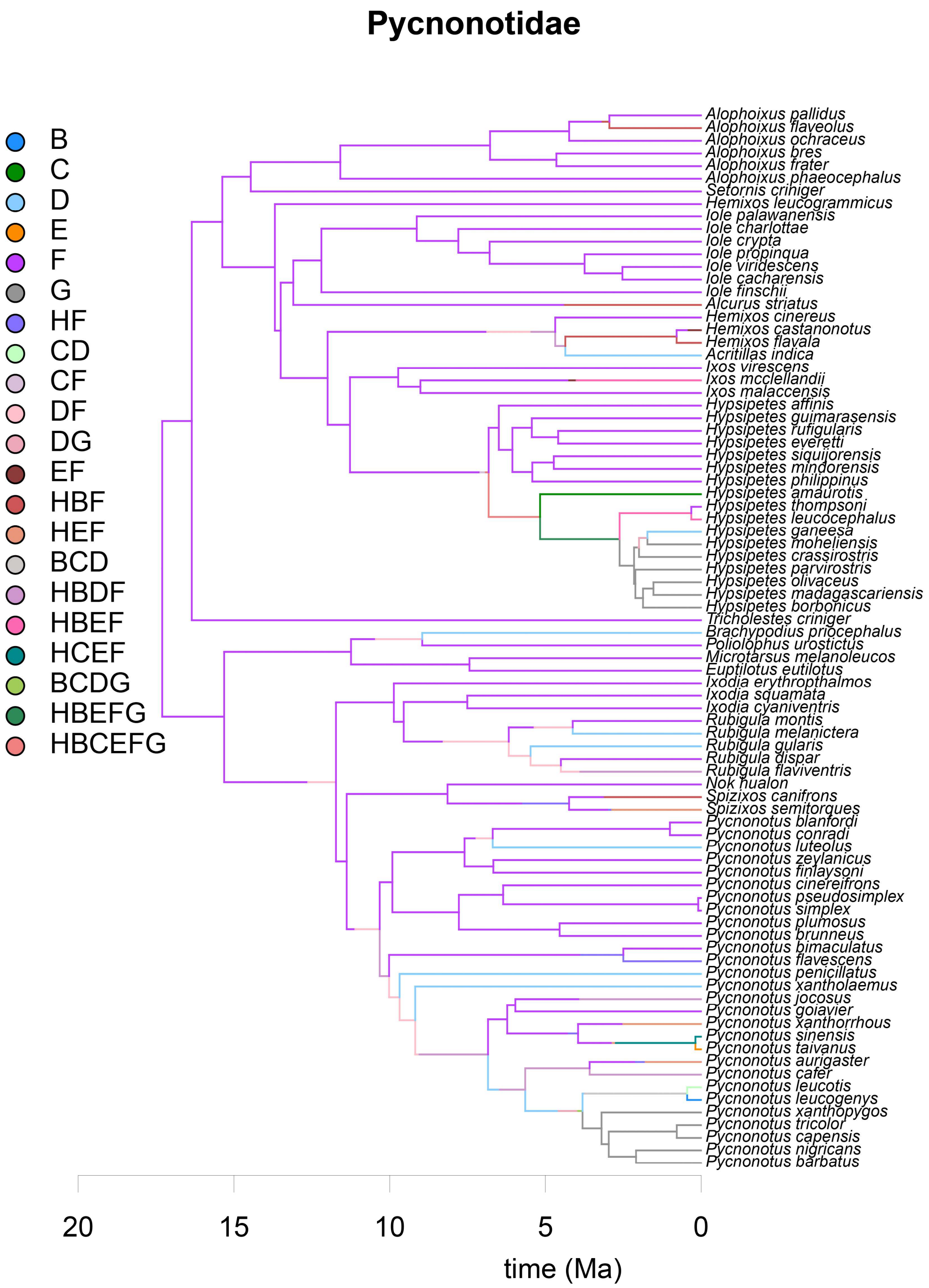

Fig. S120. Marginal maximum *a posteriori* reconstruction of the evolutionary history of geographic range on the maximum clade credibility tree of Pycnonotidae using RevBayes. Labels for geographic regions follow Fig. S2.

Locustellidae

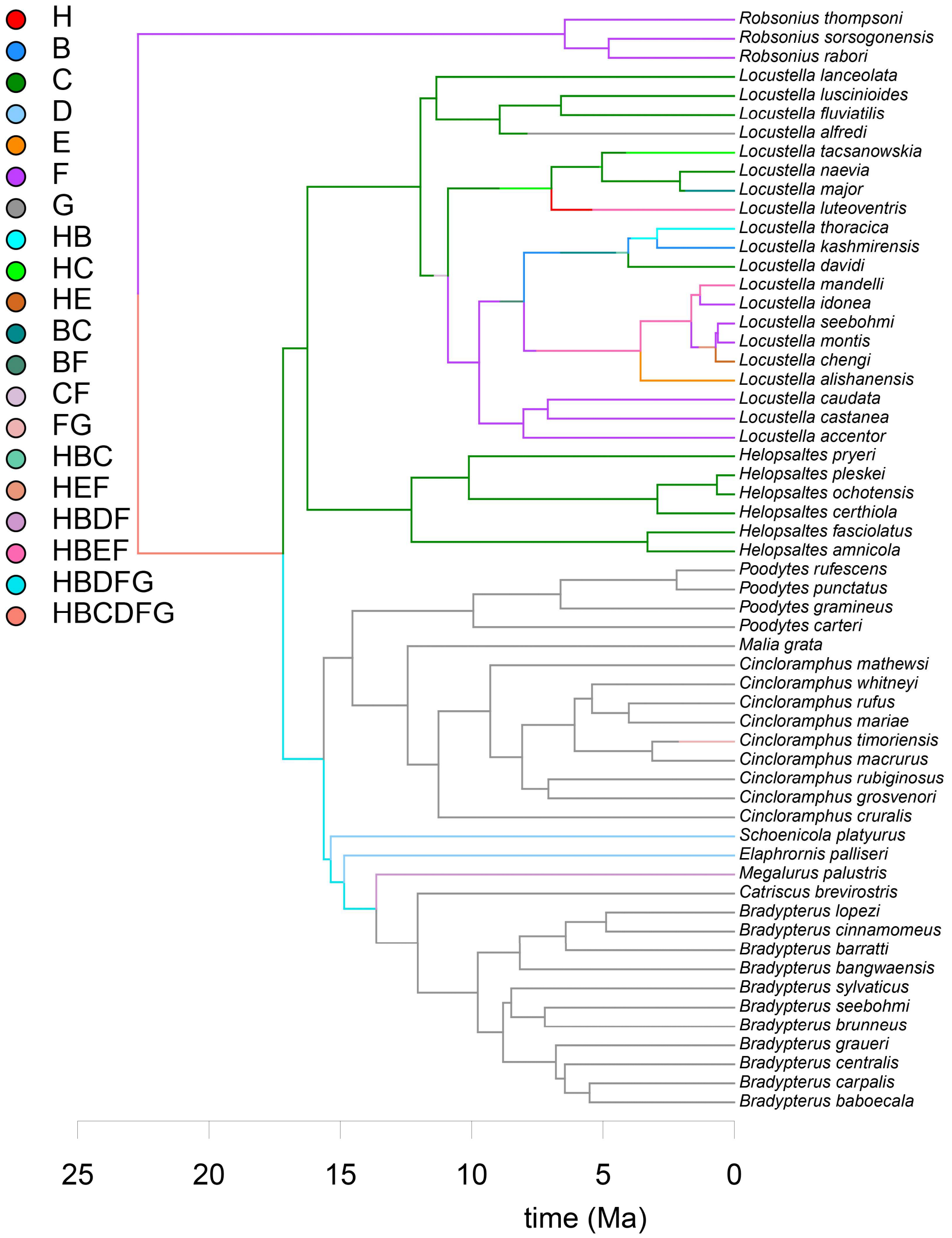

Fig. S121. Marginal maximum *a posteriori* reconstruction of the evolutionary history of geographic range on the maximum clade credibility tree of Locustellidae using RevBayes. Labels for geographic regions follow Fig. S2.

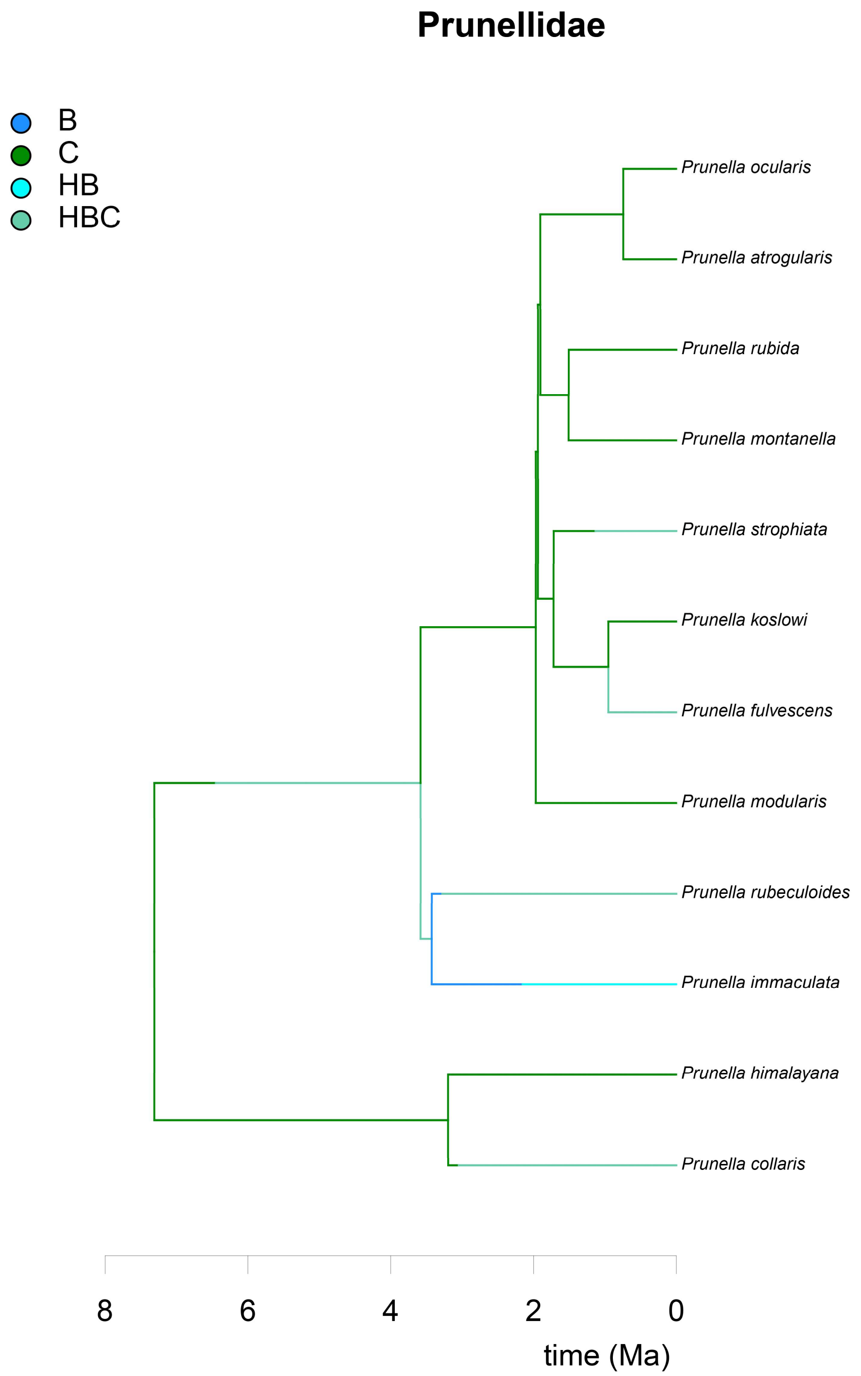

Fig. S122. Marginal maximum *a posteriori* reconstruction of the evolutionary history of geographic range on the maximum clade credibility tree of Prunellidae using RevBayes. Labels for geographic regions follow Fig. S2.

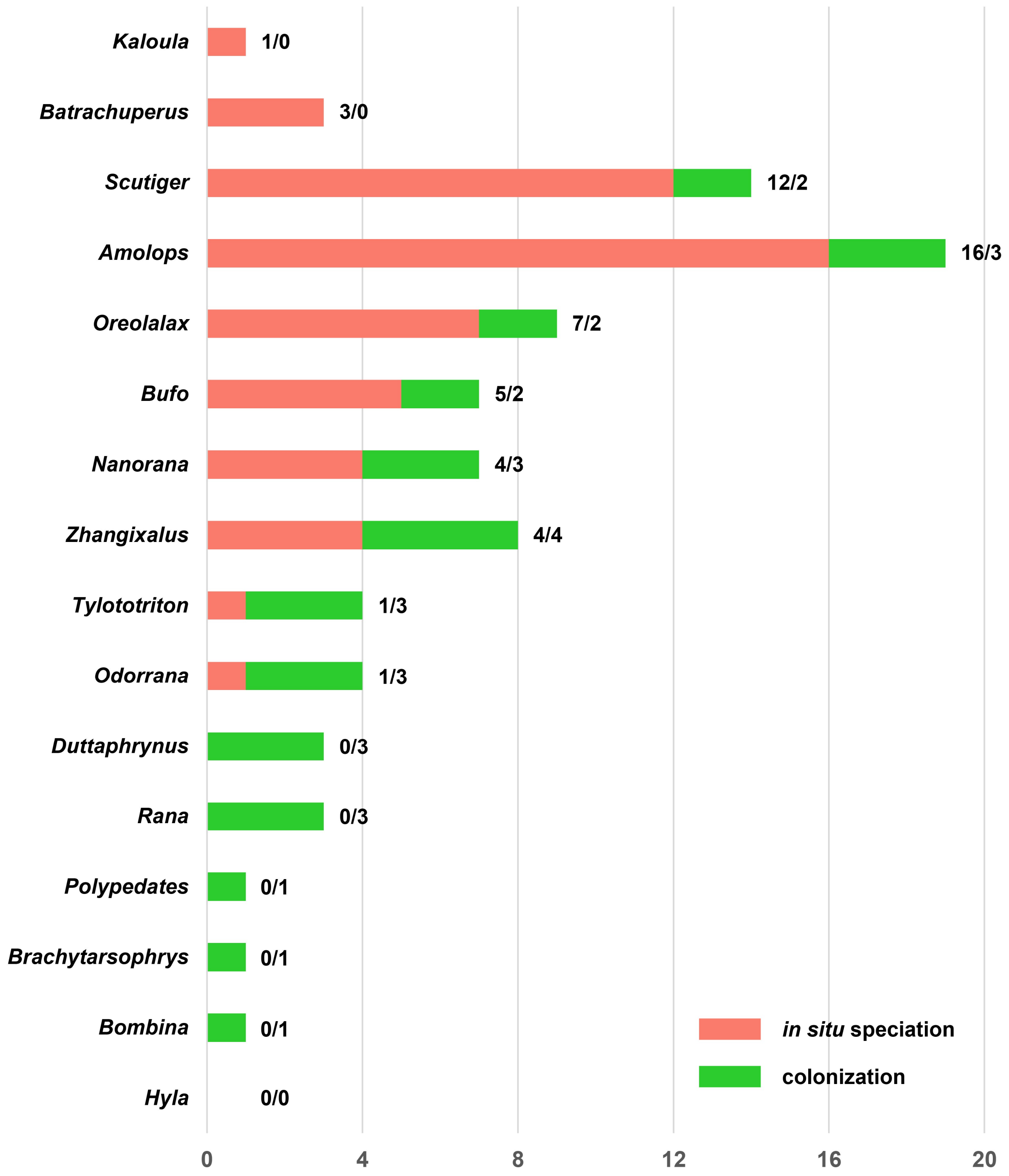

Fig. S123. Number of biogeographical events inferred from each amphibian clade. The results are estimated from 1,000 replicate simulations of biogeographic histories.

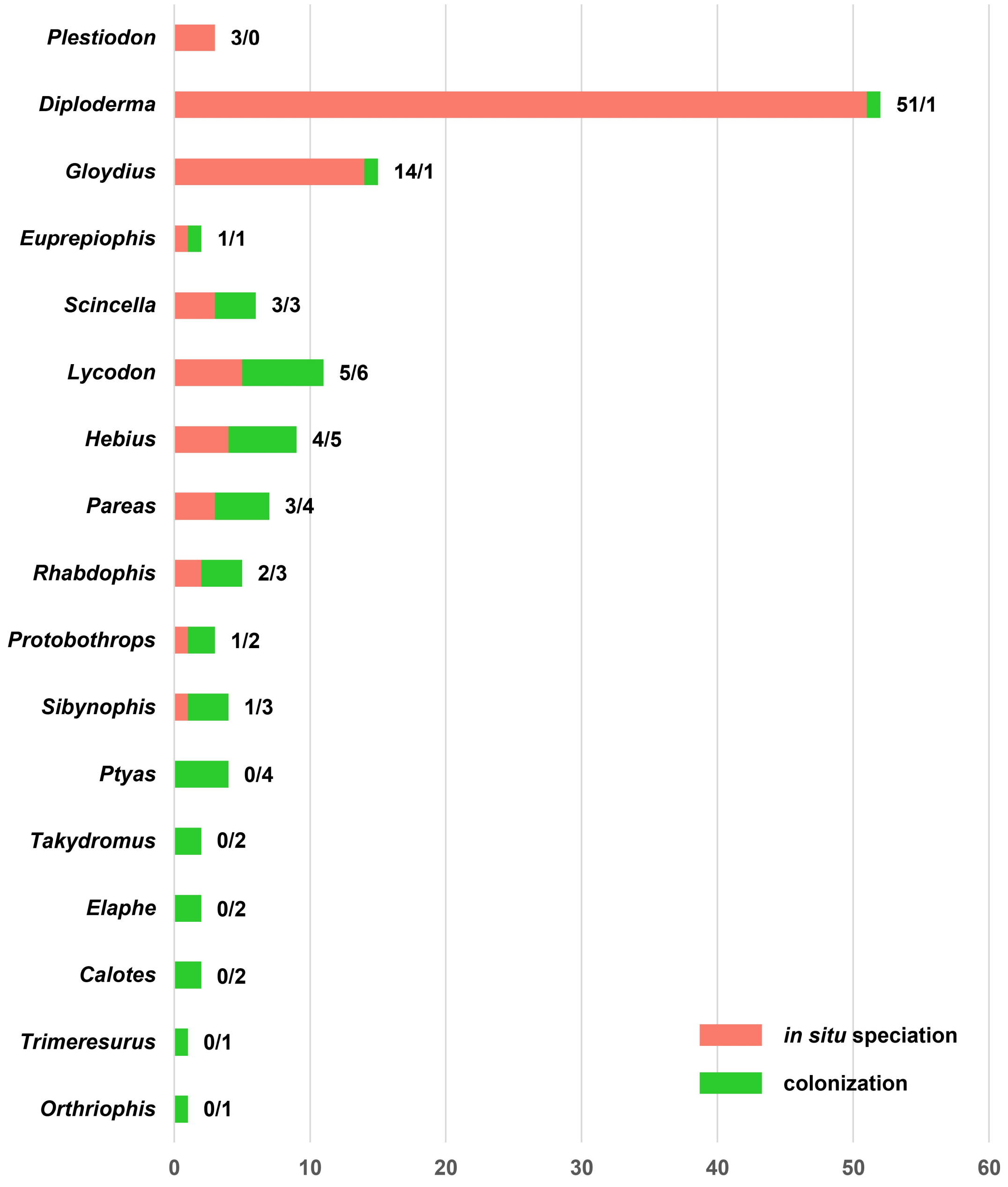

Fig. S124. Number of biogeographical events inferred from each non-avian reptile clade. The results are estimated from 1,000 replicate simulations of biogeographic histories.

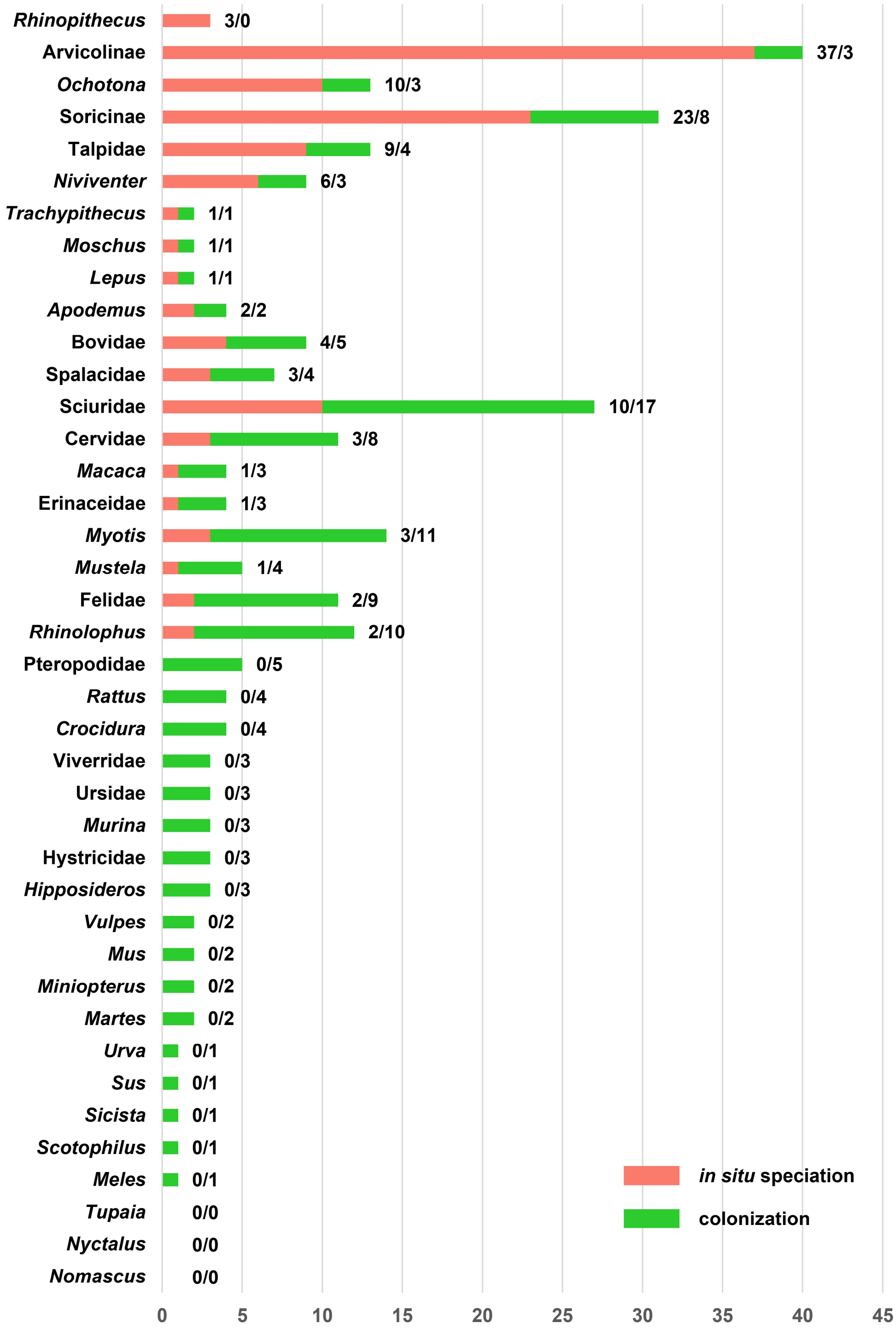

Fig. S125. Number of biogeographical events inferred from each mammal clade. The results are estimated from 1,000 replicate simulations of biogeographic histories.

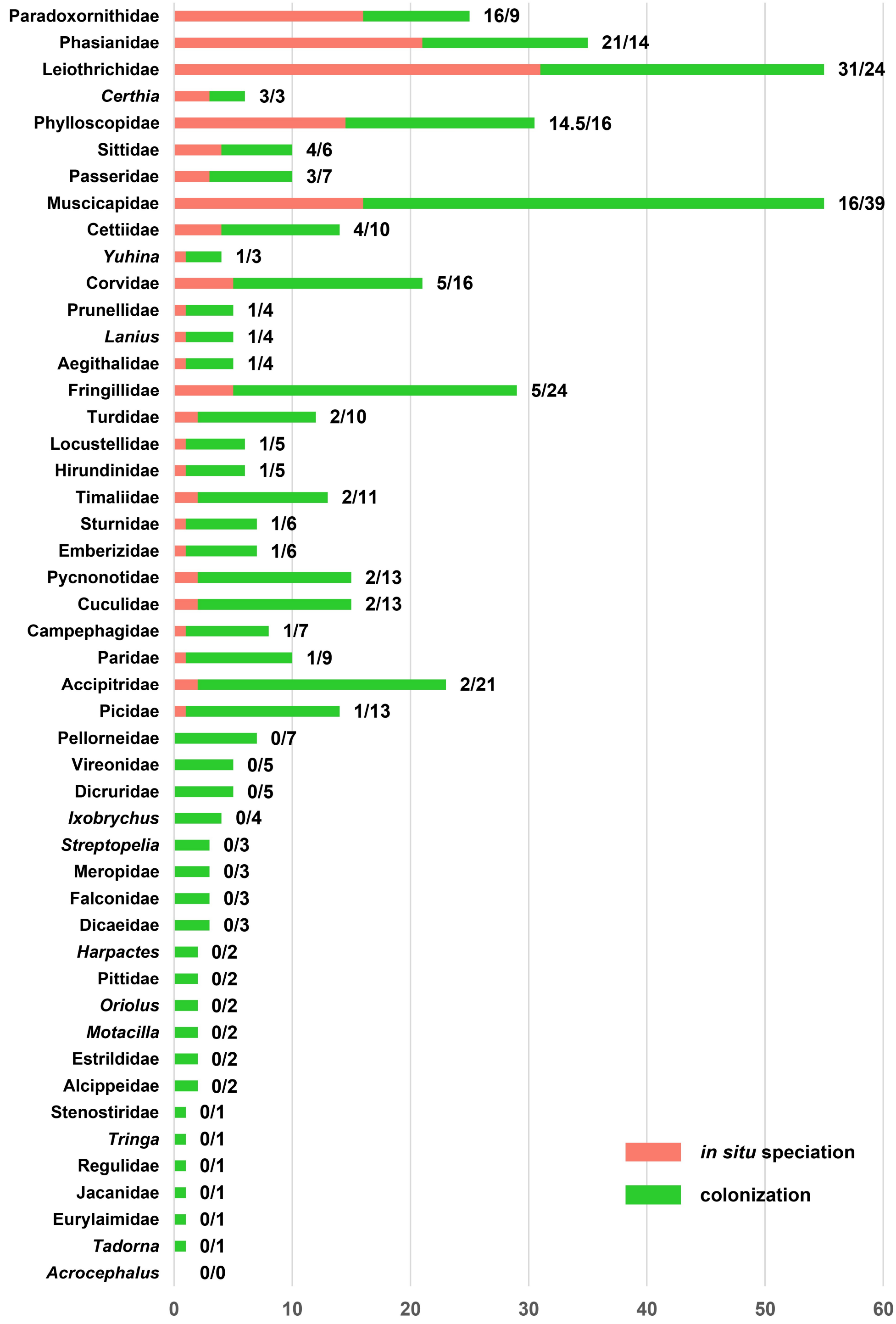

Fig. S126. Number of biogeographical events inferred from each avian clade. The results are estimated from 1,000 replicate simulations of biogeographic histories.

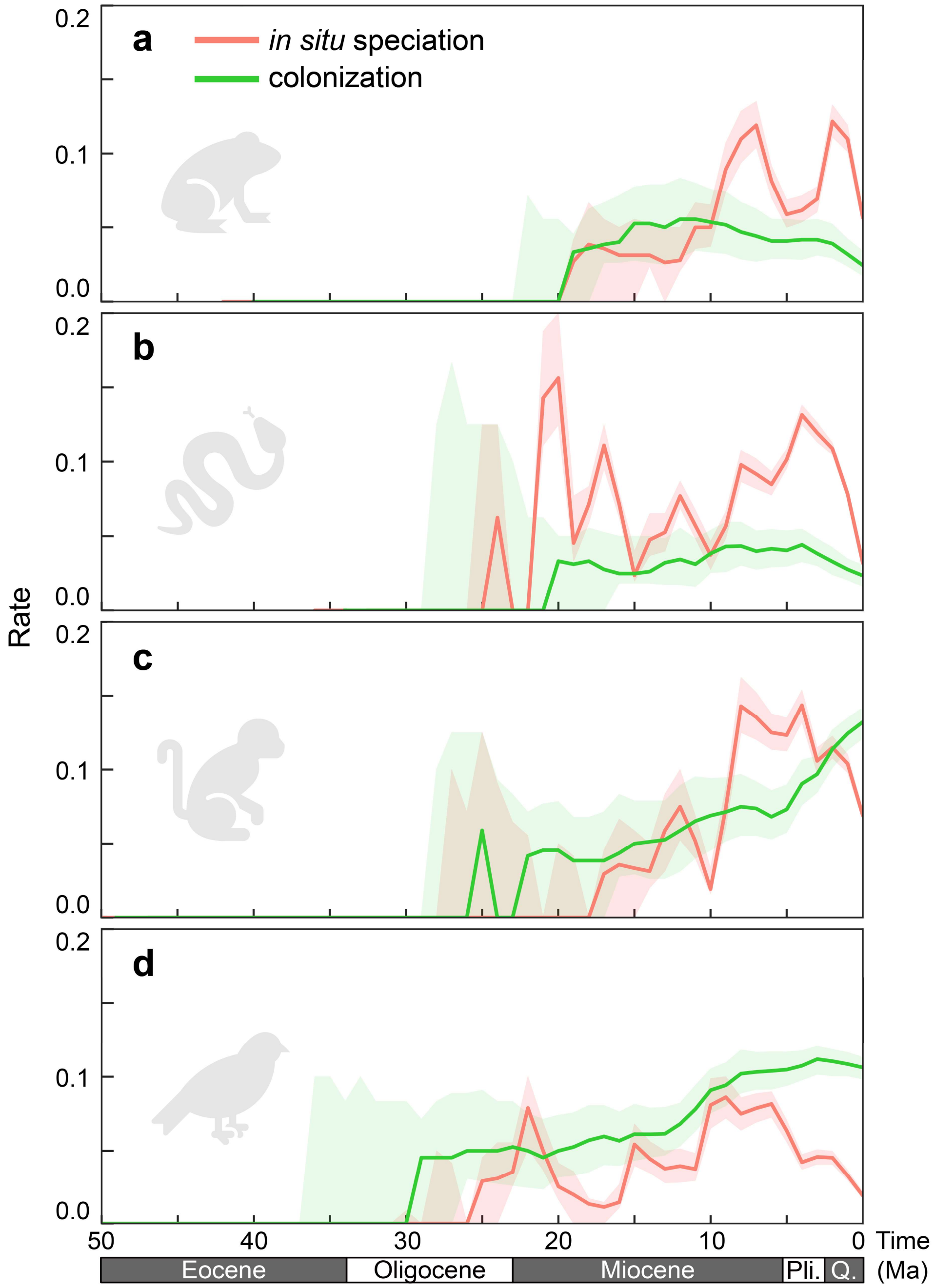

Fig. S127. Rolling estimates of *in situ* speciation and colonization rates through time for four terrestrial vertebrate taxa in the Hengduan Mountains. (a) Amphibia. (b) Non-avian Reptilia. (c) Mammalia. (d) Aves. Solid lines indicate median value and shaded regions indicate the 25% to 75% quantile intervals estimated from 1,000 replicate simulations of biogeographic histories. Rate estimates from the period before the earliest time at which the upper quantile value remains above zero continuously to the present are not shown. Pli., Pliocene; Q., Quaternary.

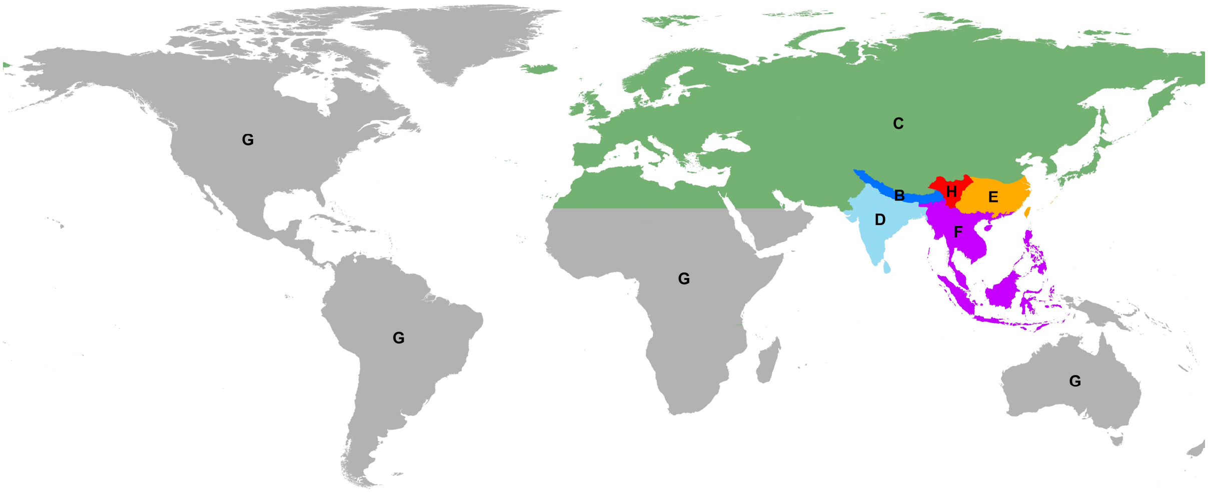

Fig. S128. Map of the 7 geographic regions used for ancestral range analyses in RevBayes. H = Hengduan Mountains, B = Himalayas, C = Palearctic, D = India, E = South China, F = Indo-Malay, G = other regions.

Supplementary Tables

Table S1. Checklist of terrestrial vertebrates in the Hengduan Mountains. # = sampled in this study. E = endemic to Hengduan Mountains. Data as of December 31, 2022.

Order	Family	Species	Endemism
**Class Amphibia**			
Caudata	Cryptobranchidae	*Andrias davidianus*	
Caudata	Hynobiidae	*Batrachuperus daochengensis*	E
Caudata	Hynobiidae	*Batrachuperus karlschmidti* #	E
Caudata	Hynobiidae	*Batrachuperus pinchonii* #	E
Caudata	Hynobiidae	*Batrachuperus tibetanus* #	
Caudata	Hynobiidae	*Batrachuperus yenyuanensis* #	E
Caudata	Salamandridae	*Tylototriton shanjing* #	
Caudata	Salamandridae	*Tylototriton taliangensis* #	
Caudata	Salamandridae	*Tylototriton verrucosus* #	
Caudata	Salamandridae	*Tylototriton wenxianensis* #	
Anura	Bombinatoridae	*Bombina maxima* #	
Anura	Bufonidae	*Bufo andrewsi*	
Anura	Bufonidae	*Bufo aspinius* #	E
Anura	Bufonidae	*Bufo gargarizans* #	
Anura	Bufonidae	*Bufo pageoti*	
Anura	Bufonidae	*Bufo tibetanus* #	E
Anura	Bufonidae	*Bufo tuberculatus* #	E
Anura	Bufonidae	*Bufo tuberospinius* #	
Anura	Bufonidae	*Duttaphrynus melanostictus* #	
Anura	Bufonidae	*Duttaphrynus stuarti* #	
Anura	Ceratobatrachidae	*Liurana xizangensis*	
Anura	Dicroglossidae	*Nanorana chayuensis* #	E
Anura	Dicroglossidae	*Nanorana parkeri* #	
Anura	Dicroglossidae	*Nanorana pleskei* #	
Anura	Dicroglossidae	*Nanorana quadranus* #	
Anura	Dicroglossidae	*Nanorana sichuanensis*	

Anura	Dicroglossidae	*Nanorana ventripunctata* #	E
Anura	Dicroglossidae	*Nanorana yunnanensis* #	
Anura	Dicroglossidae	*Quasipaa boulengeri*	
Anura	Hylidae	*Hyla annectans* #	
Anura	Megophryidae	*Oreolalax chuanbeiensis* #	E
Anura	Megophryidae	*Oreolalax longmenmontis*	E
Anura	Megophryidae	*Oreolalax major* #	E
Anura	Megophryidae	*Oreolalax popei* #	E
Anura	Megophryidae	*Oreolalax rugosus* #	E
Anura	Megophryidae	*Oreolalax schmidti* #	E
Anura	Megophryidae	*Oreolalax xiangchengensis* #	E
Anura	Megophryidae	*Scutiger boulengeri* #	
Anura	Megophryidae	*Scutiger chintingensis* #	E
Anura	Megophryidae	*Scutiger glandulatus* #	E
Anura	Megophryidae	*Scutiger gongshanensis* #	E
Anura	Megophryidae	*Scutiger jiulongensis* #	E
Anura	Megophryidae	*Scutiger maculatus*	E
Anura	Megophryidae	*Scutiger mammatus* #	E
Anura	Megophryidae	*Scutiger muliensis* #	E
Anura	Megophryidae	*Scutiger nyingchiensis* #	
Anura	Megophryidae	*Scutiger pingwuensis* #	E
Anura	Megophryidae	*Scutiger tengchongensis* #	E
Anura	Megophryidae	*Scutiger tuberculatus* #	E
Anura	Megophryidae	*Scutiger wanglangensis* #	E
Anura	Megophryidae	*Boulenophrys binchuanensis*	
Anura	Megophryidae	*Boulenophrys lushuiensis*	E
Anura	Megophryidae	*Boulenophrys minor*	
Anura	Megophryidae	*Atympanophrys nankiangensis*	
Anura	Megophryidae	*Atympanophrys shapingensis*	E
Anura	Megophryidae	*Xenophrys glandulosa*	
Anura	Megophryidae	*Xenophrys periosa*	
Anura	Megophryidae	*Brachytarsophrys platyparietus* #	
Anura	Megophryidae	*Leptobrachium tengchongense*	E

Anura	Megophryidae	*Leptobrachella tengchongensis*	E
Anura	Microhylidae	*Kaloula rugifera* #	
Anura	Microhylidae	*Kaloula verrucosa* #	
Anura	Microhylidae	*Glyphoglossus huadianensis*	E
Anura	Microhylidae	*Glyphoglossus yunnanensis*	
Anura	Ranidae	*Odorrana andersonii* #	
Anura	Ranidae	*Odorrana dulongensis*	E
Anura	Ranidae	*Odorrana grahami* #	
Anura	Ranidae	*Odorrana margaretae* #	
Anura	Ranidae	*Odorrana schmackeri* #	
Anura	Ranidae	*Nidirana occidentalis*	
Anura	Ranidae	*Nidirana pleuraden*	
Anura	Ranidae	*Amolops bellulus* #	E
Anura	Ranidae	*Amolops chaochin* #	
Anura	Ranidae	*Amolops chayuensis* #	E
Anura	Ranidae	*Amolops deng* #	
Anura	Ranidae	*Amolops granulosus* #	
Anura	Ranidae	*Amolops jinjiangensis* #	E
Anura	Ranidae	*Amolops kaulbacki* #	E
Anura	Ranidae	*Amolops lifanensis* #	E
Anura	Ranidae	*Amolops loloensis* #	
Anura	Ranidae	*Amolops mantzorum* #	E
Anura	Ranidae	*Amolops nyingchiensis* #	
Anura	Ranidae	*Amolops putaoensis* #	E
Anura	Ranidae	*Amolops wangyufani* #	E
Anura	Ranidae	*Rana chaochiaoensis* #	
Anura	Ranidae	*Rana chensinensis* #	
Anura	Ranidae	*Rana kukunoris* #	
Anura	Ranidae	*Rana omeimontis* #	
Anura	Ranidae	*Rana shuchinae* #	E
Anura	Rhacophoridae	*Polypedates braueri* #	
Anura	Rhacophoridae	*Raorchestes dulongensis*	E
Anura	Rhacophoridae	*Zhangixalus burmanus* #	

Anura	Rhacophoridae	*Zhangixalus chenfui* #	
Anura	Rhacophoridae	*Zhangixalus dugritei* #	E
Anura	Rhacophoridae	*Zhangixalus hungfuensis* #	E
Anura	Rhacophoridae	*Zhangixalus omeimontis* #	
Anura	Rhacophoridae	*Zhangixalus puerensis* #	
	**SUBTOTAL**	**97**	**45**
**Class Reptilia (excluding birds)**			
Testudines	Platysternidae	*Platysternon megacephalum*	
Serpentes	Calamariidae	*Calamaria pavimentata*	
Serpentes	Colubridae	*Ahaetulla prasina*	
Serpentes	Colubridae	*Archelaphe bella*	
Serpentes	Colubridae	*Boiga multomaculata*	
Serpentes	Colubridae	*Ptyas dhumnades* #	
Serpentes	Colubridae	*Ptyas major* #	
Serpentes	Colubridae	*Ptyas mucosa* #	
Serpentes	Colubridae	*Ptyas nigromarginata*	
Serpentes	Colubridae	*Elaphe bimaculata* #	
Serpentes	Colubridae	*Elaphe carinata* #	
Serpentes	Colubridae	*Elaphe dione* #	
Serpentes	Colubridae	*Elaphe taeniura* #	
Serpentes	Colubridae	*Elaphe zoigeensis* #	E
Serpentes	Colubridae	*Euprepiophis mandarinus* #	
Serpentes	Colubridae	*Euprepiophis perlaceus* #	E
Serpentes	Colubridae	*Lycodon chapaensis* #	
Serpentes	Colubridae	*Lycodon gongshan* #	E
Serpentes	Colubridae	*Lycodon liuchengchaoi* #	
Serpentes	Colubridae	*Lycodon multizonatus* #	
Serpentes	Colubridae	*Lycodon rufozonatus* #	
Serpentes	Colubridae	*Lycodon ruhstrati* #	
Serpentes	Colubridae	*Lycodon serratus* #	E
Serpentes	Colubridae	*Lycodon zayuensis* #	E
Serpentes	Colubridae	*Oligodon albocinctus*	
Serpentes	Colubridae	*Oligodon hamptoni*	

Serpentes	Colubridae	*Oreocryptophis porphyraceus*	
Serpentes	Dipsadidae	*Thermophis shangrila*	E
Serpentes	Dipsadidae	*Thermophis zhaoermii*	E
Serpentes	Elapidae	*Ophiophagus hannah*	
Serpentes	Elapidae	*Sinomicrurus macclellandi*	
Serpentes	Natricidae	*Hebius bitaeniatus* #	
Serpentes	Natricidae	*Hebius craspedogaster* #	
Serpentes	Natricidae	*Hebius johannis* #	
Serpentes	Natricidae	*Hebius maximus* #	
Serpentes	Natricidae	*Hebius modestus* #	
Serpentes	Natricidae	*Hebius octolineatus* #	
Serpentes	Natricidae	*Hebius weixiensis* #	E
Serpentes	Natricidae	*Hebius yanbianensis* #	
Serpentes	Natricidae	*Herpetoreas burbrinki*	E
Serpentes	Natricidae	*Rhabdophis chiwen* #	E
Serpentes	Natricidae	*Rhabdophis helleri* #	
Serpentes	Natricidae	*Rhabdophis himalayanus* #	
Serpentes	Natricidae	*Rhabdophis leonardi* #	
Serpentes	Natricidae	*Rhabdophis nuchalis* #	
Serpentes	Natricidae	*Rhabdophis pentasupralabialis* #	
Serpentes	Natricidae	*Trimerodytes percarinatus*	
Serpentes	Pareidae	*Pareas boulengeri* #	
Serpentes	Pareidae	*Pareas chinensis* #	
Serpentes	Pareidae	*Pareas macularius* #	
Serpentes	Pareidae	*Pareas monticola* #	
Serpentes	Pareidae	*Pareas nigriceps* #	E
Serpentes	Pareidae	*Pareas yunnanensis* #	E
Serpentes	Pseudoxenodontidae	*Plagiopholis styani*	
Serpentes	Pseudoxenodontidae	*Pseudoxenodon karlschmidti*	
Serpentes	Pseudoxenodontidae	*Pseudoxenodon macrops*	
Serpentes	Sibynophiidae	*Sibynophis chinensis* #	
Serpentes	Sibynophiidae	*Sibynophis collaris* #	
Serpentes	Typhlopidae	*Argyrophis diardii*	

Serpentes	Typhlopidae	*Indotyphlops braminus*	
Serpentes	Viperidae	*Azemiops kharini*	
Serpentes	Viperidae	*Gloydius angusticeps* #	E
Serpentes	Viperidae	*Gloydius brevicauda* #	
Serpentes	Viperidae	*Gloydius huangi* #	E
Serpentes	Viperidae	*Gloydius lateralis* #	E
Serpentes	Viperidae	*Gloydius lipipengi* #	E
Serpentes	Viperidae	*Gloydius monticola* #	E
Serpentes	Viperidae	*Gloydius rubromaculatus* #	
Serpentes	Viperidae	*Gloydius strauchi* #	E
Serpentes	Viperidae	*Gloydius swild* #	E
Serpentes	Viperidae	*Ovophis zayuensis*	
Serpentes	Viperidae	*Protobothrops jerdonii* #	
Serpentes	Viperidae	*Protobothrops kaulbacki* #	
Serpentes	Viperidae	*Protobothrops mucrosquamatus* #	
Serpentes	Viperidae	*Protobothrops xiangchengensis* #	E
Serpentes	Viperidae	*Trimeresurus yunnanensis* #	
Serpentes	Xenodermidae	*Achalinus meiguensis*	E
Serpentes	Xenodermidae	*Achalinus panzhihuaensis*	E
Serpentes	Xenodermidae	*Achalinus spinalis*	
Lacertilia	Agamidae	*Calotes emma* #	
Lacertilia	Agamidae	*Calotes mystaceus* #	
Lacertilia	Agamidae	*Diploderma angustelinea* #	E
Lacertilia	Agamidae	*Diploderma aorun* #	E
Lacertilia	Agamidae	*Diploderma batangense* #	E
Lacertilia	Agamidae	*Diploderma bowoense* #	E
Lacertilia	Agamidae	*Diploderma brevicauda* #	E
Lacertilia	Agamidae	*Diploderma daochengense* #	E
Lacertilia	Agamidae	*Diploderma drukdaypo* #	E
Lacertilia	Agamidae	*Diploderma dymondi* #	
Lacertilia	Agamidae	*Diploderma flaviceps* #	E
Lacertilia	Agamidae	*Diploderma flavilabre* #	E
Lacertilia	Agamidae	*Diploderma formosgulae* #	E

Lacertilia	Agamidae	*Diploderma iadinum* #	E
Lacertilia	Agamidae	*Diploderma kangdingense* #	E
Lacertilia	Agamidae	*Diploderma laeviventre* #	E
Lacertilia	Agamidae	*Diploderma limingense* #	E
Lacertilia	Agamidae	*Diploderma micangshanense* #	
Lacertilia	Agamidae	*Diploderma panchi* #	E
Lacertilia	Agamidae	*Diploderma panlong* #	E
Lacertilia	Agamidae	*Diploderma qilin* #	E
Lacertilia	Agamidae	*Diploderma shuoquense* #	E
Lacertilia	Agamidae	*Diploderma slowinskii* #	E
Lacertilia	Agamidae	*Diploderma swild* #	E
Lacertilia	Agamidae	*Diploderma vela* #	E
Lacertilia	Agamidae	*Diploderma xinlongense* #	E
Lacertilia	Agamidae	*Diploderma yangi* #	E
Lacertilia	Agamidae	*Diploderma yongshengense* #	E
Lacertilia	Agamidae	*Diploderma yulongense* #	E
Lacertilia	Agamidae	*Diploderma yunnanense* #	
Lacertilia	Agamidae	*Diploderma zhaoermii* #	E
Lacertilia	Agamidae	*Pseudocalotes kakhienensis*	
Lacertilia	Agamidae	*Pseudocalotes kingdonwardi*	E
Lacertilia	Anguidae	*Dopasia gracilis*	
Lacertilia	Anguidae	*Dopasia harti*	
Lacertilia	Gekkonidae	*Cyrtodactylus cayuensis*	E
Lacertilia	Gekkonidae	*Gekko jinjiangensis*	E
Lacertilia	Gekkonidae	*Gekko scabridus*	
Lacertilia	Gekkonidae	*Hemidactylus aquilonius*	
Lacertilia	Gekkonidae	*Hemiphyllodactylus yunnanensis*	
Lacertilia	Gekkonidae	*Hemiphyllodactylus zayuensis*	E
Lacertilia	Lacertidae	*Takydromus intermedius* #	
Lacertilia	Lacertidae	*Takydromus septentrionalis* #	
Lacertilia	Scincidae	*Protoblepharus nyingchiensis*	
Lacertilia	Scincidae	*Plestiodon capito* #	
Lacertilia	Scincidae	*Plestiodon elegans* #	

Lacertilia	Scincidae	*Plestiodon tunganus* #	E
Lacertilia	Scincidae	*Scincella doriae* #	
Lacertilia	Scincidae	*Scincella monticola* #	
Lacertilia	Scincidae	*Scincella potanini* #	E
Lacertilia	Scincidae	*Scincella reevesii* #	
Lacertilia	Scincidae	*Scincella tsinlingensis* #	
Lacertilia	Scincidae	*Sphenomorphus indicus*	
	**SUBTOTAL**	**132**	**54**
**Class Mammalia**			
Scandentia	Tupaiidae	*Tupaia belangeri* #	
Primates	Cercopithecidae	*Macaca arctoides* #	
Primates	Cercopithecidae	*Macaca assamensis* #	
Primates	Cercopithecidae	*Macaca leucogenys*	
Primates	Cercopithecidae	*Macaca mulatta* #	
Primates	Cercopithecidae	*Macaca thibetana* #	
Primates	Cercopithecidae	*Rhinopithecus bieti* #	E
Primates	Cercopithecidae	*Rhinopithecus roxellana* #	E
Primates	Cercopithecidae	*Rhinopithecus strykeri* #	E
Primates	Cercopithecidae	*Trachypithecus phayrei* #	
Primates	Cercopithecidae	*Trachypithecus shortridgei* #	E
Primates	Hylobatidae	*Hoolock tianxing*	
Primates	Hylobatidae	*Nomascus concolor* #	
Lagomorpha	Leporidae	*Lepus comus* #	
Lagomorpha	Leporidae	*Lepus oiostolus* #	
Lagomorpha	Ochotonidae	*Ochotona cansus* #	
Lagomorpha	Ochotonidae	*Ochotona curzoniae* #	
Lagomorpha	Ochotonidae	*Ochotona erythrotis* #	
Lagomorpha	Ochotonidae	*Ochotona flatcalvariam* #	
Lagomorpha	Ochotonidae	*Ochotona forresti* #	E
Lagomorpha	Ochotonidae	*Ochotona huanglongensis* #	E
Lagomorpha	Ochotonidae	*Ochotona macrotis* #	
Lagomorpha	Ochotonidae	*Ochotona qionglaiensis* #	E
Lagomorpha	Ochotonidae	*Ochotona roylei* #	

Lagomorpha	Ochotonidae	*Ochotona sacraria* #	E
Lagomorpha	Ochotonidae	*Ochotona thibetana* #	E
Rodentia	Sicistidae	*Sicista concolor* #	
Rodentia	Zapodidae	*Eozapus setchuanus*	E
Rodentia	Platacanthomyidae	*Typhlomys daloushanensis*	
Rodentia	Spalacidae	*Eospalax baileyi* #	E
Rodentia	Spalacidae	*Eospalax cansus* #	
Rodentia	Spalacidae	*Eospalax fontanierii* #	
Rodentia	Spalacidae	*Eospalax muliensis* #	E
Rodentia	Spalacidae	*Eospalax rothschildi* #	
Rodentia	Spalacidae	*Rhizomys pruinosus* #	
Rodentia	Spalacidae	*Rhizomys sinensis* #	
Rodentia	Muridae	*Apodemus chevrieri* #	
Rodentia	Muridae	*Apodemus draco* #	
Rodentia	Muridae	*Apodemus ilex*	E
Rodentia	Muridae	*Apodemus latronum* #	E
Rodentia	Muridae	*Apodemus peninsulae* #	
Rodentia	Muridae	*Bandicota indica*	
Rodentia	Muridae	*Berylmys bowersii*	
Rodentia	Muridae	*Chiropodomys gliroides*	
Rodentia	Muridae	*Dacnomys millardi*	
Rodentia	Muridae	*Leopoldamys edwardsi*	
Rodentia	Muridae	*Leopoldamys neilli*	
Rodentia	Muridae	*Micromys erythrotis*	
Rodentia	Muridae	*Mus cookii* #	
Rodentia	Muridae	*Mus musculus* #	
Rodentia	Muridae	*Mus pahari* #	
Rodentia	Muridae	*Niviventer andersoni* #	E
Rodentia	Muridae	*Niviventer brahma* #	E
Rodentia	Muridae	*Niviventer confucianus* #	
Rodentia	Muridae	*Niviventer eha* #	
Rodentia	Muridae	*Niviventer excelsior* #	E
Rodentia	Muridae	*Niviventer fulvescens* #	

Rodentia	Muridae	*Niviventer huang* #	
Rodentia	Muridae	*Niviventer niviventer* #	
Rodentia	Muridae	*Niviventer pianmaensis* #	E
Rodentia	Muridae	*Rattus andamanensis* #	
Rodentia	Muridae	*Rattus nitidus* #	
Rodentia	Muridae	*Rattus norvegicus* #	
Rodentia	Muridae	*Rattus tanezumi* #	
Rodentia	Muridae	*Vernaya fulva*	E
Rodentia	Cricetidae	*Neodon clarkei* #	
Rodentia	Cricetidae	*Neodon forresti* #	E
Rodentia	Cricetidae	*Neodon fuscus* #	E
Rodentia	Cricetidae	*Neodon irene* #	E
Rodentia	Cricetidae	*Proedromys bedfordi* #	E
Rodentia	Cricetidae	*Mictomicrotus liangshanensis* #	E
Rodentia	Cricetidae	*Volemys millicens* #	E
Rodentia	Cricetidae	*Volemys musseri* #	E
Rodentia	Cricetidae	*Caryomys eva* #	
Rodentia	Cricetidae	*Caryomys inez* #	
Rodentia	Cricetidae	*Eothenomys melanogaster* #	
Rodentia	Cricetidae	*Eothenomys shimianensis* #	E
Rodentia	Cricetidae	*Eothenomys cachinus* #	E
Rodentia	Cricetidae	*Eothenomys fidelis* #	E
Rodentia	Cricetidae	*Eothenomys miletus* #	
Rodentia	Cricetidae	*Anteliomys chinensis* #	E
Rodentia	Cricetidae	*Anteliomys custos* #	E
Rodentia	Cricetidae	*Anteliomys hintoni* #	E
Rodentia	Cricetidae	*Anteliomys jinyangensis* #	E
Rodentia	Cricetidae	*Anteliomys luojishanensis* #	E
Rodentia	Cricetidae	*Anteliomys meiguensis* #	E
Rodentia	Cricetidae	*Anteliomys olitor* #	E
Rodentia	Cricetidae	*Anteliomys proditor* #	E
Rodentia	Cricetidae	*Anteliomys tarquinius* #	E
Rodentia	Cricetidae	*Anteliomys wardi* #	E

Rodentia	Cricetidae	*Cansumys canus*	
Rodentia	Cricetidae	*Cricetulus longicaudatus*	
Rodentia	Cricetidae	*Urocricetus kamensis*	E
Rodentia	Cricetidae	*Tscherskia triton*	
Rodentia	Hystricidae	*Atherurus macrourus* #	
Rodentia	Hystricidae	*Hystrix brachyura* #	
Rodentia	Gliridae	*Chaetocauda sichuanensis*	E
Rodentia	Sciuridae	*Aeretes melanopterus* #	E
Rodentia	Sciuridae	*Belomys pearsonii* #	
Rodentia	Sciuridae	*Biswamoyopterus gaoligongensis* #	E
Rodentia	Sciuridae	*Eupetaurus nivamons* #	E
Rodentia	Sciuridae	*Hylopetes alboniger* #	
Rodentia	Sciuridae	*Petaurista alborufus* #	
Rodentia	Sciuridae	*Petaurista caniceps*	
Rodentia	Sciuridae	*Petaurista elegans* #	
Rodentia	Sciuridae	*Petaurista sybilla* #	
Rodentia	Sciuridae	*Petaurista xanthotis* #	E
Rodentia	Sciuridae	*Petaurista yunanensis* #	E
Rodentia	Sciuridae	*Priapomys leonardi* #	E
Rodentia	Sciuridae	*Trogopterus xanthipes* #	
Rodentia	Sciuridae	*Callosciurus erythraeus* #	
Rodentia	Sciuridae	*Callosciurus quinquestriatus*	E
Rodentia	Sciuridae	*Dremomys lokriah* #	
Rodentia	Sciuridae	*Dremomys pernyi* #	
Rodentia	Sciuridae	*Dremomys rufigenis* #	
Rodentia	Sciuridae	*Marmota himalayana* #	
Rodentia	Sciuridae	*Ratufa bicolor* #	
Rodentia	Sciuridae	*Sciurotamias davidianus* #	
Rodentia	Sciuridae	*Sciurotamias forresti* #	
Rodentia	Sciuridae	*Eutamias sibiricus* #	
Rodentia	Sciuridae	*Tamiops swinhoei* #	
Eulipotyphla	Talpidae	*Scapanulus oweni* #	
Eulipotyphla	Talpidae	*Scaptonyx fusicauda* #	

Eulipotyphla	Talpidae	*Euroscaptor grandis*	E
Eulipotyphla	Talpidae	*Euroscaptor longirostris* #	
Eulipotyphla	Talpidae	*Parascaptor leucurus* #	
Eulipotyphla	Talpidae	*Uropsilus aequodonenia* #	E
Eulipotyphla	Talpidae	*Uropsilus andersoni* #	E
Eulipotyphla	Talpidae	*Uropsilus atronates* #	
Eulipotyphla	Talpidae	*Uropsilus investigator* #	E
Eulipotyphla	Talpidae	*Uropsilus nivatus* #	E
Eulipotyphla	Talpidae	*Uropsilus soricipes* #	E
Eulipotyphla	Erinaceidae	*Erinaceus amurensis* #	
Eulipotyphla	Erinaceidae	*Mesechinus hughi* #	
Eulipotyphla	Erinaceidae	*Mesechinus wangi* #	E
Eulipotyphla	Erinaceidae	*Neotetracus sinensis* #	E
Eulipotyphla	Soricidae	*Crocidura attenuata* #	
Eulipotyphla	Soricidae	*Crocidura dracula*	
Eulipotyphla	Soricidae	*Crocidura indochinensis* #	
Eulipotyphla	Soricidae	*Crocidura rapax* #	
Eulipotyphla	Soricidae	*Crocidura shantungensis* #	
Eulipotyphla	Soricidae	*Crocidura vorax*	
Eulipotyphla	Soricidae	*Suncus murinus*	
Eulipotyphla	Soricidae	*Anourosorex squamipes* #	
Eulipotyphla	Soricidae	*Blarinella quadraticauda* #	
Eulipotyphla	Soricidae	*Blarinella wardi* #	E
Eulipotyphla	Soricidae	*Parablarinella griselda* #	
Eulipotyphla	Soricidae	*Chimarrogale himalayica* #	
Eulipotyphla	Soricidae	*Chimarrogale styani* #	E
Eulipotyphla	Soricidae	*Chodsigoa furva* #	E
Eulipotyphla	Soricidae	*Chodsigoa hypsibia* #	E
Eulipotyphla	Soricidae	*Chodsigoa parca* #	
Eulipotyphla	Soricidae	*Chodsigoa parva* #	E
Eulipotyphla	Soricidae	*Chodsigoa salenskii*	E
Eulipotyphla	Soricidae	*Chodsigoa smithii* #	
Eulipotyphla	Soricidae	*Episoriculus caudatus* #	

Eulipotyphla	Soricidae	*Episoriculus leucops* #	
Eulipotyphla	Soricidae	*Episoriculus macrurus* #	E
Eulipotyphla	Soricidae	*Episoriculus sacratus*	E
Eulipotyphla	Soricidae	*Soriculus nigrescens* #	
Eulipotyphla	Soricidae	*Nectogale elegans* #	E
Eulipotyphla	Soricidae	*Sorex bedfordiae* #	E
Eulipotyphla	Soricidae	*Sorex cansulus* #	E
Eulipotyphla	Soricidae	*Sorex cylindricauda* #	E
Eulipotyphla	Soricidae	*Sorex excelsus* #	E
Eulipotyphla	Soricidae	*Sorex kozlovi*	E
Eulipotyphla	Soricidae	*Sorex sinalis* #	E
Eulipotyphla	Soricidae	*Sorex thibetanus* #	E
Chiroptera	Pteropodidae	*Cynopterus sphinx* #	
Chiroptera	Pteropodidae	*Megaerops ecaudatus* #	
Chiroptera	Pteropodidae	*Megaerops niphanae* #	
Chiroptera	Pteropodidae	*Rousettus leschenaultii* #	
Chiroptera	Pteropodidae	*Sphaerias blanfordi* #	
Chiroptera	Hipposideridae	*Aselliscus stoliczkanus*	
Chiroptera	Hipposideridae	*Hipposideros armiger* #	
Chiroptera	Hipposideridae	*Hipposideros fulvus* #	
Chiroptera	Hipposideridae	*Hipposideros swinhoei* #	
Chiroptera	Rhinolophidae	*Rhinolophus affinis* #	
Chiroptera	Rhinolophidae	*Rhinolophus ferrumequinum* #	
Chiroptera	Rhinolophidae	*Rhinolophus luctus* #	
Chiroptera	Rhinolophidae	*Rhinolophus macrotis* #	
Chiroptera	Rhinolophidae	*Rhinolophus malayanus* #	
Chiroptera	Rhinolophidae	*Rhinolophus osgoodi*	
Chiroptera	Rhinolophidae	*Rhinolophus pearsonii* #	
Chiroptera	Rhinolophidae	*Rhinolophus pusillus* #	
Chiroptera	Rhinolophidae	*Rhinolophus rex* #	
Chiroptera	Rhinolophidae	*Rhinolophus schnitzleri*	
Chiroptera	Rhinolophidae	*Rhinolophus sinicus* #	
Chiroptera	Rhinolophidae	*Rhinolophus thomasi* #	

Chiroptera	Rhinolophidae	*Rhinolophus yunanensis* #	
Chiroptera	Emballonuridae	*Taphozous melanopogon*	
Chiroptera	Emballonuridae	*Taphozous theobaldi*	
Chiroptera	Molossidae	*Mops plicatus*	
Chiroptera	Miniopteridae	*Miniopterus fuliginosus* #	
Chiroptera	Miniopteridae	*Miniopterus pusillus* #	
Chiroptera	Vespertilionidae	*Kerivoula furva*	
Chiroptera	Vespertilionidae	*Kerivoula picta*	
Chiroptera	Vespertilionidae	*Murina aurata* #	
Chiroptera	Vespertilionidae	*Murina feae* #	
Chiroptera	Vespertilionidae	*Murina jinchui*	E
Chiroptera	Vespertilionidae	*Murina leucogaster* #	
Chiroptera	Vespertilionidae	*Myotis altarium* #	
Chiroptera	Vespertilionidae	*Myotis alticraniatus*	
Chiroptera	Vespertilionidae	*Myotis chinensis* #	
Chiroptera	Vespertilionidae	*Myotis davidii* #	
Chiroptera	Vespertilionidae	*Myotis fimbriatus* #	
Chiroptera	Vespertilionidae	*Myotis frater* #	
Chiroptera	Vespertilionidae	*Myotis hasseltii* #	
Chiroptera	Vespertilionidae	*Myotis horsfieldii* #	
Chiroptera	Vespertilionidae	*Myotis indochinensis*	
Chiroptera	Vespertilionidae	*Myotis laniger* #	
Chiroptera	Vespertilionidae	*Myotis montivagus* #	E
Chiroptera	Vespertilionidae	*Myotis muricola* #	
Chiroptera	Vespertilionidae	*Myotis siligorensis* #	
Chiroptera	Vespertilionidae	*Arielulus circumdatus*	
Chiroptera	Vespertilionidae	*Barbastella darjelingensis*	
Chiroptera	Vespertilionidae	*Eptesicus pachyomus*	
Chiroptera	Vespertilionidae	*Eptesicus pachyotis*	
Chiroptera	Vespertilionidae	*Hypsugo affinis*	
Chiroptera	Vespertilionidae	*Hypsugo pulveratus*	
Chiroptera	Vespertilionidae	*Ia io*	
Chiroptera	Vespertilionidae	*Nyctalus plancyi* #	

Chiroptera	Vespertilionidae	*Pipistrellus abramus*	
Chiroptera	Vespertilionidae	*Pipistrellus coromandra*	
Chiroptera	Vespertilionidae	*Pipistrellus javanicus*	
Chiroptera	Vespertilionidae	*Pipistrellus paterculus*	
Chiroptera	Vespertilionidae	*Pipistrellus tenuis*	
Chiroptera	Vespertilionidae	*Scotophilus heathii* #	
Chiroptera	Vespertilionidae	*Tylonycteris robustula*	
Chiroptera	Vespertilionidae	*Vespertilio sinensis*	
Artiodactyla	Suidae	*Sus scrofa* #	
Artiodactyla	Cervidae	*Capreolus pygargus* #	
Artiodactyla	Cervidae	*Cervus albirostris* #	
Artiodactyla	Cervidae	*Cervus elaphus* #	
Artiodactyla	Cervidae	*Cervus nippon* #	
Artiodactyla	Cervidae	*Rusa unicolor* #	
Artiodactyla	Cervidae	*Elaphodus cephalophus* #	
Artiodactyla	Cervidae	*Muntiacus gongshanensis* #	E
Artiodactyla	Cervidae	*Muntiacus reevesi* #	
Artiodactyla	Cervidae	*Muntiacus vaginalis* #	
Artiodactyla	Bovidae	*Procapra picticaudata* #	
Artiodactyla	Bovidae	*Bos gaurus* #	
Artiodactyla	Bovidae	*Budorcas taxicolor* #	E
Artiodactyla	Bovidae	*Capricornis rubidus*	
Artiodactyla	Bovidae	*Capricornis sumatraensis* #	
Artiodactyla	Bovidae	*Naemorhedus baileyi* #	E
Artiodactyla	Bovidae	*Naemorhedus evansi*	
Artiodactyla	Bovidae	*Naemorhedus griseus* #	
Artiodactyla	Bovidae	*Pseudois nayaur* #	
Artiodactyla	Moschidae	*Moschus berezovskii* #	
Artiodactyla	Moschidae	*Moschus chrysogaster* #	
Artiodactyla	Moschidae	*Moschus fuscus* #	E
Pholidota	Manidae	*Manis pentadactyla*	
Carnivora	Felidae	*Catopuma temminckii* #	
Carnivora	Felidae	*Felis bieti*	

Carnivora	Felidae	*Felis chaus* #	
Carnivora	Felidae	*Lynx lynx* #	
Carnivora	Felidae	*Otocolobus manul* #	
Carnivora	Felidae	*Pardofelis marmorata* #	
Carnivora	Felidae	*Prionailurus bengalensis* #	
Carnivora	Felidae	*Neofelis nebulosa* #	
Carnivora	Felidae	*Panthera pardus* #	
Carnivora	Felidae	*Panthera uncia* #	
Carnivora	Prionodontidae	*Prionodon pardicolor*	
Carnivora	Viverridae	*Paradoxurus hermaphroditus* #	
Carnivora	Viverridae	*Paguma larvata* #	
Carnivora	Viverridae	*Viverra zibetha* #	
Carnivora	Viverridae	*Viverricula indica* #	
Carnivora	Canidae	*Urva urva* #	
Carnivora	Canidae	*Canis lupus*	
Carnivora	Canidae	*Cuon alpinus*	
Carnivora	Canidae	*Nyctereutes procyonoides*	
Carnivora	Canidae	*Vulpes ferrilata* #	
Carnivora	Canidae	*Vulpes vulpes* #	
Carnivora	Ursidae	*Ailuropoda melanoleuca* #	E
Carnivora	Ursidae	*Ursus arctos* #	
Carnivora	Ursidae	*Ursus thibetanus* #	
Carnivora	Ailuridae	*Ailurus styani*	E
Carnivora	Mustelidae	*Lutra cinerea*	
Carnivora	Mustelidae	*Lutra lutra*	
Carnivora	Mustelidae	*Lutra perspicillata*	
Carnivora	Mustelidae	*Arctonyx collaris*	
Carnivora	Mustelidae	*Meles leucurus* #	
Carnivora	Mustelidae	*Melogale moschata*	
Carnivora	Mustelidae	*Melogale personata*	
Carnivora	Mustelidae	*Martes flavigula* #	
Carnivora	Mustelidae	*Martes foina* #	
Carnivora	Mustelidae	*Mustela altaica* #	

Carnivora	Mustelidae	*Mustela eversmanii* #	
Carnivora	Mustelidae	*Mustela kathiah* #	
Carnivora	Mustelidae	*Mustela sibirica* #	
Carnivora	Mustelidae	*Mustela strigidorsa* #	
Carnivora	Mustelidae	*Mustela aistoodonnivalis*	E
	**SUBTOTAL**	**293**	**81**
**Group Aves (Class Reptilia)**			
Anseriformes	Anatidae	*Tadorna ferruginea* #	
Anseriformes	Anatidae	*Nettapus coromandelianus*	
Anseriformes	Anatidae	*Anas poecilorhyncha*	
Anseriformes	Anatidae	*Anas zonorhyncha*	
Galliformes	Phasianidae	*Arborophila torqueola* #	
Galliformes	Phasianidae	*Arborophila rufipectus* #	E
Galliformes	Phasianidae	*Arborophila rufogularis* #	
Galliformes	Phasianidae	*Lerwa lerwa*	
Galliformes	Phasianidae	*Ithaginis cruentus* #	
Galliformes	Phasianidae	*Tragopan blythii* #	
Galliformes	Phasianidae	*Tragopan temminckii* #	
Galliformes	Phasianidae	*Tetraophasis obscurus* #	
Galliformes	Phasianidae	*Tetraophasis szechenyii* #	E
Galliformes	Phasianidae	*Lophophorus impejanus* #	
Galliformes	Phasianidae	*Lophophorus sclateri* #	E
Galliformes	Phasianidae	*Lophophorus lhuysii* #	E
Galliformes	Phasianidae	*Pucrasia macrolopha* #	
Galliformes	Phasianidae	*Tetrastes sewerzowi* #	
Galliformes	Phasianidae	*Perdix hodgsoniae* #	
Galliformes	Phasianidae	*Syrmaticus reevesii* #	
Galliformes	Phasianidae	*Syrmaticus humiae* #	
Galliformes	Phasianidae	*Chrysolophus pictus* #	
Galliformes	Phasianidae	*Chrysolophus amherstiae* #	E
Galliformes	Phasianidae	*Phasianus colchicus* #	
Galliformes	Phasianidae	*Crossoptilon crossoptilon* #	E
Galliformes	Phasianidae	*Crossoptilon auritum* #	

Galliformes	Phasianidae	*Lophura leucomelanos* #
Galliformes	Phasianidae	*Lophura nycthemera* #
Galliformes	Phasianidae	*Pavo muticus* #
Galliformes	Phasianidae	*Bambusicola fytchii* #
Galliformes	Phasianidae	*Gallus gallus* #
Galliformes	Phasianidae	*Francolinus pintadeanus* #
Galliformes	Phasianidae	*Tetraogallus tibetanus* #
Podicipediformes	Podicipedidae	*Tachybaptus ruficollis*
Pelecaniformes	Ardeidae	*Ixobrychus sinensis* #
Pelecaniformes	Ardeidae	*Ixobrychus cinnamomeus* #
Pelecaniformes	Ardeidae	*Gorsachius magnificus*
Pelecaniformes	Ardeidae	*Nycticorax nycticorax*
Pelecaniformes	Ardeidae	*Butorides striata*
Pelecaniformes	Ardeidae	*Ardeola bacchus*
Pelecaniformes	Ardeidae	*Bubulcus coromandus*
Pelecaniformes	Ardeidae	*Ardea alba*
Pelecaniformes	Ardeidae	*Ardea intermedia*
Pelecaniformes	Ardeidae	*Egretta garzetta*
Accipitriformes	Pandionidae	*Pandion haliaetus* #
Accipitriformes	Accipitridae	*Elanus caeruleus* #
Accipitriformes	Accipitridae	*Gypaetus barbatus* #
Accipitriformes	Accipitridae	*Pernis ptilorhynchus* #
Accipitriformes	Accipitridae	*Aviceda jerdoni*
Accipitriformes	Accipitridae	*Aviceda leuphotes*
Accipitriformes	Accipitridae	*Gyps bengalensis* #
Accipitriformes	Accipitridae	*Gyps himalayensis* #
Accipitriformes	Accipitridae	*Sarcogyps calvus* #
Accipitriformes	Accipitridae	*Aegypius monachus* #
Accipitriformes	Accipitridae	*Spilornis cheela* #
Accipitriformes	Accipitridae	*Nisaetus cirrhatus* #
Accipitriformes	Accipitridae	*Nisaetus nipalensis* #
Accipitriformes	Accipitridae	*Ictinaetus malaiensis* #
Accipitriformes	Accipitridae	*Aquila chrysaetos* #

Accipitriformes	Accipitridae	*Accipiter trivirgatus* #
Accipitriformes	Accipitridae	*Accipiter badius*
Accipitriformes	Accipitridae	*Accipiter soloensis* #
Accipitriformes	Accipitridae	*Accipiter virgatus* #
Accipitriformes	Accipitridae	*Accipiter nisus* #
Accipitriformes	Accipitridae	*Accipiter gentilis* #
Accipitriformes	Accipitridae	*Milvus migrans* #
Accipitriformes	Accipitridae	*Haliaeetus leucoryphus* #
Accipitriformes	Accipitridae	*Buteo hemilasius* #
Accipitriformes	Accipitridae	*Buteo refectus*
Gruiformes	Rallidae	*Rallus aquaticus*
Gruiformes	Rallidae	*Lewinia striata*
Gruiformes	Rallidae	*Gallinula chloropus*
Gruiformes	Rallidae	*Porphyrio poliocephalus*
Gruiformes	Rallidae	*Zapornia bicolor*
Gruiformes	Rallidae	*Zapornia fusca*
Gruiformes	Rallidae	*Gallicrex cinerea*
Gruiformes	Rallidae	*Amaurornis phoenicurus*
Charadriiformes	Turnicidae	*Turnix suscitator*
Charadriiformes	Ibidorhynchidae	*Ibidorhyncha struthersii*
Charadriiformes	Charadriidae	*Vanellus duvaucelii*
Charadriiformes	Charadriidae	*Vanellus indicus*
Charadriiformes	Charadriidae	*Charadrius dubius*
Charadriiformes	Charadriidae	*Charadrius mongolus*
Charadriiformes	Rostratulidae	*Rostratula benghalensis*
Charadriiformes	Jacanidae	*Hydrophasianus chirurgus* #
Charadriiformes	Scolopacidae	*Gallinago nemoricola*
Charadriiformes	Scolopacidae	*Tringa totanus* #
Charadriiformes	Laridae	*Sterna hirundo*
Pterocliformes	Pteroclidae	*Syrrhaptes tibetanus*
Columbiformes	Columbidae	*Columba rupestris*
Columbiformes	Columbidae	*Columba leuconota*
Columbiformes	Columbidae	*Columba hodgsonii*

Columbiformes	Columbidae	*Columba pulchricollis*
Columbiformes	Columbidae	*Streptopelia orientalis* #
Columbiformes	Columbidae	*Streptopelia tranquebarica* #
Columbiformes	Columbidae	*Spilopelia chinensis*
Columbiformes	Columbidae	*Macropygia unchall*
Columbiformes	Columbidae	*Chalcophaps indica*
Columbiformes	Columbidae	*Treron apicauda*
Columbiformes	Columbidae	*Treron sphenurus*
Cuculiformes	Cuculidae	*Centropus sinensis* #
Cuculiformes	Cuculidae	*Centropus bengalensis* #
Cuculiformes	Cuculidae	*Phaenicophaeus tristis* #
Cuculiformes	Cuculidae	*Clamator coromandus* #
Cuculiformes	Cuculidae	*Eudynamys scolopaceus* #
Cuculiformes	Cuculidae	*Chrysococcyx maculatus* #
Cuculiformes	Cuculidae	*Chrysococcyx xanthorhynchus* #
Cuculiformes	Cuculidae	*Cacomantis sonneratii* #
Cuculiformes	Cuculidae	*Cacomantis merulinus* #
Cuculiformes	Cuculidae	*Surniculus lugubris* #
Cuculiformes	Cuculidae	*Hierococcyx sparverioides* #
Cuculiformes	Cuculidae	*Hierococcyx nisicolor*
Cuculiformes	Cuculidae	*Cuculus poliocephalus* #
Cuculiformes	Cuculidae	*Cuculus micropterus* #
Cuculiformes	Cuculidae	*Cuculus saturatus* #
Cuculiformes	Cuculidae	*Cuculus canorus* #
Strigiformes	Tytonidae	*Tyto javanica*
Strigiformes	Tytonidae	*Tyto longimembris*
Strigiformes	Tytonidae	*Phodilus badius*
Strigiformes	Strigidae	*Ninox scutulata*
Strigiformes	Strigidae	*Taenioptynx brodiei*
Strigiformes	Strigidae	*Athene noctua*
Strigiformes	Strigidae	*Glaucidium cuculoides*
Strigiformes	Strigidae	*Otus spilocephalus*
Strigiformes	Strigidae	*Otus sunia*

Strigiformes	Strigidae	*Otus lettia*
Strigiformes	Strigidae	*Bubo bubo*
Strigiformes	Strigidae	*Ketupa nipalensis*
Strigiformes	Strigidae	*Ketupa zeylonensis*
Strigiformes	Strigidae	*Ketupa flavipes*
Strigiformes	Strigidae	*Strix leptogrammica*
Strigiformes	Strigidae	*Strix nivicolum*
Strigiformes	Strigidae	*Strix uralensis*
Caprimulgiformes	Caprimulgidae	*Lyncornis macrotis*
Caprimulgiformes	Caprimulgidae	*Caprimulgus jotaka*
Caprimulgiformes	Caprimulgidae	*Caprimulgus macrurus*
Apodiformes	Apodidae	*Aerodramus brevirostris*
Apodiformes	Apodidae	*Hirundapus caudacutus*
Apodiformes	Apodidae	*Cypsiurus balasiensis*
Apodiformes	Apodidae	*Apus pacificus*
Apodiformes	Apodidae	*Apus salimalii*
Apodiformes	Apodidae	*Apus nipalensis*
Apodiformes	Trogonidae	*Harpactes erythrocephalus* [#]
Apodiformes	Trogonidae	*Harpactes wardi* [#]
Coraciiformes	Coraciidae	*Coracias affinis*
Coraciiformes	Coraciidae	*Eurystomus orientalis*
Coraciiformes	Alcedinidae	*Halcyon smyrnensis*
Coraciiformes	Alcedinidae	*Halcyon pileata*
Coraciiformes	Alcedinidae	*Alcedo atthis*
Coraciiformes	Alcedinidae	*Ceyx erithaca*
Coraciiformes	Alcedinidae	*Megaceryle lugubris*
Coraciiformes	Alcedinidae	*Ceryle rudis*
Coraciiformes	Meropidae	*Nyctyornis athertoni*
Coraciiformes	Meropidae	*Merops orientalis* [#]
Coraciiformes	Meropidae	*Merops philippinus* [#]
Coraciiformes	Meropidae	*Merops leschenaulti* [#]
Bucerotiformes	Upupidae	*Upupa epops*
Bucerotiformes	Bucerotidae	*Rhyticeros undulatus*

Piciformes	Megalaimidae	*Psilopogon virens*	
Piciformes	Megalaimidae	*Psilopogon franklinii*	
Piciformes	Megalaimidae	*Psilopogon asiaticus*	
Piciformes	Megalaimidae	*Psilopogon haemacephalus*	
Piciformes	Indicatoridae	*Indicator xanthonotus*	
Piciformes	Picidae	*Picumnus innominatus* [#]	
Piciformes	Picidae	*Sasia ochracea* [#]	
Piciformes	Picidae	*Yungipicus canicapillus* [#]	
Piciformes	Picidae	*Picoides tridactylus* [#]	
Piciformes	Picidae	*Dryobates cathpharius*	
Piciformes	Picidae	*Dendrocopos hyperythrus* [#]	
Piciformes	Picidae	*Dendrocopos atratus*	
Piciformes	Picidae	*Dendrocopos darjellensis*	
Piciformes	Picidae	*Dendrocopos major* [#]	
Piciformes	Picidae	*Dendrocopos leucotos* [#]	
Piciformes	Picidae	*Dryocopus javensis*	
Piciformes	Picidae	*Dryocopus martius* [#]	
Piciformes	Picidae	*Chrysophlegma flavinucha* [#]	
Piciformes	Picidae	*Picus chlorolophus* [#]	
Piciformes	Picidae	*Picus canus* [#]	
Piciformes	Picidae	*Blythipicus pyrrhotis* [#]	
Piciformes	Picidae	*Micropternus brachyurus* [#]	
Falconiformes	Falconidae	*Microhierax melanoleucos*	
Falconiformes	Falconidae	*Falco tinnunculus* [#]	
Falconiformes	Falconidae	*Falco subbuteo* [#]	
Falconiformes	Falconidae	*Falco severus*	
Falconiformes	Falconidae	*Falco cherrug* [#]	
Psittaciformes	Psittaculidae	*Psittacula finschii*	
Psittaciformes	Psittaculidae	*Psittacula alexandri*	
Psittaciformes	Psittaculidae	*Psittacula derbiana*	E
Passeriformes	Eurylaimidae	*Psarisomus dalhousiae* [#]	
Passeriformes	Pittidae	*Hydrornis oatesi* [#]	
Passeriformes	Pittidae	*Pitta sordida* [#]	

Passeriformes	Vangidae	*Hemipus picatus*
Passeriformes	Vangidae	*Tephrodornis virgatus*
Passeriformes	Artamidae	*Artamus fuscus*
Passeriformes	Campephagidae	*Pericrocotus solaris* #
Passeriformes	Campephagidae	*Pericrocotus brevirostris* #
Passeriformes	Campephagidae	*Pericrocotus ethologus* #
Passeriformes	Campephagidae	*Pericrocotus speciosus* #
Passeriformes	Campephagidae	*Pericrocotus cantonensis* #
Passeriformes	Campephagidae	*Pericrocotus roseus* #
Passeriformes	Campephagidae	*Coracina macei* #
Passeriformes	Campephagidae	*Lalage melaschistos* #
Passeriformes	Laniidae	*Lanius cristatus* #
Passeriformes	Laniidae	*Lanius collurioides*
Passeriformes	Laniidae	*Lanius schach* #
Passeriformes	Laniidae	*Lanius tephronotus* #
Passeriformes	Laniidae	*Lanius sphenocercus* #
Passeriformes	Vireonidae	*Pteruthius xanthochlorus* #
Passeriformes	Vireonidae	*Pteruthius melanotis* #
Passeriformes	Vireonidae	*Pteruthius rufiventer* #
Passeriformes	Vireonidae	*Pteruthius aeralatus* #
Passeriformes	Vireonidae	*Pteruthius intermedius* #
Passeriformes	Vireonidae	*Erpornis zantholeuca* #
Passeriformes	Oriolidae	*Oriolus traillii* #
Passeriformes	Oriolidae	*Oriolus mellianus* #
Passeriformes	Oriolidae	*Oriolus chinensis* #
Passeriformes	Oriolidae	*Oriolus tenuirostris*
Passeriformes	Dicruridae	*Dicrurus aeneus* #
Passeriformes	Dicruridae	*Dicrurus remifer* #
Passeriformes	Dicruridae	*Dicrurus hottentottus* #
Passeriformes	Dicruridae	*Dicrurus leucophaeus* #
Passeriformes	Dicruridae	*Dicrurus macrocercus* #
Passeriformes	Rhipiduridae	*Rhipidura albicollis*
Passeriformes	Monarchidae	*Hypothymis azurea*

Passeriformes	Monarchidae	*Terpsiphone incei*	
Passeriformes	Corvidae	*Perisoreus internigrans* #	E
Passeriformes	Corvidae	*Garrulus glandarius* #	
Passeriformes	Corvidae	*Cyanopica cyanus* #	
Passeriformes	Corvidae	*Urocissa flavirostris* #	
Passeriformes	Corvidae	*Urocissa erythroryncha* #	
Passeriformes	Corvidae	*Cissa chinensis* #	
Passeriformes	Corvidae	*Dendrocitta formosae* #	
Passeriformes	Corvidae	*Dendrocitta frontalis* #	
Passeriformes	Corvidae	*Pica pica* #	
Passeriformes	Corvidae	*Pica bottanensis*	
Passeriformes	Corvidae	*Pica serica* #	
Passeriformes	Corvidae	*Nucifraga caryocatactes* #	
Passeriformes	Corvidae	*Pyrrhocorax pyrrhocorax* #	
Passeriformes	Corvidae	*Pyrrhocorax graculus* #	
Passeriformes	Corvidae	*Coloeus dauuricus* #	
Passeriformes	Corvidae	*Corvus splendens* #	
Passeriformes	Corvidae	*Corvus corone* #	
Passeriformes	Corvidae	*Corvus torquatus* #	
Passeriformes	Corvidae	*Corvus macrorhynchos* #	
Passeriformes	Corvidae	*Corvus levaillantii* #	
Passeriformes	Corvidae	*Corvus corax* #	
Passeriformes	Stenostiridae	*Chelidorhynx hypoxanthus* #	
Passeriformes	Stenostiridae	*Culicicapa ceylonensis* #	
Passeriformes	Paridae	*Cephalopyrus flammiceps*	
Passeriformes	Paridae	*Sylviparus modestus* #	
Passeriformes	Paridae	*Periparus rubidiventris* #	
Passeriformes	Paridae	*Periparus ater* #	
Passeriformes	Paridae	*Pardaliparus venustulus* #	
Passeriformes	Paridae	*Lophophanes dichrous* #	
Passeriformes	Paridae	*Poecile superciliosus* #	
Passeriformes	Paridae	*Poecile davidi* #	E
Passeriformes	Paridae	*Poecile hypermelaenus*	

Passeriformes	Paridae	*Poecile weigoldicus*
Passeriformes	Paridae	*Pseudopodoces humilis* #
Passeriformes	Paridae	*Parus minor*
Passeriformes	Paridae	*Parus monticolus* #
Passeriformes	Paridae	*Machlolophus spilonotus* #
Passeriformes	Alaudidae	*Alauda gulgula*
Passeriformes	Alaudidae	*Galerida cristata*
Passeriformes	Alaudidae	*Eremophila alpestris*
Passeriformes	Alaudidae	*Calandrella acutirostris*
Passeriformes	Alaudidae	*Calandrella dukhunensis*
Passeriformes	Alaudidae	*Melanocorypha maxima*
Passeriformes	Alaudidae	*Alaudala cheleensis*
Passeriformes	Pycnonotidae	*Alophoixus flaveolus* #
Passeriformes	Pycnonotidae	*Alcurus striatus* #
Passeriformes	Pycnonotidae	*Hemixos flavala* #
Passeriformes	Pycnonotidae	*Ixos mcclellandii* #
Passeriformes	Pycnonotidae	*Hypsipetes leucocephalus* #
Passeriformes	Pycnonotidae	*Rubigula flaviventris* #
Passeriformes	Pycnonotidae	*Spizixos canifrons* #
Passeriformes	Pycnonotidae	*Spizixos semitorques* #
Passeriformes	Pycnonotidae	*Pycnonotus flavescens* #
Passeriformes	Pycnonotidae	*Pycnonotus xanthorrhous* #
Passeriformes	Pycnonotidae	*Pycnonotus sinensis* #
Passeriformes	Pycnonotidae	*Pycnonotus jocosus* #
Passeriformes	Pycnonotidae	*Pycnonotus cafer* #
Passeriformes	Pycnonotidae	*Pycnonotus aurigaster* #
Passeriformes	Hirundinidae	*Riparia diluta*
Passeriformes	Hirundinidae	*Riparia chinensis*
Passeriformes	Hirundinidae	*Ptyonoprogne rupestris* #
Passeriformes	Hirundinidae	*Hirundo rustica* #
Passeriformes	Hirundinidae	*Delichon dasypus* #
Passeriformes	Hirundinidae	*Delichon nipalense* #
Passeriformes	Hirundinidae	*Cecropis daurica* #

Passeriformes	Hirundinidae	*Cecropis striolata* [#]	
Passeriformes	Pnoepygidae	*Pnoepyga albiventer*	
Passeriformes	Pnoepygidae	*Pnoepyga pusilla*	
Passeriformes	Cettiidae	*Abroscopus albogularis* [#]	
Passeriformes	Cettiidae	*Abroscopus schisticeps* [#]	
Passeriformes	Cettiidae	*Phyllergates cucullatus* [#]	
Passeriformes	Cettiidae	*Tickellia hodgsoni* [#]	
Passeriformes	Cettiidae	*Horornis fortipes* [#]	
Passeriformes	Cettiidae	*Horornis acanthizoides* [#]	
Passeriformes	Cettiidae	*Horornis flavolivaceus* [#]	
Passeriformes	Cettiidae	*Tesia cyaniventer* [#]	
Passeriformes	Cettiidae	*Tesia olivea* [#]	
Passeriformes	Cettiidae	*Cettia major* [#]	
Passeriformes	Cettiidae	*Cettia brunnifrons* [#]	
Passeriformes	Cettiidae	*Cettia castaneocoronata* [#]	
Passeriformes	Cettiidae	*Hemitesia pallidipes* [#]	
Passeriformes	Aegithalidae	*Leptopoecile sophiae* [#]	
Passeriformes	Aegithalidae	*Leptopoecile elegans* [#]	
Passeriformes	Aegithalidae	*Aegithalos glaucogularis*	
Passeriformes	Aegithalidae	*Aegithalos concinnus* [#]	
Passeriformes	Aegithalidae	*Aegithalos bonvaloti*	E
Passeriformes	Aegithalidae	*Aegithalos fuliginosus* [#]	
Passeriformes	Phylloscopidae	*Phylloscopus pulcher* [#]	
Passeriformes	Phylloscopidae	*Phylloscopus maculipennis* [#]	
Passeriformes	Phylloscopidae	*Phylloscopus humei* [#]	
Passeriformes	Phylloscopidae	*Phylloscopus yunnanensis* [#]	
Passeriformes	Phylloscopidae	*Phylloscopus chloronotus* [#]	
Passeriformes	Phylloscopidae	*Phylloscopus forresti* [#]	E
Passeriformes	Phylloscopidae	*Phylloscopus armandii* [#]	
Passeriformes	Phylloscopidae	*Phylloscopus affinis* [#]	
Passeriformes	Phylloscopidae	*Phylloscopus fuligiventer* [#]	
Passeriformes	Phylloscopidae	*Phylloscopus fuscatus* [#]	
Passeriformes	Phylloscopidae	*Phylloscopus subaffinis* [#]	

Passeriformes	Phylloscopidae	*Phylloscopus intermedius* #	
Passeriformes	Phylloscopidae	*Phylloscopus poliogenys* #	
Passeriformes	Phylloscopidae	*Phylloscopus tephrocephalus* #	
Passeriformes	Phylloscopidae	*Phylloscopus valentini* #	
Passeriformes	Phylloscopidae	*Phylloscopus soror* #	
Passeriformes	Phylloscopidae	*Phylloscopus omeiensis* #	
Passeriformes	Phylloscopidae	*Phylloscopus trochiloides* #	
Passeriformes	Phylloscopidae	*Phylloscopus emeiensis* #	E
Passeriformes	Phylloscopidae	*Phylloscopus magnirostris* #	
Passeriformes	Phylloscopidae	*Phylloscopus castaniceps* #	
Passeriformes	Phylloscopidae	*Phylloscopus ricketti* #	
Passeriformes	Phylloscopidae	*Phylloscopus cantator* #	
Passeriformes	Phylloscopidae	*Phylloscopus reguloides* #	
Passeriformes	Phylloscopidae	*Phylloscopus ogilviegranti* #	
Passeriformes	Phylloscopidae	*Phylloscopus intensior* #	
Passeriformes	Acrocephalidae	*Acrocephalus stentoreus* #	
Passeriformes	Locustellidae	*Locustella luteoventris* #	
Passeriformes	Locustellidae	*Locustella tacsanowskia* #	
Passeriformes	Locustellidae	*Locustella thoracica* #	
Passeriformes	Locustellidae	*Locustella mandelli* #	
Passeriformes	Locustellidae	*Locustella chengi* #	
Passeriformes	Locustellidae	*Megalurus palustris* #	
Passeriformes	Cisticolidae	*Cisticola juncidis*	
Passeriformes	Cisticolidae	*Cisticola exilis*	
Passeriformes	Cisticolidae	*Prinia crinigera*	
Passeriformes	Cisticolidae	*Prinia striata*	
Passeriformes	Cisticolidae	*Prinia superciliaris*	
Passeriformes	Cisticolidae	*Prinia rufescens*	
Passeriformes	Cisticolidae	*Prinia hodgsonii*	
Passeriformes	Cisticolidae	*Prinia flaviventris*	
Passeriformes	Cisticolidae	*Prinia inornata*	
Passeriformes	Cisticolidae	*Orthotomus sutorius*	
Passeriformes	Timaliidae	*Timalia pileata* #	

Passeriformes	Timaliidae	*Mixornis gularis* #
Passeriformes	Timaliidae	*Cyanoderma chrysaeum* #
Passeriformes	Timaliidae	*Cyanoderma ruficeps* #
Passeriformes	Timaliidae	*Cyanoderma ambiguum* #
Passeriformes	Timaliidae	*Spelaeornis troglodytoides* #
Passeriformes	Timaliidae	*Spelaeornis reptatus* #
Passeriformes	Timaliidae	*Pomatorhinus ferruginosus* #
Passeriformes	Timaliidae	*Pomatorhinus superciliaris* #
Passeriformes	Timaliidae	*Pomatorhinus ruficollis* #
Passeriformes	Timaliidae	*Erythrogenys gravivox* #
Passeriformes	Timaliidae	*Stachyris nigriceps* #
Passeriformes	Timaliidae	*Stachyris roberti* #
Passeriformes	Pellorneidae	*Schoeniparus cinereus* #
Passeriformes	Pellorneidae	*Schoeniparus castaneceps* #
Passeriformes	Pellorneidae	*Schoeniparus dubius* #
Passeriformes	Pellorneidae	*Schoeniparus brunneus* #
Passeriformes	Pellorneidae	*Pellorneum ruficeps* #
Passeriformes	Pellorneidae	*Pellorneum tickelli* #
Passeriformes	Pellorneidae	*Napothera malacoptila* #
Passeriformes	Alcippeidae	*Alcippe davidi* #
Passeriformes	Alcippeidae	*Alcippe fratercula* #
Passeriformes	Leiothrichidae	*Grammatoptila striata* #
Passeriformes	Leiothrichidae	*Cutia nipalensis* #
Passeriformes	Leiothrichidae	*Trochalopteron subunicolor* #
Passeriformes	Leiothrichidae	*Trochalopteron squamatum* #
Passeriformes	Leiothrichidae	*Trochalopteron affine* #
Passeriformes	Leiothrichidae	*Trochalopteron henrici* #
Passeriformes	Leiothrichidae	*Trochalopteron elliotii* #
Passeriformes	Leiothrichidae	*Trochalopteron milnei* #
Passeriformes	Leiothrichidae	*Trochalopteron erythrocephalum* #
Passeriformes	Leiothrichidae	*Trochalopteron chrysopterum* #
Passeriformes	Leiothrichidae	*Trochalopteron formosum* #
Passeriformes	Leiothrichidae	*Heterophasia picaoides* #

Passeriformes	Leiothrichidae	*Heterophasia pulchella* #	
Passeriformes	Leiothrichidae	*Heterophasia gracilis* #	
Passeriformes	Leiothrichidae	*Heterophasia desgodinsi* #	
Passeriformes	Leiothrichidae	*Actinodura waldeni* #	
Passeriformes	Leiothrichidae	*Actinodura souliei* #	
Passeriformes	Leiothrichidae	*Actinodura cyanouroptera* #	
Passeriformes	Leiothrichidae	*Actinodura strigula* #	
Passeriformes	Leiothrichidae	*Actinodura egertoni* #	
Passeriformes	Leiothrichidae	*Leiothrix lutea* #	
Passeriformes	Leiothrichidae	*Leiothrix argentauris* #	
Passeriformes	Leiothrichidae	*Minla ignotincta* #	
Passeriformes	Leiothrichidae	*Leioptila annectens* #	
Passeriformes	Leiothrichidae	*Liocichla omeiensis* #	E
Passeriformes	Leiothrichidae	*Liocichla phoenicea* #	
Passeriformes	Leiothrichidae	*Liocichla ripponi* #	
Passeriformes	Leiothrichidae	*Garrulax merulinus* #	
Passeriformes	Leiothrichidae	*Garrulax canorus* #	
Passeriformes	Leiothrichidae	*Garrulax monileger* #	
Passeriformes	Leiothrichidae	*Garrulax leucolophus* #	
Passeriformes	Leiothrichidae	*Garrulax maesi* #	
Passeriformes	Leiothrichidae	*Ianthocincla sukatschewi* #	E
Passeriformes	Leiothrichidae	*Ianthocincla cineracea* #	
Passeriformes	Leiothrichidae	*Ianthocincla ocellata* #	
Passeriformes	Leiothrichidae	*Ianthocincla maxima* #	E
Passeriformes	Leiothrichidae	*Ianthocincla bieti* #	E
Passeriformes	Leiothrichidae	*Ianthocincla lunulata* #	
Passeriformes	Leiothrichidae	*Pterorhinus sannio* #	
Passeriformes	Leiothrichidae	*Pterorhinus perspicillatus* #	
Passeriformes	Leiothrichidae	*Pterorhinus pectoralis* #	
Passeriformes	Leiothrichidae	*Pterorhinus davidi* #	
Passeriformes	Leiothrichidae	*Pterorhinus lanceolatus* #	
Passeriformes	Leiothrichidae	*Pterorhinus koslowi* #	E
Passeriformes	Leiothrichidae	*Pterorhinus albogularis* #	

Passeriformes	Leiothrichidae	*Pterorhinus caerulatus* #	
Passeriformes	Leiothrichidae	*Pterorhinus berthemyi* #	
Passeriformes	Paradoxornithidae	*Myzornis pyrrhoura* #	
Passeriformes	Paradoxornithidae	*Lioparus chrysotis* #	
Passeriformes	Paradoxornithidae	*Moupinia poecilotis* #	E
Passeriformes	Paradoxornithidae	*Fulvetta vinipectus* #	
Passeriformes	Paradoxornithidae	*Fulvetta striaticollis* #	E
Passeriformes	Paradoxornithidae	*Fulvetta ruficapilla* #	
Passeriformes	Paradoxornithidae	*Fulvetta cinereiceps* #	
Passeriformes	Paradoxornithidae	*Fulvetta manipurensis* #	
Passeriformes	Paradoxornithidae	*Chrysomma sinense* #	
Passeriformes	Paradoxornithidae	*Conostoma aemodium* #	
Passeriformes	Paradoxornithidae	*Cholornis paradoxus* #	
Passeriformes	Paradoxornithidae	*Cholornis unicolor* #	
Passeriformes	Paradoxornithidae	*Sinosuthora conspicillata* #	
Passeriformes	Paradoxornithidae	*Sinosuthora webbiana* #	
Passeriformes	Paradoxornithidae	*Sinosuthora alphonsiana* #	
Passeriformes	Paradoxornithidae	*Sinosuthora brunnea* #	
Passeriformes	Paradoxornithidae	*Sinosuthora zappeyi* #	
Passeriformes	Paradoxornithidae	*Sinosuthora przewalskii*	E
Passeriformes	Paradoxornithidae	*Suthora fulvifrons* #	
Passeriformes	Paradoxornithidae	*Suthora nipalensis* #	
Passeriformes	Paradoxornithidae	*Suthora verreauxi* #	
Passeriformes	Paradoxornithidae	*Chleuasicus atrosuperciliaris* #	
Passeriformes	Paradoxornithidae	*Psittiparus bakeri* #	
Passeriformes	Paradoxornithidae	*Psittiparus gularis* #	
Passeriformes	Paradoxornithidae	*Paradoxornis guttaticollis* #	
Passeriformes	Zosteropidae	*Parayuhina diademata*	
Passeriformes	Zosteropidae	*Staphida castaniceps*	
Passeriformes	Zosteropidae	*Staphida torqueola*	
Passeriformes	Zosteropidae	*Yuhina nigrimenta* #	
Passeriformes	Zosteropidae	*Yuhina flavicollis* #	
Passeriformes	Zosteropidae	*Yuhina bakeri* #	

Passeriformes	Zosteropidae	*Yuhina gularis* #	
Passeriformes	Zosteropidae	*Yuhina occipitalis* #	
Passeriformes	Zosteropidae	*Zosterops simplex*	
Passeriformes	Zosteropidae	*Zosterops palpebrosus*	
Passeriformes	Regulidae	*Regulus regulus* #	
Passeriformes	Elachuridae	*Elachura formosa*	
Passeriformes	Troglodytidae	*Troglodytes troglodytes*	
Passeriformes	Sittidae	*Sitta europaea* #	
Passeriformes	Sittidae	*Sitta nagaensis* #	
Passeriformes	Sittidae	*Sitta himalayensis* #	
Passeriformes	Sittidae	*Sitta yunnanensis* #	E
Passeriformes	Sittidae	*Sitta villosa* #	
Passeriformes	Sittidae	*Sitta przewalskii* #	
Passeriformes	Sittidae	*Sitta frontalis* #	
Passeriformes	Sittidae	*Sitta magna* #	
Passeriformes	Sittidae	*Sitta formosa* #	
Passeriformes	Tichodromidae	*Tichodroma muraria*	
Passeriformes	Certhiidae	*Certhia hodgsoni* #	
Passeriformes	Certhiidae	*Certhia himalayana* #	
Passeriformes	Certhiidae	*Certhia nipalensis* #	
Passeriformes	Certhiidae	*Certhia manipurensis* #	
Passeriformes	Certhiidae	*Certhia tianquanensis* #	E
Passeriformes	Sturnidae	*Acridotheres grandis* #	
Passeriformes	Sturnidae	*Acridotheres cristatellus* #	
Passeriformes	Sturnidae	*Acridotheres albocinctus* #	
Passeriformes	Sturnidae	*Acridotheres tristis* #	
Passeriformes	Sturnidae	*Spodiopsar sericeus* #	
Passeriformes	Sturnidae	*Gracupica nigricollis* #	
Passeriformes	Sturnidae	*Sturnia malabarica* #	
Passeriformes	Turdidae	*Grandala coelicolor*	
Passeriformes	Turdidae	*Cochoa purpurea*	
Passeriformes	Turdidae	*Zoothera dixoni* #	
Passeriformes	Turdidae	*Zoothera mollissima* #	

Passeriformes	Turdidae	*Zoothera salimalii*	
Passeriformes	Turdidae	*Zoothera griseiceps*	
Passeriformes	Turdidae	*Zoothera monticola* #	
Passeriformes	Turdidae	*Zoothera marginata* #	
Passeriformes	Turdidae	*Zoothera aurea*	
Passeriformes	Turdidae	*Zoothera dauma* #	
Passeriformes	Turdidae	*Geokichla citrina* #	
Passeriformes	Turdidae	*Turdus mupinensis* #	
Passeriformes	Turdidae	*Turdus mandarinus*	
Passeriformes	Turdidae	*Turdus boulboul* #	
Passeriformes	Turdidae	*Turdus dissimilis* #	
Passeriformes	Turdidae	*Turdus kessleri* #	
Passeriformes	Turdidae	*Turdus rubrocanus* #	
Passeriformes	Turdidae	*Turdus albocinctus* #	
Passeriformes	Muscicapidae	*Copsychus saularis* #	
Passeriformes	Muscicapidae	*Copsychus malabaricus* #	
Passeriformes	Muscicapidae	*Muscicapa sibirica* #	
Passeriformes	Muscicapidae	*Muscicapa muttui* #	
Passeriformes	Muscicapidae	*Muscicapa ferruginea* #	
Passeriformes	Muscicapidae	*Cyornis unicolor* #	
Passeriformes	Muscicapidae	*Cyornis whitei*	
Passeriformes	Muscicapidae	*Cyornis rubeculoides* #	
Passeriformes	Muscicapidae	*Cyornis glaucicomans*	
Passeriformes	Muscicapidae	*Niltava davidi* #	
Passeriformes	Muscicapidae	*Niltava sundara* #	
Passeriformes	Muscicapidae	*Niltava grandis* #	
Passeriformes	Muscicapidae	*Niltava macgrigoriae* #	
Passeriformes	Muscicapidae	*Eumyias thalassinus* #	
Passeriformes	Muscicapidae	*Heteroxenicus stellatus*	
Passeriformes	Muscicapidae	*Brachypteryx hyperythra*	E
Passeriformes	Muscicapidae	*Brachypteryx leucophris* #	
Passeriformes	Muscicapidae	*Brachypteryx cruralis*	
Passeriformes	Muscicapidae	*Larvivora brunnea* #	

Passeriformes	Muscicapidae	*Larvivora ruficeps*
Passeriformes	Muscicapidae	*Luscinia phaenicuroides* #
Passeriformes	Muscicapidae	*Calliope tschebaiewi*
Passeriformes	Muscicapidae	*Calliope pectardens* #
Passeriformes	Muscicapidae	*Calliope obscura*
Passeriformes	Muscicapidae	*Myiomela leucura* #
Passeriformes	Muscicapidae	*Tarsiger indicus* #
Passeriformes	Muscicapidae	*Tarsiger hyperythrus* #
Passeriformes	Muscicapidae	*Tarsiger rufilatus*
Passeriformes	Muscicapidae	*Tarsiger chrysaeus* #
Passeriformes	Muscicapidae	*Enicurus scouleri* #
Passeriformes	Muscicapidae	*Enicurus schistaceus* #
Passeriformes	Muscicapidae	*Enicurus leschenaulti* #
Passeriformes	Muscicapidae	*Enicurus maculatus* #
Passeriformes	Muscicapidae	*Myophonus caeruleus* #
Passeriformes	Muscicapidae	*Cinclidium frontale* #
Passeriformes	Muscicapidae	*Ficedula zanthopygia* #
Passeriformes	Muscicapidae	*Ficedula erithacus* #
Passeriformes	Muscicapidae	*Ficedula hodgsoni* #
Passeriformes	Muscicapidae	*Ficedula strophiata* #
Passeriformes	Muscicapidae	*Ficedula sapphira* #
Passeriformes	Muscicapidae	*Ficedula superciliaris* #
Passeriformes	Muscicapidae	*Ficedula westermanni* #
Passeriformes	Muscicapidae	*Ficedula tricolor* #
Passeriformes	Muscicapidae	*Ficedula hyperythra* #
Passeriformes	Muscicapidae	*Phoenicurus ochruros* #
Passeriformes	Muscicapidae	*Phoenicurus hodgsoni* #
Passeriformes	Muscicapidae	*Phoenicurus schisticeps* #
Passeriformes	Muscicapidae	*Phoenicurus auroreus* #
Passeriformes	Muscicapidae	*Phoenicurus erythrogastrus* #
Passeriformes	Muscicapidae	*Phoenicurus frontalis* #
Passeriformes	Muscicapidae	*Phoenicurus fuliginosus* #
Passeriformes	Muscicapidae	*Phoenicurus leucocephalus* #

Passeriformes	Muscicapidae	*Monticola solitarius* #
Passeriformes	Muscicapidae	*Monticola rufiventris* #
Passeriformes	Muscicapidae	*Saxicola maurus*
Passeriformes	Muscicapidae	*Saxicola caprata* #
Passeriformes	Muscicapidae	*Saxicola ferreus* #
Passeriformes	Cinclidae	*Cinclus cinclus*
Passeriformes	Cinclidae	*Cinclus pallasii*
Passeriformes	Chloropseidae	*Chloropsis hardwickii*
Passeriformes	Chloropseidae	*Chloropsis lazulina*
Passeriformes	Dicaeidae	*Dicaeum chrysorrheum* #
Passeriformes	Dicaeidae	*Dicaeum melanozanthum* #
Passeriformes	Dicaeidae	*Dicaeum minullum*
Passeriformes	Dicaeidae	*Dicaeum ignipectus* #
Passeriformes	Nectariniidae	*Cinnyris asiaticus*
Passeriformes	Nectariniidae	*Aethopyga gouldiae*
Passeriformes	Nectariniidae	*Aethopyga nipalensis*
Passeriformes	Nectariniidae	*Aethopyga saturata*
Passeriformes	Nectariniidae	*Aethopyga siparaja*
Passeriformes	Nectariniidae	*Aethopyga ignicauda*
Passeriformes	Nectariniidae	*Arachnothera magna*
Passeriformes	Passeridae	*Petronia petronia* #
Passeriformes	Passeridae	*Onychostruthus taczanowskii* #
Passeriformes	Passeridae	*Montifringilla henrici* #
Passeriformes	Passeridae	*Montifringilla adamsi* #
Passeriformes	Passeridae	*Pyrgilauda ruficollis* #
Passeriformes	Passeridae	*Pyrgilauda blanfordi* #
Passeriformes	Passeridae	*Passer cinnamomeus* #
Passeriformes	Passeridae	*Passer montanus* #
Passeriformes	Passeridae	*Passer domesticus* #
Passeriformes	Estrildidae	*Lonchura punctulata* #
Passeriformes	Estrildidae	*Lonchura striata* #
Passeriformes	Estrildidae	*Lonchura atricapilla*
Passeriformes	Estrildidae	*Amandava amandava* #

Passeriformes	Prunellidae	*Prunella collaris* #	
Passeriformes	Prunellidae	*Prunella rubeculoides* #	
Passeriformes	Prunellidae	*Prunella strophiata* #	
Passeriformes	Prunellidae	*Prunella fulvescens* #	
Passeriformes	Prunellidae	*Prunella immaculata* #	
Passeriformes	Motacillidae	*Dendronanthus indicus*	
Passeriformes	Motacillidae	*Motacilla citreola* #	
Passeriformes	Motacillidae	*Motacilla alba* #	
Passeriformes	Motacillidae	*Anthus rufulus*	
Passeriformes	Motacillidae	*Anthus hodgsoni*	
Passeriformes	Motacillidae	*Anthus roseatus*	
Passeriformes	Motacillidae	*Anthus sylvanus*	
Passeriformes	Urocynchramidae	*Urocynchramus pylzowi*	
Passeriformes	Fringillidae	*Mycerobas affinis* #	
Passeriformes	Fringillidae	*Mycerobas melanozanthos* #	
Passeriformes	Fringillidae	*Mycerobas carnipes* #	
Passeriformes	Fringillidae	*Pyrrhula nipalensis* #	
Passeriformes	Fringillidae	*Pyrrhula erythaca* #	
Passeriformes	Fringillidae	*Agraphospiza rubescens* #	
Passeriformes	Fringillidae	*Pyrrhoplectes epauletta* #	
Passeriformes	Fringillidae	*Procarduelis nipalensis* #	
Passeriformes	Fringillidae	*Leucosticte nemoricola* #	
Passeriformes	Fringillidae	*Leucosticte brandti* #	
Passeriformes	Fringillidae	*Carpodacus erythrinus* #	
Passeriformes	Fringillidae	*Carpodacus sipahi* #	
Passeriformes	Fringillidae	*Carpodacus rubicilloides* #	
Passeriformes	Fringillidae	*Carpodacus rubicilla* #	
Passeriformes	Fringillidae	*Carpodacus pulcherrimus* #	
Passeriformes	Fringillidae	*Carpodacus waltoni*	
Passeriformes	Fringillidae	*Carpodacus edwardsii* #	
Passeriformes	Fringillidae	*Carpodacus lepidus* #	
Passeriformes	Fringillidae	*Carpodacus verreauxii*	E
Passeriformes	Fringillidae	*Carpodacus vinaceus* #	

Passeriformes	Fringillidae	*Carpodacus sibiricus* #	
Passeriformes	Fringillidae	*Carpodacus trifasciatus* #	
Passeriformes	Fringillidae	*Carpodacus dubius*	
Passeriformes	Fringillidae	*Carpodacus puniceus* #	
Passeriformes	Fringillidae	*Carpodacus subhimachalus* #	
Passeriformes	Fringillidae	*Chloris sinica* #	
Passeriformes	Fringillidae	*Chloris ambigua* #	
Passeriformes	Fringillidae	*Linaria flavirostris* #	
Passeriformes	Fringillidae	*Loxia curvirostra* #	
Passeriformes	Fringillidae	*Spinus thibetanus* #	
Passeriformes	Emberizidae	*Emberiza lathami* #	
Passeriformes	Emberizidae	*Emberiza siemsseni* #	
Passeriformes	Emberizidae	*Emberiza godlewskii* #	
Passeriformes	Emberizidae	*Emberiza cioides* #	
Passeriformes	Emberizidae	*Emberiza fucata* #	
Passeriformes	Emberizidae	*Emberiza elegans* #	
Passeriformes	Emberizidae	*Emberiza koslowi*	E
Passeriformes	Emberizidae	*Emberiza spodocephala* #	
	**SUBTOTAL**	**638**	**25**
	**TOTAL**	**1160 species**	**205 endemics**

Table S2. Global and regional sampling of clades. Sampling 121 diverse clades of terrestrial vertebrates in Hengduan Mountains as well as their close relatives. * = New phylogenies were constructed in this study for these 34 clades.

ID	Class	Clades	Global			Hengduan Mountains		
			total	sampled	%	total	sampled	%
**1**	Amphibia	*Batrachuperus*	6	5	83	5	4	80
**2**	Amphibia	*Tylototriton*	37	14	38	4	4	100
**3**	Amphibia	*Bombina*	7	7	100	1	1	100
**4**	Amphibia	*Bufo*	24	14	58	7	5	71
**5**	Amphibia	*Duttaphrynus*	28	10	36	2	2	100
**6**	Amphibia	*Nanorana*	32	15	47	7	6	86
**7**	Amphibia	*Hyla*	15	11	73	1	1	100
**8**	Amphibia	*Oreolalax*	19	14	74	7	6	86
**9**	Amphibia	*Scutiger**	26	20	77	13	12	92
**10**	Amphibia	*Brachytarsophrys*	8	4	50	1	1	100
**11**	Amphibia	*Kaloula*	19	12	63	2	2	100
**12**	Amphibia	*Odorrana*	62	41	66	5	4	80
**13**	Amphibia	*Amolops**	76	65	86	13	13	100
**14**	Amphibia	*Rana*	58	45	78	5	5	100
**15**	Amphibia	*Polypedates*	25	13	52	1	1	100
**16**	Amphibia	*Zhangixalus*	40	18	45	6	6	100
	**SUBTOTAL**			**308**			**73**	
**17**	Reptilia	*Ptyas*	13	6	46	4	3	75
**18**	Reptilia	*Orthriophis*	4	4	100	1	1	100
**19**	Reptilia	*Elaphe*	13	11	85	4	4	100
**20**	Reptilia	*Euprepiophis*	3	3	100	2	2	100
**21**	Reptilia	*Lycodon**	72	48	67	8	8	100
**22**	Reptilia	*Hebius**	49	30	61	8	8	100
**23**	Reptilia	*Rhabdophis**	28	22	79	6	6	100
**24**	Reptilia	*Pareas**	27	27	100	6	6	100
**25**	Reptilia	*Sibynophis*	9	5	56	2	2	100
**26**	Reptilia	*Gloydius**	25	25	100	9	9	100
**27**	Reptilia	*Protobothrops*	15	12	80	4	4	100

**28**	Reptilia	*Trimeresurus*	47	27	57	1	1	100
**29**	Reptilia	*Calotes*	29	12	41	2	2	100
**30**	Reptilia	*Diploderma**	42	39	93	29	29	100
**31**	Reptilia	*Takydromus*	24	17	71	2	2	100
**32**	Reptilia	*Plestiodon*	51	41	80	3	3	100
**33**	Reptilia	*Scincella**	38	23	61	5	5	100
	**SUBTOTAL**			**352**			**95**	
**34**	Mammalia	*Tupaia*	19	9	47	1	1	100
**35**	Mammalia	*Macaca*	25	21	84	5	4	80
**36**	Mammalia	*Rhinopithecus*	5	5	100	3	3	100
**37**	Mammalia	*Trachypithecus*	22	13	59	2	2	100
**38**	Mammalia	*Nomascus*	7	6	86	1	1	100
**39**	Mammalia	*Lepus*	32	26	81	2	2	100
**40**	Mammalia	*Ochotona**	34	34	100	11	11	100
**41**	Mammalia	*Sicista*	19	6	32	1	1	100
**42**	Mammalia	Erinaceidae*	26	24	92	4	4	100
**43**	Mammalia	Spalacidae*	29	23	79	7	7	100
**44**	Mammalia	*Apodemus*	22	17	77	5	4	80
**45**	Mammalia	*Mus*	42	30	71	3	3	100
**46**	Mammalia	*Niviventer**	24	22	92	9	9	100
**47**	Mammalia	*Rattus**	69	37	54	4	4	100
**48**	Mammalia	Arvicolinae*	176	152	86	25	25	100
**49**	Mammalia	Hystricidae	11	7	64	2	2	100
**50**	Mammalia	Sciuridae*	311	240	77	24	22	92
**51**	Mammalia	Talpidae*	60	54	90	11	10	91
**52**	Mammalia	*Crocidura*	217	105	48	6	4	67
**53**	Mammalia	Soricinae*	192	124	65	25	22	88
**54**	Mammalia	Pteropodidae	197	121	61	5	5	100
**55**	Mammalia	*Hipposideros*	67	43	64	3	3	100
**56**	Mammalia	*Rhinolophus*	108	54	50	13	11	85
**57**	Mammalia	*Miniopterus*	41	20	49	2	2	100
**58**	Mammalia	*Murina*	40	29	73	4	3	75
**59**	Mammalia	*Myotis*	136	83	61	13	11	85

**60**	Mammalia	*Nyctalus*	8	6	75	1	1	100
**61**	Mammalia	*Scotophilus*	19	14	74	1	1	100
**62**	Mammalia	*Sus*	8	6	75	1	1	100
**63**	Mammalia	Cervidae*	55	44	80	9	9	100
**64**	Mammalia	Bovidae	150	129	86	9	7	78
**65**	Mammalia	*Moschus*	7	6	86	3	3	100
**66**	Mammalia	Felidae	45	34	76	10	9	90
**67**	Mammalia	*Urva*	9	7	78	1	1	100
**68**	Mammalia	*Vulpes*	12	10	83	2	2	100
**69**	Mammalia	Ursidae	8	8	100	3	3	100
**70**	Mammalia	*Meles*	4	3	75	1	1	100
**71**	Mammalia	*Martes*	8	6	75	2	2	100
**72**	Mammalia	*Mustela*	15	12	80	6	5	83
**73**	Mammalia	Viverridae*	36	31	86	4	4	100
	**SUBTOTAL**			**1621**			**225**	
**74**	Aves	Phasianidae	188	129	69	29	28	97
**75**	Aves	Accipitridae + Pandionidae	255	175	68	25	21	84
**76**	Aves	*Certhia*	9	9	100	5	5	100
**77**	Aves	*Harpactes*	10	10	100	2	2	100
**78**	Aves	Campephagidae	94	69	73	8	8	100
**79**	Aves	Dicruridae	30	18	60	5	5	100
**80**	Aves	Cuculidae	150	126	84	16	15	94
**81**	Aves	Meropidae	31	23	74	4	3	75
**82**	Aves	Falconidae	65	45	69	5	3	60
**83**	Aves	Phylloscopidae	81	64	79	26	26	100
**84**	Aves	Dicaeidae	50	31	62	4	3	75
**85**	Aves	Eurylaimidae	9	8	89	1	1	100
**86**	Aves	Emberizidae	45	36	80	8	7	88
**87**	Aves	Muscicapidae	352	231	66	57	47	82
**88**	Aves	Paridae	64	42	66	14	10	71
**89**	Aves	Aegithalidae	13	9	69	6	4	67
**90**	Aves	Sturnidae	128	94	73	7	7	100
**91**	Aves	Regulidae	6	6	100	1	1	100

**92**	Aves	Stenostiridae	9	9	100	2	2	100
**93**	Aves	*Lanius*	30	20	67	5	4	80
**94**	Aves	*Oriolus*	30	25	83	4	3	75
**95**	Aves	Pittidae	44	28	64	2	2	100
**96**	Aves	Picidae	239	137	57	17	13	76
**97**	Aves	Turdidae	175	133	76	18	12	67
**98**	Aves	Hirundinidae	89	77	87	8	6	75
**99**	Aves	Jacanidae	8	7	88	1	1	100
**100**	Aves	*Acrocephalus*	42	29	69	1	1	100
**101**	Aves	*Yuhina*	7	6	86	5	5	100
**102**	Aves	Estrildidae	139	86	62	4	3	75
**103**	Aves	*Tadorna*	6	5	83	1	1	100
**104**	Aves	*Ixobrychus*	10	6	60	2	2	100
**105**	Aves	*Motacilla*	13	9	69	2	2	100
**106**	Aves	*Tringa*	13	12	92	1	1	100
**107**	Aves	*Streptopelia*	15	11	73	2	2	100
**108**	Aves	Leiothrichidae*	133	125	94	47	47	100
**109**	Aves	Paradoxornithidae*	37	33	89	25	24	96
**110**	Aves	Passeridae*	43	30	70	9	9	100
**111**	Aves	Sittidae*	29	27	93	9	9	100
**112**	Aves	Corvidae*	135	121	90	21	20	95
**113**	Aves	Fringillidae*	234	170	73	30	27	90
**114**	Aves	Vireonidae*	62	53	85	6	6	100
**115**	Aves	Timaliidae*	58	50	86	13	13	100
**116**	Aves	Pellorneidae*	65	54	83	7	7	100
**117**	Aves	Alcippeidae*	10	10	100	2	2	100
**118**	Aves	Cettiidae*	31	29	94	13	13	100
**119**	Aves	Pycnonotidae* (Asian clade)	99	84	85	14	14	100
**120**	Aves	Locustellidae*	67	58	87	6	6	100
**121**	Aves	Prunellidae*	12	12	100	5	5	100
		**SUBTOTAL**		**2581**			**458**	
		**TOTAL**		**4862**			**851**	

Table S3. Summary of the biogeographic events estimated from 1,000 replicate simulations of biogeographic histories designed to account for phylogenetic uncertainty. Here, *in situ* = *in situ* speciation, Col = Colonization.

	Terrestrial vertebrates		Amphibians		Reptiles		Mammals		Birds	
	in situ	Col	*in situ*	Col	*in situ*	Col	*in situ*	Col	*in situ*	Col
25% quantile	423	556	53	30	87	38	124	137	149	342
Median	437	566	56	33	90	41	130	142	159	351
75% quantile	450	578	60	37	93	44	136	147	168	359

Table S4. Chronology of inferred biogeographic events of amphibians. The results are estimated from 1,000 replicate simulations of biogeographic histories.

Time (Ma)	*in situ* speciation			Colonization		
	25% quantile	Median	75% quantile	25% quantile	Median	75% quantile
0	53	56	60	30	33	37
1	50	53	57	28	31	35
2	43	46	50	26	29	32
3	33	36	39	24	26	29
4	27	30	33	21	24	26
5	25	27	31	19	21	24
6	21	23	26	16	19	21
7	19	21	24	15	17	19
8	13	15.5	18	13	15	17
9	10	12	14	11	13	15
10	6	8	10	10	12	14
11	5	7	8	8	10	12
12	4	5	7	7	9	10
13	3	4	6	6	7	9
14	2	4	5	4	6	8
15	2	3	4	4	5	6
16	1	2	3	3	4	5
17	1	2	3	2	3	4
18	1	1	2	2	2	4
19	0	1	1	1	2	3
20	0	0	0	1	2	2
21	0	0	0	0	1	2
22	0	0	0	0	1	2
23	0	0	0	0	1	1
24	0	0	0	0	0	1
25	0	0	0	0	0	1
26	0	0	0	0	0	1
27	0	0	0	0	0	1
28	0	0	0	0	0	1
29	0	0	0	0	0	1

Table S5. Chronology of inferred biogeographic events of non-avian reptiles. The results are estimated from 1,000 replicate simulations of biogeographic histories.

Time (Ma)	*in situ* speciation			Colonization		
	25% quantile	Median	75% quantile	25% quantile	Median	75% quantile
0	87	90	93	38	41	44
1	84	87	90	35	38	42
2	79	82	85	32	35	38
3	65	68	71	29	32	35
4	56	59	62	25	28	31
5	43	46	49	21	24	27
6	36	39	41	18	21	24
7	30	32	35	16	19	21
8	26	28	31	14	16	19
9	20	22	24	12	14	17
10	18	20	22	10	12	14
11	15	17	19	8	10	13
12	15	17	19	7	9	11
13	11	13	15	6	8	10
14	11	12	14	5	7	9
15	9	10	12	5	6	8
16	8	10	11	4	5	7
17	8	9	10	3	4	6
18	6	7	8	3	4	5
19	4	5	6	2	3	5
20	4	5	6	2	3	4
21	3	3	4	2	2	4
22	0	1	2	1	2	3
23	0	1	1	1	2	3
24	0	1	1	1	2	2
25	0	1	1	1	1	2
26	0	0	0	0	1	2
27	0	0	0	0	1	2
28	0	0	0	0	1	1
29	0	0	0	0	0	1
30	0	0	0	0	0	1
31	0	0	0	0	0	1
32	0	0	0	0	0	1
33	0	0	0	0	0	1

Table S6. Chronology of inferred biogeographic events of mammals. The results are estimated from 1,000 replicate simulations of biogeographic histories.

Time (Ma)	*in situ* speciation			Colonization		
	25% quantile	Median	75% quantile	25% quantile	Median	75% quantile
0	124	130	136	137	142	147
1	108	113.5	120	104	109	114
2	93	98	104	77	81	86
3	69	74	78	57	61	65
4	60	64	69	44	47	51
5	43	47	51	33	37	40
6	33	37	40	27	30	33
7	25	28	31	22	25	28
8	18	21	24	19	21	24
9	11	13	16	15	17	20
10	7	9	11	13	15	17
11	7	9	11	11	13	15
12	6	8	10	9	11	13
13	4	6	8	8	9	11
14	3	4	6	7	8	10
15	2	4	5	6	7	9
16	2	3	5	5	7	8
17	2	3	4	4	6	7
18	1	2	3	4	5	6
19	1	2	3	3	5	6
20	1	2	3	3	4	5
21	1	2	3	3	4	5
22	1	2	3	2	3	4
23	1	1	2	2	3	4
24	0	1	2	2	3	4
25	0	1	2	1	2	3
26	0	0	1	1	2	3
27	0	0	1	1	1	2
28	0	0	0	1	1	2
29	0	0	0	0	1	2
30	0	0	0	0	1	2
31	0	0	0	0	1	1
32	0	0	0	0	1	1
33	0	0	0	0	1	1
34	0	0	0	0	0	1
35	0	0	0	0	0	1
36	0	0	0	0	0	1
37	0	0	0	0	0	1
38	0	0	0	0	0	1

39	0	0	0	0	0	1
40	0	0	0	0	0	1
41	0	0	0	0	0	1
42	0	0	0	0	0	1

Table S7. Chronology of inferred biogeographic events of birds. The results are estimated from 1,000 replicate simulations of biogeographic histories.

Time (Ma)	*in situ* speciation			Colonization		
	25% quantile	Median	75% quantile	25% quantile	Median	75% quantile
0	149	159	168	342	351	359
1	143	153	162.25	293	302	310
2	132	142	151	248	256	264
3	116	126	136	208	216	223
4	100	109	119	172	179	186
5	90	98	107	142	148	155
6	78	87	95	116	122	128
7	62	70	77	95	100	106
8	50	56	62	77	83	88
9	39	44	50	62	67	72
10	31	36	41	51	55	59
11	20.75	25	29	42	46	49
12	17	20.5	24	35	38	42
13	15	18	22	30	33	36
14	11	14	18	26	28.5	32
15	10	13	16	22	25	28
16	7	10	12	19	22	24.25
17	6	8	10	17	19	22
18	6	8	10	14	17	19
19	5	7	10	12	15	17
20	5	7	9	11	13	15
21	4	6	8	10	11	13
22	4	6	8	8	10	12
23	2	3	5	7	9	11
24	1	3	4	6	8	10
25	1	2	3	6	7	9
26	1	1	2	5	6	8
27	0	1	2	4	5	7
28	0	1	2	3	5	6
29	0	1	1	3	4	5
30	0	0	1	3	4	5
31	0	0	1	2	3	4
32	0	0	1	2	3	4
33	0	0	1	2	3	4
34	0	0	1	2	2	3
35	0	0	1	1	2	3
36	0	0	1	1	2	3
37	0	0	1	1	2	3
38	0	0	1	1	2	3

39	0	0	1	1	2	2
40	0	0	1	1	1	2
41	0	0	0	1	1	2
42	0	0	0	1	1	2
43	0	0	0	1	1	2
44	0	0	0	0	1	2
45	0	0	0	0	1	2
46	0	0	0	0	1	2
47	0	0	0	0	1	1
48	0	0	0	0	1	1
49	0	0	0	0	1	1
50	0	0	0	0	0	1
51	0	0	0	0	0	1
52	0	0	0	0	0	1
53	0	0	0	0	0	1
54	0	0	0	0	0	1
55	0	0	0	0	0	1
56	0	0	0	0	0	1
57	0	0	0	0	0	1
58	0	0	0	0	0	1

Table S8. Chronology of inferred biogeographic events of terrestrial vertebrates. The results are estimated from 1,000 replicate simulations of biogeographic histories.

Time (Ma)	*in situ* speciation			Colonization		
	25% quantile	Median	75% quantile	25% quantile	Median	75% quantile
0	423	437	450	556	566	578
1	396	408	422	469.75	481	491
2	357	369	382	391	400	412
3	294	305	318	325	333	343.25
4	253	264	276	269	277	287
5	210	220	232	221	230	239
6	176	187	197	184	192	199
7	142	152	161	153	161	168
8	114	122	130	128	135	142
9	86	93	100	106	113	118
10	67	73	79	88	94	99
11	52.75	58	64	74	79	84
12	46	51	56	63	67	72
13	37	42	47	53	58	62
14	31	36	39	46	50	54
15	27	31	35	40	44	48
16	22	25	29	35	38	42
17	19	22	25	30	33	37
18	16	19	22	26	29	32.25
19	13	15	18	23	25	29
20	12	15	18	20	22.5	25
21	9	12	14	17	20	22
22	7	9	11	14	17	20
23	4	6	8	13	15	18
24	3	5	7	11	13	15
25	3	4	6	9	12	14
26	1	2	3.25	8	10	12
27	1	2	3	7	9	10
28	1	2	3	6	7	9
29	0	1	2	5	6	8
30	0	1	2	4	5	7
31	0	1	1	4	5	6
32	0	0	1	3	4	6
33	0	0	1	3	4	5
34	0	0	1	2	3	5
35	0	0	1	2	3	4
36	0	0	1	2	3	4
37	0	0	1	2	2	3
38	0	0	1	1	2	3

39	0	0	1	1	2	3
40	0	0	1	1	2	3
41	0	0	0	1	2	2
42	0	0	0	1	1	2
43	0	0	0	1	1	2
44	0	0	0	1	1	2
45	0	0	0	0	1	2
46	0	0	0	0	1	2
47	0	0	0	0	1	1
48	0	0	0	0	1	1
49	0	0	0	0	1	1
50	0	0	0	0	1	1
51	0	0	0	0	0	1
52	0	0	0	0	0	1
53	0	0	0	0	0	1
54	0	0	0	0	0	1
55	0	0	0	0	0	1
56	0	0	0	0	0	1
57	0	0	0	0	0	1

Table S9. Best substitution model for each gene inferred by IQTREE.

Class	Clade	Gene	Nucleotide substitution model
Amphibia	*Scutiger*	16S rRNA	TPM2+R2
		COI	TIM2+F+I+R2
		Cytb	TPM2u+F+G4
		RAG1	K2P
Amphibia	*Amolops*	16S rRNA	GTR+F+I+R3
		COI	TPM2u+F+I+G4
		ND2	TIM+F+I+G4
Reptilia	*Lycodon*	16S rRNA	TIM2+F+I+G4
		COI	TVM+F+G4
		Cytb	TPM2u+F+I+G4
		C-mos	HKY+F
Reptilia	*Hebius*	*Cytb*	TPM2u+F+I+R3
		C-mos	HKY+F+I
		RAG1	HKY+F+R2
		NT3	TVMe+I
Reptilia	*Rhabdophis*	16S rRNA	TIM2+F+I
		Cytb	TIM2+F+I+R3
		ND4	TN+F+I+G4
		C-mos	HKY+F+I
		NT3	TPM2+G4
Reptilia	*Pareas*	*Cytb*	TIM+F+I+G4
		ND4	TN+F+I+G4
		C-mos	K2P+G4
		RAG1	HKY+F+I
Reptilia	*Gloydius*	12S rRNA	TIM2+F+I+G4
		16S rRNA	TN+F+R2
		Cytb	TPM2u+F+I+G4
		ND4	TN+F+I+G4
Reptilia	*Diploderma*	*ND2*	TIM3+F+I+G4
		C-mos	K2P
		BDNF	K2P+I

		R35	K2P+I
Reptilia	*Scincella*	12S rRNA	TIM2+F+G4
		16S rRNA	TIM2+F+I+G4
		COI	HKY+F+I+G4
		Cytb	TPM2u+F+I+R2
		ND1	TPM2u+F+G4
		ND2	TPM2u+F+R3
Mammalia	*Ochotona*	*COI*	TPM2u+F+R3
		Cytb	TPM2u+F+I+G4
		RAG1	K2P+I
		RAG2	HKY+F+I
		OXA1L	K2P+G4
		TTN	HKY+F+I
		IL1RAPL1	HKY+F
Mammalia	Erinaceidae	12S rRNA	GTR+F+I+G4
		Cytb	TIM2+F+I+G4
		RAG1	TPM3+G4
		GHR	K3P+I
		BRCA1	TPM3u+F
		vWF	HKY+F+G4
		TTR	HKY+F+I
Mammalia	Spalacidae	12S rRNA	TIM2+F+G4
		16S rRNA	TIM2+F+G4
		COI	GTR+F+I+R3
		Cytb	TIM2+F+I+G4
		ND4	TIM2+F+I+G4
Mammalia	*Niviventer*	*COI*	TPM2u+F+I
		Cytb	TIM2+F+I+G4
		D-loop	TIM3+F+G4
		IRBP	K3Pu+F+R2
Mammalia	*Rattus*	*COI*	TPM2u+F+I+G4
		Cytb	TIM2+F+I+G4
		D-loop	TPM2u+F+I+G4

		IRBP	K2P+I
Mammalia	Arvicolinae	*ATP6*	TPM2u+F+I+R5
		ATP8	TPM3u+F+I+G4
		Cytb	GTR+F+I+R6
		COI	TIM2+F+I+R5
		COII	TPM2u+F+I+R4
		COIII	TIM2+F+I+G4
		ND1	TPM2u+F+I+R5
		ND2	TIM+F+I+R4
		ND3	K3Pu+F+I+G4
		ND4	TIM2+F+I+R5
		ND5	TIM2+F+I+R6
		ND6	GTR+F+R5
Mammalia	Sciuridae	12S rRNA	GTR+F+I+R5
		16S rRNA	TIM2+F+I+R4
		ATP6	GTR+F+I+R4
		ATP8	TN+F+I+G4
		Cytb	TIM2+F+I+R7
		COI	GTR+F+I+R5
		COII	GTR+F+R5
		COIII	TIM2+F+I+R4
		ND1	TIM2+F+R5
		ND2	GTR+F+R5
		ND3	TIM2+F+I+G4
		ND4	TIM2+F+I+R5
		ND5	GTR+F+I+R5
		ND6	K3Pu+F+I+G4
Mammalia	*Talpidae*	12S rRNA	TIM2+F+I+R3
		Cytb	TIM2+F+I+R4
		ADORA3	K2P+G4
		ADRB2	TPM3u+F+R2
		APOB	TPM3u+F+G4
		APP	TVM+F+I

		ATP7A	HKY+F+I
		BCHE	HKY+F+G4
		BDNF	TPM3+I+R2
		BMI1	HKY+F+G4
		BRCA1	TIM+F+G4
		BRCA2	TPM3u+F+G4
		CREM	HKY+F+I
		DMP1	TN+F+G4
		ENAM	HKY+F+G4
		GHR	TIM3e+I+R2
		PLCB4	HKY+F+I
		RAG1	TIM3e+I+R2
		RAG2	HKY+F+G4
		TTN	TN+F+R2
		vWF	TIM3+F+G4
Mammalia	Soricinae	*Cytb*	TIM2+F+I+R5
		COI	TIM2+F+I+G4
		RAG2	HKY+F+I
		APOB	TIM3+F+G4
		BRCA1	K3Pu+F+G4
Mammalia	Cervidae	12S rRNA	TIM2+F+I+R2
		16S rRNA	TIM2+F+I+G4
		ATP6	TPM2u+F+I+G4
		ATP8	HKY+F+R3
		Cytb	TIM2+F+I+G4
		COI	TIM2+F+I+R7
		COII	TN+F+I+G4
		COIII	TIM2+F+I+G4
		ND1	TIM2+F+I+G4
		ND2	TN+F+I+R2
		ND3	HKY+F+I+G4
		ND4	HKY+F+R3
		ND5	TIM2+F+I+G4

		ND6	HKY+F+I+G4
Mammalia	Viverridae	*Cytb*	TIM2+F+I+G4
		TriI	HKY+F+G4
		IRBP	HKY+F+G4
Aves	Leiothrichidae	*Cytb*	TVM+F+I+R4
		ND2	GTR+F+R4
		ND3	K3Pu+F+I+G4
		COI	TPM2u+F+I+G4
		FIB5	TPM2u+F+R2
		MUSK3	TPM3u+F+R2
		TGFB25	TPM2+R2
		ODC6/7	TPM2u+F+I+R2
		MB2	K2P+I+R2
		GAPDH11	K2P+R2
		RAG1	HKY+F+R2
Aves	Paradoxornithidae	*Cytb*	TIM2+F+I+G4
		ND2	TIM+F+I+G4
		ND3	K3Pu+F+G4
		COI	TIM2+F+I+R2
		FIB5	TPM2u+F+G4
		MUSK3	HKY+F
		TGFB25	K2P+G4
		ODC6/7	TPM2u+F+G4
		MB2	K2P+G4
		GAPDH11	K2P+G4
		RAG1	TN+F+G4
Aves	Passeridae	*Cytb*	TPM2u+F+R3
		ND2	TIM2+F+I+G4
		MB2	K2P+G4
		ODC6/7	HKY+F+G4
Aves	Sittidae	*COI*	TPM2u+F+I+G4
		Cytb	TIM2+F+I+G4
		RAG1	K2P+I+R2

Aves	Corvidae	*Cytb*	GTR+F+R4
		COI	TIM2+F+I+G4
		ND2	TIM+F+I+G4
		ND3	TPM2u+F+I+G4
		MB2	K2P+G4
		GAPDH11	K2P
		ODC6/7	HKY+F+I
		TGFB25	K2P+G4
Aves	Fringillidae	*Cytb*	TIM2+F+I+G4
		ND2	GTR+F+I+R5
		ND3	GTR+F+I+G4
		MB2	K2P+G4
		GAPDH11	K2P+R2
		ODC6/7	TPM3u+F+I+R2
Aves	Vireonidae	*Cytb*	TPM2u+F+I+G4
		COI	TIM2+F+I+G4
		ND2	TIM2+F+I+G4
Aves	Timaliidae	*Cytb*	TPM2u+F+I+G4
		ND2	TIM+F+I+G4
		ND3	TIM+F+I+G4
		COI	TPM2u+F+I+R2
		FIB5	TPM3u+F+G4
		MUSK3	HKY+F+G4
		TGFB25	TN+F+G4
		ODC6/7	HKY+F+G4
		MB2	K2P
		GAPDH11	HKY+F+G4
		RAG1	TN+F+I
Aves	Pellorneidae	*Cytb*	TVM+F+I+G4
		ND2	GTR+F+R4
		ND3	K3Pu+F+I+G4
		COI	TIM2+F+I+G4
		FIB5	HKY+F+G4

		MUSK3	TPM3u+F+R2
		TGFB25	K3P+R2
		ODC6/7	HKY+F+G4
		MB2	K2P+G4
		GAPDH11	K2P
		RAG1	TN+F+I
Aves	Alcippeidae	*Cytb*	TPM2u+F+G4
		ND2	TN+F+G4
		ND3	K3Pu+F+G4
		COI	TIM2+F+I
		FIB5	TPM2u+F+I
		MUSK3	HKY+F
		TGFB25	K2P
		ODC6/7	HKY+F
		MB2	K2P
		GAPDH11	K2P
		RAG1	K2P
Aves	Cettiidae	*Cytb*	TPM2u+F+I+G4
		ODC6/7	TIM2+F+G4
		MB2	K2P+G4
		GAPDH11	K2P+G4
Aves	Pycnonotidae	*ATP6*	TIM+F+I+R2
	(Asian clade)	*Cytb*	TVM+F+I+G4
		ND2	GTR+F+R5
		ND3	TIM+F+I+G4
		FIB7	TPM2u+F+G4
		TGFB25	K2P+R2
		ODC6/7	TPM2u+F+G4
		MB2	TPM2+G4
Aves	Locustellidae	*Cytb*	GTR+F+I+G4
		MB2	HKY+F
		ODC6/7	HKY+F+G4
		GAPDH11	HKY+F+R2

Aves	Prunellidae	*LDH*	HKY+F+G4
		ND2	TN+F+R2
		ACO1I9	TPM3u+F
